# Prospective multicentre validation study of a new standardised version of the 400-point hand assessment

**DOI:** 10.1186/s12891-020-03303-4

**Published:** 2020-05-20

**Authors:** Michel Konzelmann, Cyrille Burrus, Colette Gable, François Luthi, Jean Paysant

**Affiliations:** 1 Department for Musculoskeletal Rehabilitation, Clinique Romande de Réadaptation suva, avenue du grand champsec, 1950 Sion, Switzerland; 2 Institute for Research in Rehabilitation, Clinique Romande de Réadaptation suva, avenue du grand champsec, 1950 Sion, Switzerland; 3 Regional institute of physical medecine and rehabilitation, 75 boulevard Lobeau, CS 34209, 54042 Nancycedex, France; 4 Department of Physical Medicine and Rehabilitation,Orthopaedic Hospital, Lausanne University Hospital, Avenue Pierre Decker, 1011 Lausanne, Switzerland

**Keywords:** Hand- 400-point hand assessment- functional evaluation-rehabilitation-MCID-MDC-reliability-validity

## Abstract

**Background:**

Hand rehabilitation needs valid evaluation tools; the 400-point Hand Assessment (HA) is an exhaustive but not standardised tool. The aim of this study was to validate a standardised version of this test.

**Methods:**

A modified version and a standardised prototype was made for this prospective validation study (four centres, three countries). Psychometric properties studied: reliability (intra-rater and inter-rater, standard error of measurement [SEM], minimum detectable change [MDC],internal consistency); content validity, construct validity with Jebsen Taylor hand function test, Q*uick*DASH, MOS-SF 36 and pain; responsiveness, using an anchor-based approach (ROC curve with area under curve, mean response change) with calculation of MCID. For SEM, MDC and responsiveness, *Quick*DASH was used for comparison.

**Results:**

One hundred and seventy-six patients with hand/wrist injuries were included between May 2013 and February 2015. One hundred and seventy were available for final analysis: 67% men; mean age 43.4 ± 13.2 years; both manual and office workers (46, 5% of each); 37% had a hand or wrist fracture.

*Reliability:* ICC intra-rater = 0.967 [0.938–0.982]; inter-rater = 0.868 [0.754–0.932]. Distribution-based approach: for 400-point HA/Q*uick*DASH: SEM = 3.48/4.52, MDC = 9.065/12.53, internal consistency of 400-point HA: Cronbach α = 0.886.

*Validity:* Content validity was good according to COSMIN guidelines. Construct validity: correlation coefficient: Jebsen-Taylor hand function test = − 0.573 [− 0.666–0.464], Q*uick*DASH = − 0.432 at T0 [− 0.545–0.303], − 0.551 at T3 [− 0.648–0.436]; MOS-SF 36 physical component = 0.395 [0.263–0.513]; no correlation with MOS-SF 36 mental component = 0.142 [− 0.009 + 0.286] and pain = − 0.166 [− 0.306 + 0.018].

*Responsiveness:* Anchor-based approach: AUC Δ400-point HA = 0.666 [0.583–0.749], AUC ΔQ*uick*DASH = 0.556 [0.466–0.646]. MCID (optimal ROC curve cut-off): 6.07 for 400-point HA, − 2.27 for Q*uick*DASH. MCID with mean response change + 12.034 ± 9.067 for 400-point HA and − 8.03 ± –9.7 for Q*uick*DASH. The patient’s global impression of change was only correlated with the Δ400-point HA.

**Conclusions:**

The 400-point HA standardised version has good psychometric properties. For responsiveness, we propose an MCID of at least 12.3/100. However, these results must be confirmed in other populations and pathologies.

**Trial registration:**

This study was retrospectively registered into ISCTRN registry (Number ISRCTN25874481) the 07/02/2019.

## Background

Injuries of the hand and wrist are very common and are responsible for significant costs which comprise direct costs (surgery, rehabilitation) and indirect costs (days off work, compensations). For example, in Switzerland (8 million inhabitants) in 2008, 160,115 injuries of the hand/wrist/fingers occurred costing 419 million Swiss francs (estimation in 2012). They represented 24, 4% of the total injuries in 2008 in Switzerland [1].

Many of these injuries occurred in the active working population and could lead to permanent sequela and disability. In rehabilitation and particularly in vocational rehabilitation, useful evaluation tools are needed with good psychometric properties to establish a treatment plan based on precise goals, to measure improvements in the rehabilitative process and to measure functional capacities at the end of treatment and to prove treatment efficacy among providers [2]. In rehabilitation, the international classification of functioning (ICF) model is used [3] from the World Health Organization (WHO); it is a biopsychosocial view of functioning organized in two parts. The first part (functioning and disability) comprises body functions and structure, activities and participation. The second part (contextual factors) comprises environmental and personal factors.

A good evaluation process needs to cover all of the ICF dimensions [4–9]. For example, pain is a body function and may be evaluated by a visual analogue scale (VAS), as is range of motion, which is evaluated with a clinical exam and a goniometer. “Activities” refers to the execution of a task or “to do something” and participation reflects the implications to the patient in real life (work and leisure activities for example).

To measure activities and participation, different tools are available. First, the patient-reported outcomes (PRO) have been commonly used for at least 20 years; for the upper limb, the gold standard is the Disabilities Arm Shoulder and Hand (DASH) questionnaire [10]. Many other questionnaires are available [11]. In 2017, Kus [9] published a brief ICF core set for hand conditions (ICF Hand A) with different evaluations for screening all the dimensions of the ICF. To assess activities and participation, they proposed using a functional capacity evaluation (FCE) [12], Moberg pick up test [13] and the DASH. For patients with a recent injury or surgery, administration of a FCE may not be feasible. Moreover, the FCE is time-consuming and not only focused on the upper limbs. Less time-consuming and more specific evaluation tools are needed. Many hand evaluation tests have become available since 1965 [14]. Despite insufficient psychometric properties [15], the most-used test in the literature is the Jebsen-Taylor hand function test (JTHFT) [16]. It is standardised with seven tasks and easy to administer with norms. It is mainly used in neurology and less used for orthopaedic injuries. Two other tests have good psychometric properties: the Test d’Evaluation des Membres supérieurs des Personnes Agées, or upper-extremity performance test for the elderly (TEMPA) [17–19], and the Sequential Occupational Dexterity Assessment (SODA) [20] and its short version [21], but they were developed for the elderly population (TEMPA) or for patients with rheumatoid arthritis (SODA) and are also used in neurology. Schoneveld [6] recommends using the functional dexterity test [22, 23], which is not exhaustive for all hand function.

A more comprehensive hand function test is required. The 400-point Hand Assessment (400-point HA, bilan 400 points in French) test was designed and validated by a French team of occupational therapists and physical medicine and rehabilitation (PMR) physicians in Nancy, France, from 1985 to 1997 [24–27]. It was inspired by different tests published previously [16, 28, 29]. The item choices were made by the team, based on clinical practice, specifically to cover hand function and to target the overall functional deficit. No patients were involved in the choice of items but they tested the different versions. It’s a quantitative and evaluative test used in pathologies of the musculoskeletal system in adults. It covers the first part of the ICF well, with 1 item for body structures,12 items for body functions and 21 items for activities and participation [30]. There are four tests, each based on 100 points: function of the hand (mobility with 12 movements), strength (five dynamometers), handling and displacement of objects (20 objects), and function with both hands (20 tasks of daily living). The final result is the sum of each test divided by 4 and is expressed as a percentage of 100. The protocol is well described [26]. The test is unidimensional [25, 31]. A principal component analysis was made for the injured hand and showed three factors; each factor explained 44, 10 and 4% of the total variance, respectively [25]. For the authors, three arguments in favour of test unidimensionality were: the first factor being greater than 20%, only 6 of the 67 items having a weak correlation (< 0.30) with first factor, and finally the ratio between the first and second factor being 4.4 (it should be > 2). The results were the same for the non-injured hand and for the total analyses. Cronbach’s α value at 0.97 testified to good congruence of the items, and the Rasch model was also consistent with the unidimensionality; only one item was rejected by the U test of Molinar [25]. After removal of some redundant items, the final version of the 400-point HA was published in 1996 [26, 27]. The inter-rater and the test retest reliability were studied and found to be good [24, 31, 32], with intraclass correlation coefficients (ICC) of more than 0.95 and 0.82, respectively.

The 400-point HA is used mainly in France, the French-speaking part of Belgium and Switzerland, Portugal and in some Spanish-speaking countries. The Portuguese translation is available and was done by a Portuguese occupational therapist (OT) who spoke French. The Spanish version was translated and validated in 2014 by a team of OTs in Chile [31]. For this article, we translated the protocol in English.

Because of non-standardised equipment, a complicated strength test, partial data on construct validity and responsiveness, it was decided to build a modified and standardised version of the 400-point HA and validate it.

The aim of this prospective, multicentre study was to modify and simplify the 400-point HA, to build a standardised prototype and to validate its psychometric properties: reliability (intra- and inter-rater reliability, measurement error, MDC and internal consistency), content and construct validity and responsiveness.

## Methods

### Construction of the 400-point HA, version 2

The first stage of the study was to modify the original version of the 400-point HA. This was done by the team of occupational therapists (Mrs. Gable et al.) of the Institut Régional de Réadaptation, Nancy, France. The modifications were based on the opinion of a team experts. The protocol is available as supplementary material in French, Portuguese and English (see Additional files 1, 2 and 3).

In the first part (mobility of the hand), the wrist flexion/extension was added to give an overall view of the entire function of the hand. Fourteen global movements are tested in this first part.

In the second part, assessing strength, three dynamometers were removed for practical reasons; two of them were outdated (the Collins dynamometer and the vigorimeter), and one was homemade. Administration time should be improved by using only two dynamometers, one for grip strength (Jamar® Dynamometer, at the second notch), and one for pinch strength between the thumb and index finger (Jamar® pinch-meter dynamometer). Three measures are made (alternately) on both sides and the mean is calculated. For the norms of the two dynamometers, it was decided to use the Swiss norms published in 2010 [33].

For the third part (handling and displacement of objects) objects were standardised for size and weight. Two objects were modified: a cylinder of 7.5 cm was added and a trunnion removed (expert team opinion). Twenty actions of prehension are tested.

For the fourth part (function with both hands), the material was also standardised and some minor modifications of objects were done: a 20 mm nut was removed, and the purse and medication tube with crimped cap were updated (expert team opinion). Twenty bimanual actions are available for this test.

### Realisation and production of four prototypes of the 400-point HA

Four standardised prototypes were built with the help of an engineering school in Nancy, France, for some objects (cube, cylinder). The four prototypes were similar and were given to each study centre.

### Protocol of the study (see Fig. 1)

This was a prospective validation multicentre study involving four centres in three countries. Two French centres (Institut régional de médecine physique et de réadaptation, centre Louis Pierquin, Nancy; centre médical Rocheplane, Saint Martin d’Hères); one Portuguese centre (Hospital particular do Algarve Gambelas, Faro) and one Swiss centre (Clinique romande de réadaptation suva, Sion). The inclusion period was expected to run from May 2013 to May 2014 and the results analysis from May 2014 to September 2014. Two hundred and fifty patients were planned to be included: 100 at Nancy and 50 at each of the other centres. For sample size, no clear scientific recommendations are available [34]. A sample size of approximately 100 is considered sufficient for multivariate analysis [34]. Based on other studies of hand test validations [15, 16, 18, 20, 22], the number of planned subjects is greater in this study. For the original version of the 400-point HA, Luquet [25] administered the test to 173 patients.

**Fig. 1:**
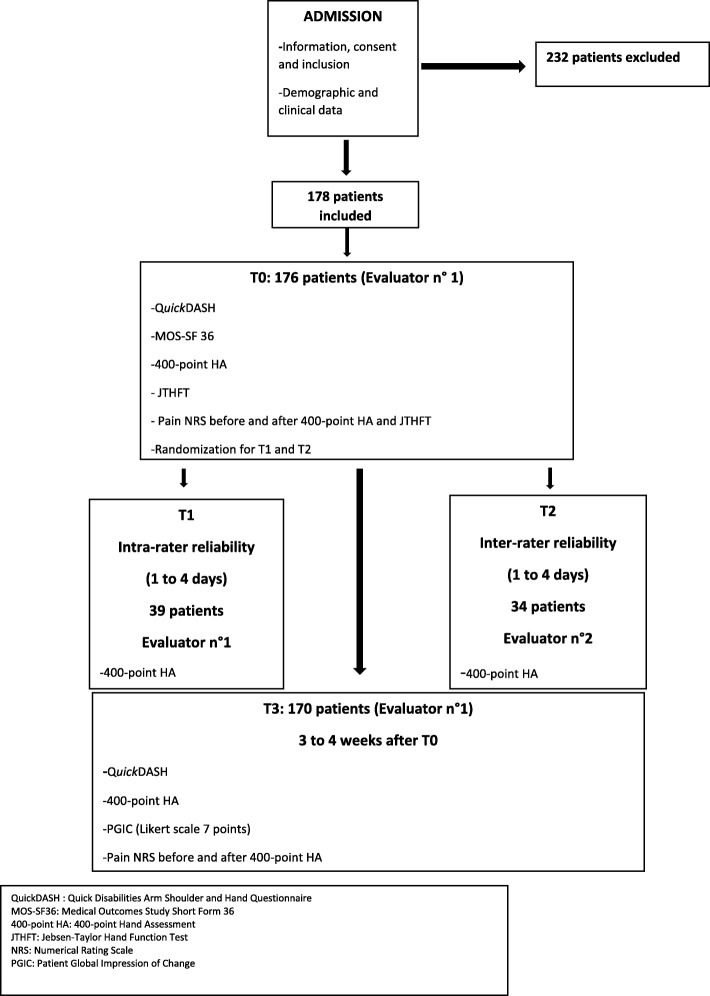
summary and flow chart of the study

#### Inclusion criteria

Patients over 18 years old, with unilateral traumatic hand impairment (fractures, tendon lesions, wounds, peripheral traumatic neuropathy, Complex Regional Pain Syndrome [CRPS]) were included, whether hospitalized or not.

Exclusion criteria: Patients under 18 years old, bilateral hand impairment, central neurological impairment, severe psychiatric disorders, patients unable to fulfil the questionnaires or to understand the instructions of the 400-point HA, impossibility to plan the 400-point HA at admission, contraindication for the 400-point HA at admission (recent surgery).

Data at admission: Patients were screened for inclusion and exclusion criteria and received an information sheet. Patients who fulfilled the inclusion criteria and were willing to participate signed an informed consent. If the patient met the exclusion criteria or refused to participate, he or she was assigned to the group of excluded patients. The following data were collected: birth date, sex, profession, school education (under nine years or over nine years), pathology and type of lesion, hand dominance, hand side involved, injury date, surgical intervention date, rehabilitation start date, hospitalized or ambulatory treatment, work incapacity, pain numerical rating scale (NRS) maximum and average in the past week.

The measurement properties of the 400-point HA were planned according to COSMIN guidelines consensus [35, 36]. Reliability, validity and responsiveness were to be studied.

**Reliability** reflects “the extent to which scores for patients who have no change are the same for repeated measurement under several conditions”. It includes intra-rater and inter-rater reliability, measurement error and internal consistency [36].

### Intra- and inter-rater reliability

Some patients after inclusion and first evaluation (T0) were randomly assessed for intra-rater reliability (T1) or for inter-rater reliability (T2). These patients were not the same. The number of patients for T1 and T2 was estimated at 40 patients in each group. Because no international recommendations are available, this number was chosen based on previous studies about the original 400-point HA [31, 32] and on other studies of functional hand tests [18, 20, 22]. The intraclass correlation coefficient (ICC) of the original version of the 400-point HA was good with 20 to 30 patients, so we expected the same result with 40 patients in each group. The 400-point HA was administered 1–4 days after T0, by the same evaluator (T1) and by another evaluator (T2). This interval was chosen because no improvement was expected in this short span of time.

For statistical analysis, *intra-rater and inter-rater reliability* were assessed with the intraclass coefficient correlation (ICC) [37] and the Bland-Altman method [38]. The mean of the two 400-point HA tests (1–4 days) was calculated between T0 and T1 for intra-rater reliability, and between T0 and T2 for inter-rater reliability. The ICC was considered excellent if r > 0.91, good if r was between 0.71 and 0.9, medium between 0.51 and 0.70, weak between 0.31 and 0.50 and no correlation if r <  0.30 [37].

We expected the ICC to be > 0.8 for each reliability.

### Measurement error

The s*tandard error of measurement (SEM)* was measured, which is the variation in scores due to the unreliability of the scale used. A SEM value is based on the standard deviation (SD) of the sample and the reliability of the measurement instrument, expressed as SEM = SD from the first test x (√(1-ICC)) [39]. The ICC is the result of the intra-rater reliability of the 400-point HA. *Minimum detectable change (MDC)* was also calculated. It refers to the least amount of change outside of error that reflects true change by a patient between two time points, rather than a variation in measurement. The formula for MDC is MDC = 1.96 X SEM X √2. The value 1.96 was chosen to achieve a confidence interval of 95% [40].

### Internal consistency

Internal consistency refers to “the homogeneity of a measure in terms of how the items of an instrument group together into units” [41]. Internal consistency of the 400-point HA was determined by Cronbach’s α, which is a general coefficient of homogeneity between items. Values for α can range from zero (no internal consistency) to one (perfect internal consistency). A value above 0.8 is considered acceptable [37]. Cronbach’s α was calculated for each test and for the total score of the 400-point HA.

Cronbach’s α was hoped to be > 0.8 for all the tests and for the total 400-point HA.

**Validity** is “the degree to which a measure instrument measures the construct it purports to measure” [36]. It includes content validity, construct validity and criterion validity.

***Content validity*** is the degree to which the content of an instrument is an adequate reflection of the construct to be measured. Usually it is an expert opinion (the authors) and COSMIN guidelines were used for it [35].

***Construct validity*** is “the degree to which a score of an instrument is consistent with hypotheses based on the assumption that the instrument validly measures the construct to be measured” [36]. It could be studied by testing different hypotheses. For the 400-point HA, which is a hand function assessment, construct validity must be studied with instruments designed for hand function and which comprise activities of daily living in particular.

Two questionnaires were used, the Quick Disabilities Arm Shoulder and Hand questionnaire (Q*uick*DASH) ( [42, 43] and the Medical Outcomes Study-Short Form (MOS-SF) 36 [44–46] in validated French and Portuguese versions; the Jebsen-Taylor hand function test (JTHFT), another hand function test [16]; and the pain numerical rating scale (NRS).

The Q*uick*DASH has been used for many years worldwide, and many translations are available. It is easy to administer and less time-consuming, with only 11 questions, reason why it was chosen. It was administered at T0 and T3.

The MOS-SF 36 is a validated questionnaire about quality of life (QOL), with different components. For our study, the physical component (PC) and the mental component (MC) of the questionnaire were used for comparison. The questionnaire was administered at T0.

Because no gold standard test for hand function was available, the JTHFT was chosen as comparator for the 400-point HA. It is standardised, commercialised in French [47] and Portuguese [48] versions and is easy to administer. Each of the seven tests of the JTHFT is timed. The two tests were administered at T0 by very seasoned occupational therapists, six at Nancy, five at Saint Martin d’Hères, three at Sion and one at Faro. The protocol at T0 was: NRS from 0 to 10 before administration of the 400-point HA, administration of the 400-point HA in one session (30 to 40 min), NRS from 0 to 10 after, rest for 10 min, NRS from 0 to 10 before JTHFT, administration of JTHFT [16] and NRS from 0 to 10 after. For comparison, mean pain was used at T0 after the test.

For statistical analysis, Pearson’s correlation coefficients (r) was used between the 400-point HA and the Q*uick*DASH, MOS-SF 36 physical component (PC) and mental component (MC) and mean pain NRS at T0. Spearman’s correlation coefficients (r) were used between the 400-point HA and JTHFT at T0. For statistical analysis, the times of the seven tests were compared to the norms of the JTHFT and summed up for each hand (injured and non-injured) and compared to the score of the 400-point HA. Only the time of the JTHFT and 400-point HA scores for the injured hand was compared.

Correlation was considered excellent if r > 0.91, good if r was between 0.71 and 0.9, medium between 0.51 and 0.70, weak between 0.31 and 0.50 and no correlation if r <  0.30 [37]. Ninety-five percent confidence intervals for the correlation coefficients were calculated by means of Fisher’s transformation*.*

### Our hypotheses were

Expected good correlation between JHFT and 400-point HA

Expected medium correlation between *Quick*DASH and 400-point HA

Expected weak correlation between MOS-SF 36-PC and 400-point HA

No expected correlation between SF 36 MC and 400-point HA

No expected correlation between pain NRS and 400-point HA

***Criterion validity:*** In the absence of a reasonable gold standard, COSMIN does not recommend studying this validity [35].

**Responsiveness** is **“**the ability of an instrument to detect change over time in the construct to be measured” [36]. No consensus exists for its calculation [49]. The anchor-based approach was chosen which is the method of choice for numerous authors*.* The anchor used was the level of improvement based on the patients’ subjective feelings reported on the Patient Global Impression of Change (PGIC) a Likert scale of seven items [49]. It asks patients how their health status has improved following treatment (1 = worse than ever; 2 = much worsened; 3 = slightly worsened, 4 = unchanged, 5 = slightly improved; 6 = much improved; 7 = completely improved). The score was treated as a binary outcome (scores of 6 or 7 = “improved” versus scores of 1 through 5 = “not improved”). Minimal clinically important difference (MCID) of the 400-point HA was estimated using the receiver operating characteristic (ROC) method by comparing patients with and without improvement. The optimal cut-off on the ROC curve was determined by using the optimal Youden’s index [50]. The mean change of score according each patient’s response level on the PGIC was also calculated. Revicki [51] recommended that the MCID must be based primarily on appropriate patient-based anchors that are correlated at ≥0.30 with the patient-reported outcome. To reinforce the validity of our anchor, the Spearman correlation coefficient was calculated between the PGIC and delta scores. For the ROC curve, an AUC > 0.70 was expected for the 400-point HA [52].

The effect size (ES) and the standardised response mean (SRM) were not calculated because COSMIN guidelines recommended not to use this distribution-based approach for responsiveness [35].

As a comparison, the MCID of the Q*uick*DASH was also estimated. The ICC for the test-retest of the DASH was 0.95 [42] for the French version.

All calculations were performed using the Stata 16.0 statistical package for Windows.

#### Interventions between T0 and T3

Therapeutic interventions were different in centres and were not standardised. All the patients in all centres were treated by an occupational therapist (OT). Some patients were treated every day (for example, inpatients from Sion, Nancy and Saint-Martin D’Hères centres), some others two or three times a week (outpatients from Faro, Nancy and Saint-Martin d’Hères). Other therapies were used for inpatients: physiotherapy and group therapy (balneotherapy, fitness, and adapted physical activities) centered not only on hand pathologies but on general physical well-being. Pain medications were taken if necessary.

##### Data summary

All the data were summarised in an anonymous booklet with a number for each patient. When the booklet was finished, each centre sent it to the Clinique Romande de Réadaptation suva in Switzerland and the booklet was verified and scanned directly in an anonymous Excel file.

##### Ethics

The current study was approved by the local ethical committee of the four evaluation centres and was conducted in accordance with the Helsinki Declaration. For Clinique Romande de Réadaptation, the Commission Cantonale Valaisanne d’Ethique Médicale (n° CCVEM 050/12); for France (two centres) Comité d’Ethique de l’Institut Régional de Médecine Physique et Réadaptation and for Portugal Comissao de Etica para a Saude do Hospital Particular de Algarve (n° 1/2013) gave consent. All patients received an information sheet about the study and signed an informed consent if they were included.

## Results

Descriptive statistics: Patients were included from May 2013 to February 2015; 410 patients were eligible. One hundred and seventy-eight patients were included; 176 were available at T0 and 170 at T3 for the final analysis. Two hundred and thirty-two were excluded for several reasons. The excluded group differed by fewer cases of CRPS and fewer workers than among the included group. The characteristics of the two groups are shown in Table 1. Our patients were mainly men (67%), middle-aged (median 43 years old), workers (46.5%) and employees (46.5%) and in total incapacity to work (82%). The main lesions were hand or wrist fractures (37%) and 28% had an associated CRPS. The characteristics of patients between centres are available in supplementary material (see Additional file 4). The following differences were found: the Faro centre had more women, more employees, fewer cases of CRPS, and more acute patients and 400-point HA results were better at T0 than in other centres. The Saint Martin d’Hères Centre had no CRPS patients. The Sion centre had more workers, a longer interval between injury and T0 and a higher pain level than other centres. The Sion and Nancy centres were relatively similar.

**Table 1:** Characteristics of the two populations

	PATIENTS INCLUDED	PATIENTS EXCLUDED
**Number of patients (total)**	176	235
Nancy	66	88
Sion	58	81
Saint Martin d’Hères	32	49
Faro	20	17
**Causes of exclusion**	NA	
Age < 18 years		6 (2.5%)
Bilateral injury		39 (16.6%)
Central neurological injury		4 (1.7%)
Psychiatric comorbidity		8 (3.4%)
Planning impossible		49 (20.9%)
Fluency in French or Portuguese		39 (16.6%)
Refusal		8 (3.4%)
Contra indication to 400-point HA		82 (34.9%)
**Men**	118 (67.05%)	157 (66.8%)
**Women**	58 (32.95%)	78 (33.2%)
**Age (years)** Median + − SD	43.4 + − 13.2	44.3 + − 15.3
**School education (*n*** **= 176)**		
< 9 years	50 (28.4%)	
> 9 years	126 (71.6%)	
**Employment status (n = 176)**		
Employee	135 (76.7%)	
Unemployment	25 (14.3%)	
Retired	12 (6.8%)	
Student	2 (1.1%)	
Housewife	2 (1.1%)	
**Occupation (*n*** **= 172)**		
Worker/farm worker	80 (46.5%)	80 (34%)^a^
Employee (office)	80 (46.5%)	70 (29.8%)^a^
Manager/self employed	6 (3.5%)	16 (6.8%)
Other	6 (3.5%)	58 (29.4%)^a^
**Work capacity (*n*** **= 175)**		
0%	143 (81.7%)	
Partial	8 (4.6%)	
Complete	24 (13.7%))	
**Hand dominance (n = 176)**		
Right handed	162 (92%)	
Left handed	12 (6.8%)	
Ambidextrous	2 (1.2%)	
**Hand injured (n = 176)**		
Right	93 (52.8%)	
Left	83 (47.2%)	
**Diagnostic (n = 176)**		
Fracture hand/wrist	65 (36.9%)	62 (26.4%)
Sprain/luxation	18 (10.3%)	22 (9.4%)
Isolated tendon	23 (13%)	25 (10.6%)
Complex lesion (> 3 structures)	26 (14.8%)	49 (20.8%)
Others	44 (25%)	77 (32.8%)
**CRPS (n = 176)**		
Yes	50 (28.4%)	36 (15.3%)^a^
No	126 (71.6%)	199 (84.7%)
**Interval between injury and T0 (*n*** **= 163)** Days median + − SD	233 + − 460.3	
**Number of surgery (n = 176)** median + − SD	1 + − 1.22	
**Interval between last surgery and T0 (*n*** **= 143)** Days Median + −SD	109 + − 175.2	
**Pain at T0 before test (n = 175)** median + − SD		NA
Average pain	3 + − 2.11	
Maximum pain	5 + − 2.76	
**Pain at T3 before test (*n*** **= 170)** median + − SD		NA
Average pain	3 + − 2.17	
Maximum pain	5 + − 2.85	
**Q*uick*DASH/100** median + − SD		NA
T0 (n = 176)	52.2 + − 20.2	
T3 (n = 170)	40.9 + − 19.5	
**400-point HA (in %) at T0 (n = 176)** median + − SD		NA
**Total score**	**60.3 + − 19.2**	
Test n°1	63.8 + −16.9	
Test n°2	48.8 + − 25.3	
Test n°3	60 + − 25.5	
Test n°4	65 + −21.9	
**400-point HA (in %) at T3 (n = 170)** median + − SD **Total score**	**72.6 + − 18.7**	NA
Test n°1	71.9 + − 16.9	
Test n°2	57.1 + − 24.9	
Test n°3	80.8 + − 22.9	
Test n°4	83.3 + −21.05	

*SD* standard deviation, *CRPS* complex regional pain syndrome, Q*uick*DASH Quick Disabilities Arm Shoulder and Hand, *400-point HA* 400-point Hand Assessment, *NA* not applicable

^a^ Statistically significant difference

### Reliability: results are presented in Fig. 2 and Table 2

*Intra-rater reliability* was made for 39 patients between T0 and T1 (mean = 3.23 days). The 39 patients differed from the others only by their pain at T0 being slightly lower (*p* = 0.008). The ICC was excellent at 0.967 [0.938–0.982] for the total score of the 400-point HA. The ICC for each test of the 400-point HA ranked from 0.974 (test n°4), 0.972 (test n°3); 0.810 (test n°2) to 0.752 (test n°1). With the Bland–Altman method, the mean difference between tests at T0 and T1 was − 2.5, with 95% upper and lower limits of agreement of − 10.33 and 5.32, respectively, including three outliers.

**Fig. 2:**
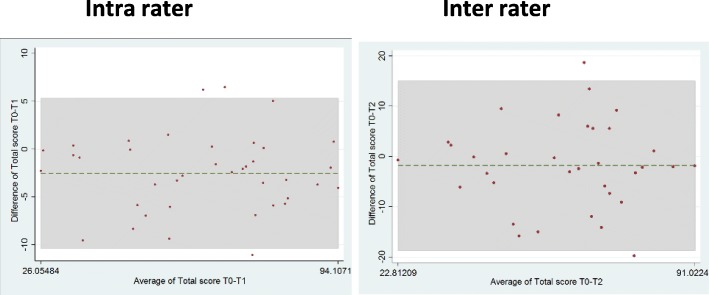
Bland Altman method for intra-rater and inter-rater reliability. Intra-rater and inter-rater reliability with the Bland–Altman method. The middle. Line represents the mean difference between the two tests. The upper and lower lines represent the upper and lower limits of agreement, i.e. mean difference +/− 1.96 SD of the differences respectively

**Table 2:** Reliability of 400-point HA (all centers)

	INTRA RATER (***n*** = 39)	CONFIDENCE INTERVAL 95%	INTER RATER (***n*** = 34)	CONFIDENCE INTERVAL 95%	INTERNAL CONSISTENCY	SEM	MDC
**TOTAL SCORE 400-point HA**	0.967	[0.938–0.982]	0.868	[0.754–0.932]	0.886	3.48	9.65
**TEST n°1**	0.752	[0.577–0.861]	0.815	[0.663–0.803]	0.842	NA	NA
**TEST n°2**	0.810	[0.668–0.895]	0.920	[0.847–0.959]	0.543	NA	NA
**TEST n°3**	0.945	[0.899–0.971]	0.784	[0.613–0.885]	0.968	NA	NA
**TEST n°4**	0.974	[0.952–0.986]	0.700	[0.481–0.837]	0.944	NA	NA

*Inter-rater reliability* was made for 34 patients between T0 and T2 (mean = 3.35 days). The 34 patients differed from the others only by maximum and mean pain at T0 which was also slightly lower (*p* = 0.088 and *p* = 0.0065 respectively). The ICC was good at 0.868 [0.754–0.932] for the total score of the 400-point HA. The ICC for each test of the 400-point HA ranked from 0.920 (test n°2), 0.815 (test n°1) and 0.784 (test n°3) to 0.700 (test n°4). With the Bland–Altman method, the mean difference between tests at T0 and T2 was − 1.8, with 95% upper and lower limits of agreement of − 18.68 and 15.05, including two outliers.

#### Measurement error

SEM and MDC were calculated for all centres and by each centre. The results of all centres were available for 169 patients. For the 400-point HA SEM, the ICC of 0.967 (intra-rater reliability) was taken. The SEM was at 3.48, the MDC at 9.65. There were some disparities between centres for SEM and MDC. For the Q*uick*DASH SEM, the ICC of 0.95 was taken [42]. The SEM was at 4.52, the MDC at 12.05.

#### Internal consistency

The Cronbach’s α for the entire 400-point HA was 0.886 and, for each test, it was 0.842 for test n°1, 0.543 for test n°2, 0.968 for test n°3 and 0.944 for test n°4.

### Validity: results are presented in Table 3

#### Content validity

COSMIN recommendations [35] about content validity were applied (Box D of recommendations). The 400-point HA items were relevant for the construct to be measured (D1) (global functional evaluation of the hand comprising mobility, strength, different prehension, bi manual activities), study population of patients with orthopaedic lesions of the hand (D2) and evaluative purpose of the test (D3). For the comprehensiveness of the items (D4), we think that all the relevant aspects of the construct were covered by the items and the domains. As is said in the introduction, ICF domains of body functions and structure, activities and participation were covered by the 400-point HA [30]. The conceptual basis of the 400-point HA was to appreciate global hand functional capacity and to target parameters that are responsible of the global deficit. We think that this aim was achieved with this test. For the last requirements (D5), we think that the design and methods of the study have no important flaws. We conclude that the 400-point HA has good content validity.

**Table 3:** Construct validity of 400-point HA

Variable	Correlation	Confidence interval 95%	***P*** value
**Total score of 400-point HA vs total score JHFT at T0**	−0.573	[−0.666–0.464]	< 0.001
Score test n°1 of 400-point HA vs total score JHFT	−0.441	[− 0.554–0.312]	< 0.001
Score test n°2 of 400-point HA vs total score JHFT	− 0.560	[− 0.655–0.448]	< 0.001
Score test n °3 of 400-point HA vs total score JHFT	−0.481	[− 0.588–0.358]	< 0.001
Score test n°4 of 400-point HA vs total score JHFT	− 0.445	[− 0.557–0.317]	< 0.001
**Total score 400-point HA vs total score Q*uick*DASH**	At T0–0.432 At T3–0.551	[− 0.545–0.303] [− 0.648 -0.436]	< 0.001 < 0.001
**Total score 400-point HA vs MOS-SF 36 PC at T0**	0.395	[0.263–0.513]	< 0.001
**Total score 400-point HA vs MOS-SF36 MC at T0**	0.142	[−0.009 + 0.286]	0.024
**Total score 400-point HA vs mean pain at T0**	−0.166	[− 0.306–0.018]	0.028

Correlations: Spearman was use for correlations between 400-point HA and JHFT, 400-point HA and mean pain (distribution not normal). Pearson was used for 400-point HA and Q*uick*DASH and MOS-SF 36 MCS/PCS

JHFT = Jebsen Hand Function Test

400-point HA = 400-point Hand Assessment

Q*uic*kDASH = Quick Disabilities Arm Shoulder Hand

MOS-SF36MCS/PCS = Medical Outcomes Study Short Form 36 Mental Component Score/Physical Component Score

#### Construct validity

At T0, 173 patients were available.

With JTHFT, the correlation with the total 400-point HA score was medium at − 0.573 [− 0.666–0.464]. Of the four tests of the 400-point HA, the second test (force) correlated the best with the JTHFT, also with a medium correlation. The three others had a weak correlation.

With Q*uick*DASH, the correlation at T0 was weak at − 0.432 [− 0.545–0.303] and at T3 was medium at − 0.551 [− 0.648–0.436].

With the MOS-SF 36 physical component (PC), the correlation was weak at 0.395 [0.263–0.513].

With the MOS-SF 36 mental component (MC), the correlation was at 0.142 [− 0.009 + 0.286] (no correlation).

With mean pain, the correlation was at − 0.166 [− 0.306–0.018] (no correlation).

### Responsiveness: results are presented in Table 4 and Fig. 3

#### Anchor-based approach

Patients were categorised in two groups, according to their answers to the anchor question: 103 patients (60.6%) reported an improvement (i.e., much improved or completely improved, according to the PGIC), and 67 patients (39.4%) reported no improvement.

**Table 4:** Responsiveness of 400-point HA and Q*uick*DASH

	400-point HA total score	Q***uick***DASH
**AUC**_**roc**_ **[Confidence interval of 95%]**	0.666 [0.583–0.749]	0.556 [0.466–0.646]
**MCID ROC curve cut off**	6.07 (Sensitivity: 72.82% and specificity 53.03%)	−2.27 (Sensitivity: 91.26% and specificity 16.42%)
**MCID mean response change in improved group**	12.3 + − 9.67	−8.3 + − 9.7

*AUC* Area Under Curve

*ROC* Receiver Operating Characteristic

*MCID* Minimum Clinically Important Difference

**Fig. 3:**
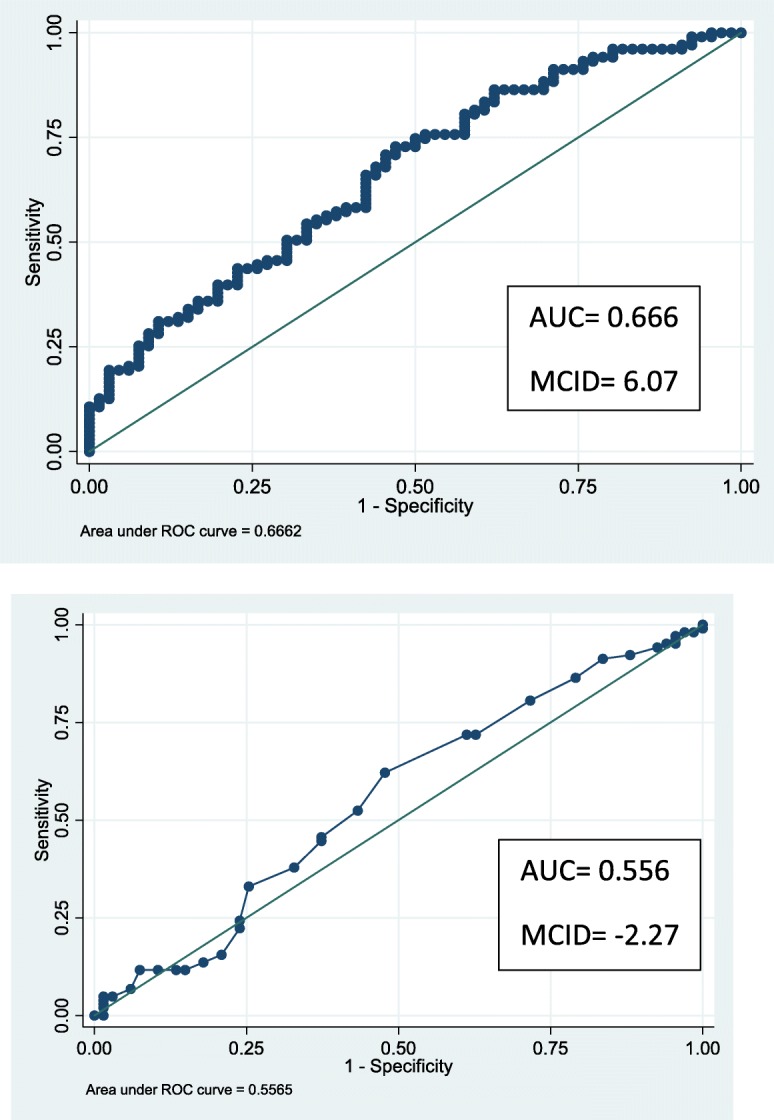
MCID Anchor based approach with ROC curve and AUC of 400-point HA and *Quick*DASH. ROC curve of 400-point HA. ROC curve of Q*uick*DASH. ROC: Receiver Operating Characteristic. AUC: Area Under Curve

ROC curves for the progression of the scores (Δ-400-point HA T0–T3 and Δ-Q*uick*DASH T0–T3) showed an AUC of 0.666 [0.583–0.749] for the Δ-400-point HA and 0.556 [0.466–0.646] for the Δ-*Quick*DASH (see Figure n°3).

The MCID was estimated by the optimal ROC cut-off value at 6.07 for the 400-point HA (sensitivity: 72.82% and specificity 53.03%) and − 2.27 for the Q*uick*DASH (sensitivity: 91.26% and specificity 16.42%).

In the improved group, the Δ-400-point HA and Δ-*Quic*kDASH were + 12.34 ± 9.67 and − 8.3 ± 9.7, respectively. In the unimproved group, the Δ-400-point HA and Δ-*Quick*DASH were 5.63 ± 9.26 and − 3.2 ± 9.8, respectively. The PGIC was correlated with the Δ-400-point HA (Spearman rho = 0.310) and not correlated with the Δ-Q*uick*DASH (Spearman rho = 0.110).

## Discussion

The purpose of this prospective study was to validate a standardised and modified version of the 400-point HA. This test has good psychometrics properties, similar to the original 400 -point HA. Our hypothesis for both intra-rater and inter-rater reliabilities and internal consistency were confirmed. The content validity was good and the 400-point HA covers well the most important hand functions (mobility, strength, prehension and object displacement and bi manual activities) and the first part of ICF (body functions and structures, activities and participation) which are very important in rehabilitation. For construct validity, four hypotheses from the five suggested were strictly fulfilled, while the last one was partly verified. Lastly we were able to propose an MCID which is a very important element for daily practice. Of the ten hypotheses put forward, eight were fulfilled (80%). Due to the large number of hypotheses that were tested, we cannot exclude that some observed associations were actually type 1 errors. Nevertheless, given the large observed values and the reasonable width of confidence intervals, we are confident about the validity of our results.

In our opinion, this modified version of the 400-point HA is more comprehensive than other existing instruments [13, 14, 16, 17, 20, 22, 28, 29, 53] for hand function evaluation.

We resume in Table 5, according to Rudman [41] the properties of the 400-point HA.

**Table 5:** summarize of the evaluation framework of 400 point HA according to Rudman and Hannah [41]

CATEGORIES	400 point HA
**Category 1 : Clinical utility**	
**A) Clinical applicability**	
a. Type of results	Quantitative and qualitative
b. Type of tasks	Covers CIF (21 items for activities and participation)
i. Representative of ADL	Yes
ii. Unilateral	Yes
iii. bilateral	Yes
c. Administration method	Observation
d. Interpretation of results	Comprehensive: four sub-tests (mobility, strength, prehension and displacement of objects, bi manual function), quality of tasks, percentage of each sub-tests compare to the non-injured hand.
**B) Specificity**	Orthopaedics hand injury or pathology, adults
**C) Availability**	
a. Prefabricated	Yes
b. Public domain	Yes
c. Language	French/English/Portuguese/Spanish
d. Cost	In progress
**D) Time demands**	
a. Administration/scoring/interpretation	30 to 45 minutes
b. Training for evaluator	Yes, important at the beginning
**E) Acceptability to patients**	
a. Purpose understand by patients	Yes
b. Appropriate for adults	Yes
c. Language	French/Portuguese/Spanish/English
d. Cultural applicability	Yes used in French and Spanish speaking countries, in Portugal for more than 10 years
**Category 2 : Standardization**	
A) Instructions	
a. Administration	Yes very precise manual
b. Scoring	Yes very precise manual
c. Interpretation	No, the comparison is with the non-injured hand which is considered the normal hand
B) Equipment prefabricated	Yes
**Category 3 ; Purpose**	
A) Descriptive	Yes (comparison with the normal hand)
B) Evaluative	Yes (can be done at the beginning and at the end of the therapy)
C) Predictive	No
**Category 4 : Psychometric properties**	
A) Items construction	Broad range of items, evaluative and descriptive, items selection by Rasch analysis and principal component analysis
B) Reliability	
a. Inter-rater	ICC at 0.868
b. Intra-rater	ICC at 0.96
c. Test-retest	No for this version
d. Internal consistency	Good Cronbach α at 0.886
C) Validity	
a. Content	Yes COSMIN recommendations fulfilled
b. Construct	Medium correlation with JHFT (-0.573), weak to medium correlation with QuickDASH (-0.432 to -0.559), weak correlation with MOS-SF 36 PC (0.395), no correlation with MOS-SF36 MC and mean pain (0.142 and -0.166 respectively)
c. Criterion	Not applicable no gold standard
D) Responsiveness	MCID of 12 points proposed in our study population, not study in other populations
E) Norms	
a. Availability	Yes for the second sub-test (strenght) otherwise the normality is the non-injured hand which is 100% by definition. If the two hands are injured two 400 point HA should be done
b. Quality	Swiss strength norms for adults
**Category 5 : patient’s perspective**	Not addressed in the development Can be evaluated with other instruments like questionnaires

The discussion for each psychometric properties is developed here below.

### Reliability

*Intra-rater reliability* was excellent. Only test 1(mobility) had an ICC < 0.8. Gable [32], with 30 patients, also found an ICC > 0.90 for the total score of the original 400-point HA carried out twice the same day by three evaluators. Naranjo-Olguin for the Spanish version of the 400-point HA [31] found an intra-rater correlation of 0.99. In our study, the two evaluations were performed on average within a time window of 3 days. We chose this interval because no improvement was expected within this short period. Nevertheless, according to the Bland–Altman method, the results at T1 were a little bit different than at T0, with a deviation from zero. However this difference was negligible (− 2, 5 points) and smaller than the SEM and MDC. A learning bias from the patients’ side between T0 and T1 may explain this difference within a short period of 3 days.

*Inter-rater reliability* was good but less than previous findings of the original version. Gable [24], with 85 patients, found an ICC ranging from 0.90 to 0.97, depending on the specialization of the different evaluators (physiotherapists, occupational therapists). In a second study [32], the same group found an ICC superior to 0.95. Naranjo-Olguin in the Spanish version of the 400-point HA [31] found an inter-rater correlation of 0.98. Generally, test n°4 is the most difficult to quote because of the complexity of the activities which requires both hands. Good experience of the evaluators is necessary for the judgement [32]. In our study, 16 couples of very seasoned occupational therapists (OT) were involved in the inter-rater reliability. This could explain a lesser ICC in our study compared to the previous studies which involved a maximum of 2 or 3 different evaluators.

#### Measurement error

According to Beaton and Schmitt [54, 55], we used the method with the SEM to calculate the MDC. These authors stipulated that a higher MDC than SEM is expected. Both SEM and MDC depend on intra-rater reliability. The higher the intra-rater ICC is, the lower the SEM and MDC will be. In our study, the intra-rater ICC is very high with a value of 0.967. The MDC of the 400-point HA was 2.7 fold higher than the SEM. As a comparison in the validation of the original version of the 400-point HA, in 56 patients we found an SEM of 4.29 and an MDC at 11.9, which are very similar to the current values (unpublished personal data).

For the Q*uick*DASH, as expected, the MDC was also higher than the SEM. Our results are in accordance with previous studies with various pathologies of the upper limb [56, 57], but less than the MDC found in the Smith-Forbes study [58] only for hand pathologies.

#### Internal consistency

Cronbach’s α of the 400-point HA was lower than the ones from the original French(0.97) [25] and the Spanish versions (0.98) [31]. This could be explained by a low Cronbach’s α observed for the second test (strength). In the original version, the strength was measured using five different dynamometers which evaluated quite similar grip capacities. Thus the result was more homogeneous. In the present version, only two dynamometers were used, and they measured two different types of grips (entire hand and thumb–index pinch). From a statistical point of view, the assessment with more items is likely to demonstrate higher internal consistency.

A Cronbach’s α of more than 0.80 is considered as good [37], and a value of 0.88 seems sufficient to avoid redundancy of the items. We are very confident of our results because the original 400-point HA was well developed and validated in 1996 and its unidimensionality was confirmed [25].

### Validity

#### Content validity

For content validity, all the COSMIN guidelines (Box D) [35] were fulfilled. The 400-point HA is a descriptive and evaluative instrument that covers the hand function well, except for sensitivity. The ICF items not covered by the 400-point HA may be evaluated by a specific test (for example sensitivity) or by questionnaires: *Quick*DASH or Brief Pain Inventory, for instance.

#### Construct validity

For the construct validity we explored the correlation between the 400-point HA and the JTHFT. In this study, the correlation can be considered as medium, which was not as good as expected. This result could be explained by the fact that the 400-point HA explores the entire hand function, whereas the JTHFT mainly assess unilateral tasks involving direct manipulation and use of tools with seven tests. Surprisingly, the third and fourth part of the 400-point HA, which are close to the JTHFT, showed a weak correlation. A possible explanation for this, could be the difference in the number of tests in the third and fourth parts of the 400-point HA (40 vs. 7), their complexity and a difference of quotation between the two tests. The JTHFT is only based on time of completion with the dominant and non-dominant hands, whereas the 400-point HA is based on the quality of prehension and displacement of objects by the injured hand and movement quality for the third and fourth tests respectively (the uninjured hand is the comparator.) Time is only informative; it is not used for the calculation of the final score. Our study is the first to make a comparison between the 400-point HA and the JHFT. In the field of locomotor apparatus, no other studies are available for comparison.

The correlation with the Q*uick*DASH was weak at T0 but medium at T3. Similarly, correlation with the MOS-SF 36 PC at T0 was weak. These results were expected and in accordance with our hypotheses because the *Quick*DASH and MOS-SF36 are self-assessment outcomes (subjective) and the 400-point HA is observational and tends therefore to be more objective. As expected, the correlation was slightly better with the specific questionnaire for the upper limb (Q*uick* DASH) than with the MOS-SF36 PC, which is a more generic tool. These results are in accordance with a previous study from our group. In 2011 [59], we found a weak correlation (− 0.388) between the 400-point HA and the Patient Rated Wrist Evaluation (PRWE) questionnaire in 30 patients with various wrist disorders; we also found a weak correlation (− 0.491) between the original version of 400-point HA and the DASH questionnaire in 56 patients with various hand pathologies (unpublished personal data).

As expected, there was no correlation of the 400-point HA with the MOS-SF 36 MC and pain, as these are not evaluated by the 400-point HA.

Construct validity is an evolving property which improves along the way with the successive studies [37]. Therefore the validation process never really ends.

### Responsiveness

The 400-point HA has an acceptable responsiveness and we are able to propose an MCID.

*First using the ROC curve:* for the 400-point HA, the AUC was acceptable, but for the Q*uick*DASH, it was poor. Terwee [52] considered an AUC of at least 0.70 to be adequate. Despite that limit, we calculated an MCID with the optimal cut-off of the ROC curve of 6.07 for the 400-point HA and − 2.27 for the Q*uick*DASH.

Some disparities of AUC between centres were observed in our study and may explain these disappointing results. Three centres had an AUC of 0.76, 0.69 and 0.66, respectively, whereas one centre had a discordant result of 0.27.Fortunately, this centre only included 18 patients at T3. Combined the AUC of the other centres was 0.70. These three centres (Sion, Saint-Martin d’Hères, Nancy) had the same typology of patients (mainly men, aged between 40 and 50 years, manual workers, injuries), with an interval between injury and study of 1 year or more. The fourth centre (Faro) included more women, who were older, with a shorter evolution of 4 months and with more benign pathologies (see additional file 4). We verified all the data from this centre. A plausible explanation for this quite low result may come from the fact that from the 18 patients evaluated at T0 and T3, 13 rated 6 or 7 in the PGIC, and five rated 5. This make it difficult to discriminate improved from unimproved patients in this centre.

As commonly observed in multi centre studies, there were a few differences among the four centres that may explain, in part, these discrepancies. One centre was only ambulatory (Faro). Two centres (Nancy and Saint-Martin d’Hères) combined hospitalised and ambulatory patients; management of the patients started just after the injury, and whether hospitalised or not, management continued in an ambulatory setting for a long period of time. The last clinic (Sion) was a tertiary centre, with patients hospitalised for 4 to 5 weeks. Another problem that was identified in our study comes from the interpretation of the PGIC. Indeed, the patients were asked to answer about how their health status had improved following treatment. We realized that the question was not precise enough. Patients followed for a long time by the same centre (all except Sion) referred to their situation immediately after the injury and at T3. They did not compare their situations between T0 and T3, which was the initial goal of the study. This probably led to underestimation of the MCID (around one point in these centres) and thus a lower AUC. An example to illustrate our interpretation, a few patients could have answered “much improved” to the PGIC at T3 (in comparison to their status immediately after the injury), but only displayed a minor improvement in the 400-point HA between T0 and T3. In contrast, in Sion for instance, most of the patients entered months after the injury and their answers to the PGIC were related specifically to the current hospitalization. The AUC was better (0.765), and the MCID was 10.4.

There was a consistency between the four centres for the Q*uick*DASH, for not being able to discriminate between the improved and unimproved patients. The MCID was very far from the MDC. Perhaps the interval between T0 and T3 was not long enough to reveal a change. Kennedy [60], in a review about the Q*uick*DASH, reported that responsiveness was poor to fair in eight over the nine studies selected, and that most of the studies did not report correlations between changed scores or the calculation of the AUC.

*Second with the mean change score:* This approach compares patients who improved (PGIC = 6 or 7) to the others who did not improve (PGIC from 1 to 5). This method is recommended in association with the ROC curve for the determination of the MCID [39, 61]. With this method, the results were more consistent for the 400-point HA (MCID >MDC) in correlation with PGIC.

For most authors [39, 49, 61, 62], the combination of the two methods (ROC curve and mean change score) is the gold standard for MCID determination. With this method, several values of the MCID are calculated. There is no clear consensus about the ratio between MCID and MDC. A few authors recommend that the MCID must be greater than the MDC [54, 61], whereas others suggest the exact opposite [48, 54]. Factors influencing the MCID are well known: the population studied (age, disease group and severity, treatment), the choice of PGIC, the choice of anchor, the base-values and the direction of change [39, 49]. For our population, the 400-point HA MCID was lower than the MDC when assessed with the ROC curve and superior to the MDC, with mean change score. We thought that for clinical use, values under the MDC should be rejected, so we suggested to keep a value of MCID greater than 12.3 for the 400-point HA in our population. That proposition was reinforced by the calculation of the correlation between the PGIC and the delta 400-point HA as recommended in the literature [51].

These results must be confirmed in other studies and other pathologies.

### Our study has a few limitations

First, our population was mainly composed of middle-aged men and manual workers with injuries. We cannot generalize our results to other populations. Second, the standardised equipment needed was not yet easily available, but it will be commercialised soon. Third, as there is a consensus towards the development of less- time consuming tools, our test could appear relatively long to administer (30 to 40 min). In our opinion this should not be a barrier as the 400-point HA covers four tests in one. The 400-point HA needs a long learning curve, but its advantage is that you can focus the treatment on the target deficit and do the test again (or a part of it) after treatment. Finally, the 400-point HA was developed for an inpatient population and seems to be more used in hospitals than in the private practice of occupational therapists. From our experience, we are convinced that once mastered this tool could also be easily used in private practice.

## Conclusions

The standardised version of the 400-point HA has good psychometric properties for our population of injured workers.

The reliability (intra and inter-rater reliability, measurement error, MDC and internal consistency) is the same as the original version.

For validity, content validity is good and construct validity i*s* acceptable, with four to five hypotheses fulfilled completely and one hypothesis partially fulfilled. It has to be improved in other studies with other PROs or hand evaluation tests.

For responsiveness, the 400-point HA is sensitive to change, and we propose a minimum MCID of 12.3 points (anchor-based approach) for clinical practice. However, the MCID must be studied in other pathologies, particularly in non-injury lesions (tendinitis, carpal tunnel, repetitive injuries, osteoarthritis, and so on) and in other populations which are not represented in our study.

For interpretation of the results, for daily practice, we recommend to look at the total 400-point HA score which is interesting for evaluation of the overall hand function. Then, with the values of the sub-scores, the clinician can focus the therapy upon one or more deficit (mobility, strength, dexterity or bi manual activities).

## Supplementary information

**Additional file 1.**

**Additional file 2.**

**Additional file 3.**

**Additional file 4.**

## Data Availability

The data sets used and/or analysed during the current study are available from the corresponding author upon reasonable request.

## Abbreviations

ICF: International Classification of Functioning
WHO: World Health Organization
PROs: Patients Reported Outcomes
DASH: Disabilities Arm Shoulder and Hand
Q*uick*DASH: Q*uick* Disabilities Arm Shoulder and Hand
FCE: Functional Capacity Evaluation
JTHFT: Jebsen-Taylor Hand Function Test
TEMPA: Test d’Evaluation des Membres supérieurs des Personnes Agées
SODA: Sequential Occupational Dexterity Assessment
400-point HA: 400-point Hand Assessment
ICC: Intraclass Correlation Coefficient
MOS-SF 36: Medical Outcome Study-Short Form 36
MOS-SF 36 PC: Medical Outcomes Study-Short Form 36 Physical Component
MOS-SF 36 MC: Medical Outcomes Study- Short Form 36 Mental Component
NRS: Numerical Rating Scale
PGIC: Patient Global Impression of Change
MCID: Minimal Clinically Important Difference
ROC: Receiver Operating Characteristic
AUC: Area Under Curve
SEM: Standard Error of Measurement
SD: Standard Deviation
MDC: Minimum Detectable Change
ES: Effect Size
SRM: Standardized Response Mean
CCVEM: Commission Cantonale Valaisanne d’Ethique Médicale
CRPS: Complex Regional Pain Syndrome
PRWE: Patient Rated Wrist Evaluation
MHQ: Michigan Hand Questionnaire
ADL: Activities of Daily Living
FDT: Functional Dexterity Test
OT: occupational therapist
