# Supplementary material for: Prospective multicentre validation study of a new standardised version of the 400-point hand assessment
Source: BMC Musculoskelet Disord. 2020 May 20;21:313. doi: 10.1186/s12891-020-03303-4 (PMC7240941; doi:10.1186/s12891-020-03303-4)

BILAN 400 POINTS

(V 2)

Mme Colette GABLE, ergothérapeute
Professeur Jean PAYSANT

Institut Régional de Médecine Physique et de Réadaptation
75, Boulevard Lobau 54042 NANCY

INTRODUCTION

L’évaluation de la capacité de préhension dans sa complexité reste un véritable défi. L’emploi classique des bilans orientés vers la mesure d’un aspect particulier de la fonction de préhension (articulaire, musculaire, trophique, sensitif) n’apporte qu’une réponse fragmentée et incomplète de la capacité de préhension.

« Le bilan 400 POINTS » propose une évaluation à quatre niveaux : motricité – force – prise mono manuelle – coordination bi manuelle. L’observation dans une soixantaine de gestes de la vie courante renseigne sur les qualités de préhension et les ressources d’adaptation. La note attribuée à chaque épreuve « pointe » le secteur déficitaire.

Cette évaluation nécessite un temps de 30 à 45 minutes pour sa passation.

DESCRIPTION

Ce bilan est composé de 4 épreuves, chacune notée sur 100 points :

1 – MOBILITE DE LA MAIN ....

2 – FORCE DE PREHENSION ....

3 – PRISE ET DEPLACEMENT D'OBJETS EN MONOMANUEL...

4 – FONCTION BIMANUELLE ....

Chacune de ces épreuves comporte :

- ✓ son matériel,
- ✓ son mode d'emploi,
- ✓ sa cotation,
- ✓ son mode de calcul.

La fiabilité de ce bilan nécessite des conditions de réalisation constante, l'utilisation d'un même matériel et d'une installation identique :

- Une table réglable en hauteur (environ 60 cm de large et 90 cm de long).
- Un siège stable (tabouret avec patins), réglable en hauteur pour le patient.
- Un tabouret à roulettes, réglable en hauteur pour l'ergothérapeute.
- Un meuble rassemblant le matériel nécessaire à portée de main.
- Une table réglable en hauteur sur laquelle sont placés deux plans de référence espacés de 50 cm : la table est réglée de manière à ce que le plan supérieur soit à la hauteur des épaules du patient testé.

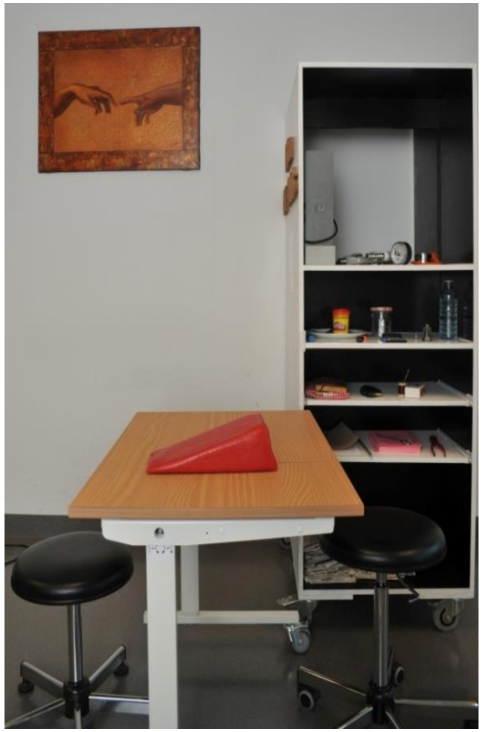	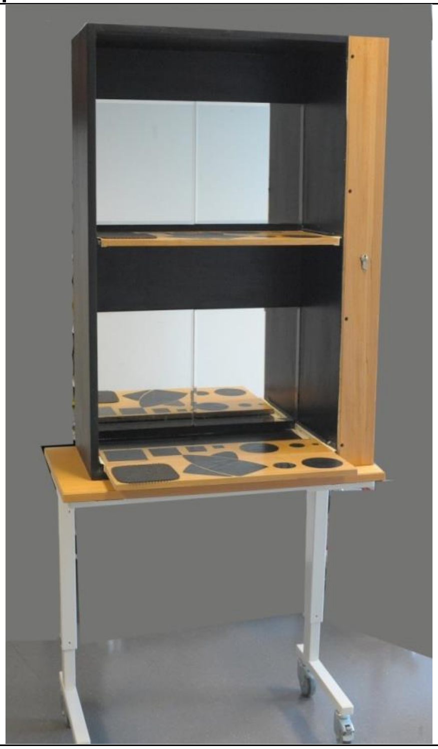	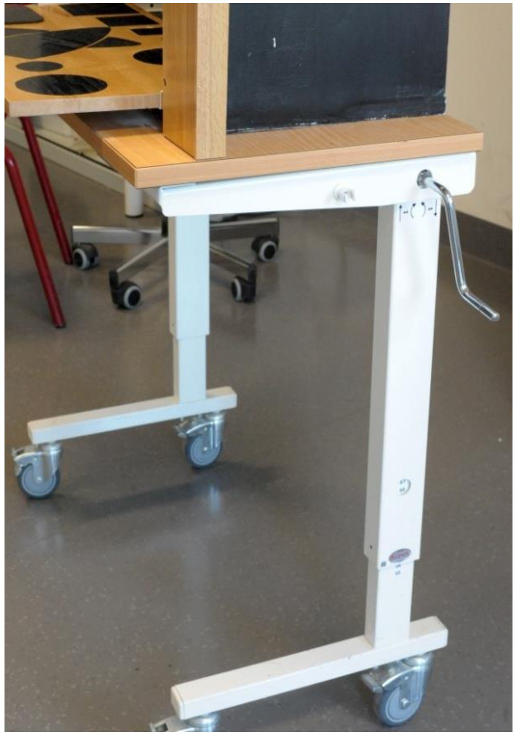

EPREUVE 1 : MOBILITE DE LA MAIN

Il s'agit de tester simultanément les deux mains du patient au moyen de quatorze mouvements globaux dont on note la qualité d'exécution :

1. Fermeture de la main
2. Ouverture de la main
3. Écartement des doigts longs
4. Rapprochement des doigts longs
5. Écartement du pouce
6. Adduction du pouce
7. Opposition du pouce avec les 4 doigts longs
8. Opposition du pouce avec les 4 doigts longs contre Résistance
9. Pinces latérales contre Résistance au moyen d'une carte
10. Prises globales de 3 bâtonnets contre Résistance
11. Flexion du poignet
12. Extension du poignet
13. Pronation
14. Supination

Matériel

- Un coussin triangulaire est utilisé pour les 3 items contre résistance.
- Une carte de type carte à jouer ou carte bancaire
- Trois cylindres de 22 cm de long :
  - un de 3 mm de diamètre
  - un de 10 mm de diamètre
  - un de 20 mm de diamètre

Mode d'emploi

Le patient est assis confortablement devant une table, face à l'ergothérapeute.

POSITION DE DEPART
**Les avant-bras en appui sur le bord de la table et les mains soulevées du plan de la table.**

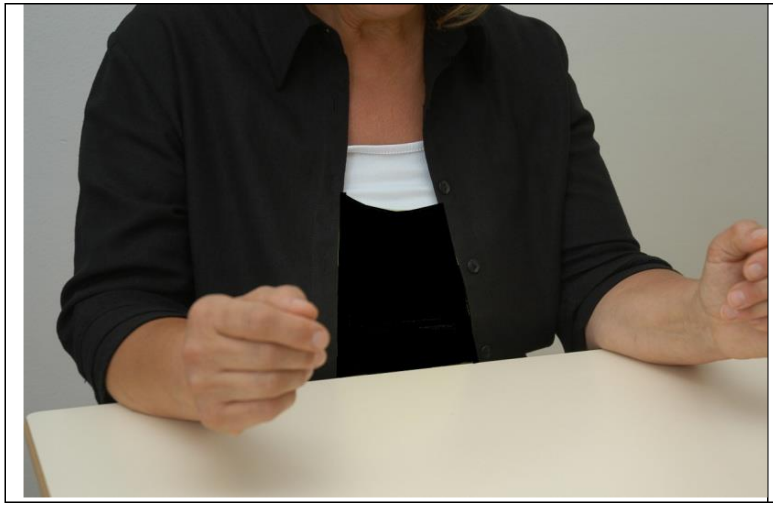

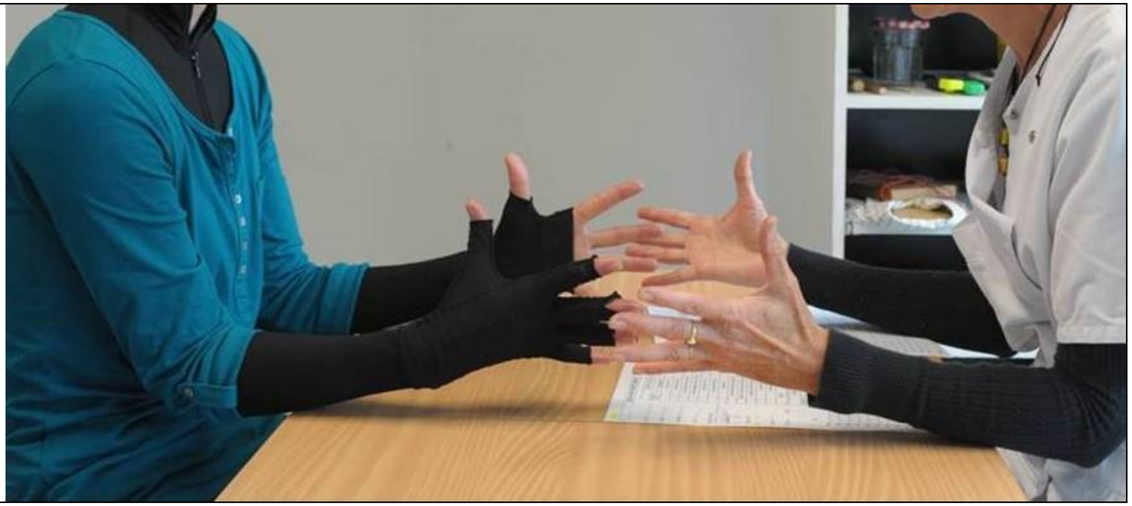

L’ergothérapeute explique et montre les mouvements, le patient doit les reproduire. Les deux mains sont testées en même temps. Pour certains items, il convient de faire répéter les mouvements plusieurs fois de suite, voire même de tester deux mouvements à la fois pour qu’ils soient plus spontanés.

1 et 2 « Fermez vos 2 mains comme si vous vouliez donner un coup de poing puis ouvrez les, fermez, ouvrez, fermez, ouvrez… »

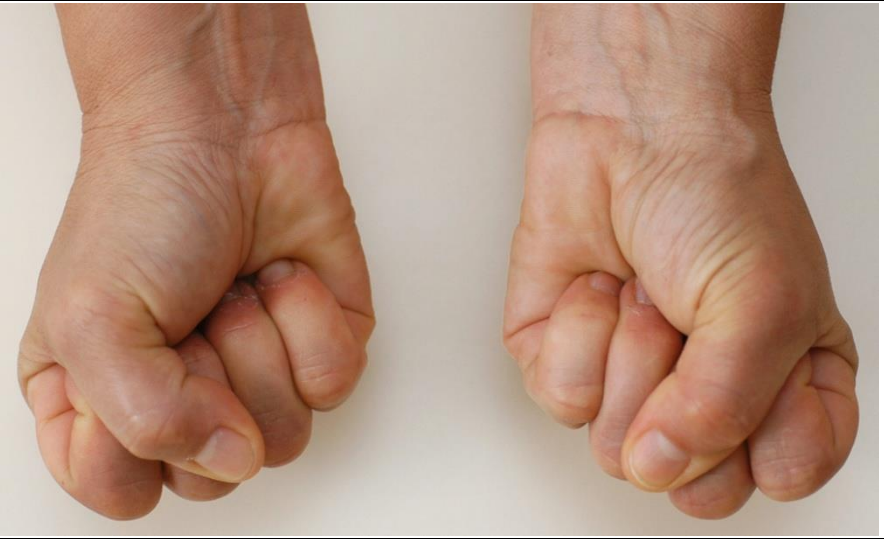	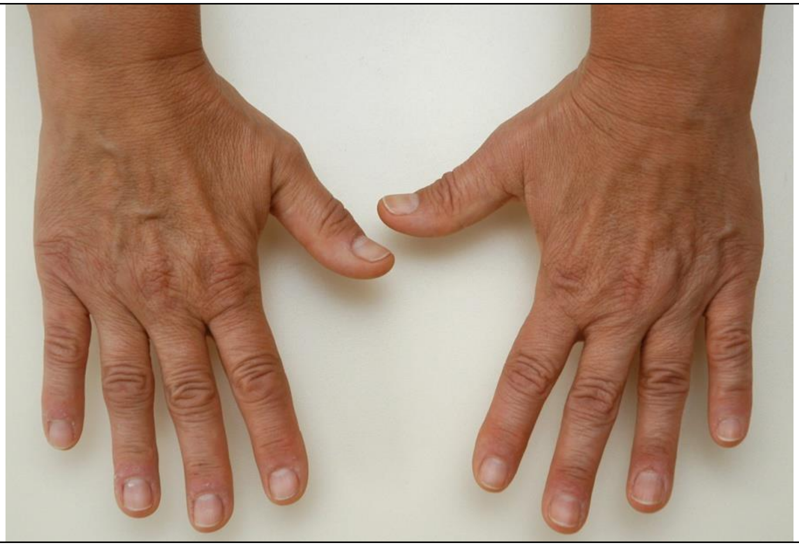

3 et 4 « Écartez les doigts longs de vos 2 mains, rapprochez les, écartez, rapprochez, écartez, rapprochez… »

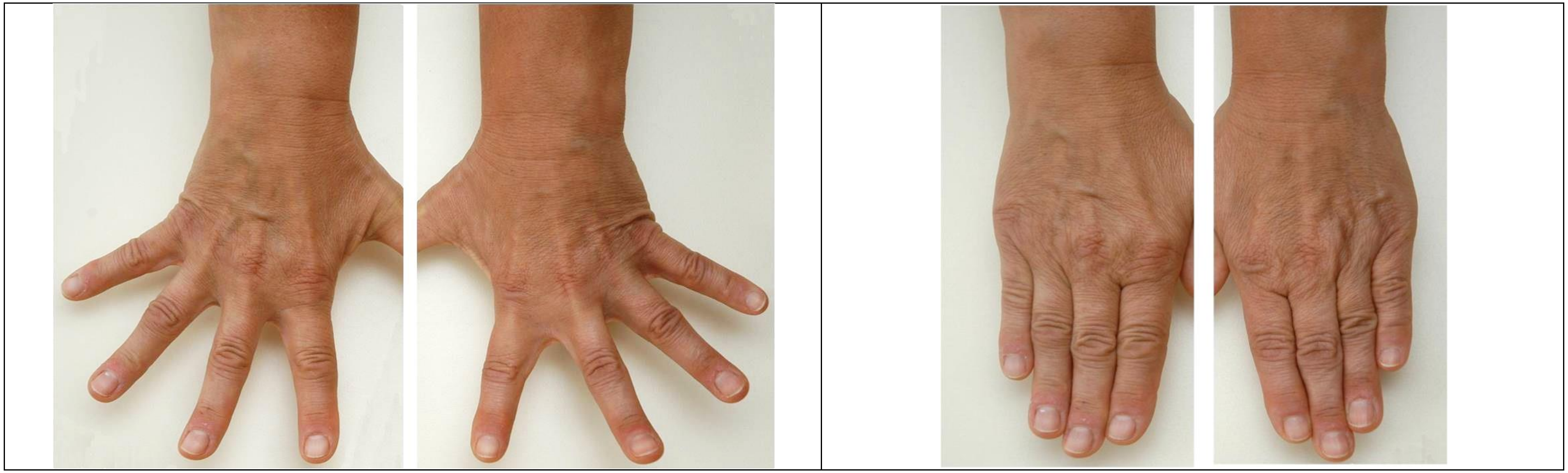

5 et 6 « Ecartez les pouces de vos mains, étendus, dans le même plan que les autres doigts, ramenez les, toujours étendus en les glissant sur l'index jusqu'à la 2eme commissure, écartez, rapprochez… »

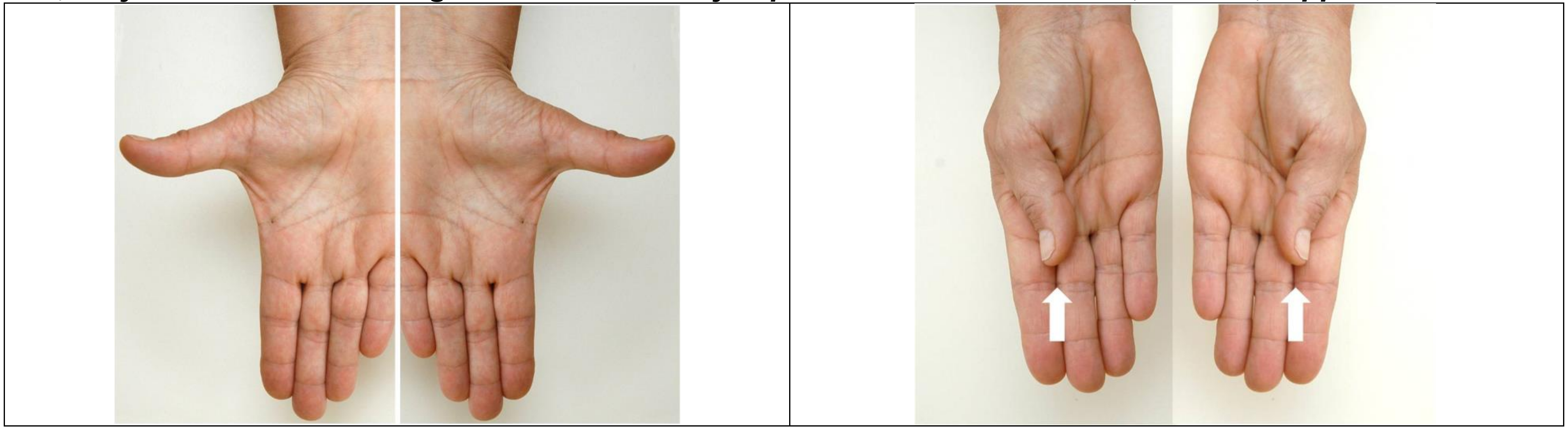

7 « Des deux côtés, venez toucher le bout de chaque doigt long avec votre pouce en faisant de beaux ronds, recommencez… »

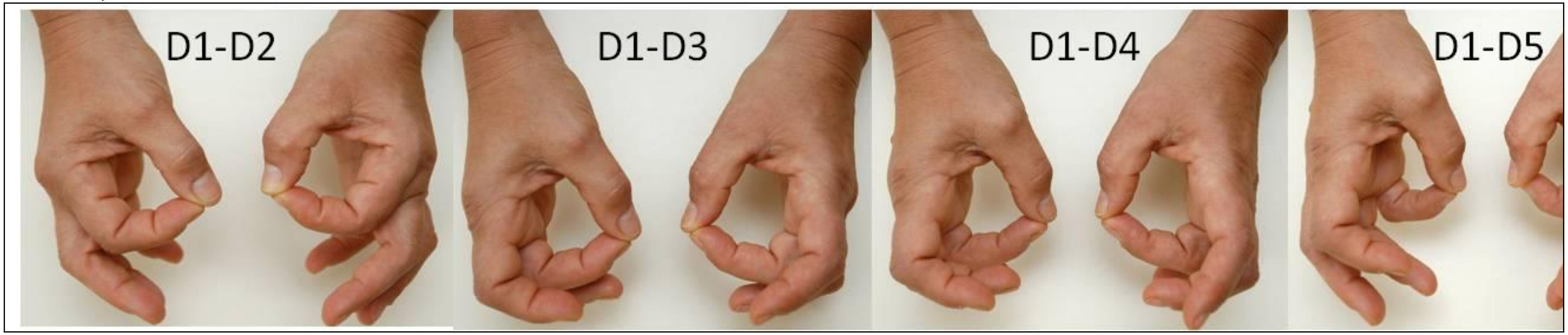

Attention !!!

Même si une seule pince « pouce-doigt long » est impossible, la note globale de «1 » sera donnée pour cet item.

Pour les 3 items contre résistance, chaque main est testée séparément, l'avant-bras du patient repose sur un coussin triangulaire, la main en dehors du coussin.

L'ergothérapeute commence par évaluer la main saine du patient puis il continue avec la main lésée effectuant les Résistances nécessaires.

8 « On recommence avec les mêmes beaux ronds mais cette fois, résistez à ma pression, j'essaie de passer entre vos doigts (d'abord du côté sain puis du côté lésé)… »

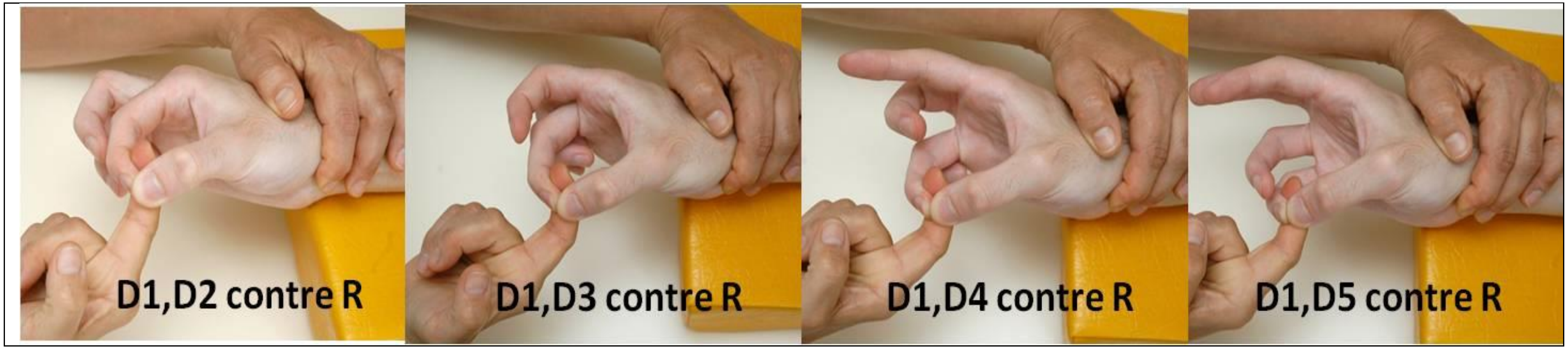

9 « Maintenant, essayez de retenir cette carte entre vos doigts, je vais tirer dessus pour la récupérer (d'abord du côté sain puis du côté lésé)… »

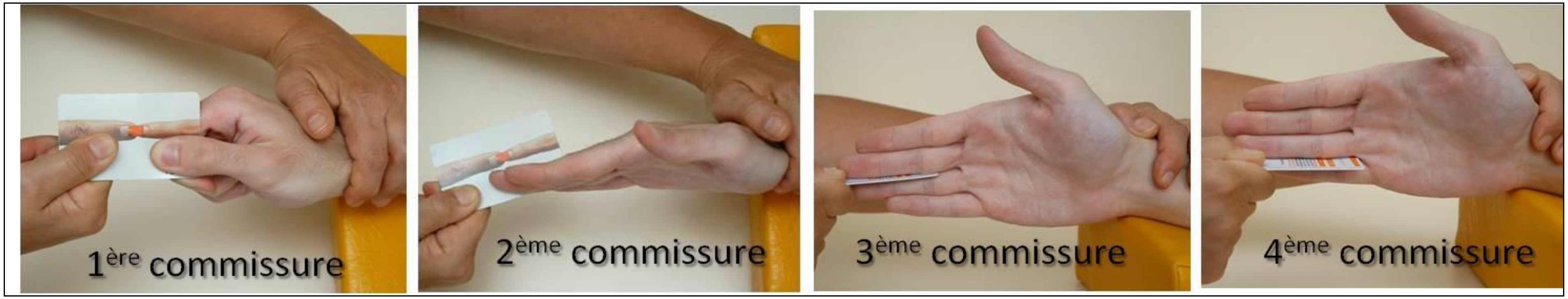

10 « Essayez de retenir ce petit cylindre en fermant bien tous vos doigts et en le maintenant au creux de votre main (au niveau du pli de flexion des MP des doigts longs et placé par le thérapeute), je vais tirer dessus pour le récupérer… (D'abord du côté sain puis du côté lésé du plus petit vers le plus grand diamètre)… »

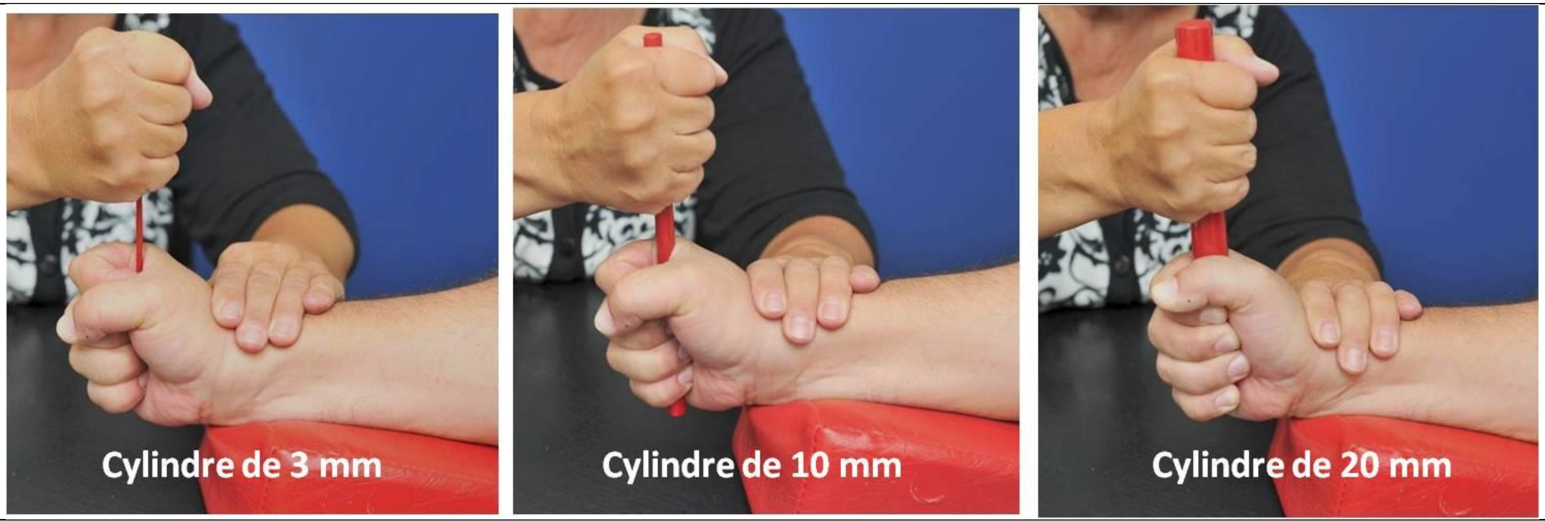

11 « Joignez vos mains en croisant les doigts (dans le sens de la flexion) puis levez vos coudes le plus possible vers une ligne horizontale au niveau de la poitrine… »

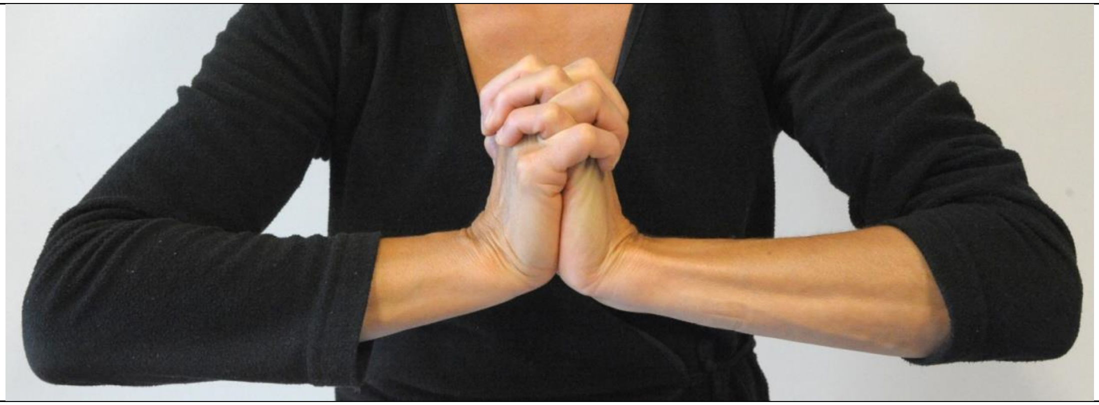

12 « Placez vos mains dos à dos (les doigts dans le sens de l'extension) puis amenez vos coudes vers une ligne horizontale au niveau de la poitrine… »

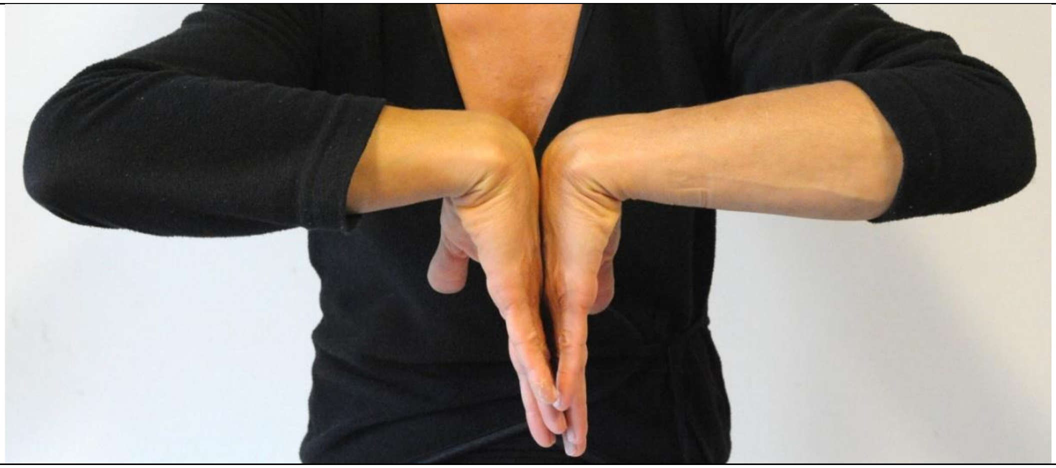

Pour les items 13 et 14, le patient se décale de la table, les coudes collés au corps, fléchis à 90°, les pouces placés au zénith.

13 et 14 « Décalez-vous de la table, placez vos coudes collés au corps en les pliant à 90° avec vos pouces orientés vers le ciel, et tournez vos mains vers le plafond, puis vers le sol, vers le plafond puis vers le sol… »

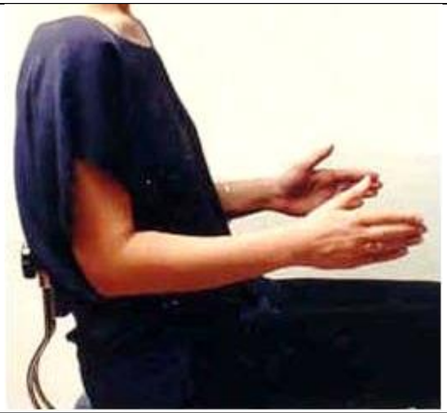

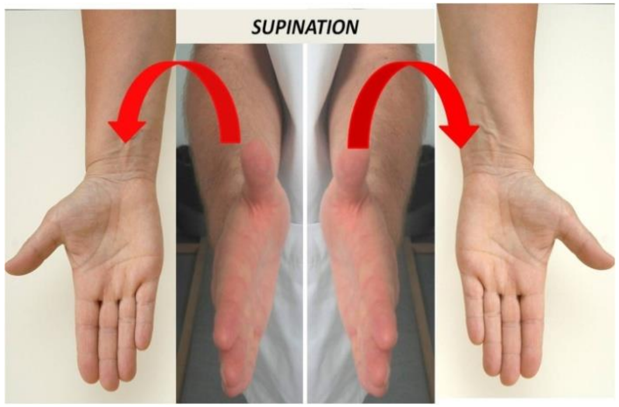

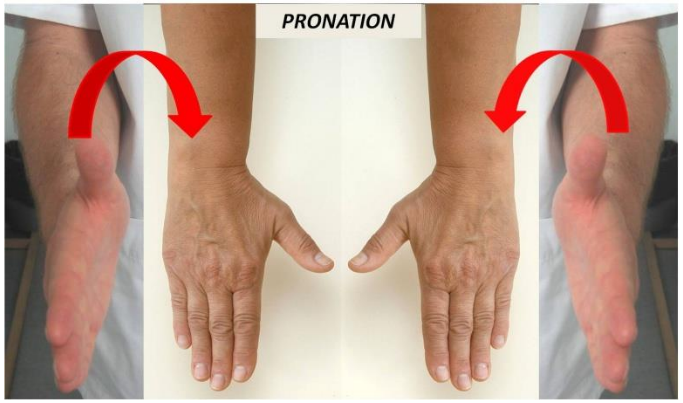

Cotation

COTATION pour les ITEMS 1 à 7 et 11 à 14	MOUVEMENT COMPLET*, HARMONIEUX ET PRECIS	MOUVEMENT COMPLET MAIS LENT, DISHARMONIEUX OU IMPRECIS	MOUVEMENT INCOMPLET OU AVEC EXCLUSION OU AVEC COMPENSATION	MOUVEMENT IMPOSSIBLE**
	3	2	1	0

Un « mouvement complet » est un mouvement effectué dans toutes ses amplitudes et avec toutes ses qualités :
Harmonie du geste, bonne vitesse et précision grâce à l'intégrité physique de la main, du poignet et de la commande volontaire.

*****Un mouvement peut être « impossible » à cause d'une contre-indication médicale ou d'une incapacité provisoire ou définitive.***

Cotation spécifique pour les mouvements contre Résistance :

Pour les items 8 et 9

COTATION POUR LES ITEMS 8 ET 9	PRISE MAINTENUE CONTRE UNE RESISTANCE IMPORTANTE DU THERAPEUTE	PRISE MAINTENUE CONTRE UNE RESISTANCE MOYENNE DU THERAPEUTE	PRISE MAINTENUE CONTRE UNE LEGERE RESISTANCE DU THERAPEUTE	PRISE NON MAINTENUE
	3	2	1	0

Pour l'item 10

COTATION POUR L'ITEM 10	BATONNET DE 3 mm MAINTENU CONTRE LA RESISTANCE DU THERAPEUTE	BATONNET DE 10 mm MAINTENU CONTRE LA RESISTANCE DU THERAPEUTE	BATONNET DE 20 mm MAINTENU CONTRE LA RESISTANCE DU THERAPEUTE	BATONNET DE 20 mm NON MAINTENU CONTRE LA RESISTANCE DU THERAPEUTE
	3	2	1	0

Coefficients

Tous ces items n'ont pas tous la même importance dans la fonction de la main, aussi chacun d'entre eux possède un coefficient modérateur de manière à les hiérarchiser.
En effet, il est plus important d'avoir une fermeture complète de la main qu'une extension parfaite des doigts, aussi le coefficient de la fermeture est de 3 et celui de l'ouverture est de 2.

Tableau des coefficients modérateurs

**FERMETURE DE LA MAIN**	**3**
**OUVERTURE DE LA MAIN**	**2**
**ECARTEMENT DES DOIGTS LONGS**	**3**
**RAPPROCHEMENT DES DOIGTS LONGS**	**2**
**ABDUCTION DU POUCE**	**3**
**ADDUCTION DU POUCE**	**2**
**OPPOSITION DU POUCE AUX 4 DOIGTS LONGS**	**3**
**OPPOSITION DU POUCE AUX 4 DOIGTS LONGS CONTRE RESISTANCE**	**2**
**PINCES LATERALES D'UNE CARTE CONTRE RESISTANCE**	**2**
**PRISES GLOBALES DE 3 BATONNETS CONTRE RESISTANCE**	**3**
**FLEXION DU POIGNET**	**2**
**EXTENSION DU POIGNET**	**3**
**PRONATION**	**3**
**SUPINATION**	**2**

Mode de calcul

- Chaque item est coté de **0 à 3**.
- Chaque cotation est **multipliée par son coefficient** pour obtenir une note.
- Le total de l'épreuve est obtenu en additionnant les 14 notes, il **correspond à la somme des notes du côté lésé**.
- Enfin, cette note est **divisée par 105** (score maximum) puis **multipliée par 100** pour aboutir au **pourcentage de mobilité de la main lésée par rapport à la mobilité d'une main saine.**

Exemple : Score d'une main saine = score maximum = 105 points
Score de la main lésée = 69

On fera :

$$\frac{69}{105} \times 100 = 65{,}7$$

Attention !!!
Lors du calcul du résultat final de l'épreuve, si le chiffre n'est pas rond, il convient :
d'arrondir au chiffre inférieur quand la décimale est inférieure ou égale à 5 et d'arrondir au chiffre supérieur quand la décimale est supérieure à 5. **Ainsi 65,7 devient 66 /10**

Interprétation : Le patient a une mobilité de 66% au niveau de sa main lésée (par rapport à une main saine)

EPREUVE 2 : FORCE DE PREHENSION

La mesure de la force de préhension se fait au moyen de deux instruments de mesure. Elle est faite successivement du côté sain puis du côté lésé. Pour se familiariser, chacun des appareils peut être essayé une ou deux fois par le patient, du côté sain, avant de démarrer l'épreuve.

Il s'agit donc de tester :
1• LA FORCE DE POIGNE à l'aide du dynamomètre de Jamar (réglé au 2ème cran), référence internationale, testant les muscles extrinsèques et les intrinsèques.
2. LA FORCE DE SERRAGE de la pince latérale entre la pulpe du pouce et le bord radial de l'index à l'aide d'un pinchmètre Jamar, testant intrinsèques et extrinsèques du pouce.

Matériel

- un dynamomètre Jamar
- un dynamomètre pinchmètre Jamar

Mode d'emploi

Position de départ : le patient est installé conformément aux recommandations de la Société Américaine de Rééducation de la Main :

 - assis avec un dos droit
 - les pieds à plat au sol

- les épaules en adduction neutre
- les bras sans support
- les coudes fléchis à 90°
- les avant bras en position neutre
- le poignet de 0 à 30° d'extension avec une déviation ulnaire de 0 à 15°.

Les dynamomètres sont maintenus par l’ergothérapeute. Pour chaque dynamomètre, le score est obtenu en faisant la moyenne de 3 mesures successives Celles ci sont faites alternativement du côté sain puis côté lésé puis côté sain, côté lésé.....

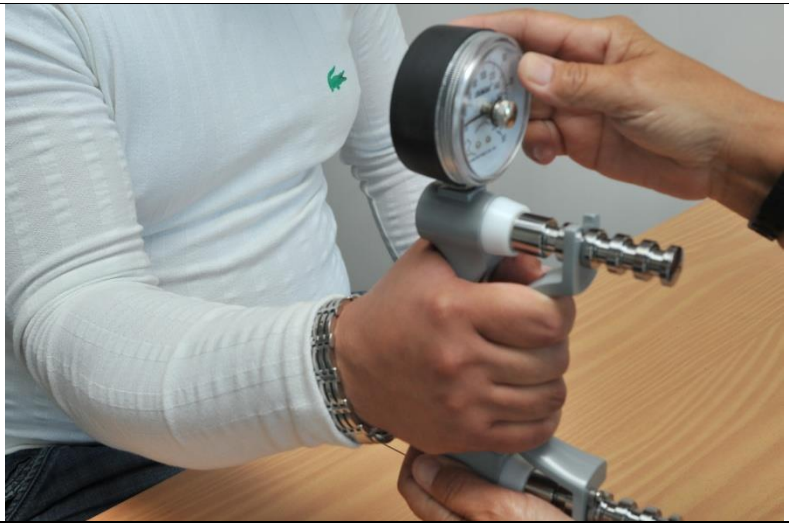

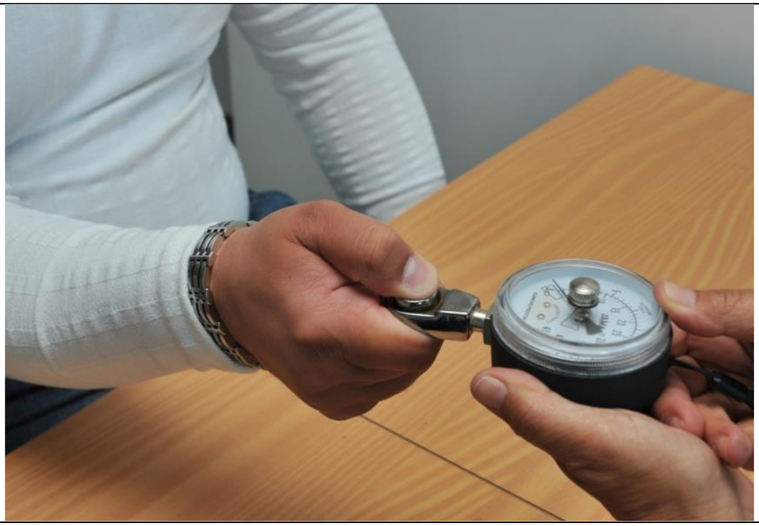

Mode de calcul

Par convention, la main saine a une force maximale. La valeur obtenue par celle-ci, pour chacun des dynamomètres, correspond donc à la note maximale c'est à dire 10/10.
Pour chaque dynamomètre, la main lésée est notée par rapport à la main saine, sa note est obtenue en faisant une règle de trois.

°Par exemple, pour le dynamomètre Jamar :

Côté sain = 48 kg ; la note du côté sain correspond à 10/10.
Côté lésé = 18 kg ; la note du côté lésé correspond à :

$$\frac{\text{Valeur côté lésé}}{\text{Valeur côté sain}} \times 10 = \text{ soit } \frac{18}{48} \times 10 = \mathbf{3{,}7\ /\ 10}$$

° Pour le dynamomètre Pinch, les calculs sont les mêmes :

Côté sain = 9,5 kg ; la note du côté sain correspond à 10/10.
Côté lésé = 3,5 kg ; la note du côté lésé correspond à :

$$\frac{\text{Valeur côté lésé}}{\text{Valeur côté sain}} \times 10 = \text{ soit } \frac{3{,}5}{9{,}5} \times 10 = \mathbf{1{,}2\ /\ 10}$$

Coefficients

Chacun des 2 dynamomètres à un coefficient de 5. Ainsi dans notre exemple, le côté lésé du patient obtient :
° pour le dynamomètre Jamar : 3,7 x 5 soit 18,5 / 50.

° pour le dynamomètre pinch : 1,2 x 5 soit 6 / 50.
La somme pour le côté lésé est faite : 18, 5 + 6 = 24,5

Le total sera de 100 du côté sain et de 24,5 / 100.

Pondération

Dans la littérature, les études de mesure de la force de préhension accordent en règle générale 8 à 12% de force de préhension en plus au niveau de la main dominante.

Ainsi, il convient de réajuster le résultat :

°si la main dominante est la main lésée, 10% sont retirés au score obtenu
°si la main d'appoint est la main lésée, 10% sont ajoutés au score obtenu

Ainsi, dans notre exemple :

°Total des notes côté sain et d'appoint : 100 /100

°Total des notes côté lésé et dominant : 24,5 /100

La main lésée étant la main dominante, il convient d'ôter 10% au résultat soit 2,4 à 24,5.

On obtient : 24,5 – 2,4 = 22,1 soit **22/100**

- ***Interprétation : On peut dire que ce patient a une force de préhension de 22% par rapport à sa main saine.***

- ***Si les deux mains sont atteintes, le score obtenu est comparé aux moyennes établies à partir d'une étude suisse effectuée sur une population saine, (496 hommes et 482 femmes de 18 à 85 ans et plus) en 2009 :***

FORCE AU JAMAR EN KG					
JAMAR HOMMES (496)			JAMAR FEMMES (482)		
Age	Main Dominante	Main d'appoint	Age	Main Dominante	Main d'appoint
18 à 19 (33)	51,2	48,3	18 à 19 (31)	32	30,7
20 à 29 (59)	53,4	50,8	20 à 29 (61)	33,8	32,5
30 à 39 (69)	55,4	53	30 à 39 (72)	34,8	33,6
40 à 49 (68)	53	56,7	40 à 49 (79)	34	34,1
50 à 59 (70)	52,2	55	50 à 59 (62)	32,8	32,6
60 à 69 (79)	45,4	44,9	60 à 69 (64)	29,1	28
70 à 79 (61)	39,2	38,7	70 à 79 (53)	25,7	24,8
80 à 85 (29)	30,7	29,4	80 à 85 (32)	19,2	19,7
> 85 (28)	22,4	23,2	> 85 (28)	16,9	16,7

FORCE AU PINCH EN KG					
PINCH HOMMES (496)			PINCH FEMMES (482)		
Age	Main Dominante	Main d'appoint	Age	Main Dominante	Main d'appoint
18 à 19 (33)	9,5	9,1	18 à 19 (31)	6,9	6,5
20 à 29 (59)	9,9	9,3	20 à 29 (61)	6,6	6,4
30 à 39 (69)	10,1	9,7	30 à 39 (72)	7	6,7
40 à 49 (68)	10	9,6	40 à 49 (79)	7,1	6,8
50 à 59 (70)	10	9,6	50 à 59 (62)	6,8	6,6
60 à 69 (79)	9,2	8,8	60 à 69 (64)	6,5	6,2
70 à 79 (61)	8,2	7,8	70 à 79 (53)	5,4	5
80 à 85 (29)	6,4	6,5	80 à 85 (32)	4,3	3,9
> 85 (28)	5,4	5,5	> 85 (28)	3,1	2,8

EPREUVE 3 : PRISE MONOMANUELLE ET DEPLACEMENT D'OBJETS

Il s'agit de tester la capacité du patient à :

- prendre 20 objets de taille, poids et forme différents posés sur un plan de référence dans un ordre précis
- et à les transporter sur un plan situé 50 cm plus haut dans les emplacements correspondants.
- L'épreuve débute avec la main saine, puis se poursuit avec la main lésée. Elle est chronométrée, ainsi le patient concentre davantage son attention sur la rapidité d'exécution plutôt que sur la manière dont il le fait.

Le patient est ainsi plus naturel et spontané dans ses gestes, l'ergothérapeute peut alors facilement observer les défauts de préhension comme l'exclusion, les défauts de commande, les compensations..…

Matériel

1-Un cube de 10 cm de côté (700gr)
2-Un cube de 7,5 cm de côté (300 gr)
3-Un cube de 5 cm de côté (100 gr)
4-Un cube de 2,5 cm de côté (10 gr)
5-Un cylindre de 10 cm de diamètre et 12 cm de haut (700 gr)
6-Un cylindre de 7,5 cm de diamètre et 11 cm de haut (300 gr)
7-Un cylindre de 5 cm de diamètre et 10 cm de haut (100gr)
8- Une bille de 25 mm de diamètre
9- Une bille de 16 mm de diamètre
10-Une balle de tennis

11-Une pointe de 4 cm de long et 2 mm de diamètre
12-Un briquet électronique
13- Une pièce de 15 mm de diamètre
14-Une pièce de 25 mm de diamètre
15-Une pièce de 30 mm de diamètre
16-Une clef plate
17-La serrure correspondante
18-Un fer à repasser de 2 kg
19-Une cruche munie de 2 becs verseurs avec 500 ml d'eau
20-Un verre ordinaire

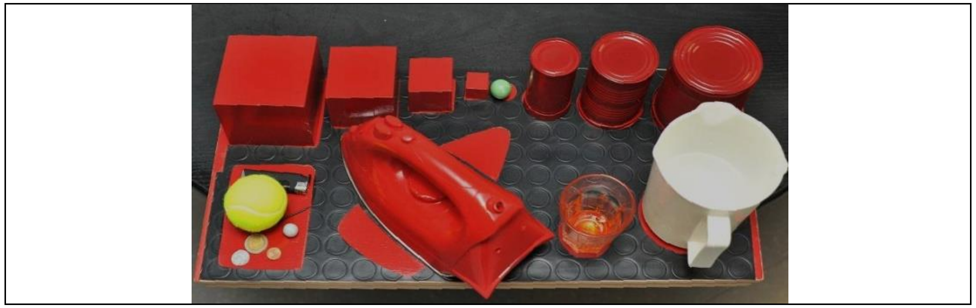

-1 plan de référence (30 cm sur 60 cm) solidarisé et espacé d'un autre plan (de 30 cm sur 6O cm) 50 cm plus haut.
Ceux-ci sont posés sur une table réglable en hauteur ou contre un mur avec un système réglable en hauteur.
La table est réglée de manière à ce que le plan supérieur soit à la hauteur des épaules du patient testé.

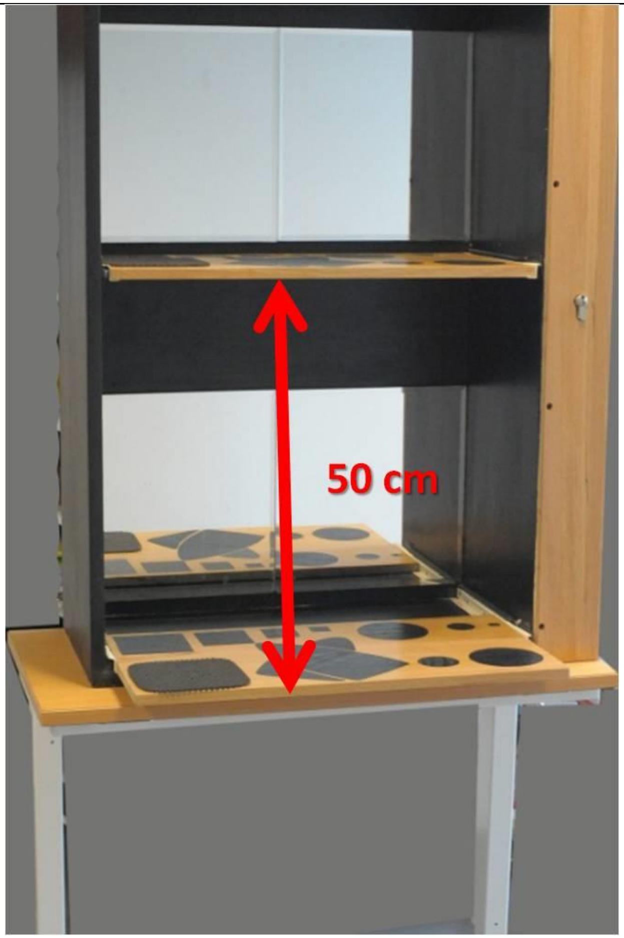
50 cm

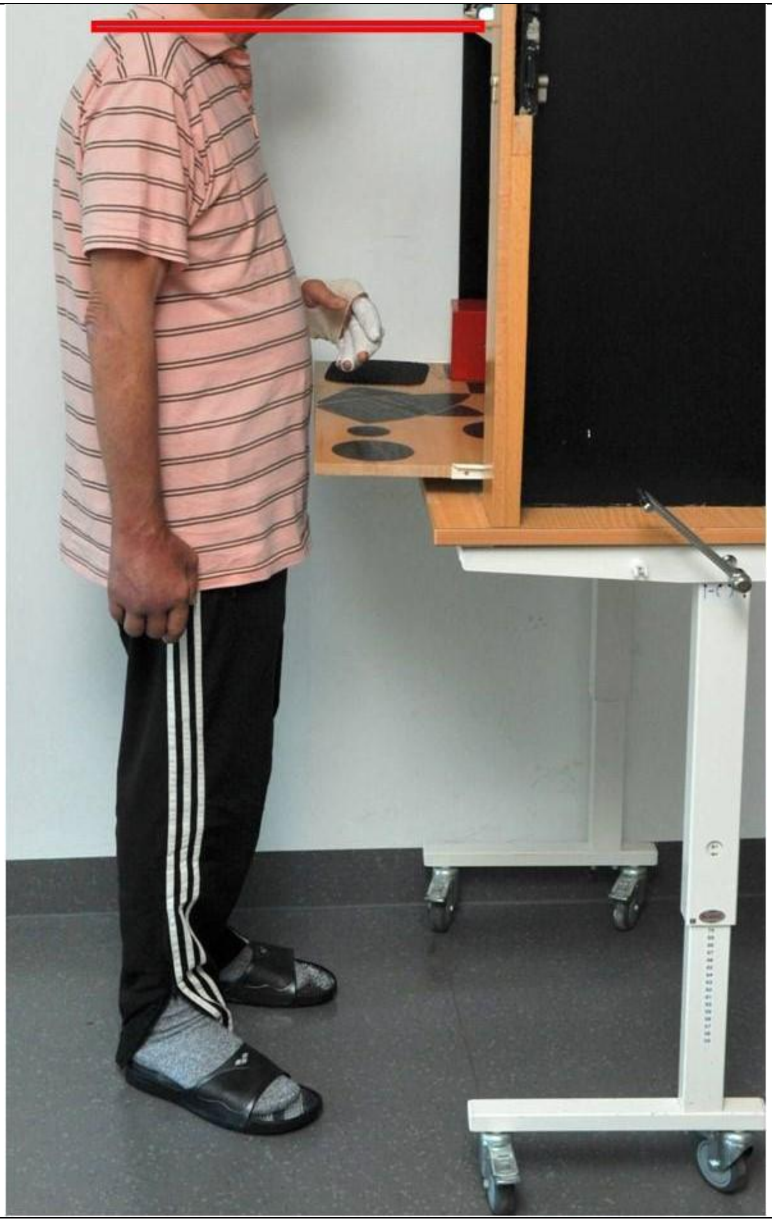

. Un miroir collé au fond de chaque étage permet une observation plus facile de la main.

Mode d'emploi

Attention !!

3 objets nécessitent une action avant de les transporter sur le plan en hauteur :

- le briquet électronique qu'il faut allumer par une pression du pouce
- la clef qu'il faut engager dans la serrure, puis verrouiller et déverrouiller avant de la retirer et de la poser sur le plan supérieur
- l'eau de la cruche (500 ml) dont il faut verser une partie de l'eau en pronation pour la première moitié du verre puis en supination pour l'autre moitié. L'eau du verre est ensuite reversée dans la cruche avant le transport du verre et de la cruche sur le plan en hauteur.

Avant de démarrer l'épreuve, l'ergothérapeute prend soin de montrer au patient la manière de faire tout en lui expliquant qu'il est chronométré et doit donc faire le plus vite possible :

« Prenez les objets, un par un, en commençant par la gauche et en continuant vers la droite, d'abord la rangée du fond puis faites la même chose de la gauche vers la droite avec les objets de la rangée en avant »

« Déposez les objets dans les espaces correspondants, n'oubliez pas d'allumer le briquet, d'actionner la clef dans la serrure et de verser l'eau de la cruche dans le verre comme ceci (en pronation) et comme cela (en supination) »
« Faites le plus vite possible, car vous êtes chronométré. »
« Vous êtes prêts ? Allez-y !!…

Ci dessous, un exemple de chaque prise avec une vue radiale et une vue ulnaire de chacun des objets ainsi que de l'action à faire pour certains d'entre eux. Les vues ont été prises avec le respect de l'ordre des prises, et le bon réglage en ce qui concerne les 2 plans de référence.

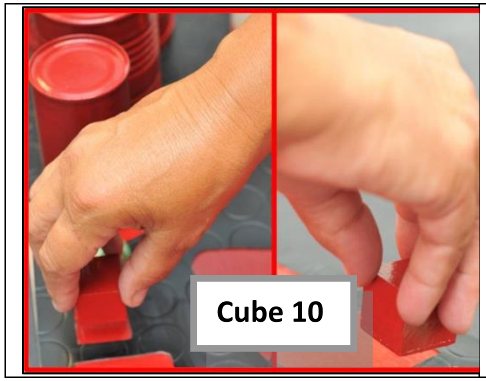

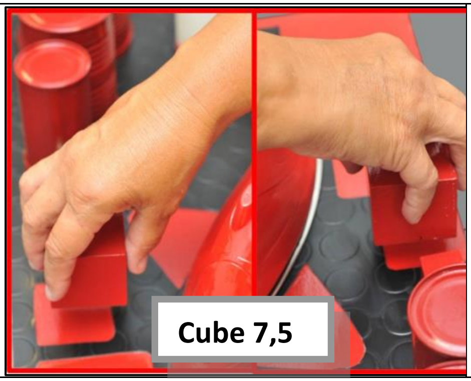

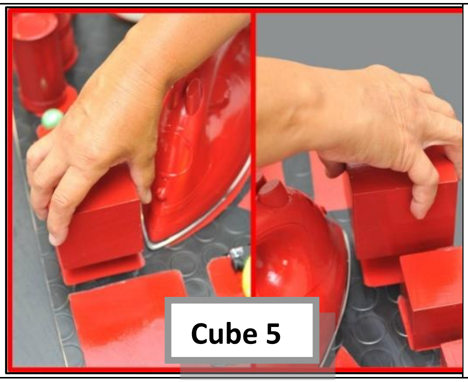

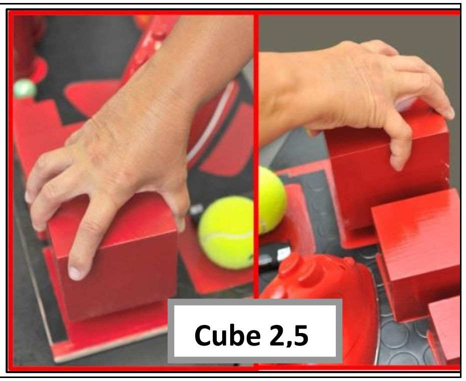

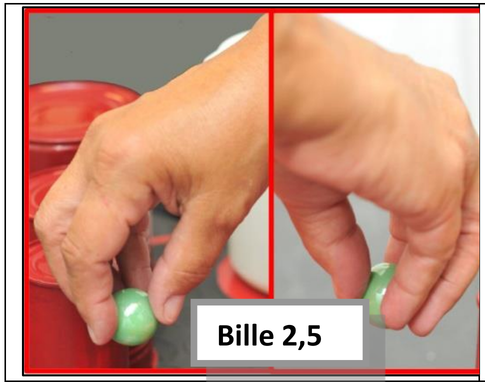
Bille 2,5

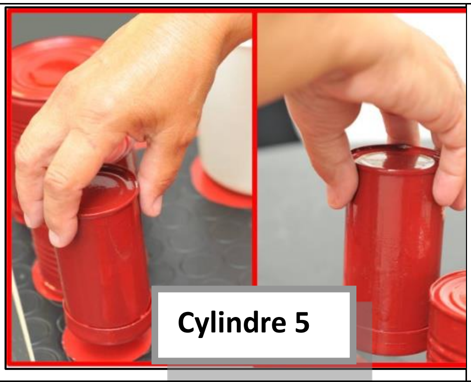
Cylindre 5

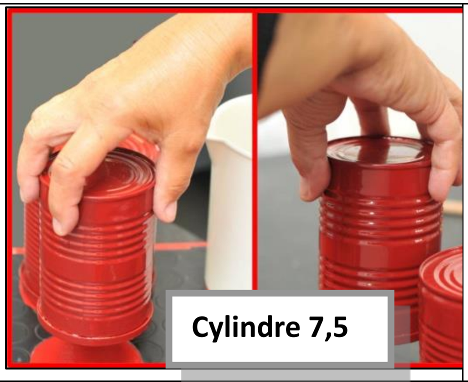
Cylindre 7,5

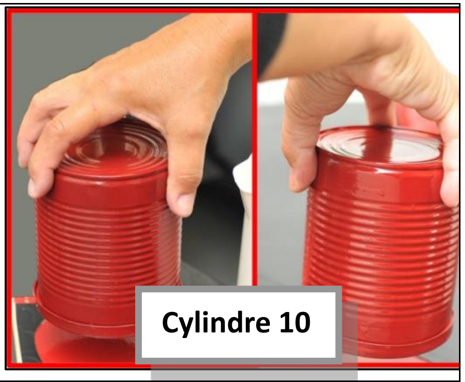
Cylindre 10

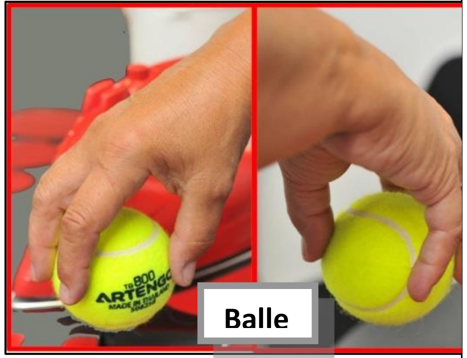
Balle

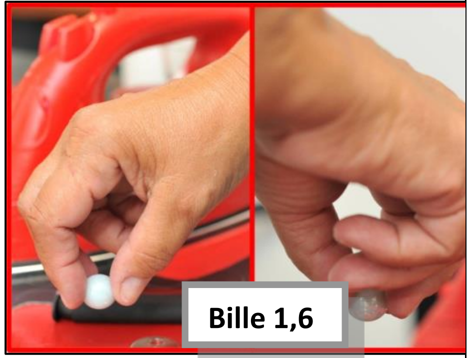
Bille 1,6

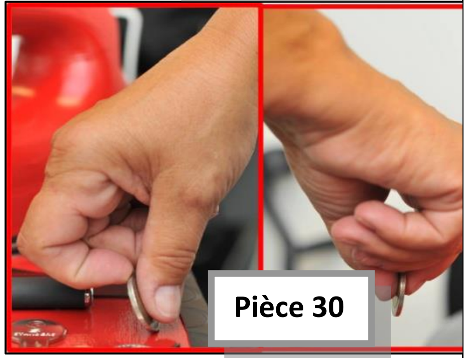
Pièce 30

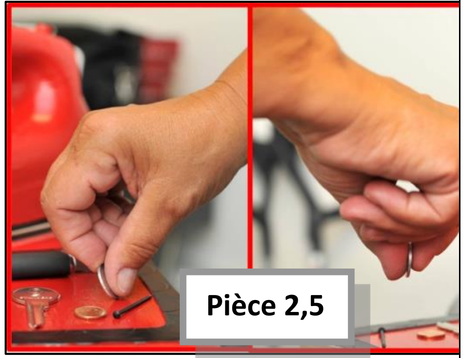
Pièce 2,5

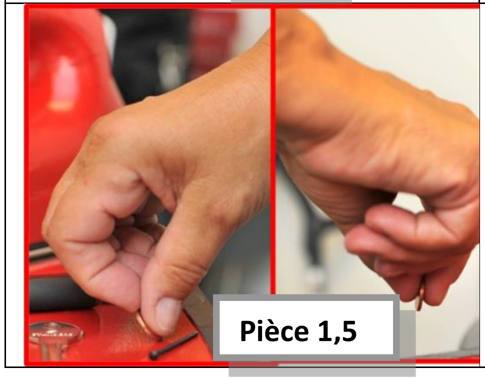
Pièce 1,5

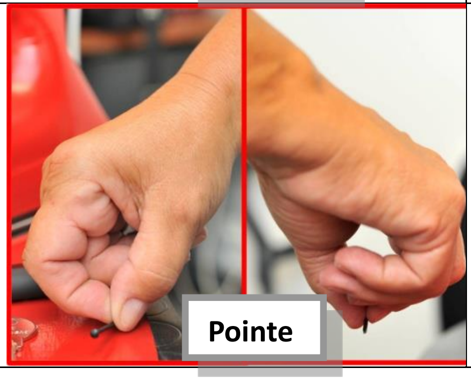
Pointe

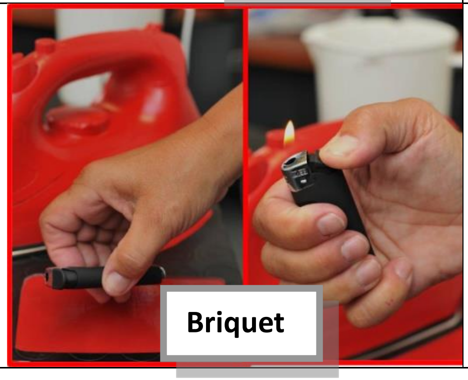
Briquet

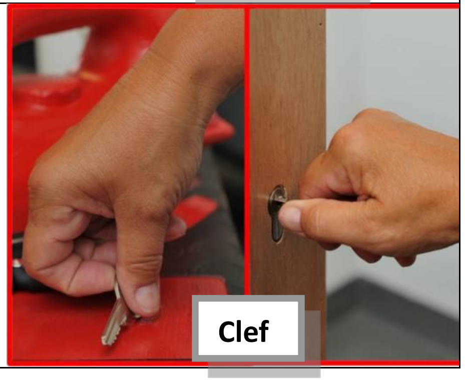
Clef

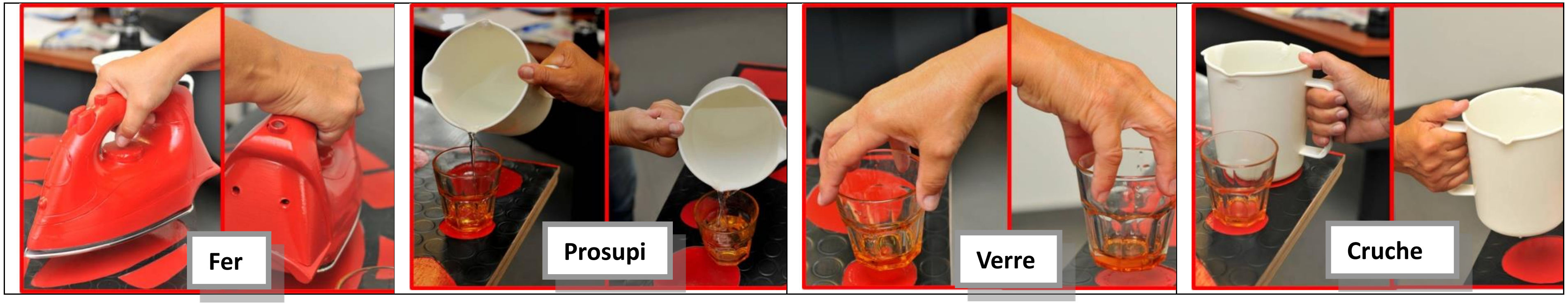

Cotation

La cotation nécessite d'observer la prise mais aussi le déplacement. Ainsi, le tableau à double entrée permet de noter rapidement après une bonne observation.

COTATION	PRISE CONFORME, PRECISE ET HARMONIEUSE	PRISE CONFORME MAIS LENTE OU IMPRECISE OU DISHARMONIEUSE	PRISE AVEC EXCLUSION OU COMPENSATION	PRISE IMPOSSIBLE
DEPLACEMENT RAPIDE ET PRECIS	3	2	1	0

**DEPLACEMENT LENT OU IMPRECIS**	**2**	**2**	**1**	**0**
**DEPLACEMENT AVEC COMPENSATION**	**1**	**1**	**1**	**0**
**DEPLACEMENT NON REALISE**	**0**	**0**	**0**	**0**

Ainsi, il suffit que la prise seule ou le déplacement seul soit incorrect pour que l'item soit noté avec le mode le plus péjoratif.

Un patient qui présente une séquelle définitive (amputation digitale, arthrodèse…) et effectue les items dans des conditions proches de la normale, avec une utilisation optimale de ses possibilités restantes. Dans ce cas, les compensations indispensables sont permises.

Attention !!

Le temps moyen pour réaliser cette épreuve est inférieur ou égal à 1 minute pour une main saine.
En cas de dépassement de temps, le score n'est pas modifié, cependant il sert d'information supplémentaire pour l'ergothérapeute quant à la rééducation de son patient.

Mode de calcul

Chaque item est noté de 0 à 3.
Le total de l'épreuve est obtenu en faisant le total des 20 notes : ce total correspond à la somme des notes du côté lésé.
Cette somme est ensuite divisée par 60 (score maximum) puis multipliée par 100 pour obtenir **le pourcentage d'utilisation mono manuelle de la main lésée du patient par rapport à une main saine.**

Exemple

- La somme des notes du côté sain est égale au score maximum c'est-à-dire 60 points.
- Ici la somme des notes du côté lésé est égale à 46 points.

Puis, une règle de trois est faite en divisant la somme des notes du côté lésé par le score maximal et en la multipliant

par 100 : $\frac{46}{60}$ **x** 100 = 76,6%

Attention !!!

Lors du calcul du résultat final, il convient d'arrondir au chiffre en dessous quand la décimale se trouve inférieure ou égale à 5 et au chiffre au-dessus quand la décimale se trouve supérieure à 5.

Ainsi, 76,6 devient **77/100.**

Interprétation : on peut dire que ce patient a une utilisation monomanuelle de 77% pour sa main lésée par rapport à ses possibilités antérieures.

EPREUVE 4 : FONCTION BIMANUELLE

Il s'agit de tester la fonction bi manuelle du patient au moyen de 20 tâches de la Vie Quotidienne et d'en vérifier le respect de sa Dominance. Cette épreuve est faite au rythme du patient, sans chronométrage et sans consigne particulière. Toutefois, cette épreuve ne devrait pas excéder15 minutes.

Matériel

1. Une assiette
2. De la pâte à modeler
3. Des couverts courants
4. Bocal à confiture (plein de particules et couvercle vissé)
5. Bouteille d'eau (50 ml) à capsule vissée
6. Boulon et écrou de 4 mm
7. Tube de médicament à couvercle serti
8. Chemise sur planche avec 3 boutons de 12mm de diamètre
9. Planche avec 3 lacets
10. Aiguille avec gros chas et fil
11. Grosse boîte d'allumettes
12. Porte-monnaie (avec bouton pression et fermeture éclair)
13. Feuille de papier A4
14. Stylo bille « bic »
15. Règle de section carrée (22 cm de long)
16. Carton (1mm d'épaisseur et 20 cm long)
17. Ciseaux de bureau
18. Tige de métal cuivré (10/10^{me}mm)
19. Pince coupante en bout
20. Feuilles de journal (4 doubles feuilles)

Mode d'emploi

L'ergothérapeute donne successivement les 20 tâches à réaliser, en prenant soin de poser sur la table, devant le patient, le matériel nécessaire pour chacun des items. Il lui explique qu'il doit faire comme d'habitude sans lui donner d'autre consigne que celles qu'il va lui fournir en même temps que le matériel de bilan:

« Faites le plus naturellement possible, comme vous le pouvez, comme vous le feriez chez vous, en ce moment, si vous étiez   seul à la maison, sans personne pour vous aider…….. »

1- « Avec les couverts, coupez 3 petits morceaux de pâte à modeler dans votre assiette comme si c'était un beefsteak. » 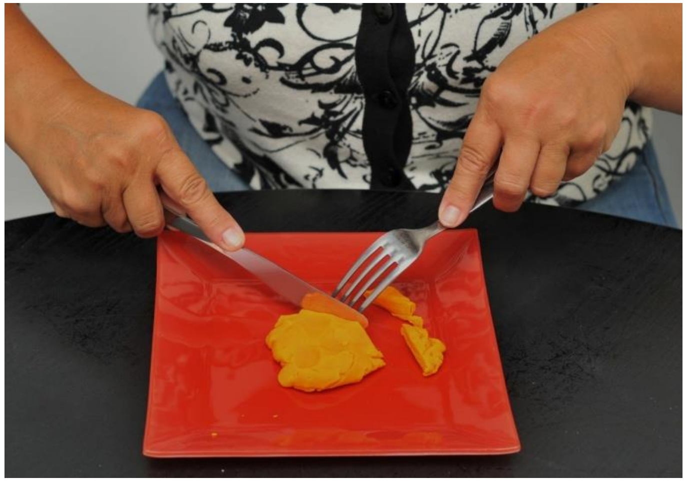	***2- « Ouvrez cette bouteille d'eau (500ml) et refermez la, le couvercle est vissé »*** 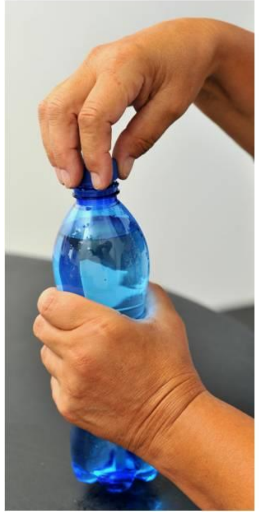 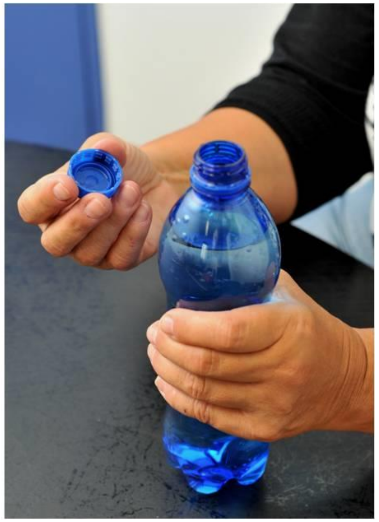
3 - ***« Ouvrez ce bocal de confiture et refermez le »***(au préalable, il a été vissé à fond)	***4 - « Débouchez ce tube et refermez le, attention le couvercle n'est pas vissé, il se clipse. »***

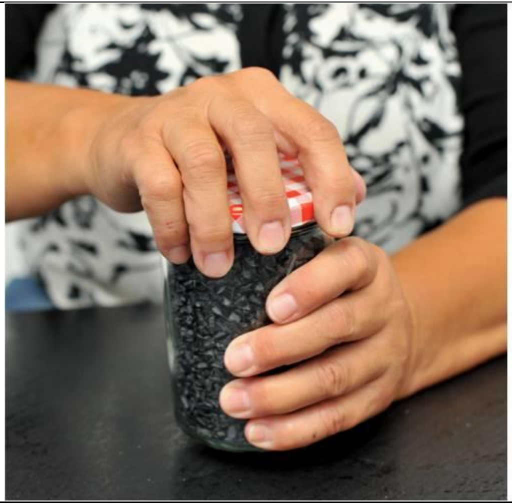
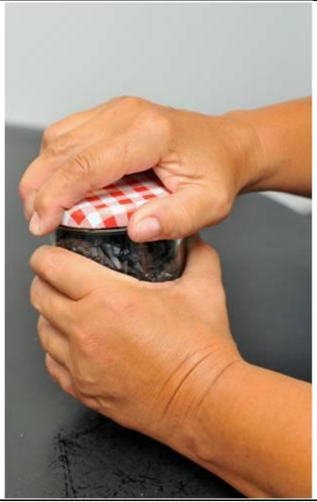

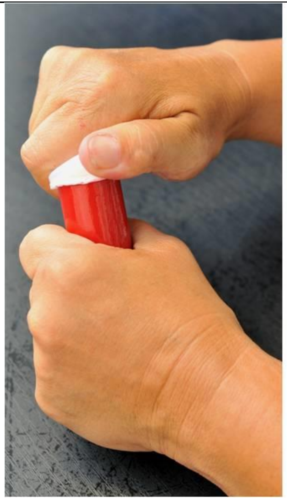
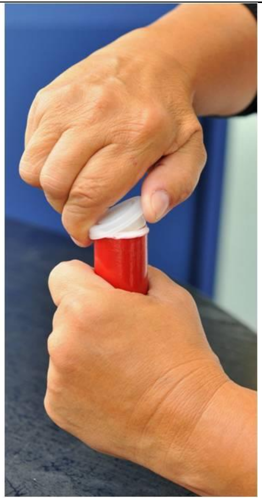

5- « Défaites les 3 boutons de cette chemise. »
6- « Remettez les boutons. »

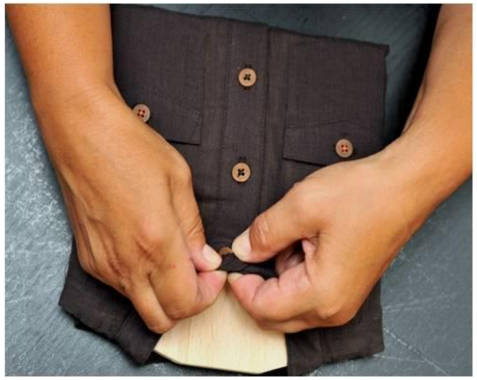
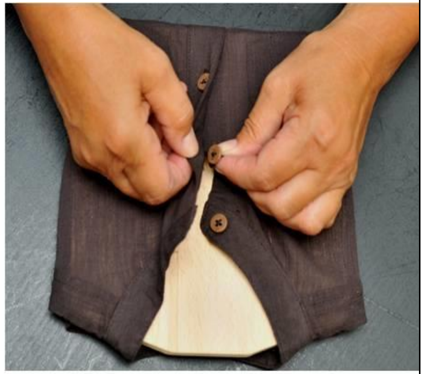

7- « Défaites ces 3 lacets. »
8- « Refaites-les. »

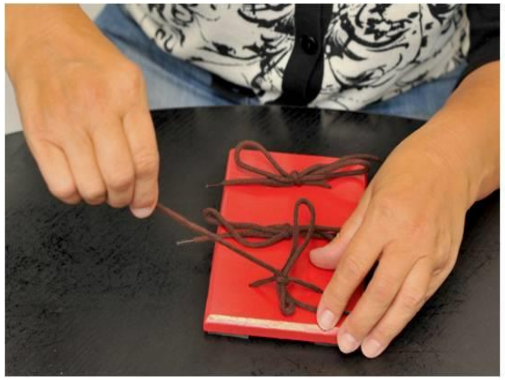
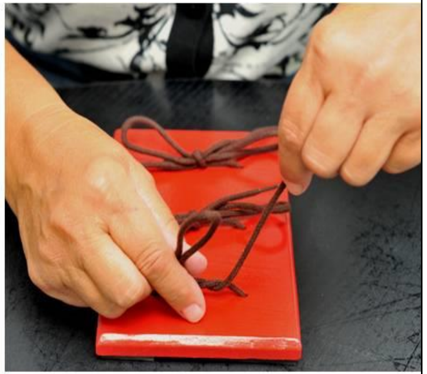

9- « Dévissez cet écrou du boulon, ôtez le et revissez le

10- « Enfilez le fil dans cette aiguille. »

au moins jusqu'à la moitié » 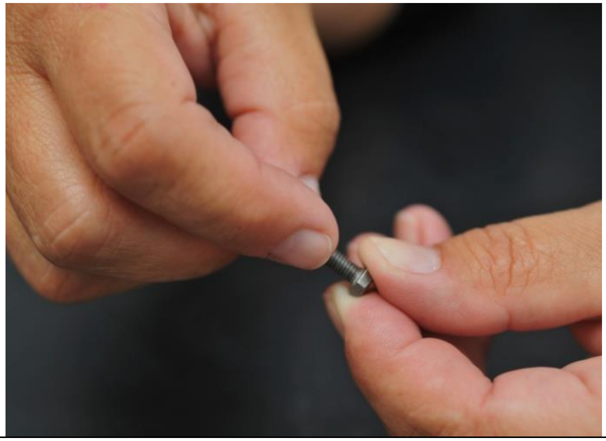	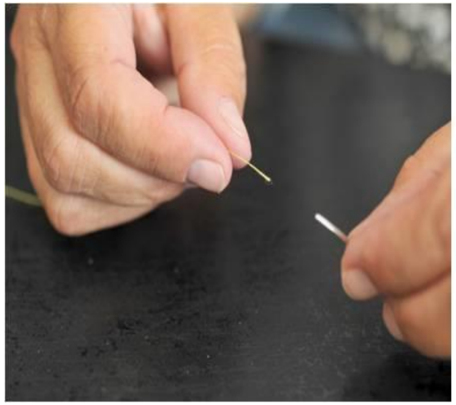 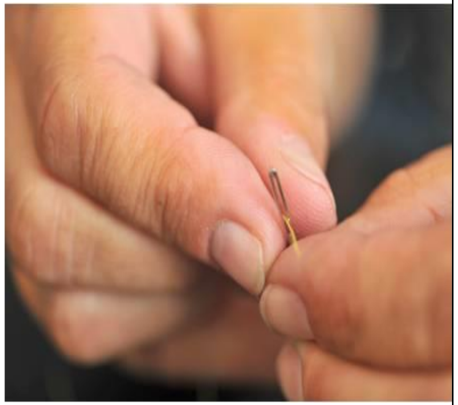

11-« « Ouvrez cette boîte d'allumettes, sortez en une, allumez la. »	*12-«Éteignez votre allumette sans souffler, mais en la secouant »*

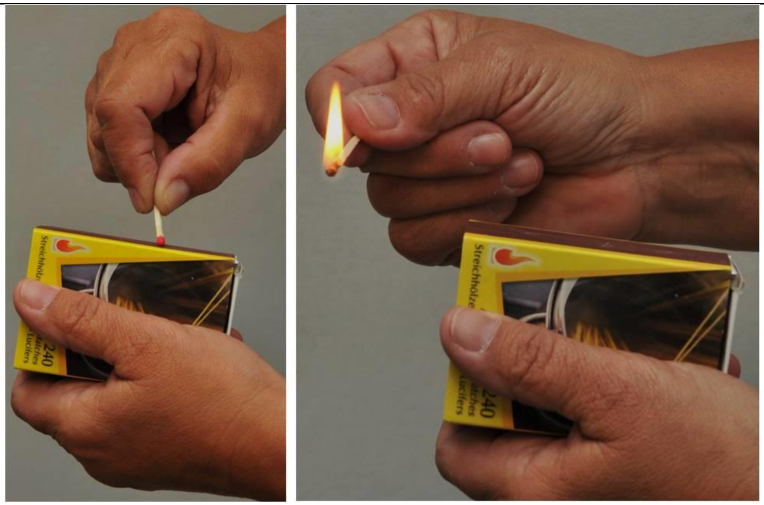	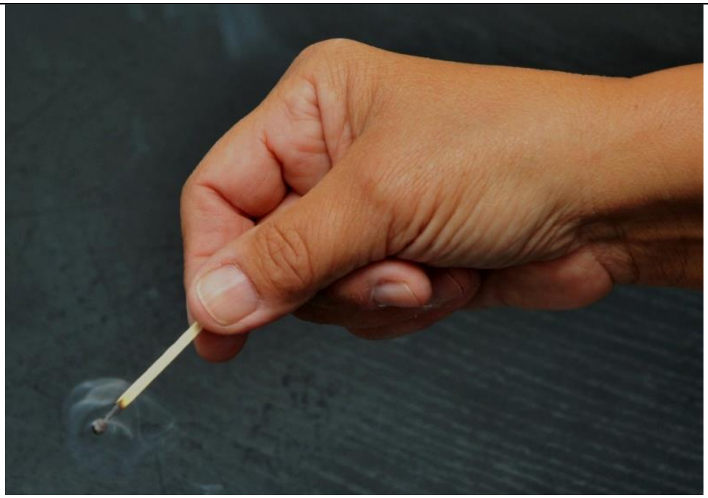
13- « Ouvrez la fermeture éclair de ce porte-monnaie, changez les 5 pièces de compartiment, refermez le. Ouvrez le porte-monnaie du côté du clips , sortez le billet, dépliez-le, repliez-le et remettez le dedans. » 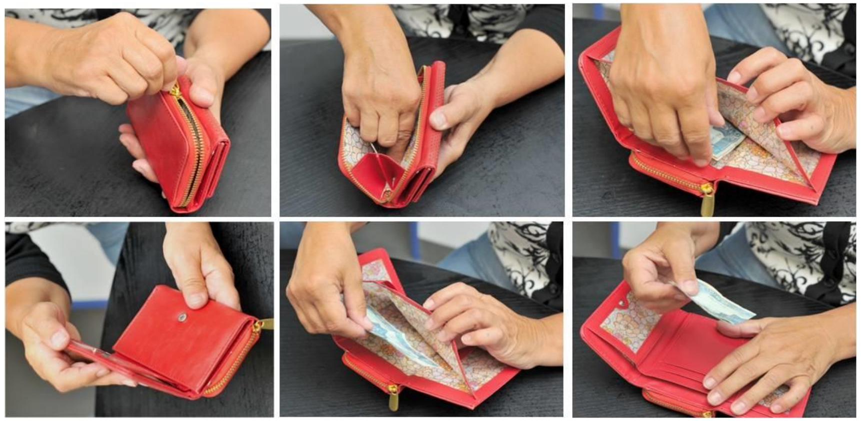	***14- « Écrivez cette phrase »:*** la main est un des plus beaux atouts de l'homme et de la femme 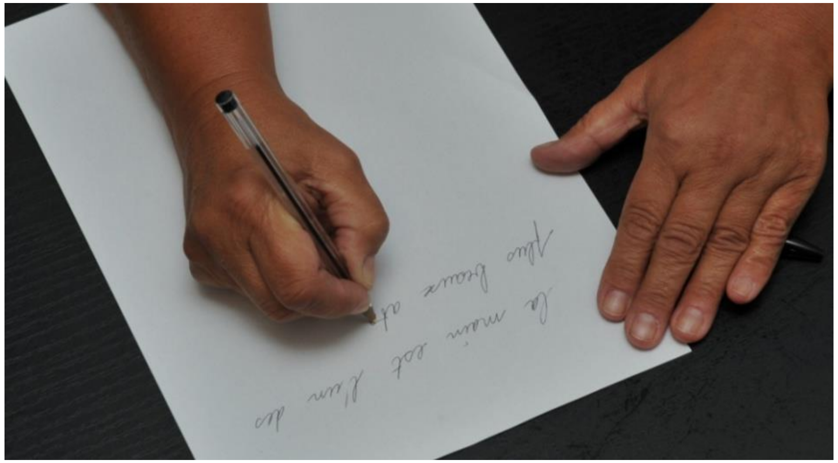

15-« Tracez un trait avec cette règle. »<br>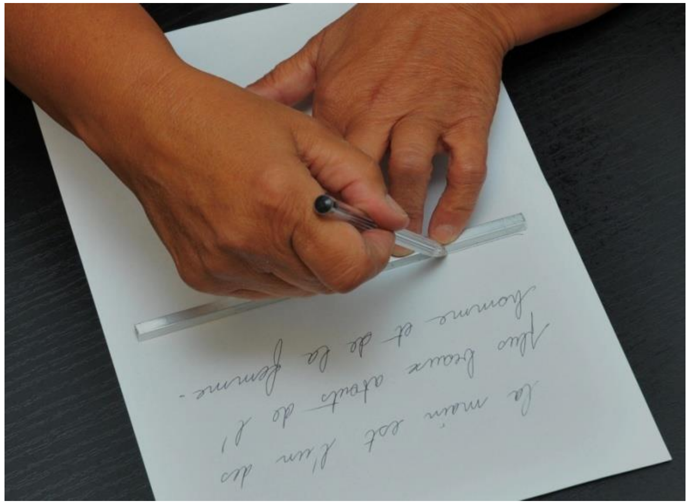	***16- « Pliez la feuille sur le trait que vous venez de faire. »***<br>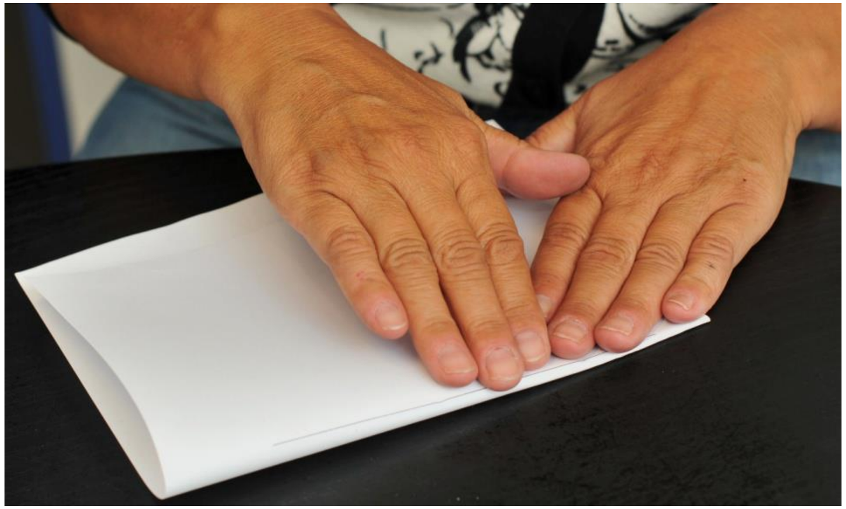
17- « Déchirez la feuille le long de la pliure. »	***18-« Coupez une bandelette de carton avec ces ciseaux. »***

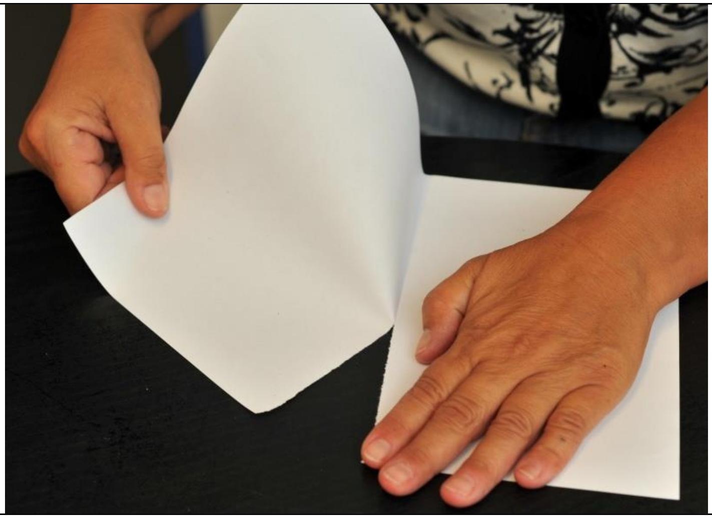	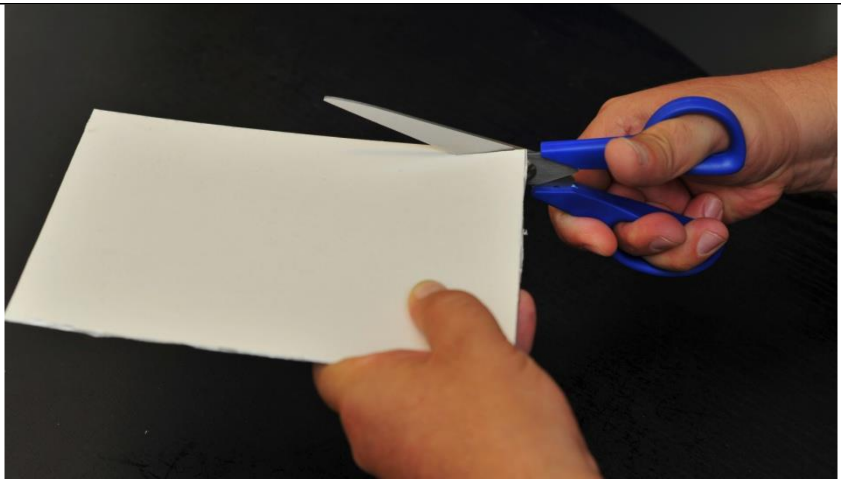

19-« Coupez 3 petits morceaux de métal avec cette pince coupante. »	***20-« Essayez de déchirer ce journal en son milieu et de ce côté. »*** (Côté charnière, 4 doubles feuilles pliées 3 fois soit 32 épaisseurs de feuille)

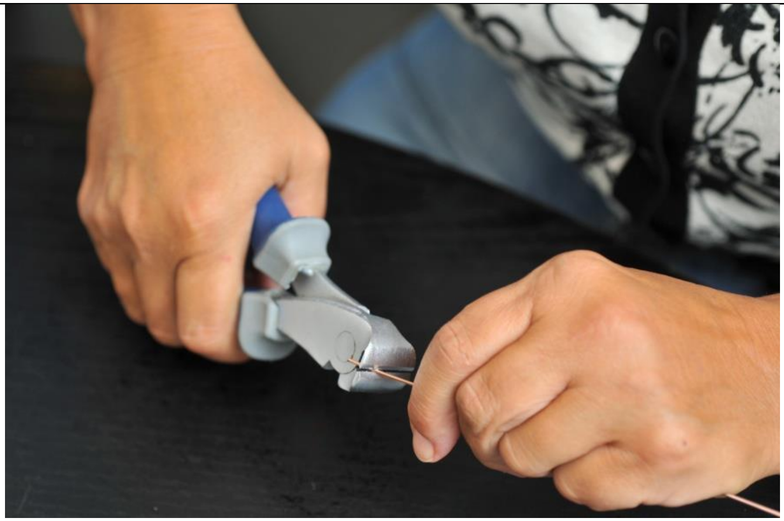

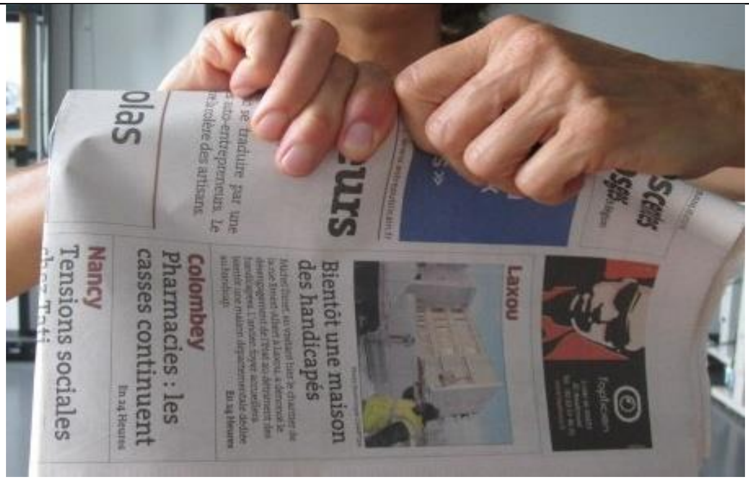

Cotation

COTATION EPREUVE 4	GESTE NORMAL*	GESTE COMPLET MAIS LENT OU IMPRECIS	GESTE INCOMPLET OU AVEC EXCLUSION OU AVEC COMPENSATION	GESTE IMPOSSIBLE
	3	2	1	0

GESTE NORMAL* : dans le cas d’amputation ou d’arthrodèse, les compensations indispensables sont admises et peuvent conduire à une cotation "geste normal".

Cette épreuve sert également à observer **le transfert de dominance** éventuel, surtout quand la main dominante est lésée.

Par exemple, un patient droitier, se servant de sa main gauche pour ouvrir un bocal mais utilisant bien sa main droite pour le maintenir, sera noté « 3 » mais ponctué d’un signe **« -».**
Ce qui signifie : « conforme » mais transfert de dominance.
Si la dominance du patient est bien respectée, il sera noté « 3 » ponctué d’un signe **« + ».**

Ces observations concernant la dominance n’influencent en rien sur les notes, simplement elles donnent des indications complémentaires pour orienter la suite de la rééducation éventuelle.

Mode de calcul

- Chaque item est noté de 0 à 3.

- Le total des 20 notes est obtenu en les additionnant.

- Ce résultat est alors divisé par 60 (score maximum) et multiplié par 100 pour obtenir le pourcentage d'utilisation bi manuelle du patient par rapport à une personne ayant les 2 mains saines.

Par exemple :

- Somme des notes obtenues par le patient = 47 points
- Score maximum = 60 points

- On fera : $\frac{47}{60} \times 100 = 78{,}3 / 100$

Attention !!!

Lors du calcul du résultat final, il convient d'arrondir au chiffre en dessous quand la décimale se trouve inférieure ou égale à 5 et au chiffre au-dessus quand la décimale se trouve supérieure ou à 5.

- Ainsi, 78,3 devient **78/100.**

Interprétation : on peut dire que le patient a une fonction bimanuelle de 78% par rapport à une personne ayant toutes ses possibilités.

MODE DE CALCUL GLOBAL DU BILAN

Le score global est obtenu en faisant la somme des résultats obtenus dans chacune des 4 épreuves.

Exemple :

- Epreuve de mobilité de la main = 77
- Epreuve de force de préhension = 52
- Epreuve mono manuelle = 77
- Epreuve bi manuelle = 78

On fera : 77 + 52 + 77 + 78 = **284 points / 400 points (score maximum)**

On peut également diviser ce résultat par 4 pour avoir un chiffre sur 100 points ou pourcentage plus facile à retenir : 284 soit 284/4 = **71 %**

Ces résultats peuvent être affichés sous forme d'histogramme pour rendre la lecture agréable et facile aussi bien pour les patients que pour les différents intervenants de l'équipe médicale et para médicale.

Interprétation : on peut dire que la capacité d'utilisation fonctionnelle de la main lésée du patient est de 71% par rapport à une main saine ou qu'elle est diminuée de 29% par rapport à une main saine.

Le Bilan 400 Points objective l'évolution du patient, en soulignant les progrès ou au contraire en marquant la nécessité de continuer l'effort entrepris.
Par ailleurs, au delà de l'évaluation globale, il met l'accent sur les secteurs particulièrement déficitaires permettant de réajuster au mieux le traitement de rééducation ou d'orienter vers un traitement chirurgical.

MATERIEL NECESSAIRE

Epreuve 1

- Un coussin triangulaire est utilisé pour les 3 items contre résistance.
- Une carte de type carte à jouer ou carte bancaire
- Trois cylindres de 22 cm de long :
  - un de 3 mm de diamètre
  - un de 10 mm de diamètre
  - un de 20 mm de diamètre

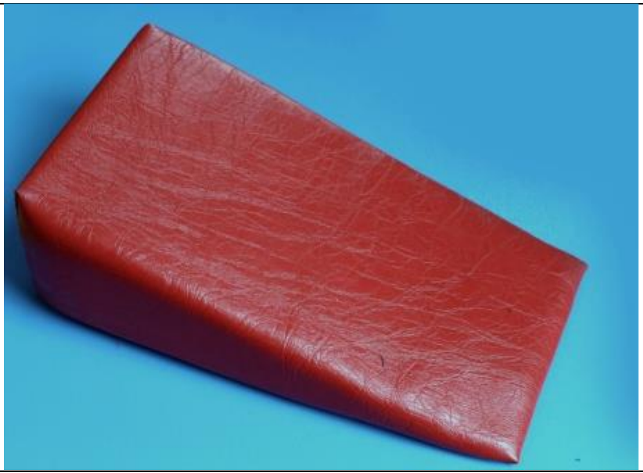

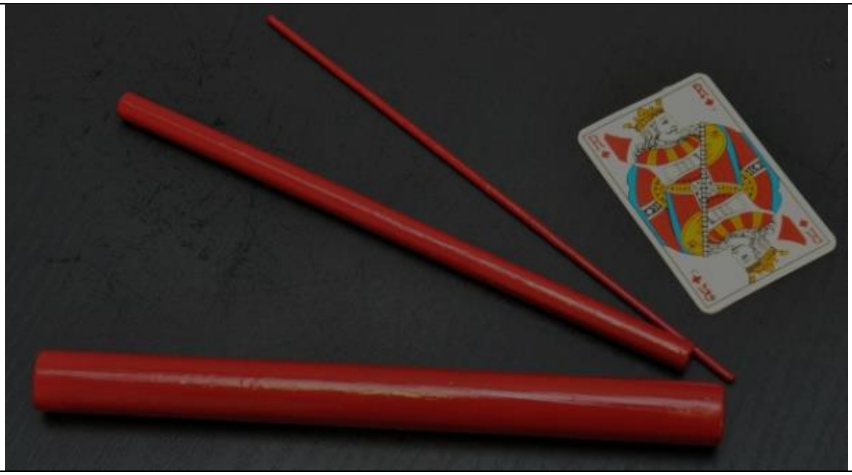

Epreuve 2

- Un dynamomètre Jamar
- Un Pinchmètre Jamar

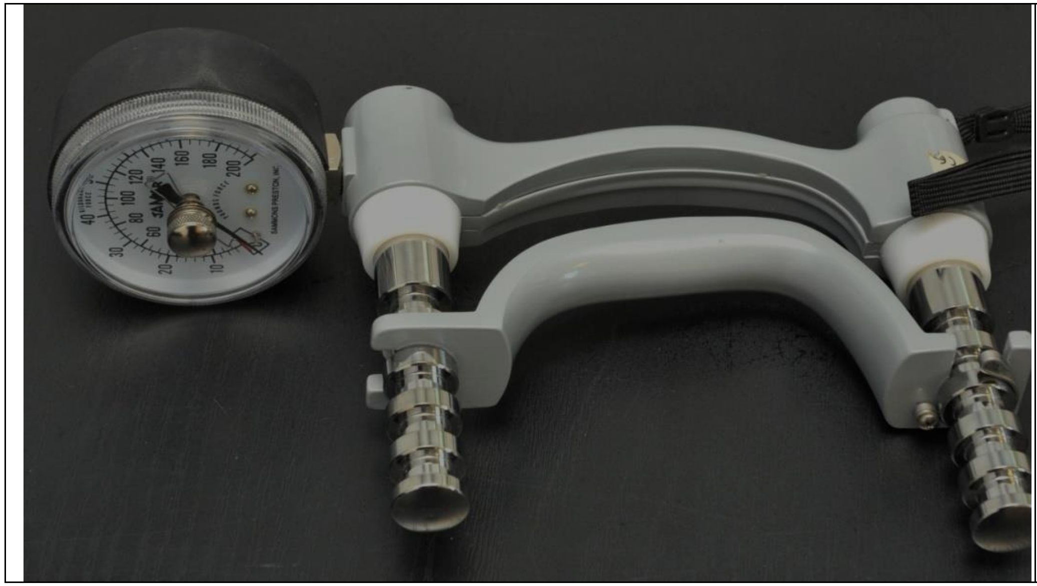

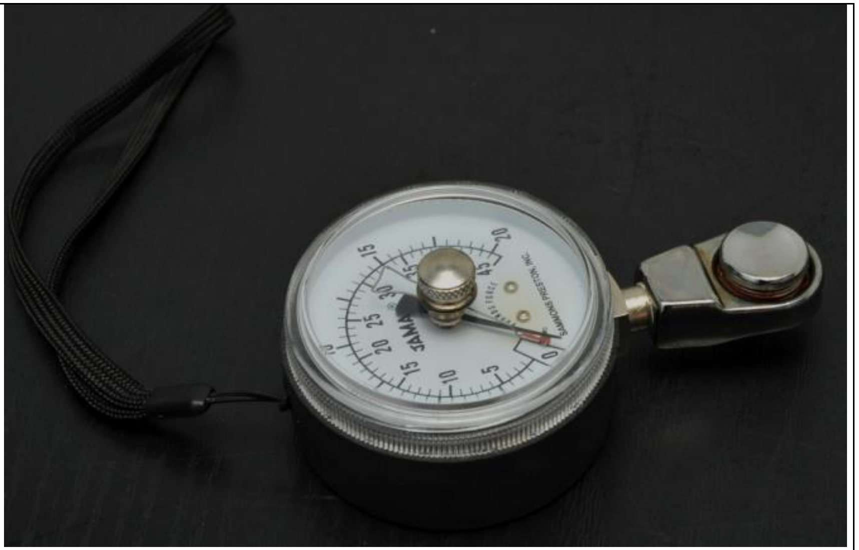

Epreuve 3

Un chronomètre

Un cube de 10 cm de côté (700gr)
Un cube de 7,5 cm de côté (300 gr)
Un cube de 5 cm de côté (100 gr)
Un cube de 2,5 cm de côté (10 gr)

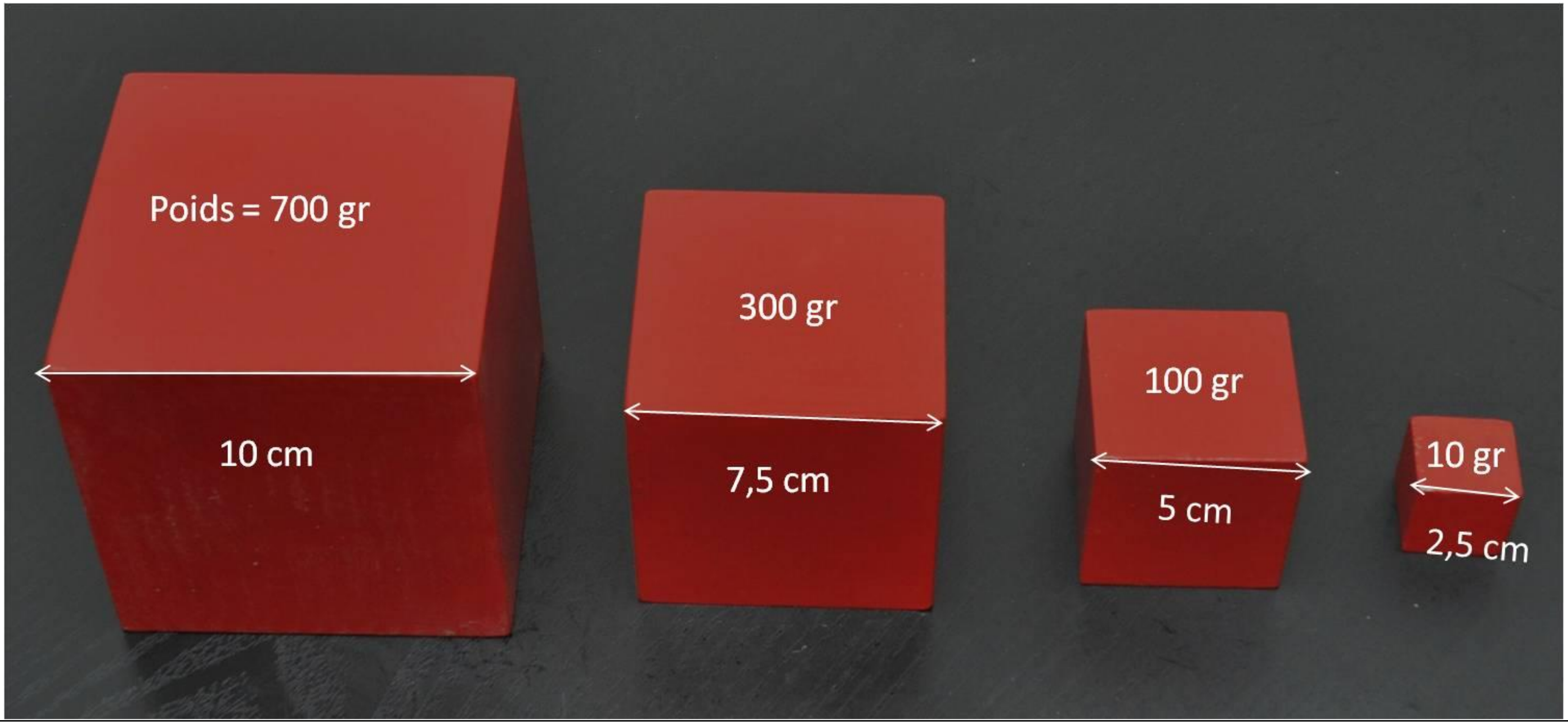

Un cylindre de 10 cm de diamètre et 12 cm de haut (700 gr)
Un cylindre de 7,5 cm de diamètre et 11 cm de haut (300 gr)
Un cylindre de 5 cm de diamètre et 10 cm de haut (100gr)
Une bille de 25 mm de diamètre

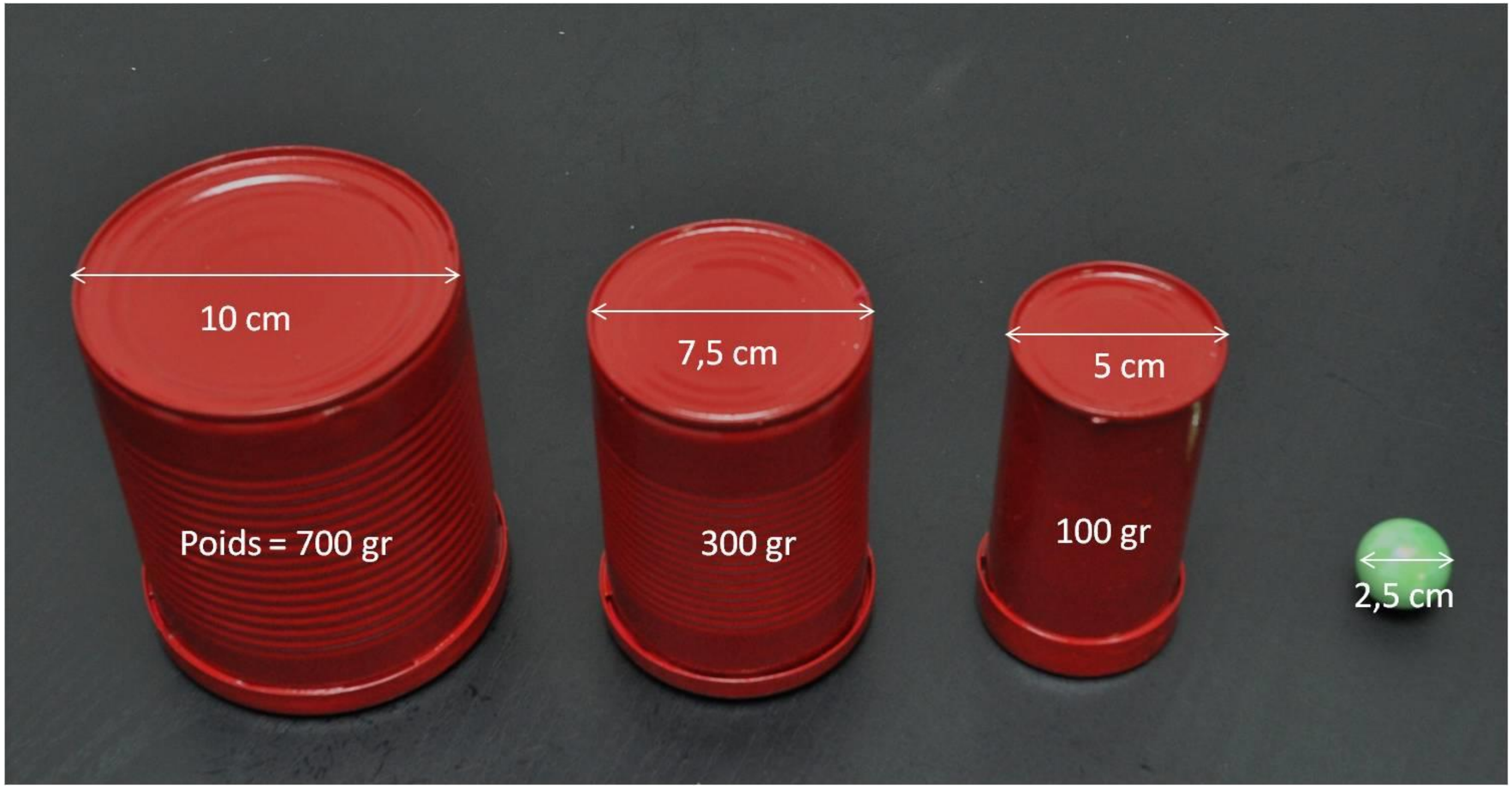

Une balle de tennis
Une pièce de monnaie de 30 mm de diamètre
Une pièce de monnaie de 25 mm de diamètre
Une pièce de monnaie de 15 mm de diamètre
Une bille de 16 mm de diamètre
Une pointe de 4 cm de long et 2 mm de diamètre
Un briquet électronique

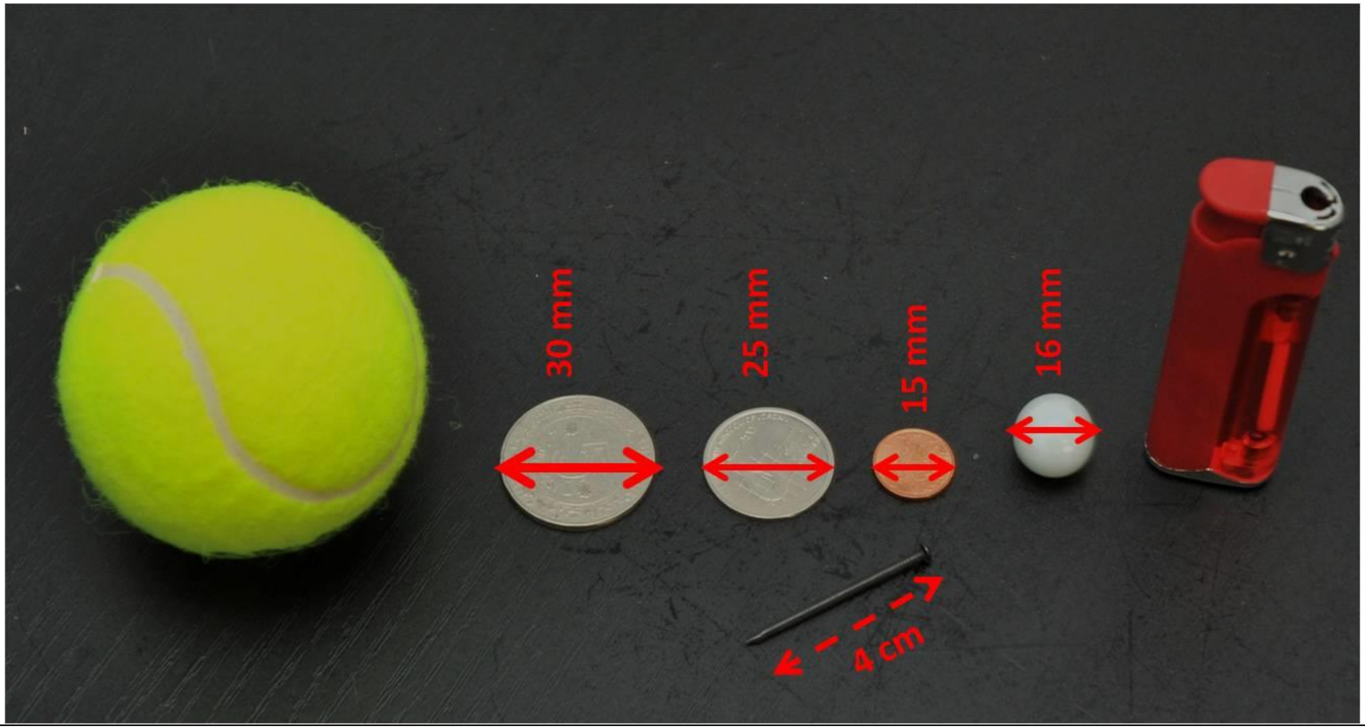

Une clef plate et la serrure correspondante et un fer à repasser de 2kg

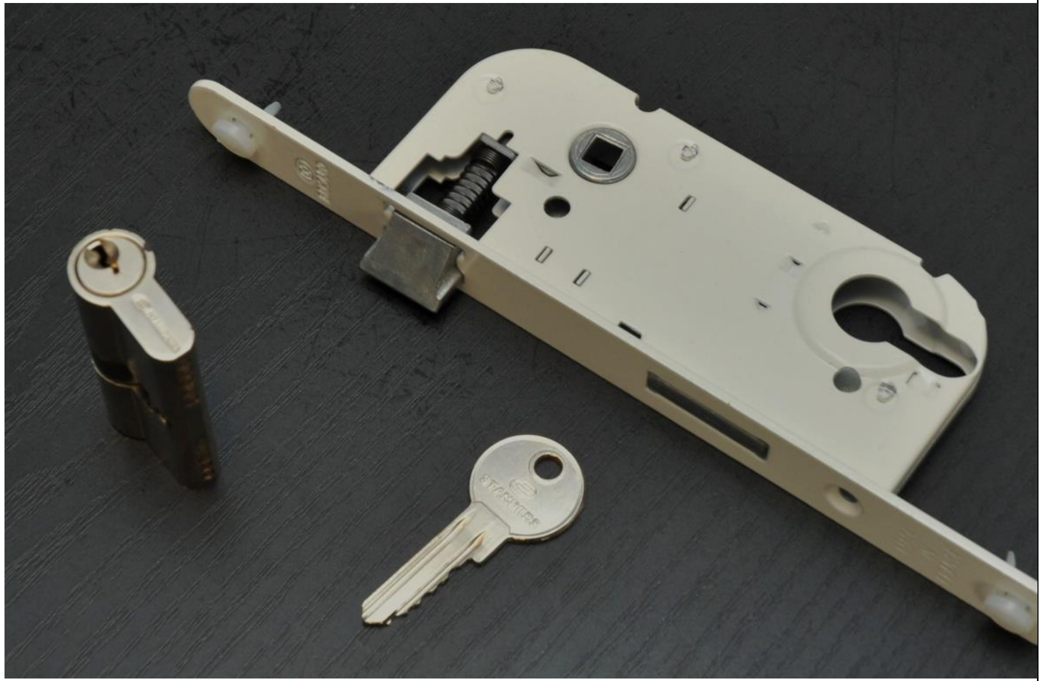	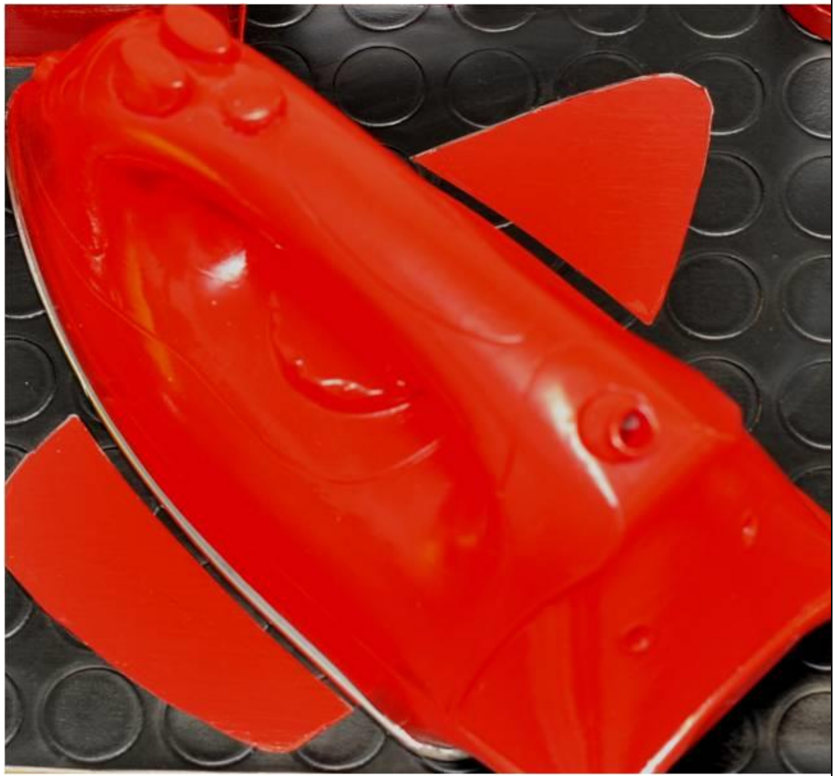

Une cruche munie de 2 becs verseurs avec 500 ml d'eau
Un verre ordinaire

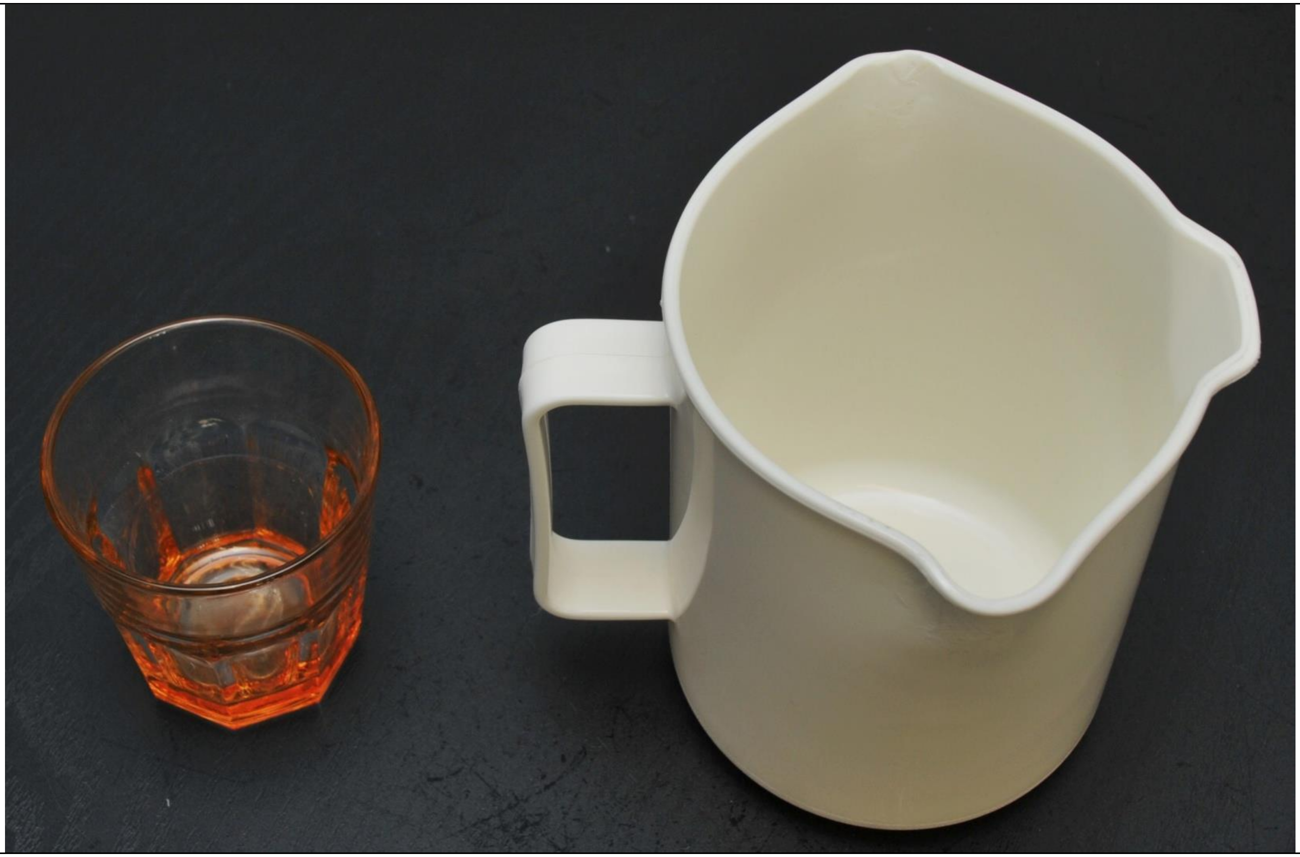

Epreuve 4

Une assiette et de la pâte à modeler ainsi que des couverts courants et de la pâte à modeler.

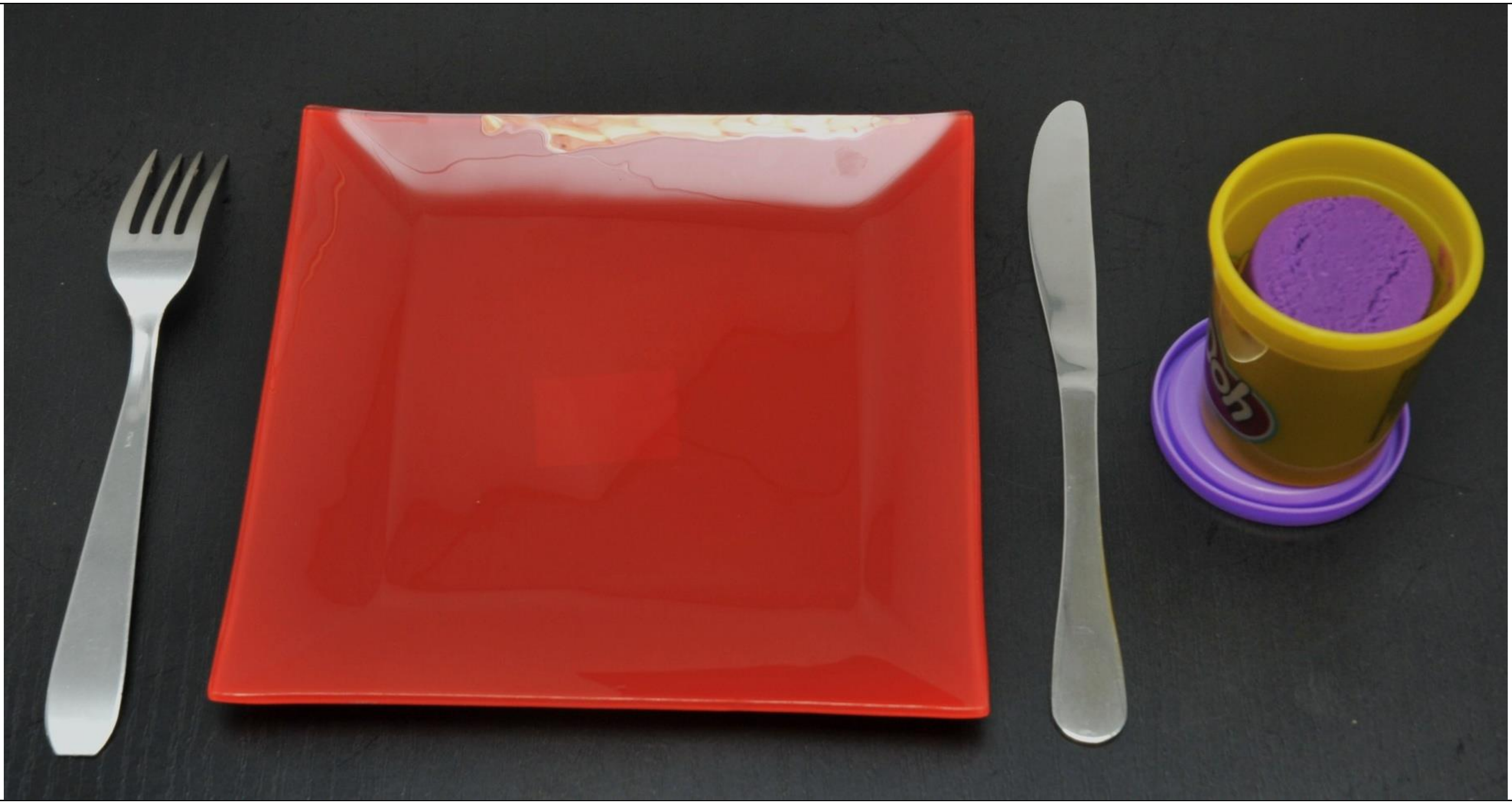

Un bocal à confiture (plein de particules et couvercle vissé) ;
Une bouteille d’eau (50 ml) à capsule vissée
et un tube de médicament à couvercle serti

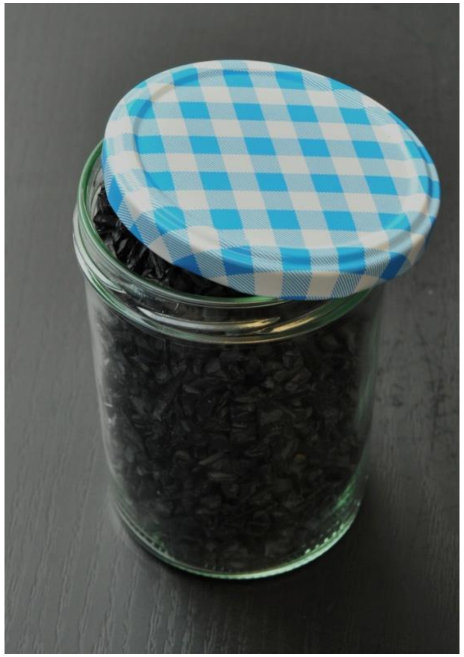 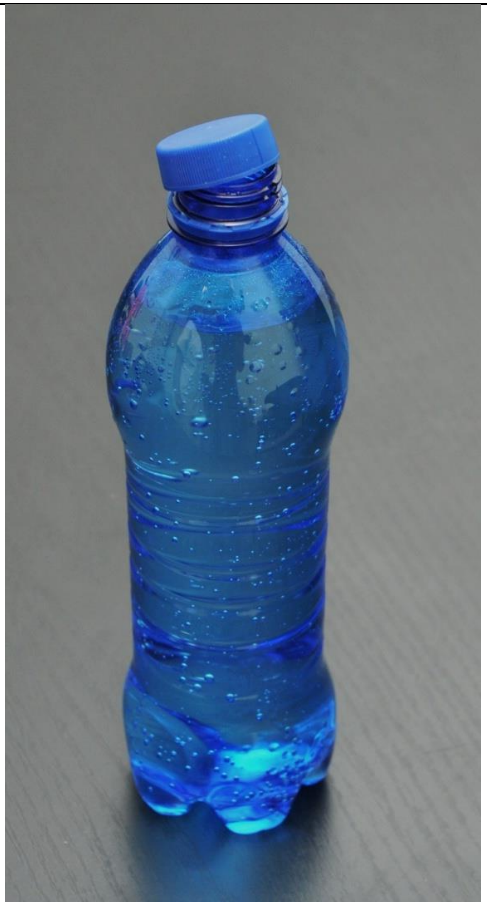 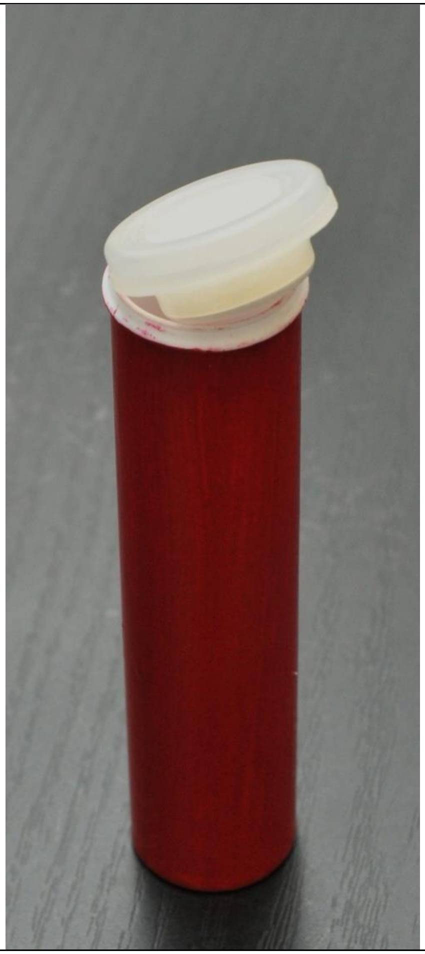

Un boulon et écrou de 4 mm
Une grosse boîte d’allumettes

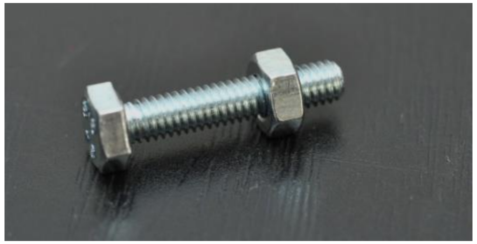	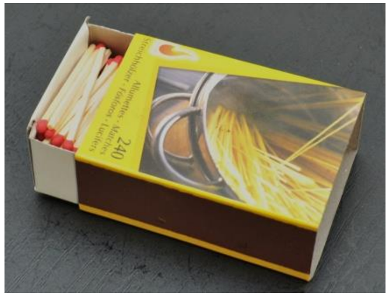

Une chemise sur planche avec 3 boutons de 12mm de diamètre et une planche avec 3 lacets
Une aiguille avec gros chas et fil

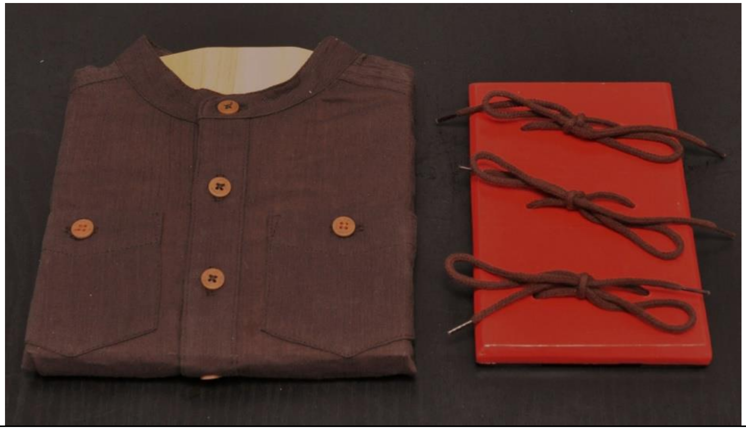	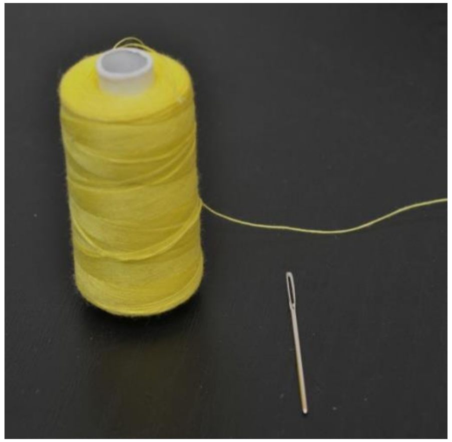

Un porte-monnaie (avec bouton pression et fermeture éclair)

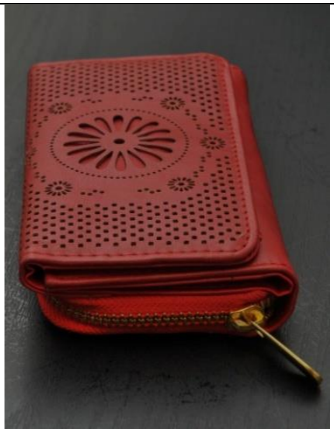
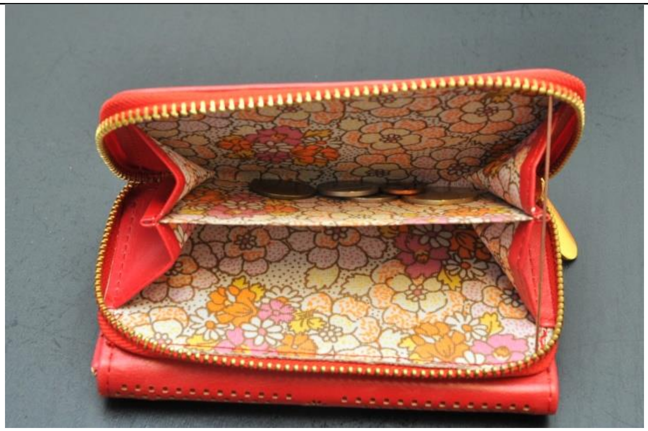
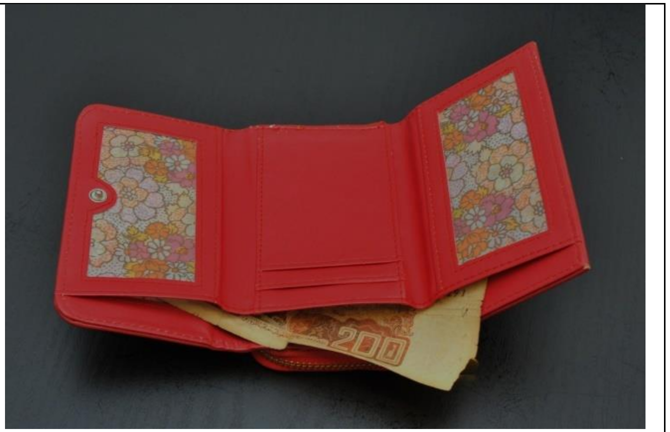

Une feuille de papier A4 avec un stylo bille « bic » et une règle de section carrée (22 cm de long)
Des feuilles de journal (4 doubles feuilles)

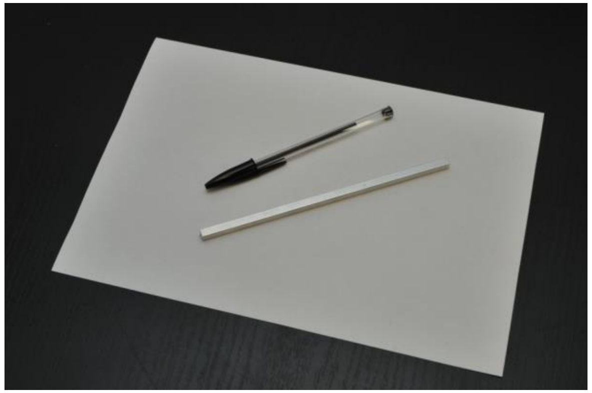
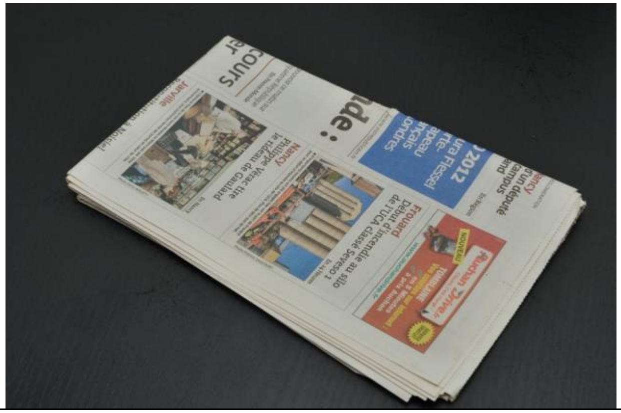

Du carton (1mm d’épaisseur et 20 cm long) et des ciseaux de bureau
Une tige de métal cuivré ($10/10^{me}$mm) ainsi qu’une pince coupante en bout

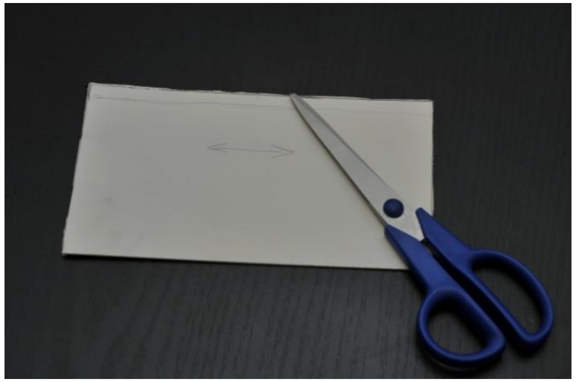

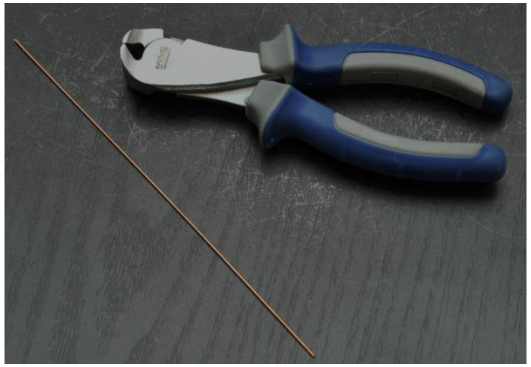

Supplement: Supplementary file 1 — Additional file 1. [file 12891_2020_3303_MOESM1_ESM.pdf]
