# Supplementary material for: Prospective multicentre validation study of a new standardised version of the 400-point hand assessment
Source: BMC Musculoskelet Disord. 2020 May 20;21:313. doi: 10.1186/s12891-020-03303-4 (PMC7240941; doi:10.1186/s12891-020-03303-4)
Supplement: Supplementary file 2 — Additional file 2. [file 12891_2020_3303_MOESM2_ESM.pdf]

400-POINT ASSESSMENT

(V 2)

Ms Colette Gable,
occupational therapist
Professor Jean Paysant

Institut Régional de Médecine Physique et de Réadaptation
(Regional Institute of Physical Medicine and Rehabilitation)
75, Boulevard Lobau 54042 NANCY

INTRODUCTION

The assessment of prehension ability, in all its complexity, remains a real challenge. The conventional use of assessments oriented towards measuring one particular aspect of prehension function (articular, muscular, trophic or sensory) only provides a partial and fragmented response to the question of prehension ability.

The “400-POINT Assessment” offers a four-fold evaluation covering motor function, strength, one-handed grasp, and two-handed coordination. The observation of these during approximately 60 everyday movements provides information about prehension quality and means of adaptation. The score awarded to each test “points to” the area of deficit.

A period of 30-45 minutes is needed to carry out this assessment.

DESCRIPTION

This assessment comprises four tests, each graded out of 100 points:

1 – HAND MOBILITY..
2 – PREHENSION STRENGTH...
3 – ONE-HANDED GRASP AND OBJECT TRANSFER..
4 – TWO-HANDED FUNCTION..

Each test has its own:

- ✓ equipment,
- ✓ procedure,
- ✓ grading system,
- ✓ calculation method.

In order to be reliable, this assessment must be performed in constant conditions using identical equipment always set up in the same way:

- A height-adjustable table (approx. 60cm in width and 90cm in length).
- A stable seat (stool with feet pads), adjustable in height, for the patient.
- A stool with casters, adjustable in height, for the occupational therapist.
- A shelving unit to keep all objects needed within reach.
- A height-adjustable table upon which two reference surfaces are installed 50cm apart; the table is adjusted so that the upper surface is at shoulder height to the patient undergoing the test.

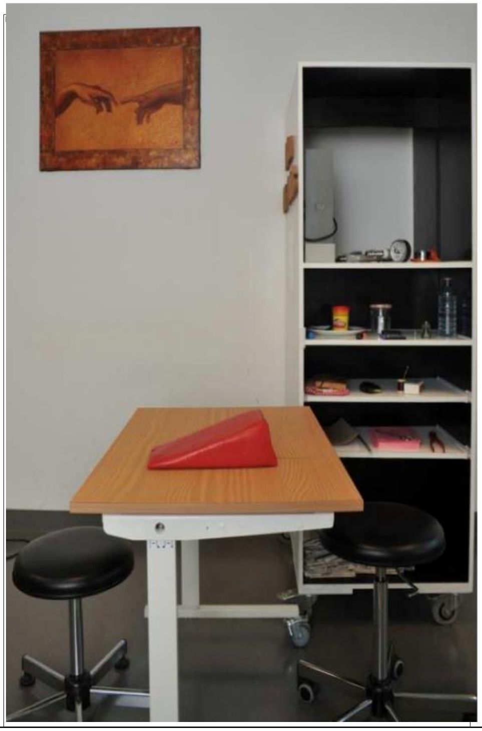
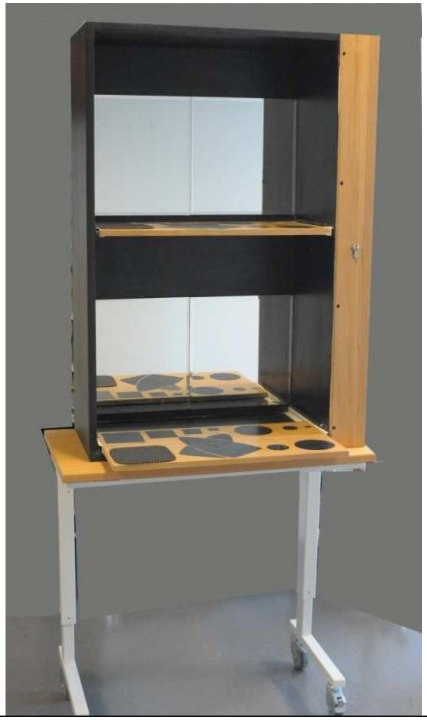
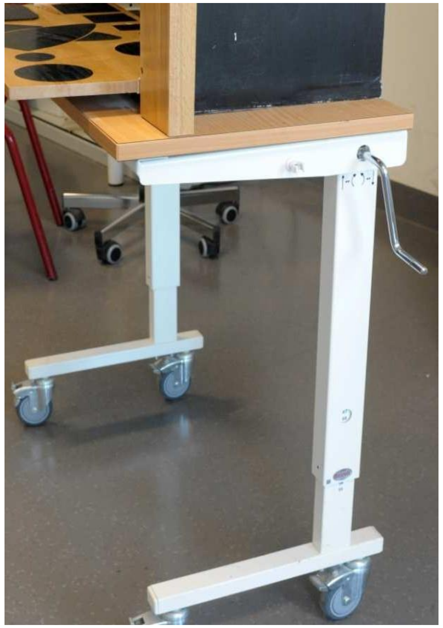

TEST 1: HAND MOBILITY

This test evaluates both hands of the patient at the same time by means of the 14 general movements below, grading the quality of execution for each one:

1. Closing the hand
2. Opening the hand
3. Spreading the fingers
4. Bringing the fingers together
5. Spreading the thumb
6. Adduction of the thumb
7. Opposition of the thumb to the four fingers
8. Opposition of the thumb to the four fingers against a resistance
9. Lateral pinch against a resistance using a card
10. Overall grip of three types of stick, against a resistance
11. Wrist flexion
12. Wrist extension
13. Pronation
14. Supination

Equipment

- A triangular cushion is used for the three exercises involving resistance
- A playing card or bank card
- Three cylinders 22cm in length:
    - one with a diameter of 3mm
    - one with a diameter of 10mm
    - one with a diameter of 20mm

Procedure

The patient sits comfortably at a table, facing the occupational therapist.

STARTING POSITION
**The forearms rest on the edge of the table, with the hands held slightly above the surface of the table.**

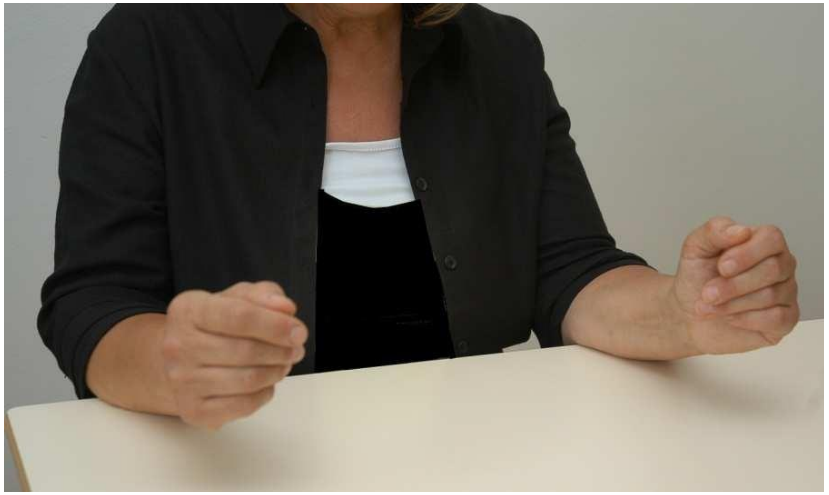

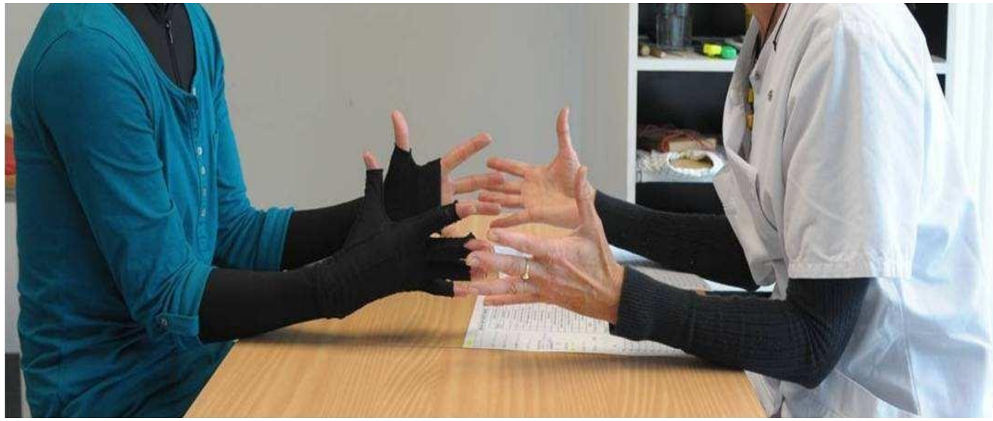

The occupational therapist explains the movements and shows them to the patient, who must reproduce them. Both hands are tested at the same time. For certain items, it is preferable for the patient to repeat some of the movements several times in a row or to event to test two movements at the same time so that they are more natural.

1 and 2: “Close both hands as if you wanted to throw a punch, then open and close them both repeatedly.”

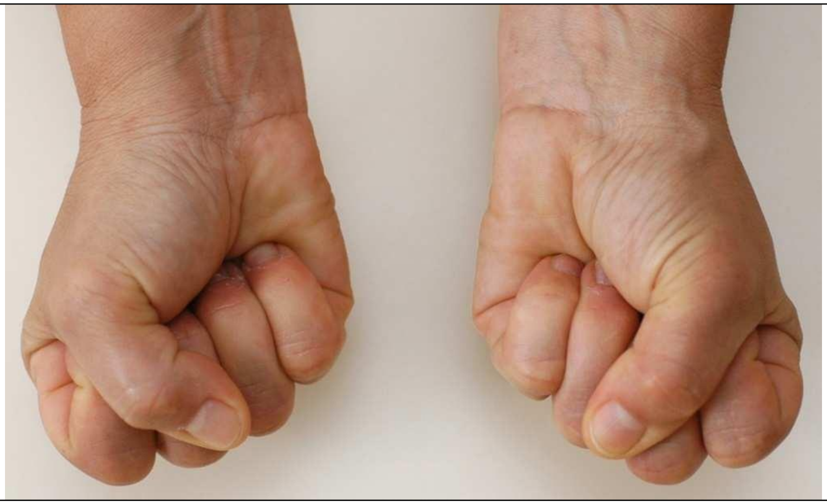

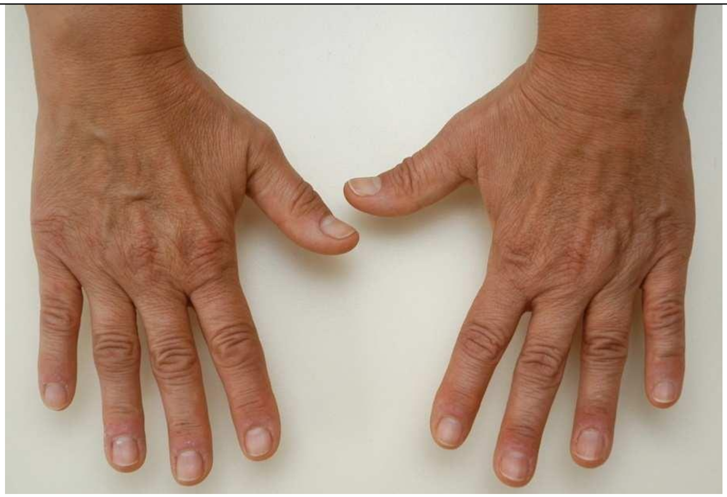

3 and 4: “Spread all four fingers of both hands and bring them together again. Repeat this several times.”

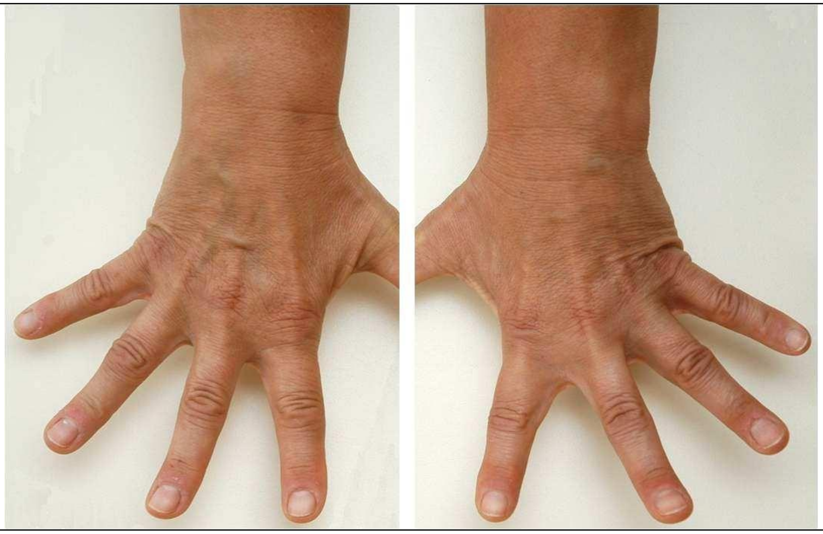

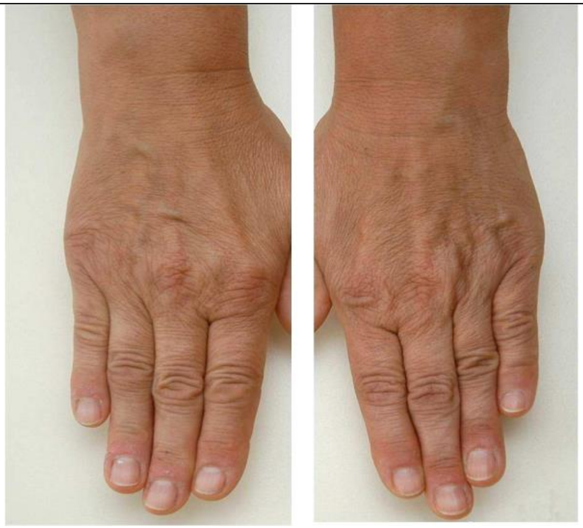

5 and 6: “Spread both of your thumbs and stretch them out in the same plane as the fingers. Keeping them stretched, draw them back in and slide across to reach the second commissure. Spread and draw in, then repeat, etc.”

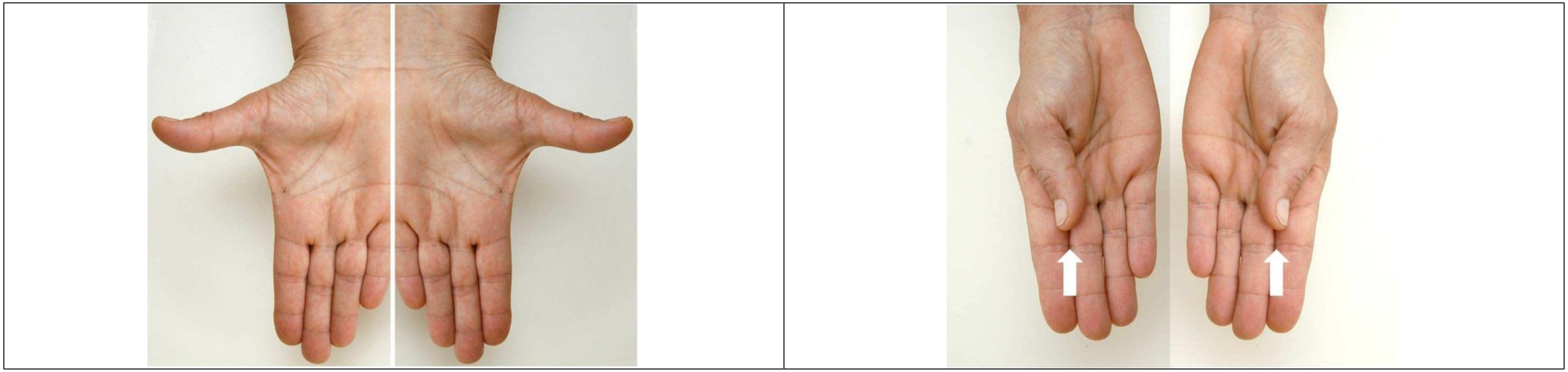

7: "With both thumbs, try to make perfect circles by touching the tip of each finger, then repeat, etc.”

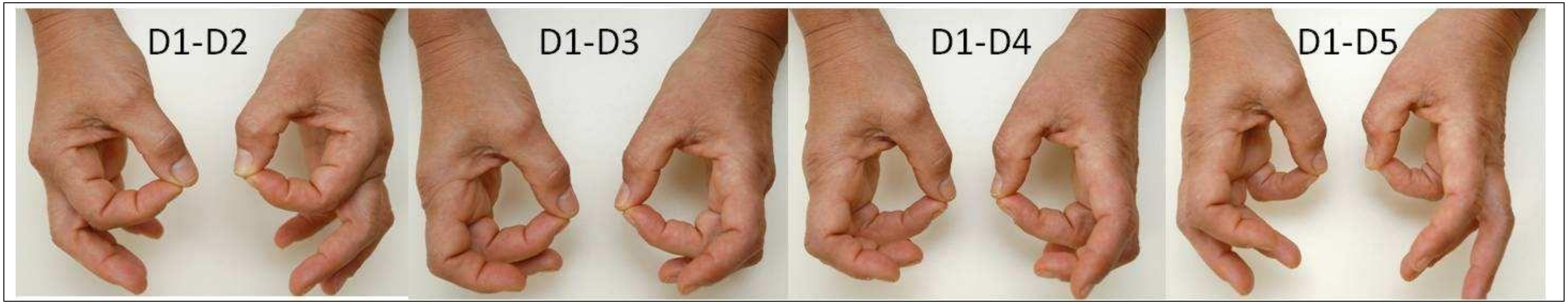

Please note:

An overall mark of 1 is awarded for this exercise even if one thumb-to-fingertip touch cannot be achieved.

For the three exercises with resistance, each hand is tested separately. The patient's forearm rests on a triangular cushion with the hand reaching over the side.
The occupational therapist begins by assessing the patient's healthy hand, then moves to the impaired hand, providing the necessary resistance.

8: "We're going to do the same circles, but this time, resist the pressure I apply. I will try to come between your thumb and finger (on the healthy hand first, then the impaired one)."

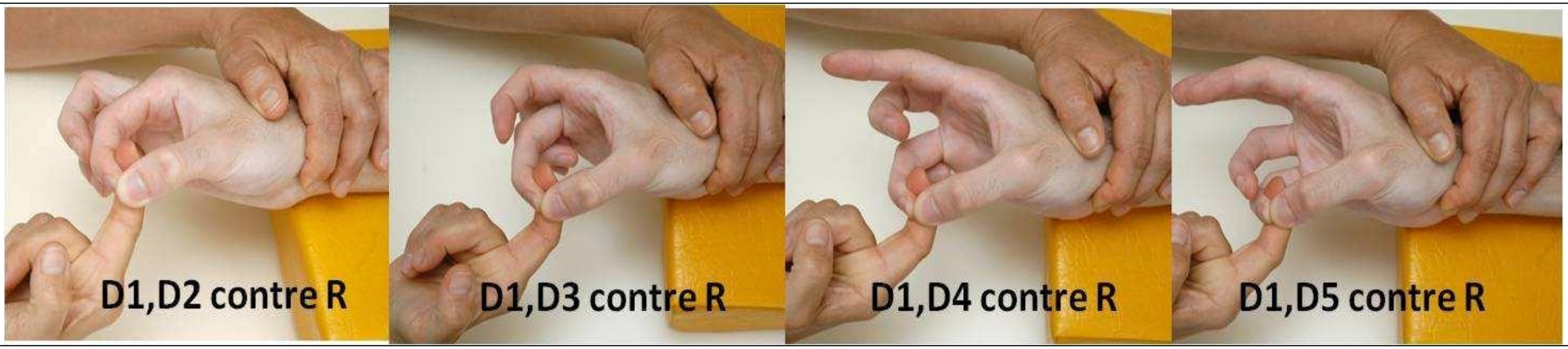

9: "Now try to hold this card between your fingers. I will try to pull it out from between them (firstly from your healthy hand, then the impaired one."

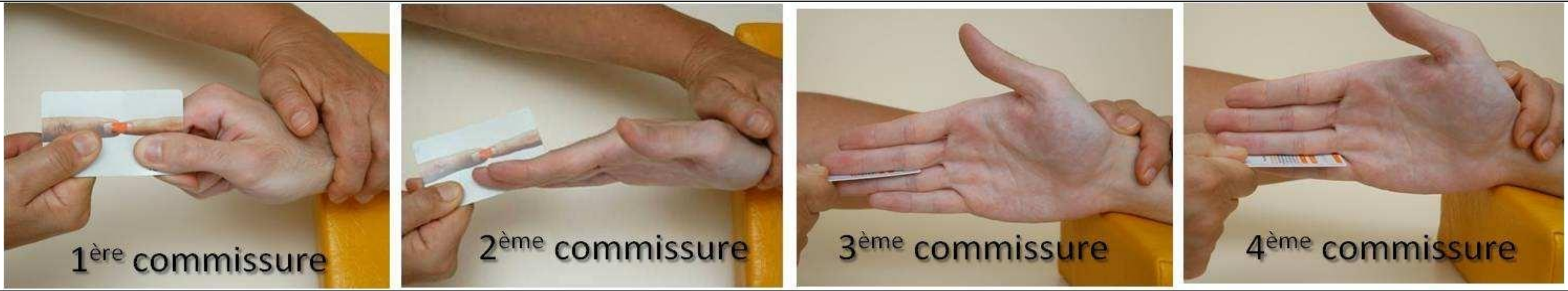

10: ”Try to grip this small cylinder by closing all your fingers around it and keeping it in the hollow of your hand (placed by the OT at the flexion crease of the finger MCPs). I will try to pull the stick out of your hand (working from the smallest to the largest cylinder and beginning with the healthy hand first).”

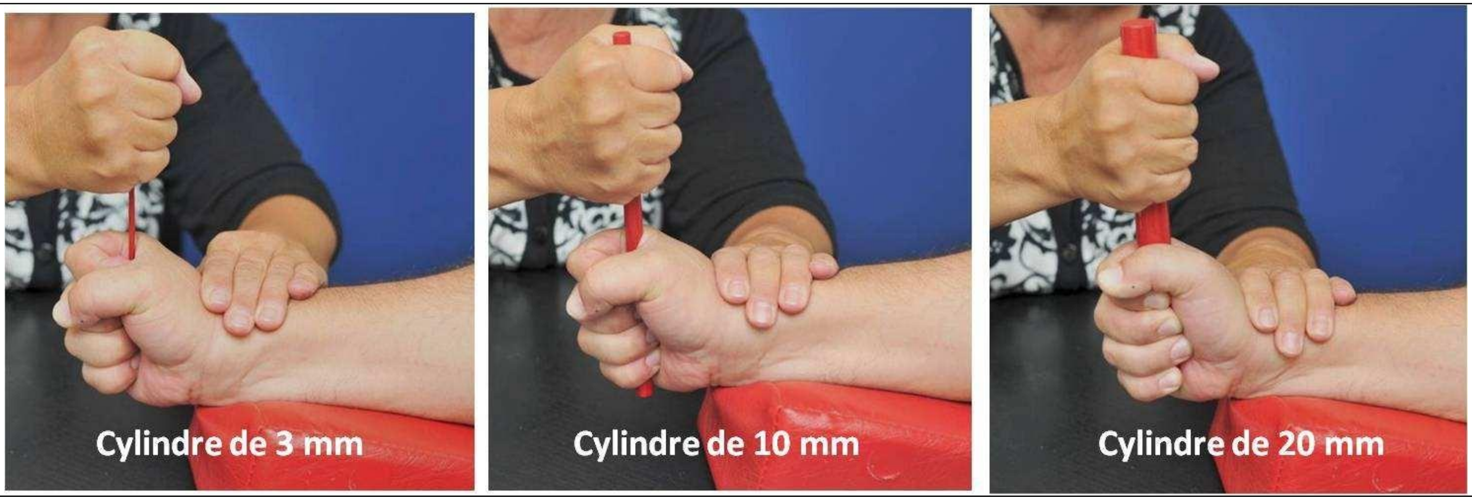

11: Clasp your hands together by interconnecting your fingers (in the direction of flexion), then raise your elbows as far as you can towards a horizontal line at chest height.”

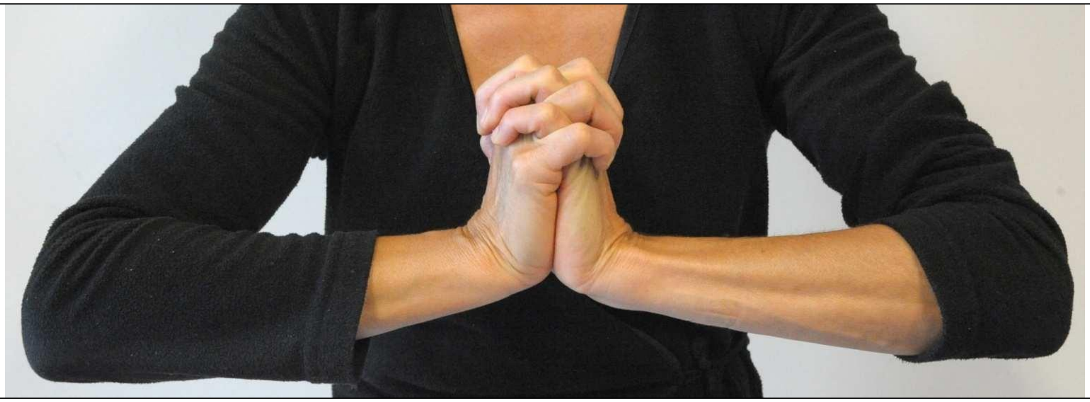

12: "Place your hands back to back, with your fingers extending downwards, then raise your elbows towards a horizontal line at chest height.”

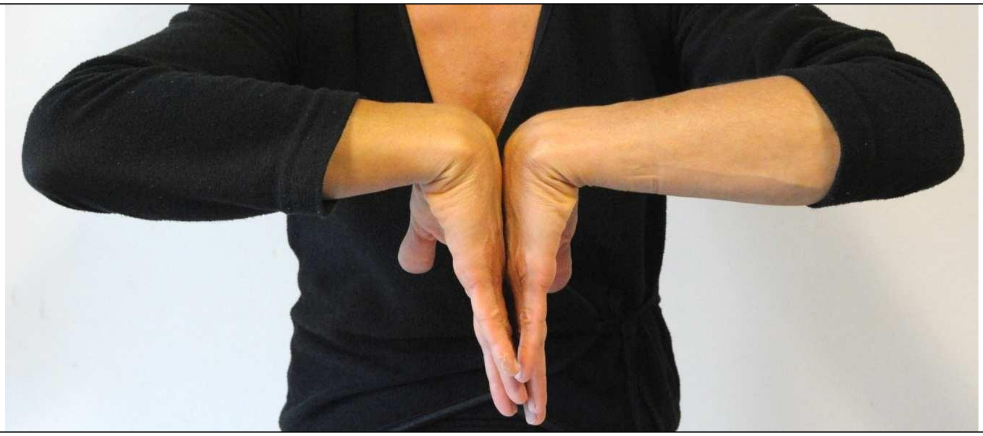

For exercises 13 and 14 the patient moves away from the table, sitting with their elbows tucked into the body, their forearms at a 90° angle and his thumbs pointing upwards.

13 and 14: “Move away from the table and tuck your elbows into your body. Hold your forearms at a right angle and point your thumbs to the sky. Turn your hands towards the ceiling then the floor, the ceiling then the floor, etc.”

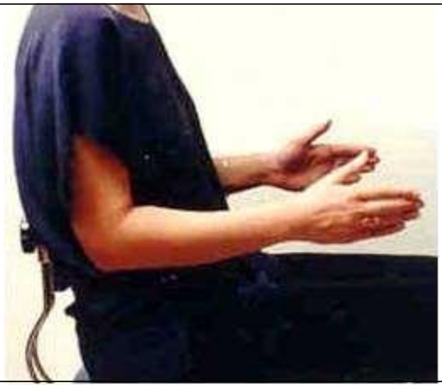

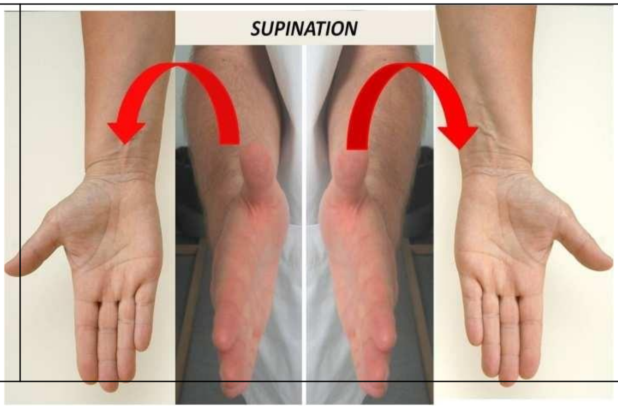

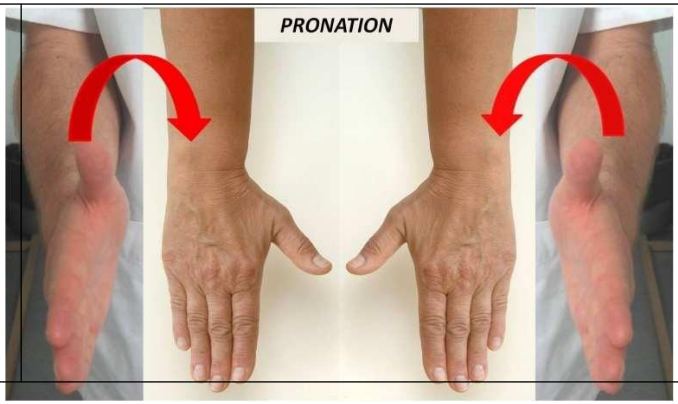

Grading

GRADING for exercises 1-7 and 11-14	COMPLETE MOVEMENT*, WITH BALANCE AND PRECISION	COMPLETE BUT SLOW MOVEMENT, WITH IMBALANCE OR LACK OF PRECISION	MOVEMENT INCOMPLETE, OR SHOWING EXCLUSION OR COMPENSATION	MOVEMENT IMPOSSIBLE**
	3	2	1	0

A "complete movement" is a movement performed to its full extent and with all the qualities required – balance, correct speed and precision – as a result of the physical integrity of the hand and wrist, and the patient's voluntary control.

*****A movement may be "impossible" because of a medical contraindication or a temporary or permanent incapacity.***

Specific grading for movements made against resistance:

For exercises 8 and 9

GRADING FOR EXERCISES 8 AND 9	GRIP MAINTAINED AGAINST STRONG RESISTANCE FROM OT	GRIP MAINTAINED AGAINST MEDIUM RESISTANCE FROM OT	GRIP MAINTAINED AGAINST LIGHT RESISTANCE FROM OT	GRIP NOT MAINTAINED
	3	2	1	0

For exercise 10

GRADING FOR EXERCISE 10	3mm STICK HELD AGAINST RESISTANCE APPLIED BY OT	10 mm STICK HELD AGAINST RESISTANCE APPLIED BY OT	20mm STICK HELD AGAINST RESISTANCE APPLIED BY OT	20mm STICK NOT HELD AGAINST RESISTANCE APPLIED BY OT
	3	2	1	0

Coefficients

Not all of these exercises have the same importance for hand function. Each is therefore attributed a coefficient that determines its position in the hierarchy of importance.
It is more important, for example, to be able to close one's hand completely than to fully extend the fingers; thus closing the hand has a coefficient of 3, but opening it has a coefficient of 2.

Table of coefficients

CLOSING THE HAND	3
OPENING THE HAND	2
SPREADING THE FINGERS	3
BRINGING THE FINGERS TOGETHER	2
ABDUCTION OF THE THUMB	3
ADDUCTION OF THE THUMB	2
OPPOSING THE THUMB TO THE FINGERS	3
OPPOSING THE THUMB TO THE FINGERS (WITH RESISTANCE)	2
LATERAL PINCH OF A CARD (WITH RESISTANCE)	2
OVERALL GRIP OF 3 STICKS AGAINST RESISTANCE	3
WRIST FLEXION	2
WRIST EXTENSION	3
PRONATION	3
SUPINATION	2

Calculation method

- Each exercise is graded from **0 to 3**.
- Each grade is **multiplied by its coefficient** to obtain a score.
- The test total is obtained by adding together the 14 scores, and **equates to the sum of the scores for the impaired hand**.
- Finally, this score is **divided by 105** (the maximum score) and then **multiplied by 100** to arrive at the **mobility percentage for the impaired hand compared to the mobility of a healthy hand.**

For example: Score of a healthy hand = maximum score = 105 points;
Score of the impaired hand = 69

Calculation:

$$\frac{69}{105} \times 100 = 65.7$$

NB:
If the final result of the test is not a round number:
round the figure down if the decimal is 5 or lower, but round the figure up if the decimal is higher than 5. **For example, 65.7 becomes 66/10**

Interpretation: The patient has 66% mobility in their impaired hand (compared to a healthy hand)

TEST 2: PREHENSION STRENGTH

Pretension strength is measured using two different measuring instruments. It is firstly measured on the healthy side, followed by the impaired side. Patients can familiarise themselves with the devices by trying them out with their healthy hand once or twice before starting the test.
This test concerns:
1. GRIP STRENGTH using a Jamar® dynamometer (the international standard) set to the second position. This tests the extrinsic and intrinsic muscles.
2. TIGHTENING STRENGTH of the lateral pinch between the surface of the thumb and the radial side of the index finger using a Jamar pinch gauge to test the thumb's extrinsic and intrinsic muscles.

Equipment

- Jamar dynamometer
- Jamar pinch gauge

Procedure

Starting position: the patient sits according to the recommendations of the American Society of Hand Therapists, with:

- a straight back and feet flat on the floor
- the shoulders in an unclenched position
- the arms resting loosely
- the elbows bent to a 90° angle
- the forearms in a neutral position
- wrist extension of 0 to 30° with an ulnar deviation of 0 to 15°.

The dynamometers are held by the occupational therapist. For each dynamometer, the score is obtained by finding the average of three successive readings, which are taken alternately on the healthy hand and then the impaired one, etc.

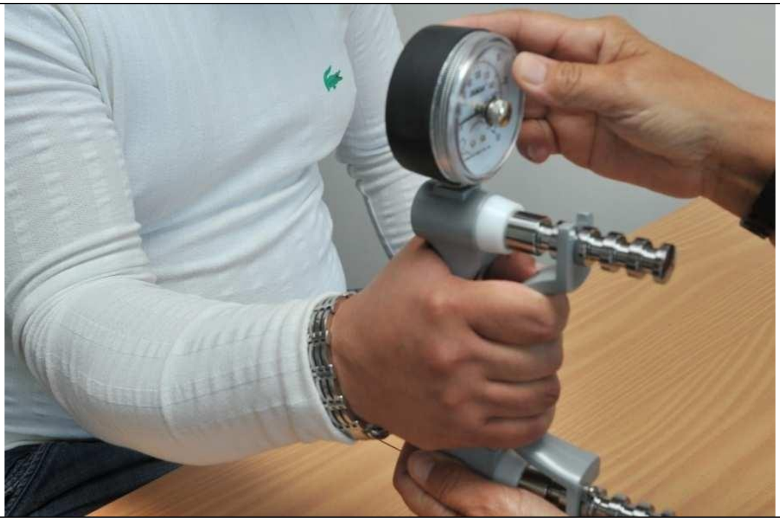

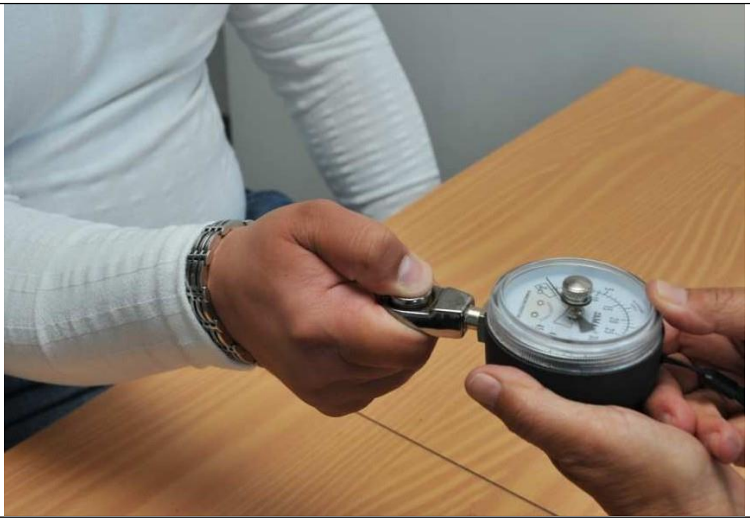

Calculation method

A healthy hand is conventionally considered to have maximum strength. This means that the reading it obtains is the maximum for both dynamometers, namely 10/10.
For each dynamometer, the impaired hand is graded by comparison with the healthy hand, and its score is obtained from a cross-multiplication.

°For example, for the Jamar dynamometer:

Healthy side = 48 kg; the score for this side is 10/10.
Impaired side = 18 kg; the score for this side is as follows:

$$\frac{\text{Impaired side reading}}{\text{Healthy side reading}} \times 10 = \frac{18}{48} \times 10 = 3.7 / 10$$

°The same calculations are applied to the pinch dynamometer:

Healthy side = 9.5 kg; the score for this side is 10/10.
Impaired side = 3.5 kg; the score for this side is as follows:

$$\frac{\text{Impaired side reading}}{\text{Healthy side reading}} \times 10 = \frac{3.5}{9.5} \times 10 = 1.2 / 10$$

Coefficients

Both dynamometers have a coefficient of 5. Therefore, in our example, the patient’s impaired hand obtains:

°for the Jamar dynamometer: 3.7 x 5, namely 18.5 / 50.
°for the pinch dynamometer: 1.2 x 5, which is 6 / 50.
The total for the impaired side is therefore: 18.5 + 6 = 24.5

The total is 100 for the healthy hand and 24.5 / 100 for the impaired one.

Weighting

In literature, studies measuring prehension strength attribute 8-12% more prehension strength to the dominant hand as a general rule.

We therefore adjust the result accordingly:

°**if the impaired hand is the dominant hand,** 10% is deducted from the score obtained
°**if the impaired hand is the non-dominant hand,** 10% is added to the score obtained

Thus in our example:

°Total scores for healthy non-dominant hand: 100 /100

°Total scores for impaired dominant hand: 24.5 /100

Since the impaired hand is the dominant hand, we need to deduct 10% from the result, so

we subtract 2.4 from 24.5. We therefore obtain: 24.5 – 2.4 = 22.1 or **22/100**

- ***Interpretation: We can state that this patient has a prehension strength of 22% compared to their healthy hand.***

- ***If both hands are affected, the score obtained is compared to the averages taken from a Swiss study conducted on a healthy cohort (496 men and 482 women aged 18 to 85 years and over) in 2009:***

**JAMAR STRENGTH IN KG**					
**JAMAR MEN (496)**			**JAMAR WOMEN (482)**		
Age	Dominant hand	Non-dominant hand	Age	Dominant hand	Non-dominant hand
18-19 (33)	5.2	48.3	18-19 (31)	32	30.7
20-29 (59)	53.4	50.8	20-29 (61)	33.8	32.5
30-39 (69)	55.4	53	30-39 (72)	34.8	33.6
40-49 (68)	53	56.7	40-49 (79)	34	34.1
50-59 (70)	52.2	55	50-59 (62)	32.8	32.6
60-69 (79)	45.4	44.9	60-69 (64)	29.1	28
70- 79 (61)	39.2	38.7	70-79 (53)	25.7	24.8
80-85 (29)	30.7	29.4	80-85 (32)	19.2	19.7
> 85 (28)	22.4	23.2	> 85 (28)	16.9	16.7

**PINCH STRENGTH IN KG**					
**PINCH MEN (496)**			**PINCH WOMEN (482)**		
Age	Dominant hand	Non-dominant hand	Age	Dominant hand	Non-dominant hand
18-19 (33)	9.5	9.1	18-19 (31)	6.9	6.5
20-29 (59)	9.9	9.3	20-29 (61)	6.6	6.4
30-39 (69)	10.1	9.7	30-39 (72)	7	6.7
40-49 (68)	10	9.6	40-49 (79)	7.1	6.8
50-59 (70)	10	9.6	50-59 (62)	6.8	6.6
60-69 (79)	9.2	8.8	60-69 (64)	6.5	6.2
70-79 (61)	8.2	7.8	70-79 (53)	5.4	5
80-85 (29)	6.4	6.5	80-85 (32)	4.3	3.9
> 85 (28)	5.4	5.5	> 85 (28)	3.1	2.8

TEST 3: ONE-HANDED GRASP AND OBJECT TRANSFER

This tests the patient's ability to:

- pick up 20 objects of varying sizes, weights and shapes placed on a reference surface in a specific order;
- transfer them to another surface 50cm above the first, placing them in their corresponding locations.
- The test begins with the healthy hand and continues with the impaired one. The test is timed, so the patient gives more attention to the speed of execution than to the method.

As a result, the patient's movements are more natural and spontaneous, and the occupational therapist can easily observe prehension deficits such as exclusion, control dysfunction, compensation, etc.

Equipment

1-One cube with sides measuring 10cm (700g)
2-One cube with sides measuring 7.5cm (300g)
3-One cube with sides measuring 5cm (100g)
4-One cube with sides measuring 2.5cm (10g)
5-One cylinder 12cm high with a diameter of 10cm (700g)
6-One cylinder 11cm high with a diameter of 7.5cm (300g)
7-One cylinder 10cm high with a diameter of 5cm (100g)
8-One marble with a diameter of 25mm
9-One marble with a diameter of 16mm
10-One tennis ball
11-One pin 4cm in length with a diameter of 2mm
12-One electronic lighter
13-One coin with a diameter of 15mm
14-One coin with a diameter of 25mm
15-One coin with a diameter of 30mm
16-One house key
17-Its corresponding lock
18-One iron weighing 2kg
19-One jug with two pouring spouts and 500ml water
20-One ordinary glass

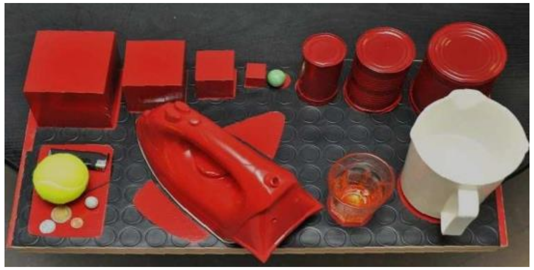

- One reference surface is installed as part of a pair of surfaces measuring 30cm x 60cm, with one placed 50cm above the other. They are placed over a height-adjustable table or against a wall with a mechanism to adjust their height.
The table is adjusted so that the upper surface is positioned at shoulder height to the patient.

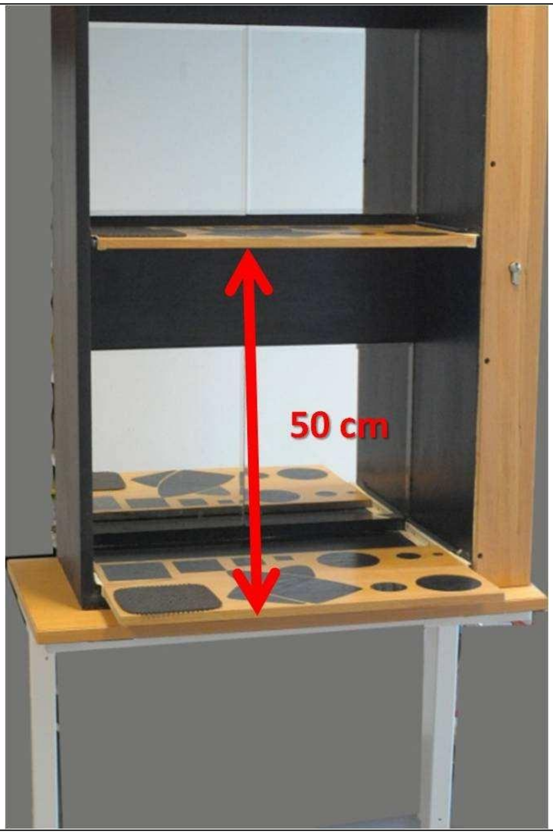

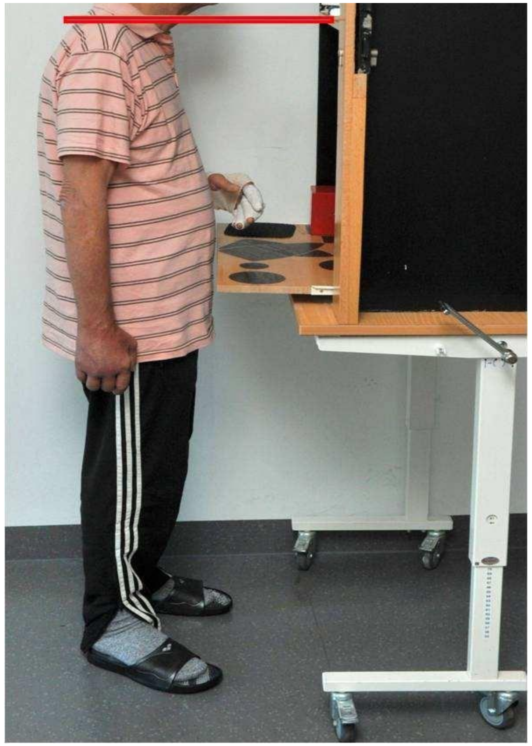

A mirror affixed to the back of each shelf allows the hand to be observed more easily.

Procedure

Please note:

Three objects require an action to be performed before they are moved to the top surface:

- the electronic lighter should be lit using pressure from the thumb
- the key should be inserted into the lock, which is then locked and unlocked before the patient removes the key and places it on the top surface
- part of the 500ml water in the jug is poured into a glass, with the first half of the glass poured in a pronated position and the second half in a supinated position. The water is then poured back into the jug before the patient carries both jug and glass to the top surface.

Before the test begins, the occupational therapist will show the patient how everything is done, explaining that the test is timed and so should be completed as quickly as possible:

"Pick up the objects one by one, starting from the left and moving to the right. Start with the row at the back and repeat for the row in front, again working from left to right."
"Place the objects in their corresponding spaces, and remember to light the lighter, to turn the key in the lock and to pour the water from the jug into the glass like this (pronated position) and then like this (supinated position)."
"The exercise is timed, so work as fast as you can."
"Are you ready? Then please begin!"

Below are examples of each grasp action, showing both the radial and ulnar view for each object, as well as the actions required for some of the objects. The shots were taken in the correct order of the grasp actions, and with the proper adjustment of the two reference surfaces.

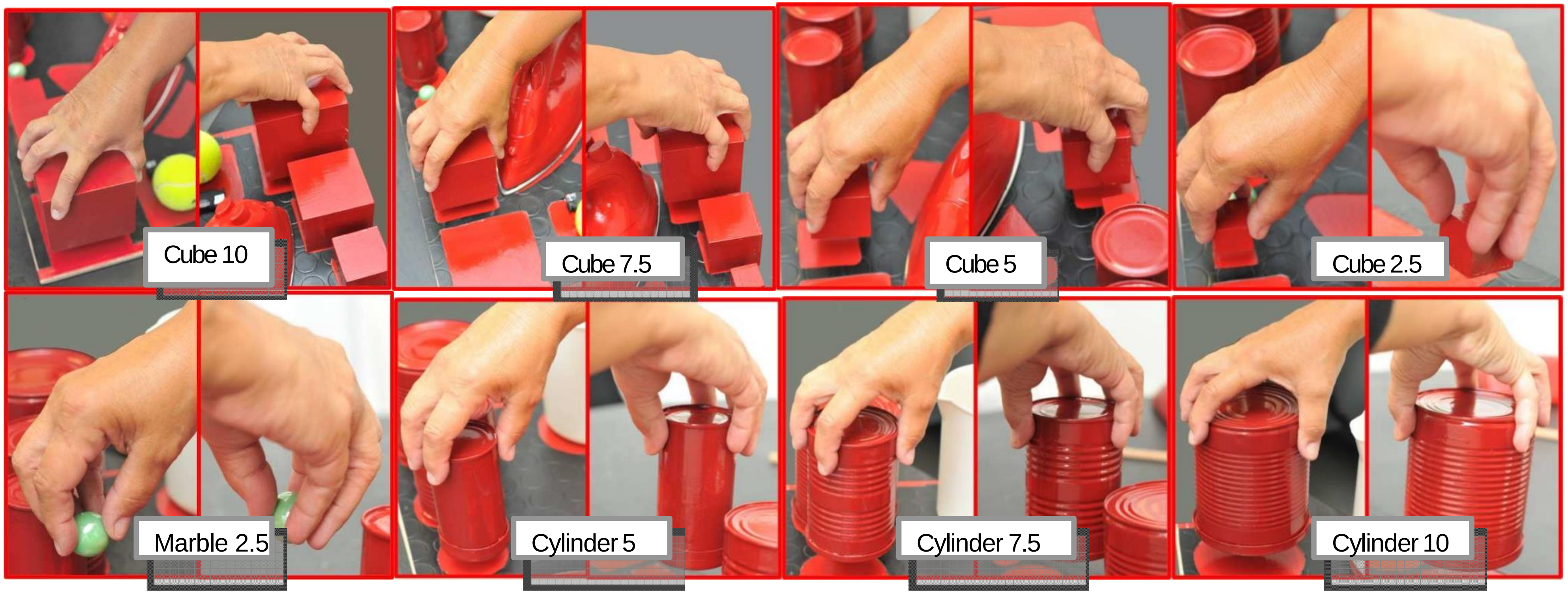

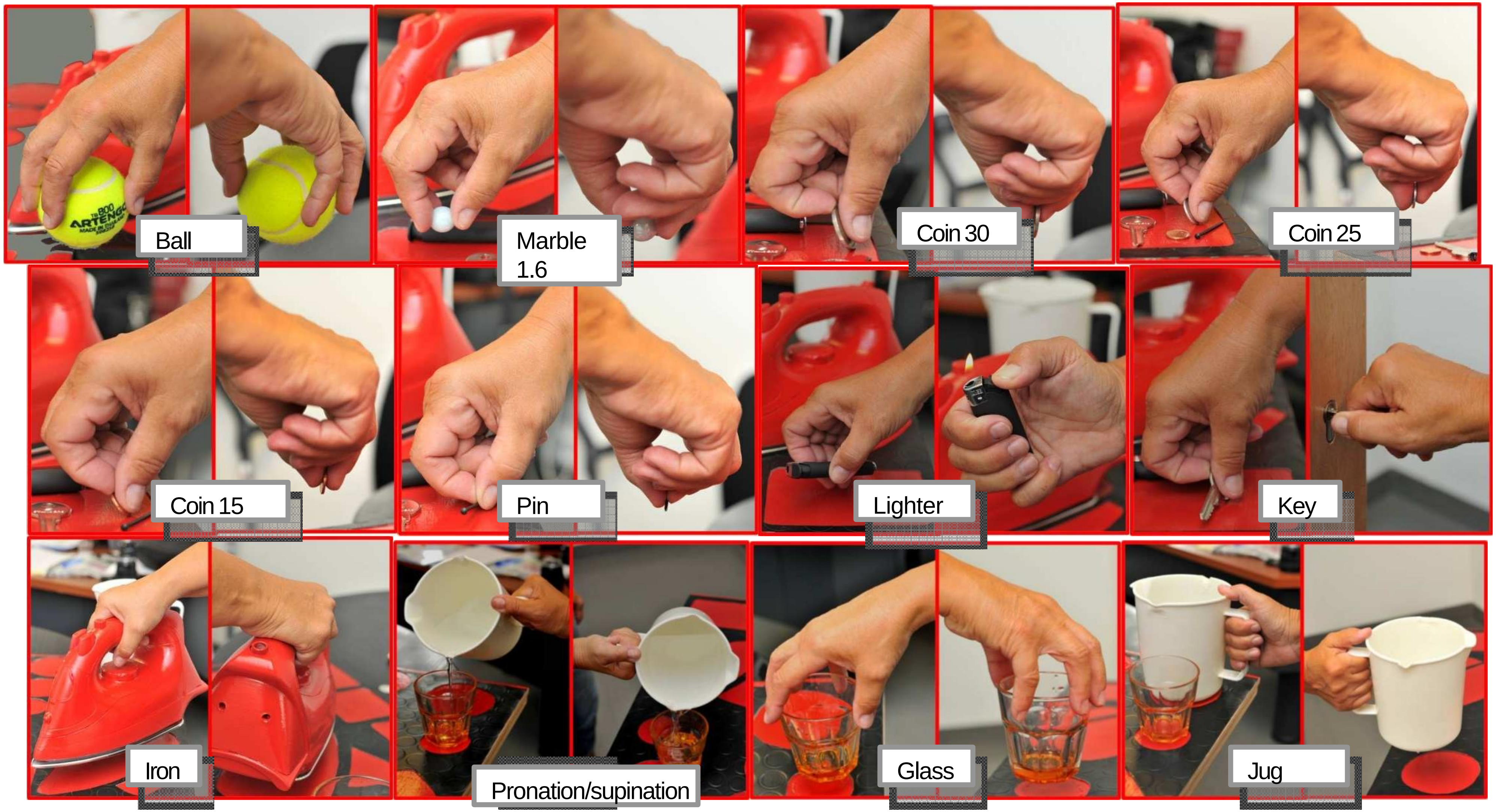
Ball
Marble 1.6
Coin 30
Coin 25
Coin 15
Pin
Lighter
Key
Iron
Pronation/supination
Glass
Jug

Grading

Grading the activity requires grasp and movement to be observed. The double-entry table allows these to be graded quickly following successful observation.

GRADING	GRASP CORRECT, PRECISE AND BALANCED	GRASP CORRECT BUT SLOW OR IMPRECISE OR LACKING BALANCE	GRASP WITH EXCLUSION OR COMPENSATION	GRASP IMPOSSIBLE
FAST, PRECISE TRANSFER	3	2	1	0
SLOW OR IMPRECISE TRANSFER	2	2	1	0
TRANSFER WITH COMPENSATION	1	1	1	0
TRANSFER NOT ACHIEVED	0	0	0	0

Thus it is sufficient for the grasp alone or the transfer alone to be wrong for the lowest mark to be awarded for the exercise. Where patients have permanent impairments caused by the amputation of a finger or arthrodesis, for example, but are able to make optimal use their remaining capacities and carry out the exercises almost normally, unavoidable compensation is permitted.

Please note:

The average time needed for this test is 1 minute or less for a healthy hand. If more than this amount of time is taken, the occupational therapist does not change the score, but can note it as extra information relevant to their patient's rehabilitation.

Calculation method

Each exercise is graded from 0 to 3.
The test total is obtained by adding together the 20 different scores: this total equates to the total points for the impaired side. This figure is divided by 60 (the maximum score) and then multiplied by 100 to obtain the **one-handed percentage capacity of the patient's impaired hand compared to a healthy hand.**

Example:

- The total score for the healthy side is equal to the maximum score, namely 60 points.
- Here, the total score for the impaired side equals 46 points.

A cross-calculation is then carried out by dividing the total score for the impaired hand by the maximum score, and multiplying this by 100:

$$\frac{46}{60} \times 100 = 76.6\%$$

NB:

When calculating the final result, round the figure down when the decimal is 5 or lower, and round the figure up when the decimal is higher than 5.

Thus 76.6 becomes **77/100.**

Interpretation: We can state that this patient has a one-handed percentage capacity of 77% for his impaired hand compared to his previous ability.

TEST 4: TWO-HANDED FUNCTION

This test evaluates the patient's two-handed function by means of 20 tasks drawn from everyday life, and verifies whether hand dominance is upheld. The test is carried out at the patient's pace, without particular instructions or timing of the exercise. However, the test time should not exceed 15 minutes.

Equipment

1. One plate
2. Some modelling paste
3. Everyday cutlery
4. Jam jar (with a screw lid and filled with particles)
5. Bottle of water (50ml) with a screw cap
6. Bolt and 4mm nut
7. Medicine tube with push-down cap
8. Shirt on a board, with 3 buttons 12mm in diameter
9. Board with 3 laces
10. Needle with large eye and thread
11. Large box of matches
12. Purse (with press stud and zip)
13. A4 sheet of paper
14. Bic-style ballpoint pen
15. Section of cube ruler (22cm in length)
16. Some card (1mm thick and 20cm long)
17. Office scissors
18. Copper wire (1mm)
19. Pliers to cut the end
20. Sheets of newspaper (4 double sheets)

Procedure

The occupational therapist gives the 20 tasks to carry out in succession, ensuring they place the items needed for each exercise on the table in front of the patient. The OT then tells the patient to act as they usually would, giving no further instructions other than those provided with the assessment equipment:

"Carry out the tasks as naturally as possible, just as you can and would if you were at home on your own without anybody else to help you do them."

1 - "Using the cutlery, cut three small pieces of paste on your plate as if you were cutting steak."

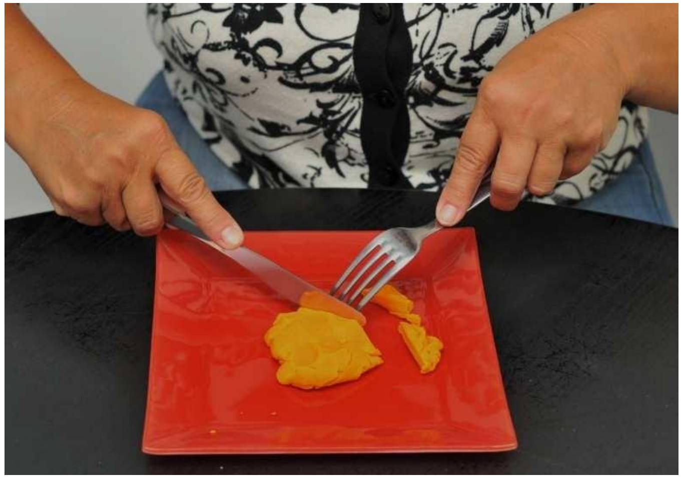

2 - "Open this 500ml bottle of water, then close it again. The bottle has a screw cap."

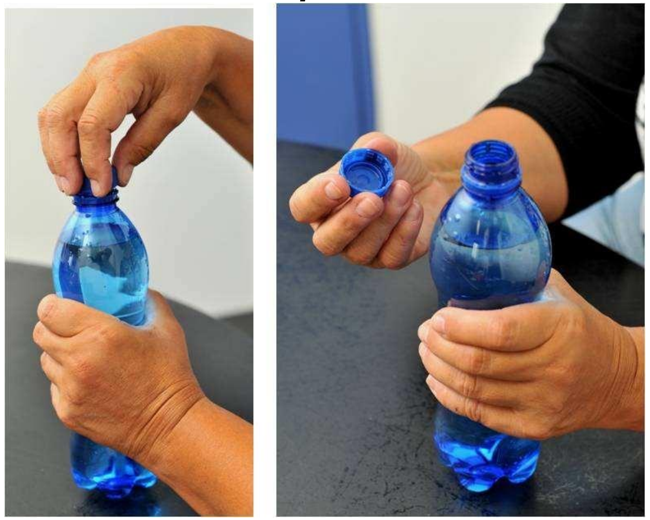

3 - "Open this jam jar and close it again" *(the lid is screwed on as tightly as possible in advance).*

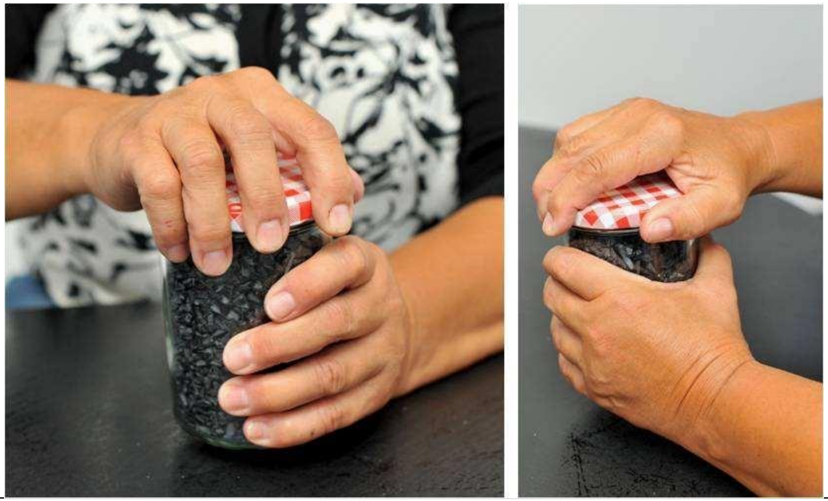

4 - "Open this tube, then replace the cap. Note that the cap is not screwed on, but pushed on."

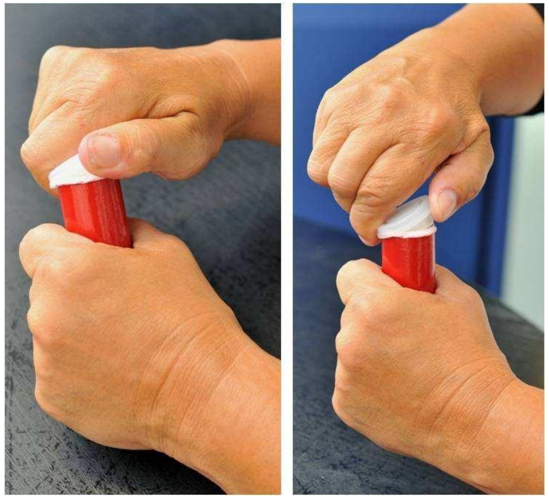

5 - "Undo the three buttons on this shirt.”
6 - “Do the buttons up again.”

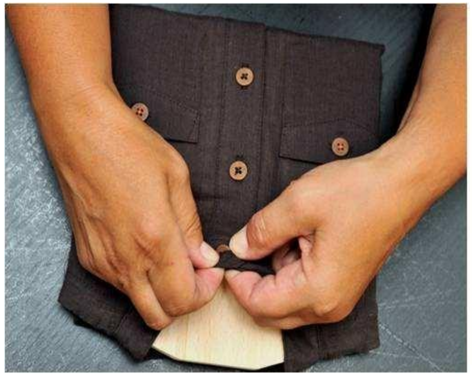
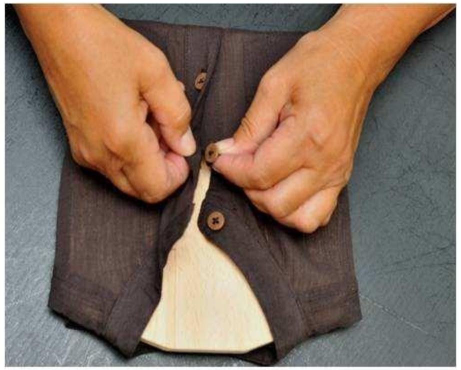

7 - "Untie these three laces.”
8 - “Tie the laces again.”

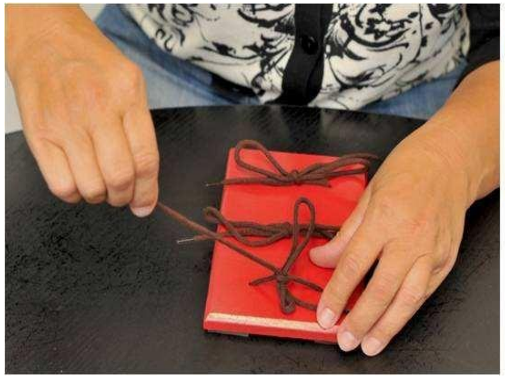
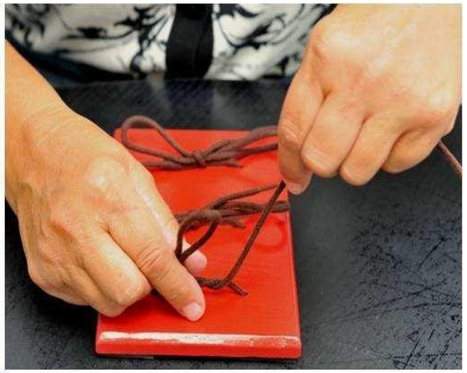

9 - "Unscrew this nut from the bolt, remove it and then screw it on again at least half way.”

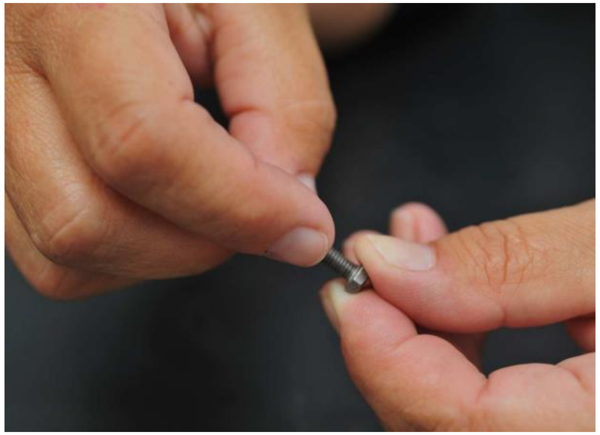

10 - “Thread this needle.”

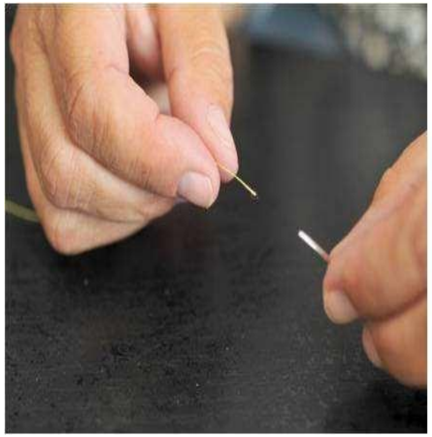
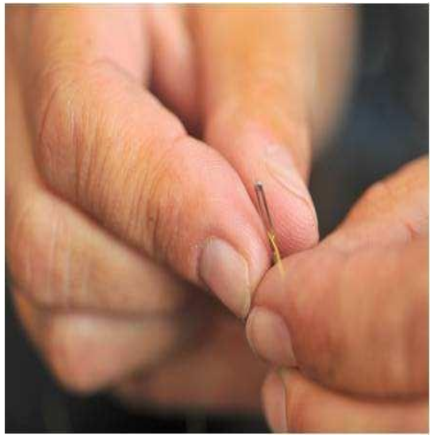

11 - “Open this matchbox, take out a match and light it.”

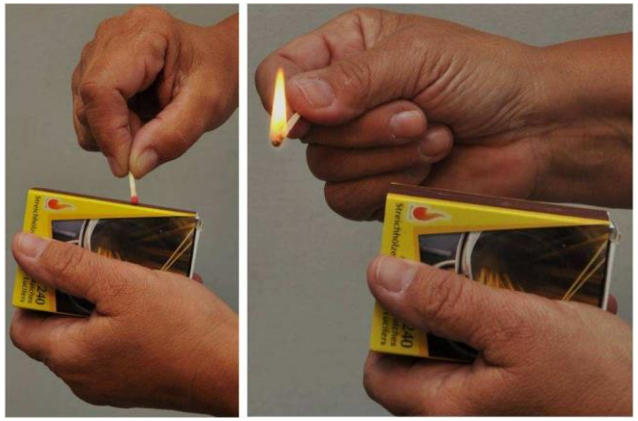

12 - “Extinguish the match by shaking it, not by blowing it out.”

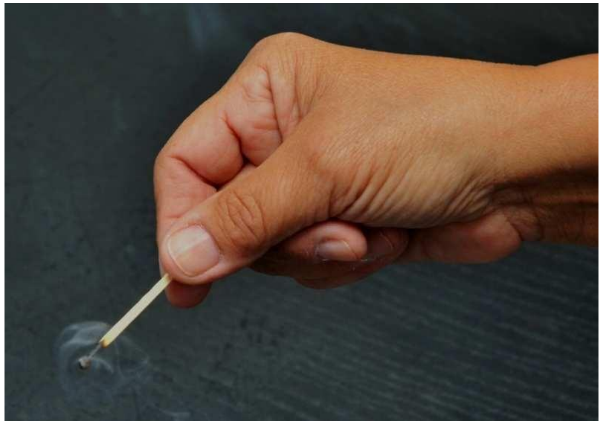

13 - “Open the zip on this purse, replace the 5 coins inside, and close the purse again. Open the purse on the clip side, take out the note, unfold it, fold it again and put it back inside.”

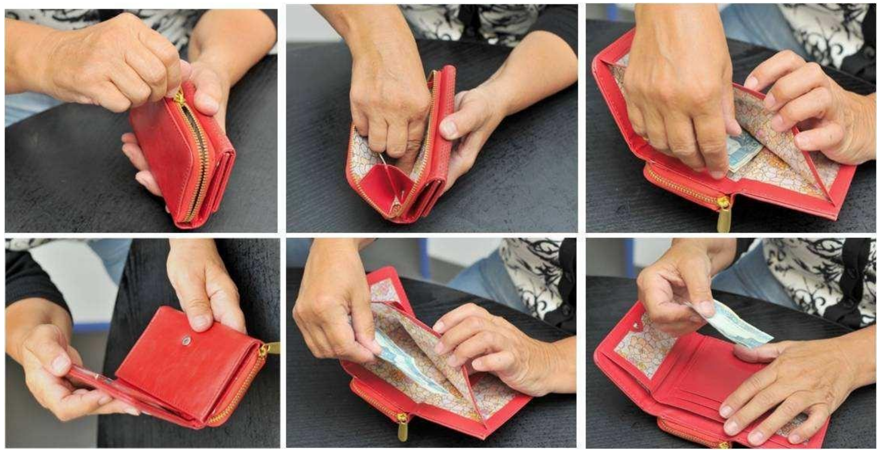

14 - “Write this sentence: *‘the hand is one of the greatest assets that men and women have’.”*

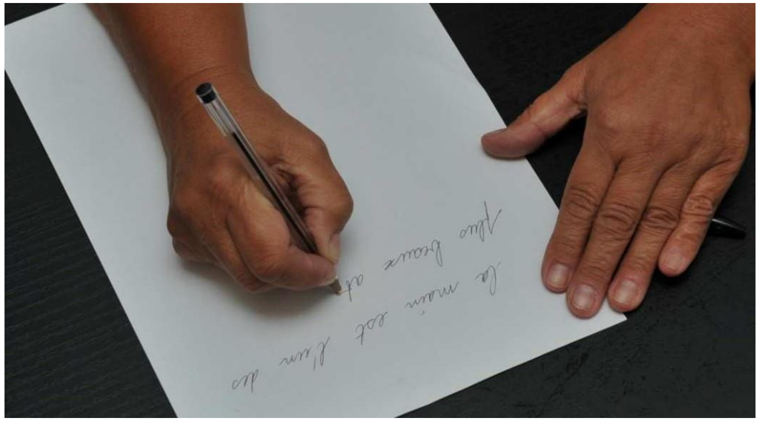

15 - “Draw a line using this ruler.”

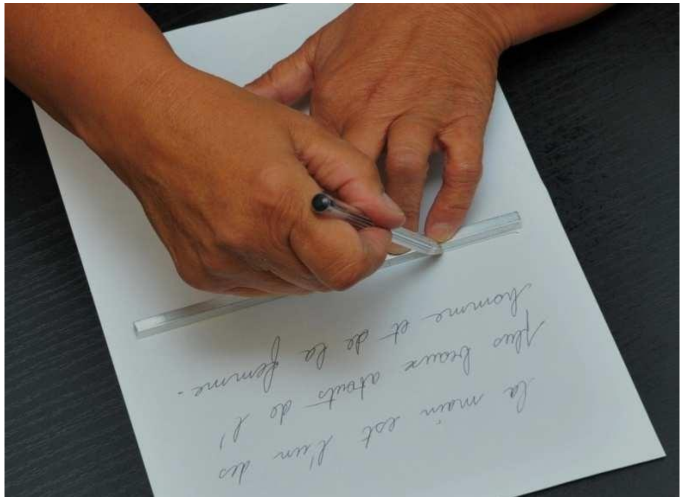

16 - “Fold the paper along the line you have just drawn.”

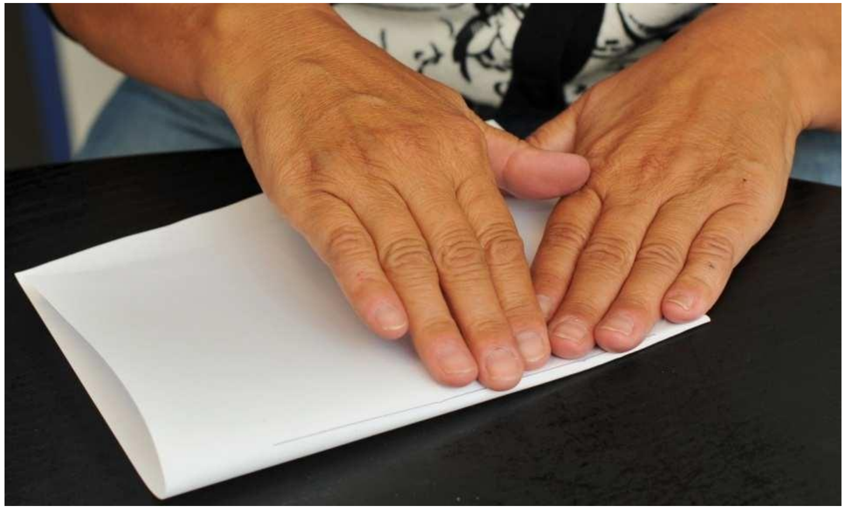

17 - “Tear the paper along the folded line.”

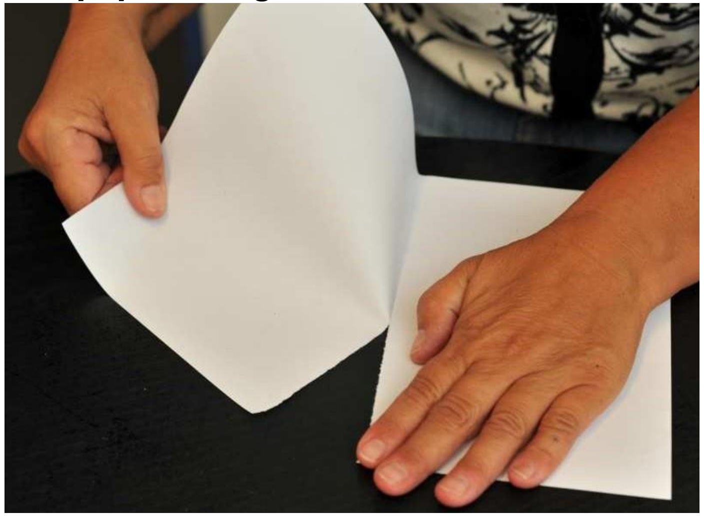

18 - “Cut a strip of card using these scissors.”

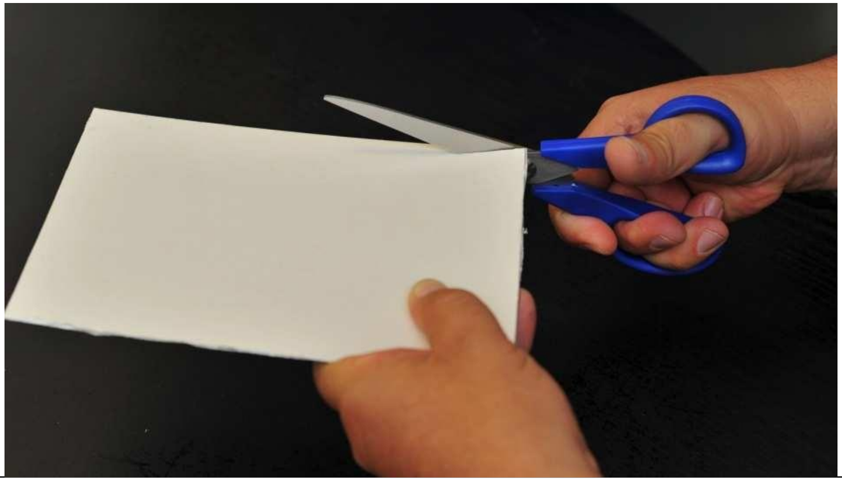

19 - *“Using these pliers, cut three small pieces of metal.”*	20 - *“Try to tear this newspaper down the middle, starting from the side shown.”* (Use the side of the join, with 4 double sheets folded 3 times to make a 32-ply newspaper)
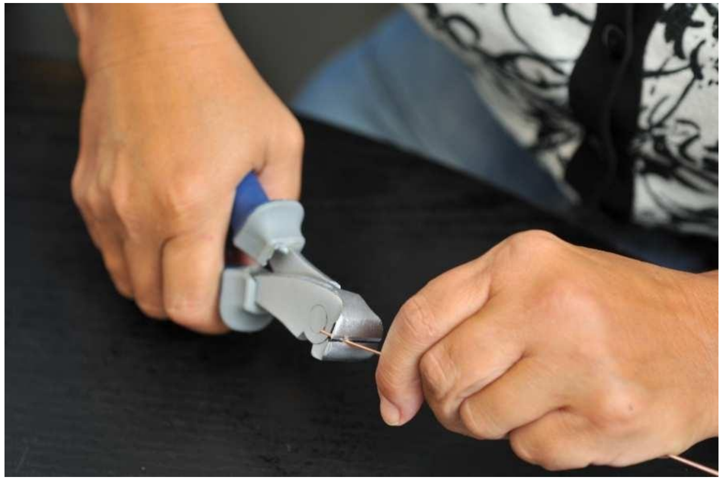	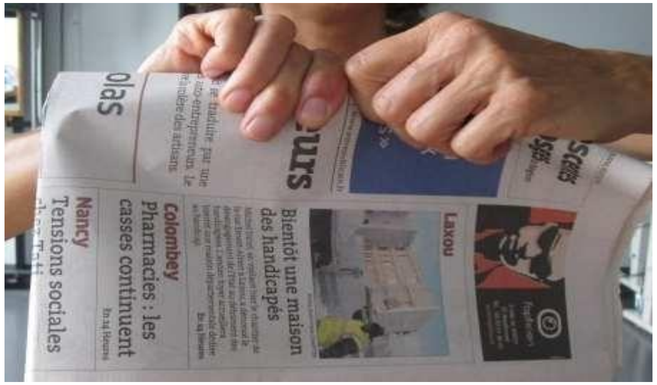

Score

TEST4 GRADING	NORMAL MOVEMENT*	MOVEMENT COMPLETE BUT SLOW OR IMPRECISE	MOVEMENT IINCOMPLETE OR WITH EXCLUSION OR COMPENSATION	MOVEMENT IMPOSSIBLE
	3	2	1	0

NORMAL MOVEMENT*: in cases of amputation or arthrodesis, unavoidable compensation is permitted and a “normal movement” grade may be awarded.

This test also allows any potential **transfer of dominance** to be observed, especially if the dominant hand is impaired.

For example, a right-handed patient who uses their left hand to open a jar but is able to use their right hand to hold it will be awarded a “3” annotated with the **“-”** sign.
This sign signifies “correct” but with a transfer of dominance.
If the patient’s hand dominance is respected, they are graded “3” annotated with the **“+”** sign.

These observations relating to dominance have no influence whatsoever on the scores awarded; they are simply additional indications that can be used as a guide for any subsequent rehabilitation.

Calculation method

-Each exercise is graded from 0 to 3.

-The total of the 20 scores is obtained by adding them together.

-This result is then divided by 60 (the maximum score) and multiplied by 100 to obtain the patient's two-handed percentage use capacity compared to a person with two healthy hands.

For example:

- Total marks obtained by the patient = 47 points
- Maximum score = 60 points

Calculation: $$\frac{47}{60} \times 100 = 78.3 / 100$$

NB:

When calculating the final result, round the number down if the decimal is 5 or lower, and round it up if the decimal is higher than 5.

- 78.3 therefore becomes **78/100.**

Interpretation: We can state that this patient has a two-handed function of 78% compared to a person whose function is unimpaired.

OVERALL ASSESSMENT CALCULATION METHOD

The overall score is obtained by calculating the sum of the results obtained in all four tests.

For example:

- Hand mobility test = 77
- Prehension strength test = 52
- One-handed test = 77
- Two-handed test = 78

Calculation: 77 + 52 + 77 + 78 = **284 points / 400 points (maximum possible score)**

We can also divide this result by 4, which gives a figure out of 100 points (or a percentage) that is easier to retain:
We have 284 points, therefore 284/4 = **71%**

These results can be illustrated in the form of a bar chart to make them easier and simple to read, both for patients and the various members of the medical and auxiliary staff.

Interpretation: We can state that the functional use capacity of the patient's impaired hand is 71% of that of a healthy hand, or that it is reduced by 29% compared to a healthy hand.

The 400-Point Assessment illustrates patient development in a concrete manner, emphasising progress made or, conversely, underlining a need to continue the work.
Beyond a general evaluation, it also highlights particularly significant deficits so that the rehabilitation programme can be adjusted and optimised, or the patient can be referred for surgery.

EQUIPMENT REQUIRED

Test 1

- A triangular cushion is used for the three exercises involving a resistance.
- A playing card or bank card
- Three cylinders 22cm in length:
    - one with a diameter of 3mm
    - one with a diameter of 10mm
    - one with a diameter of 20mm

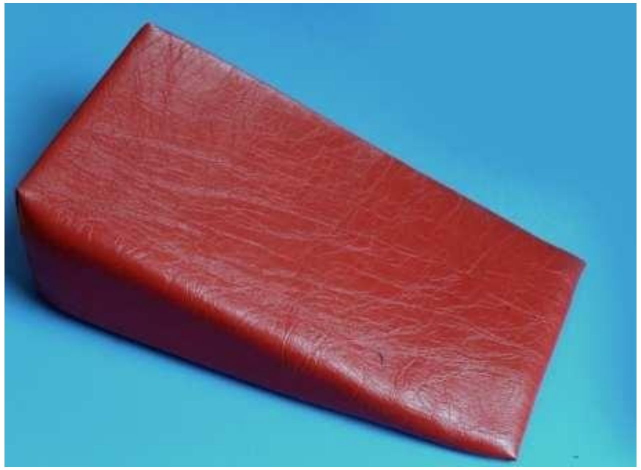

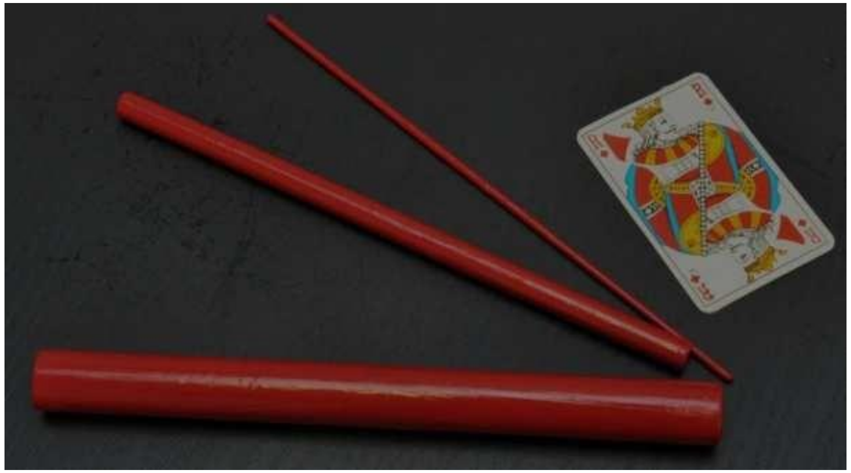

Test 2

- A Jamar dynamometer
- A Jamar pinch dynamometer

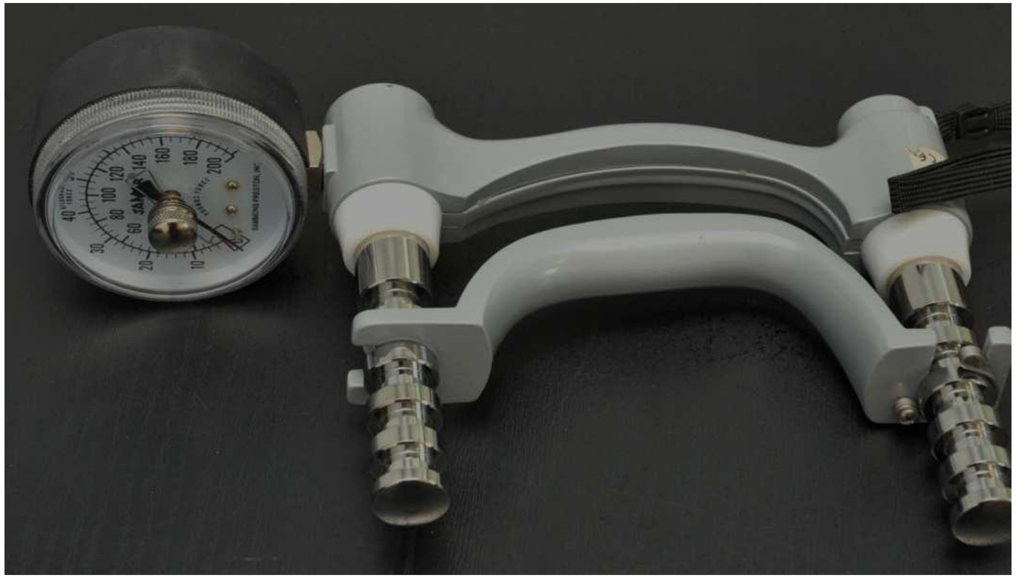

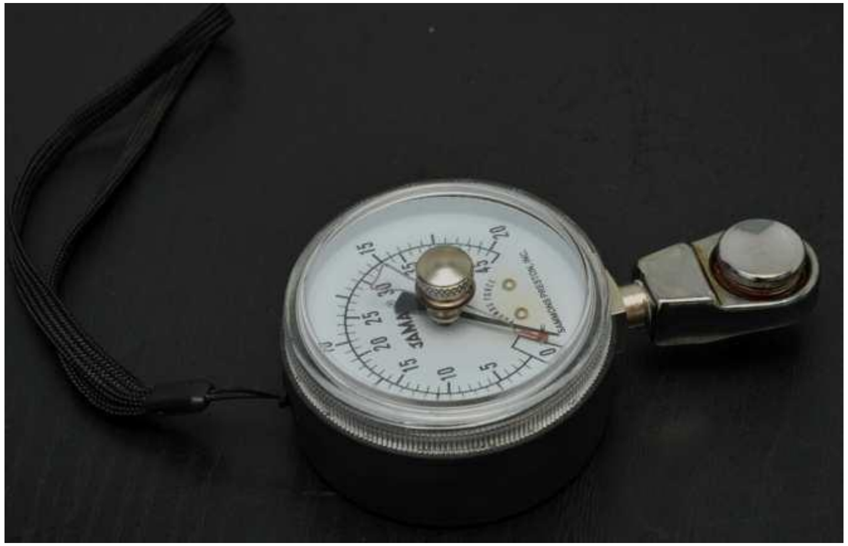

Test 3

A stopwatch

One cube with sides measuring 10cm (700g)
One cube with sides measuring 7.5cm (300g)
One cube with sides measuring 5cm (100g)
One cube with sides measuring 2.5cm (10g)

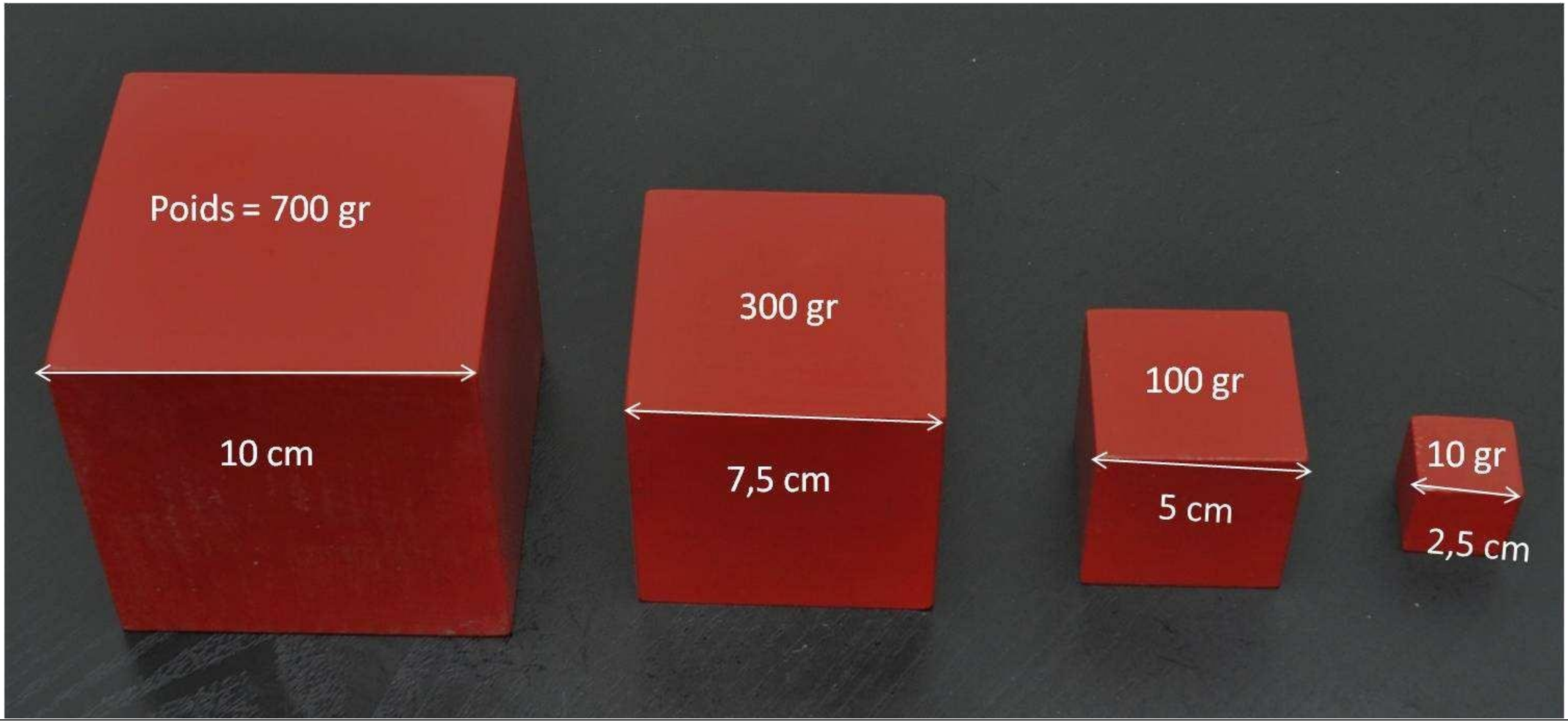

One cylinder 12cm in height with a diameter of 10cm (700g)
One cylinder 11cm in height with a diameter of 7.5cm (300g)
One cylinder 10cm in height with a diameter of 5cm (100g)
One marble with a diameter of 25mm

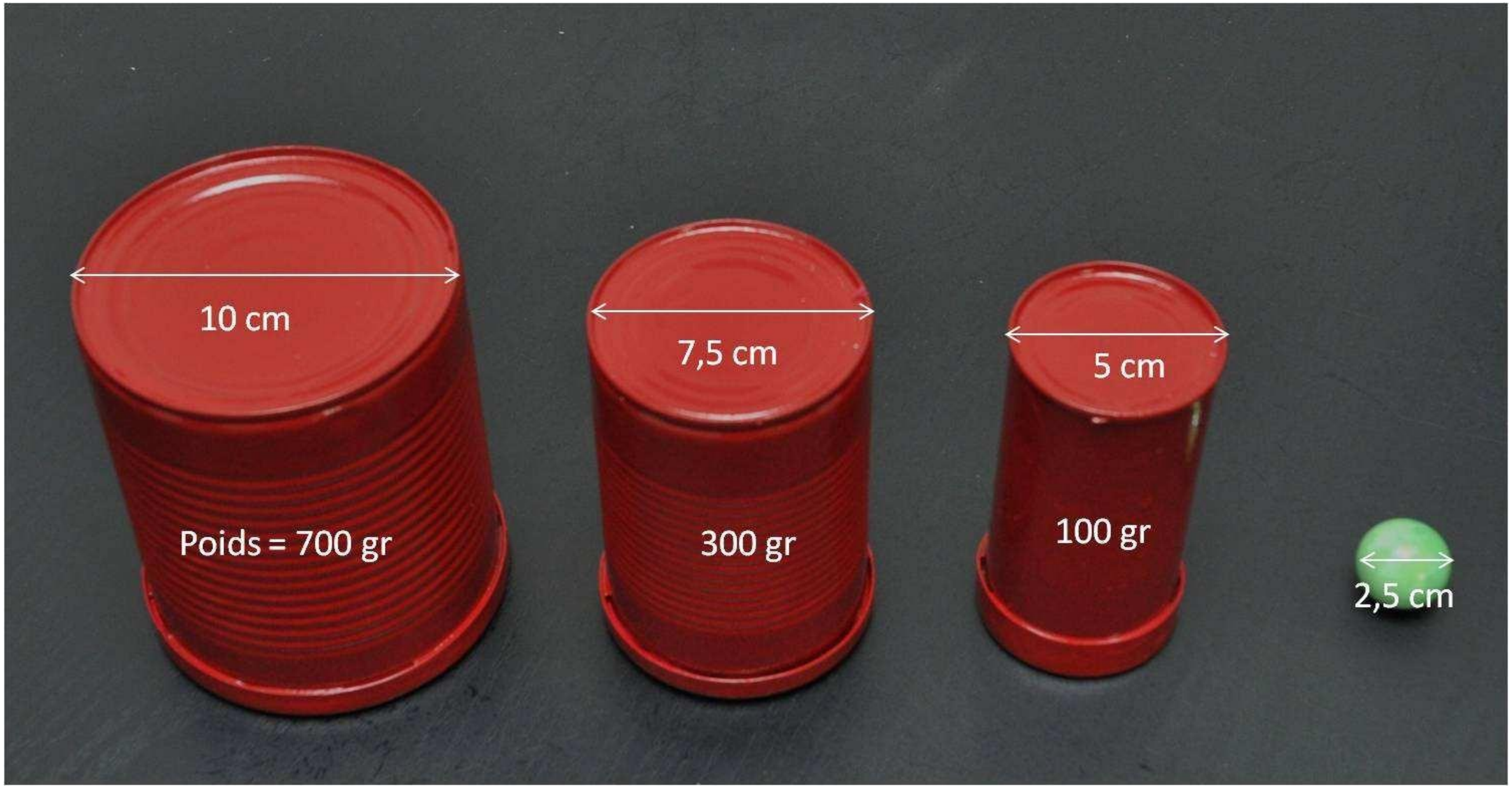

One tennis ball
One coin with a diameter of 30mm
One coin with a diameter of 25mm
One coin with a diameter of 15mm
One marble with a diameter of 16mm
One pin measuring 4cm with a diameter of 2mm
One electronic lighter

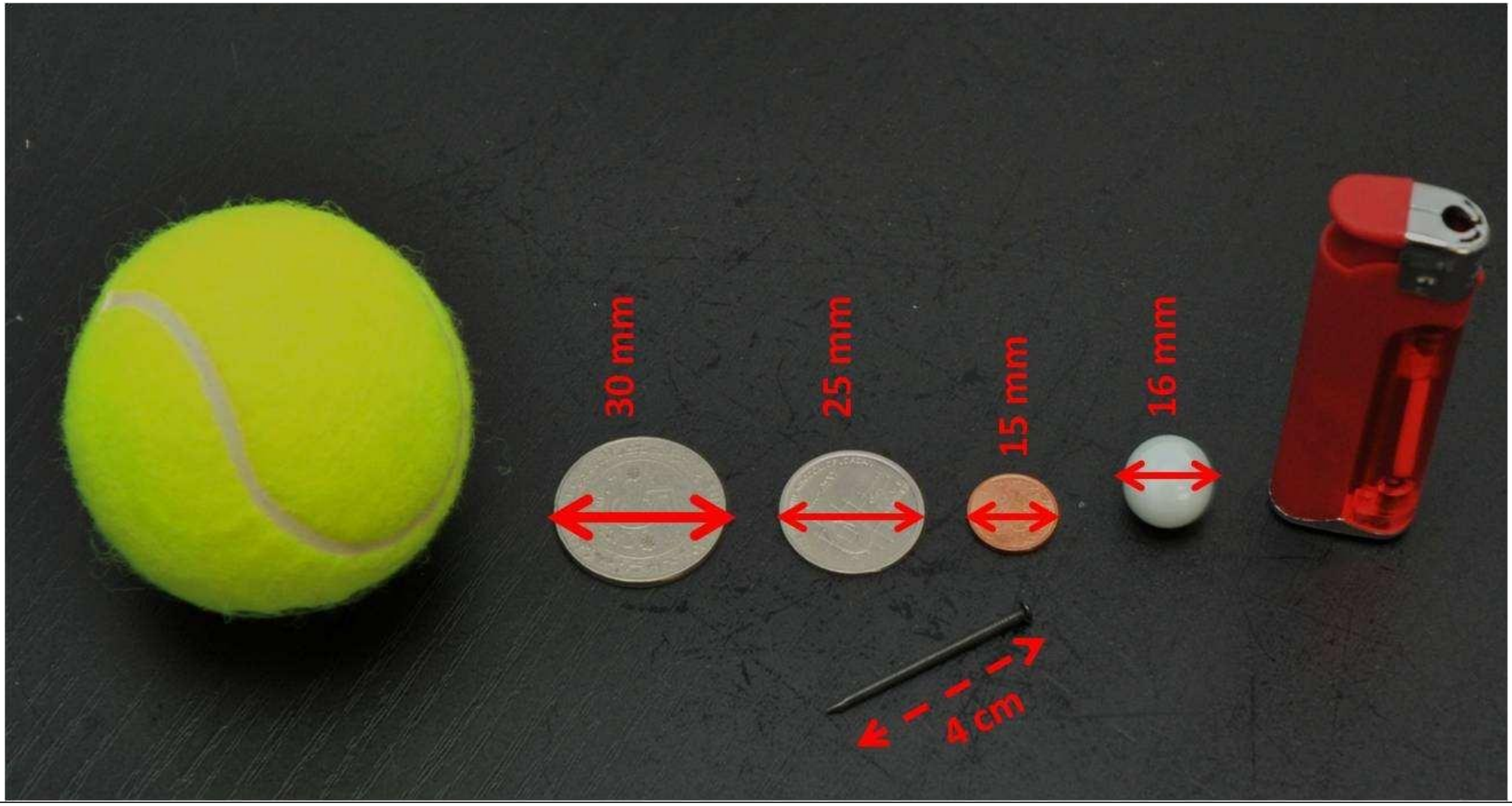

One house key with its corresponding lock and one iron weighing 2kg

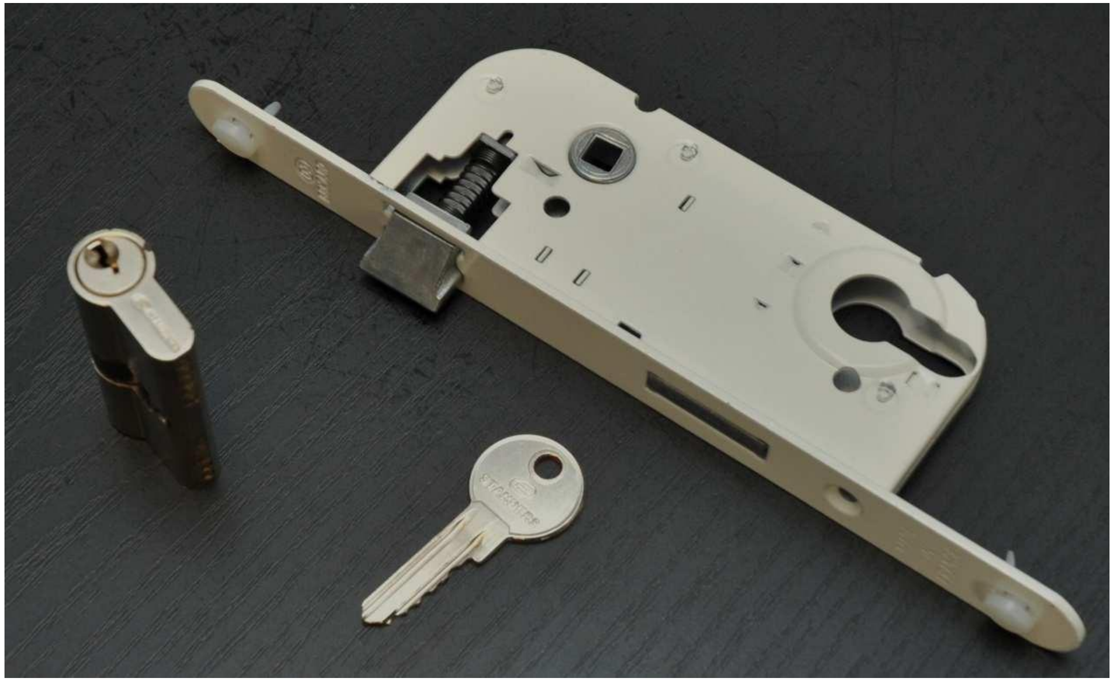	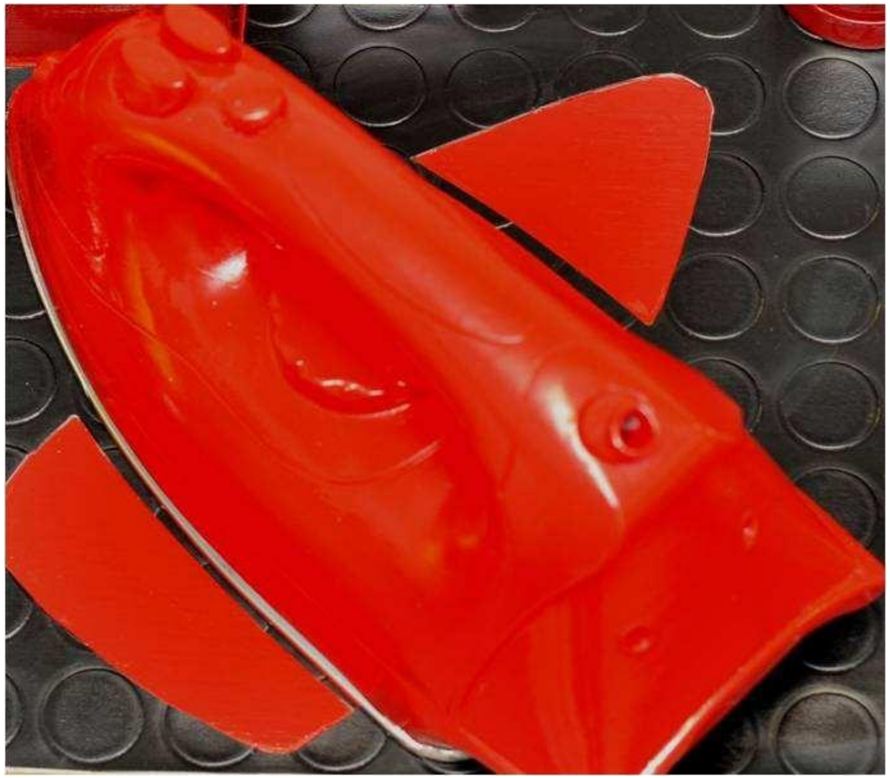

One jug with two pouring spouts and 500ml water
One ordinary glass

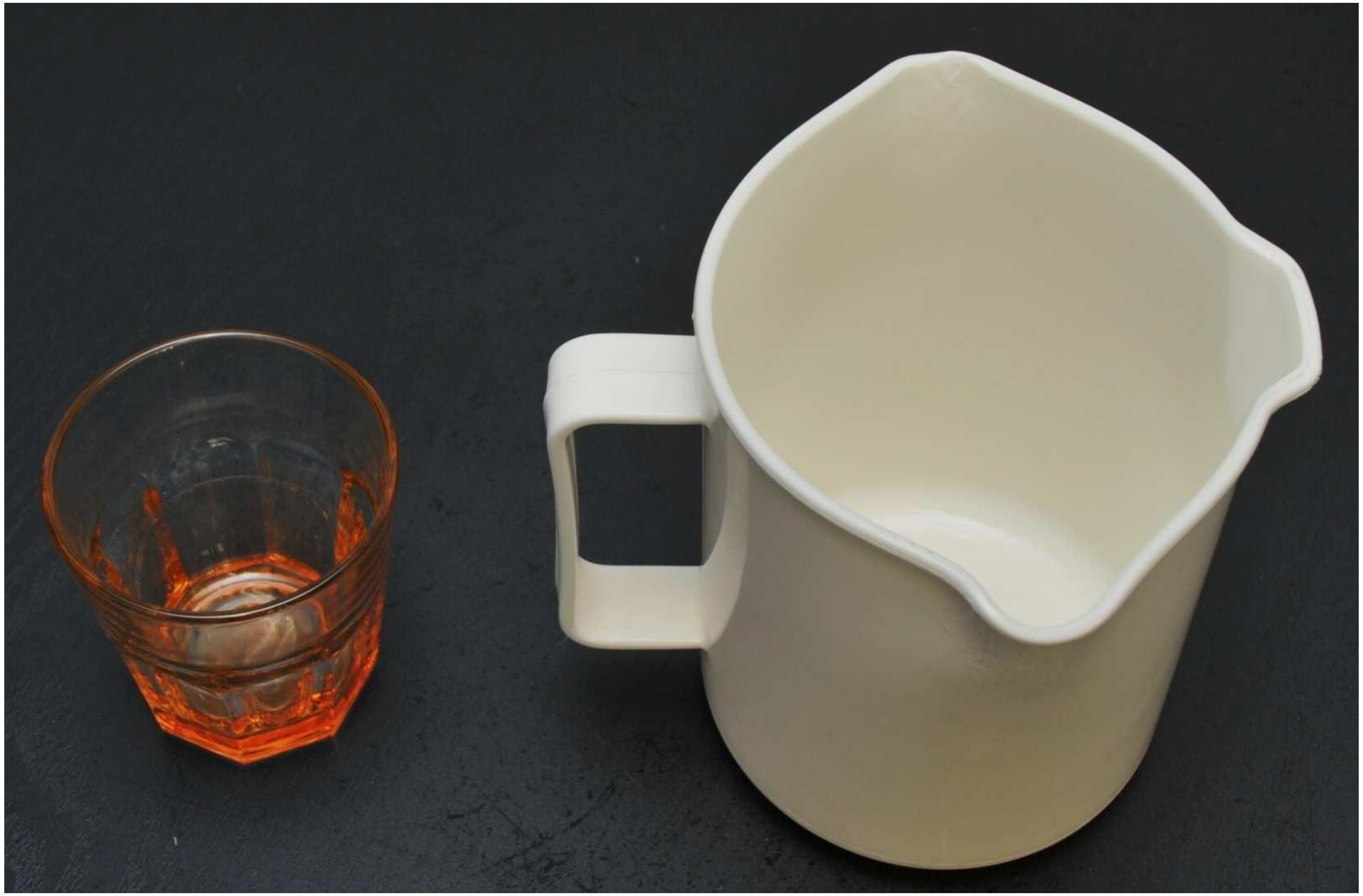

Test 4

One plate with everyday cutlery, and some modelling paste.

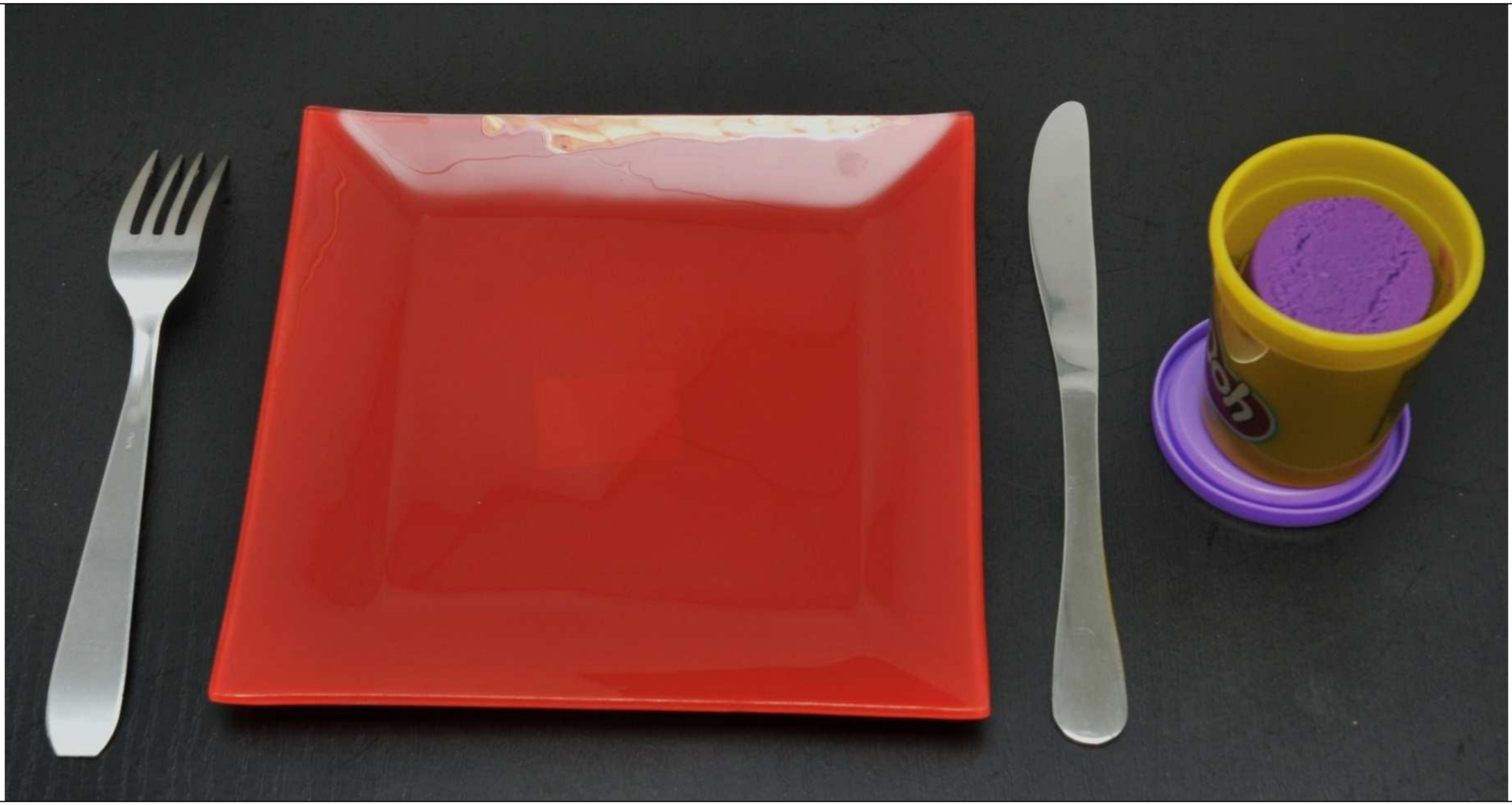

One jam jar with screw lid, filled with particles
One bottle of water (50ml) with screw cap
and one medicine tube with a push-on lid

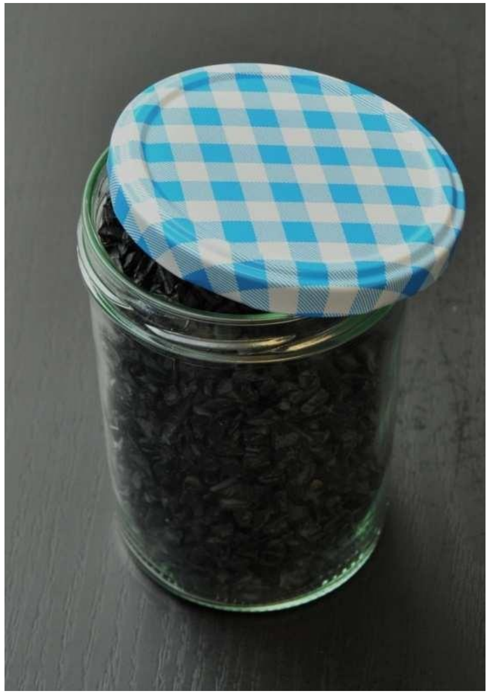
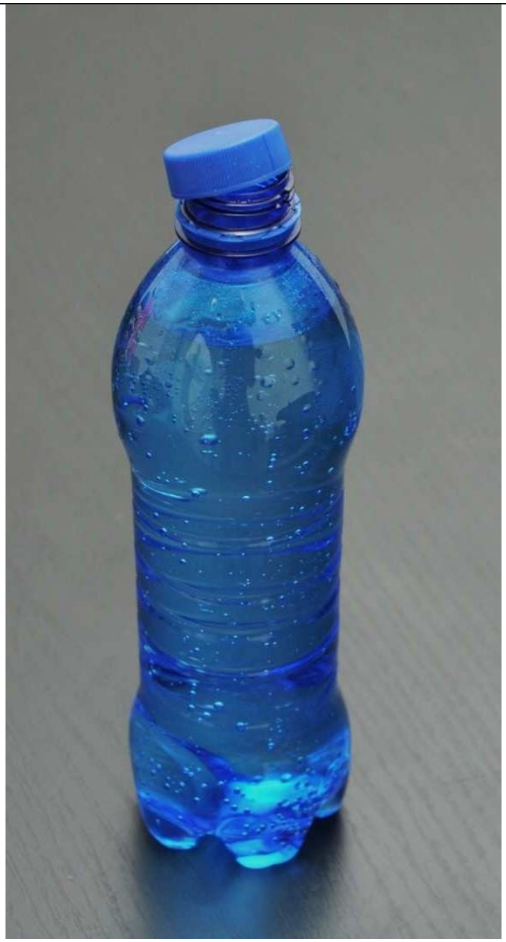
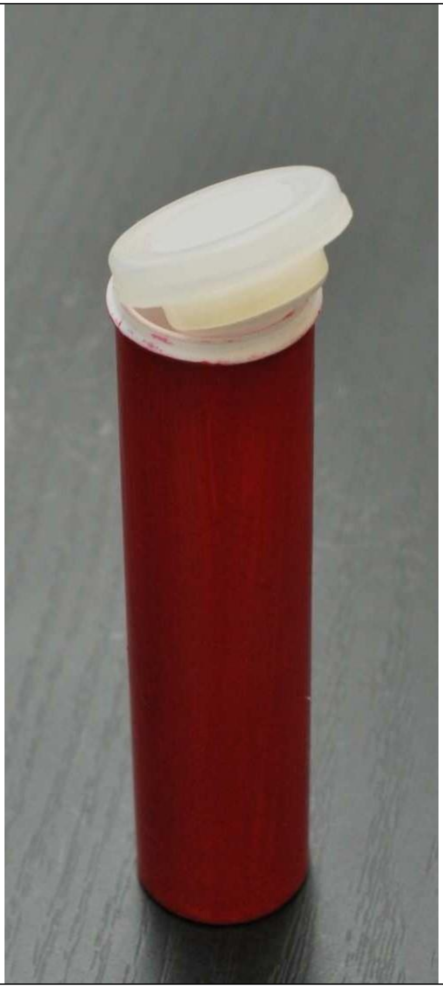

One bolt with 4mm nut
One large box of matches

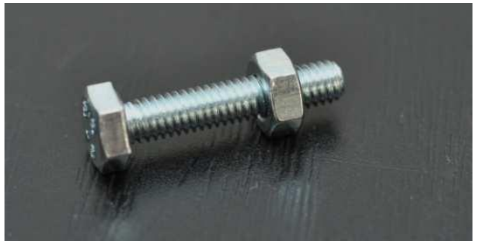

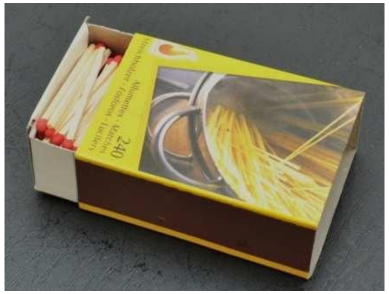

One shirt over a board, with 3 buttons 12mm in diameter, and a further board with 3 laces
One needle with a large eye, and some thread

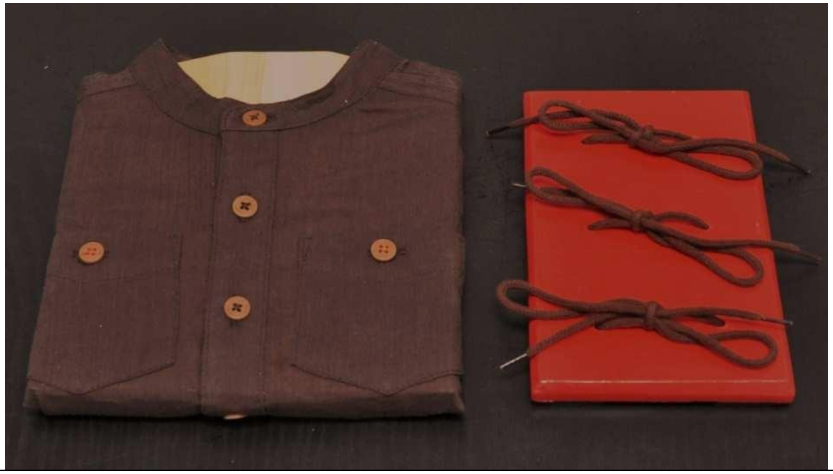

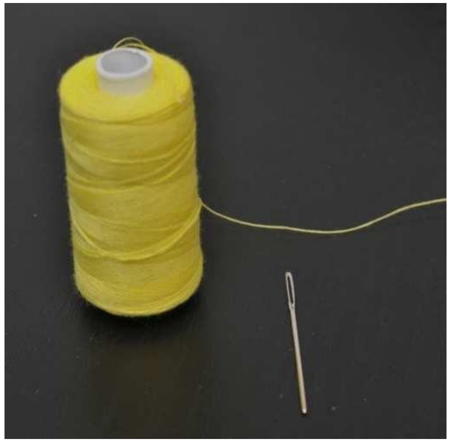

One purse (with press stud and zip fastening)

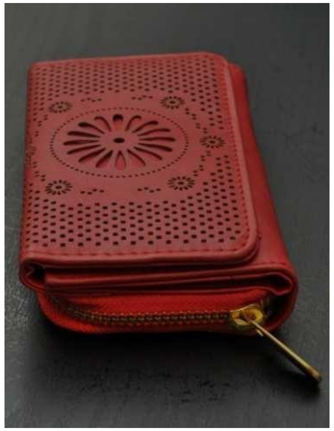	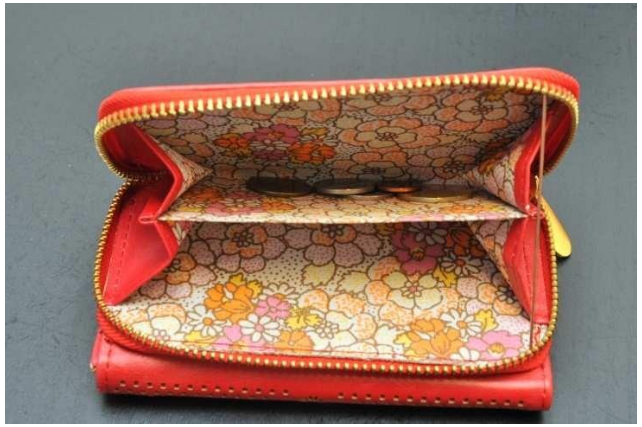	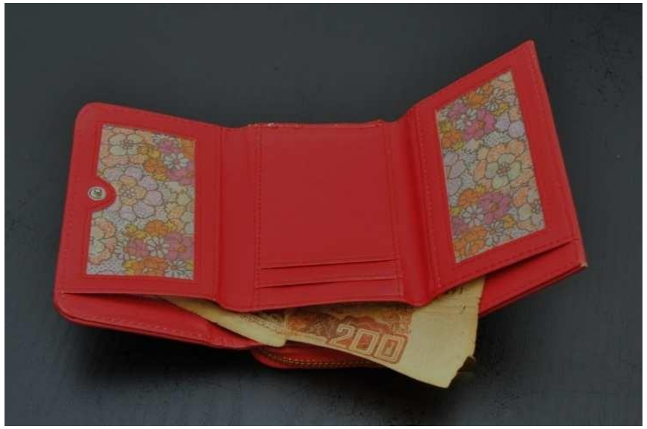

One A4 sheet of paper with a bic-style biro and a section of cube ruler 22cm long. Some pages of a newspaper (4 double sheets)

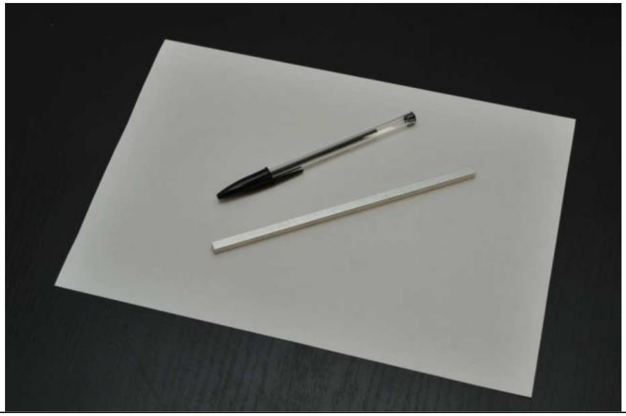	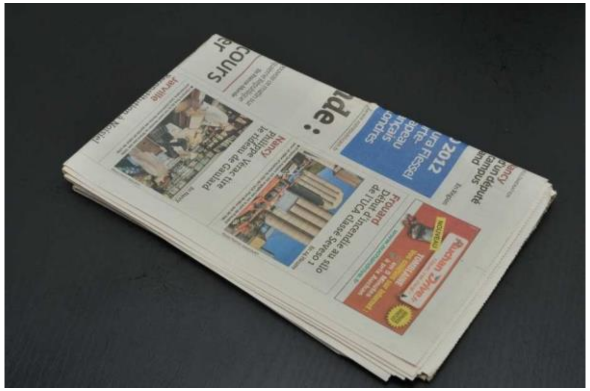

A piece of card (1mm thick and 20cm long) and some office scissors
Some copper wire (1mm) and some pliers to cut the end

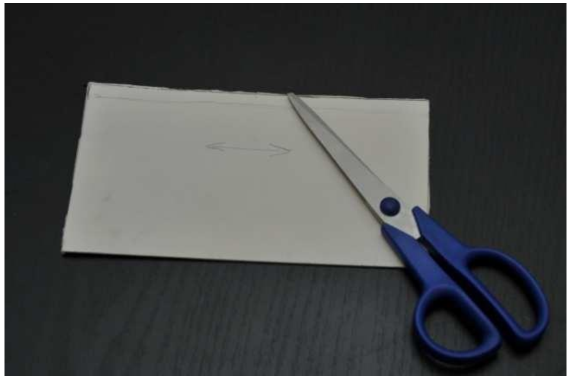	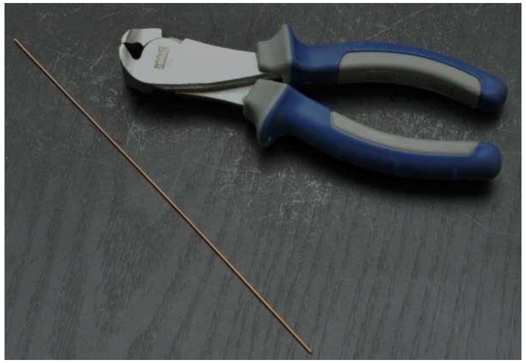
