# Supplementary material for: Prospective multicentre validation study of a new standardised version of the 400-point hand assessment
Source: BMC Musculoskelet Disord. 2020 May 20;21:313. doi: 10.1186/s12891-020-03303-4 (PMC7240941; doi:10.1186/s12891-020-03303-4)
Supplement: Supplementary file 3 — Additional file 3. [file 12891_2020_3303_MOESM3_ESM.pdf]

AVALIAÇÃO 400 PONTOS

(V 2)

Mme Colette GABLE, ergothérapeute
Professeur Jean PAYSANT

Institut Régional de Médecine Physique et de Réadaptation
75, Boulevard Lobau 54042 NANCY

Traduzido por: **Rosário Simões, Terapeuta Ocupacional**
Hospital Particular do Algarve-Gambelas
Faro, Portugal

INTRODUÇÃO

A avaliação da capacidade de preensão na sua complexidade é um verdadeiro desafio. A clássica utilização de avaliações orientadas apenas para um aspeto particular da função de preensão (articular, muscular, trófica, sensitiva), dá-nos apenas uma resposta fragmentada e incompleta da capacidade de preensão.

«A Avaliação 400 PONTOS» propõe uma avaliação a quatro níveis: motricidade – força – atividade monomanual – coordenação bimanual. A observação em cerca de 60 gestos da vida quotidiana, elucida-nos sobre a qualidade das preensões e capacidade de adaptação. A nota atribuída a cada prova «pontua» o setor deficitário.

Para a realização desta avaliação são necessários cerca de 30 a 45 minutos

Descrição

Esta avaliação é composta por quatro provas, cada uma cotada em 100 pontos:

1 – MOBILIDADE DA MÃO ....

2 – FORÇA DE PREENSÃO ....

3 –AGARRAR E DESLOCAR OBJECTOS MONOMANUALMENTE ....

4 – FUNÇÃO BIMANUAL ....

CADA UMA DESTA PROVAS COMPORTA:

- ✓ o seu material;
- ✓ o seu modo de aplicação;
- ✓ a sua cotação;
- ✓ o seu modo de cálculo.

A fiabilidade desta avaliação necessita de condições de realização constantes, a utilização do mesmo material e de instalações idênticas:

- Uma mesa regulável em altura (60 cm de largura e 90 cm de profundidade).
- Uma cadeira estável para o paciente, regulável em altura.
- Um banco de rodas regulável em altura, para o terapeuta.
- Um móvel com o material necessário e fácil de deslocar.
- Uma mesa regulável em altura, sobre a qual são colocados dois planos de referência com um espaço de 50 cm entre cada um: a mesa é regulável, para que o plano superior esteja à altura dos ombros do paciente que esteja a ser avaliado.

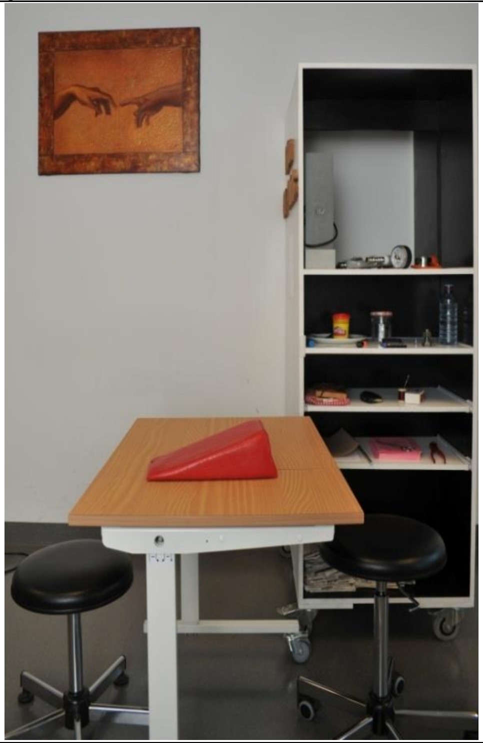	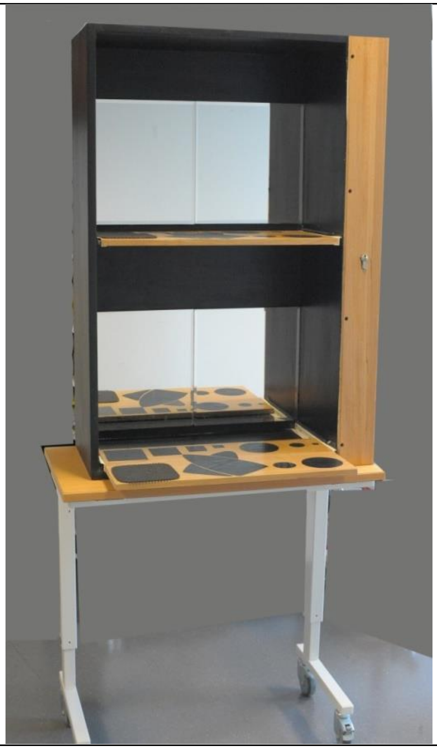	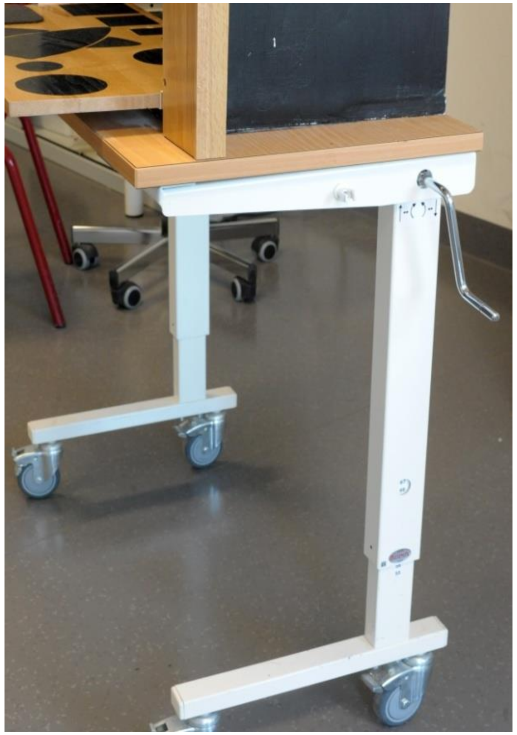

PROVA 1: MOBILIDADE DA MÃO

Trata-se de testar rem simultâneo as duas mãos do paciente, com catorze movimentos globais, anotando-se a qualidade de execução:

1. Fechar a mão
2. Abrir a mão
3. Afastamento dos dedos longos
4. Aproximação dos dedos longos
5. Afastamento do polegar
6. Adução do polegar
7. Oponência do polegar com os 4 dedos longos
8. Oponência do polegar com os 4 dedos longos, com Resistência
9. Pinças laterais com Resistência, utilizando um cartão ou carta
10. Preensões globais com 3 bastões contra resistência
11. Flexão do punho
12. Extensão do punho
13. Pronação
14. Supinação

Material

- Uma cunha triangular é utilizada para os 3 itens contra resistência.
- Uma carta do tipo de “jogar” ou cartão bancário
- Três cilindros de 22 cm de comprimento:
    - Um de 3 mm de diâmetro
    - Um de 10 mm de diâmetro
    - Um de 20 mm de diâmetro

Modo de realização

O paciente fica sentado confortavelmente a uma mesa, em frente ao terapeuta.

POSIÇÃO DE PARTIDA
**O antebraço em apoio sobre o bordo da mesa e as mãos elevadas acima da mesa.**

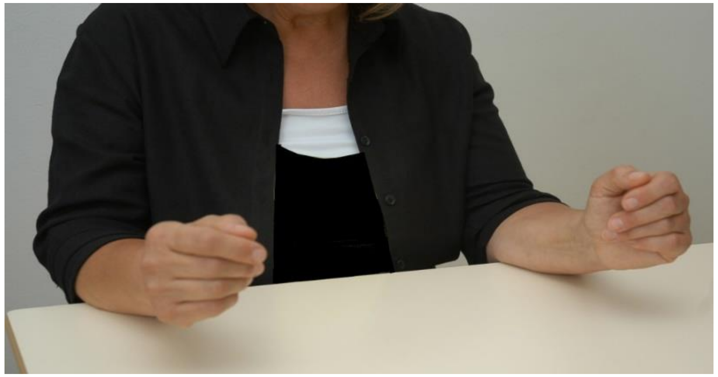	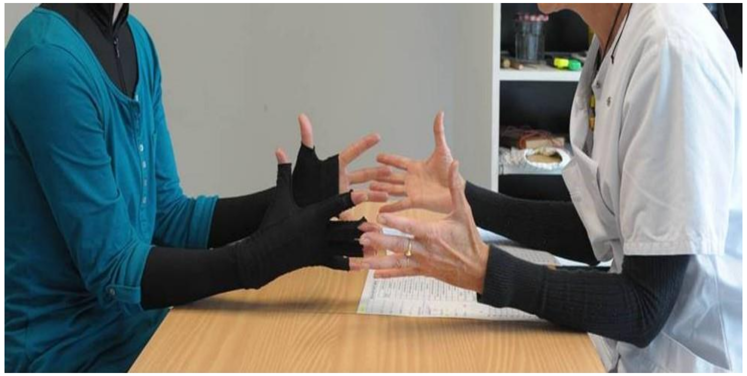

O Terapeuta Ocupacional explica os movimentos e exemplifica-os para o paciente reproduzir. As duas mãos são testadas ao mesmo tempo.
Para alguns itens, é conveniente fazer repetir os movimentos várias vezes de seguida, ou mesmo testar os dois movimentos de cada vez para que estes sejam mais espontâneos.

1 e 2 «Feche as duas mãos como se quisesse dar um soco, depois abri-las, fecha-las, abrir, fechar, abrir…»

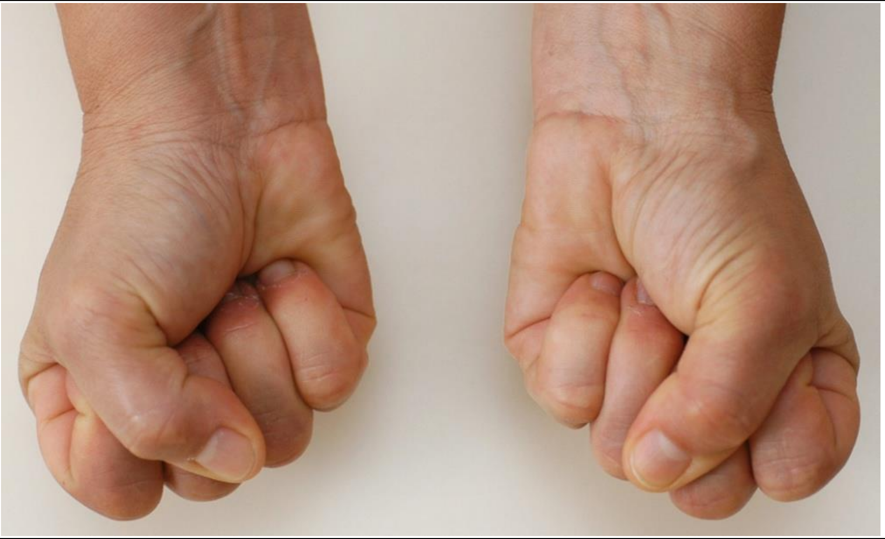

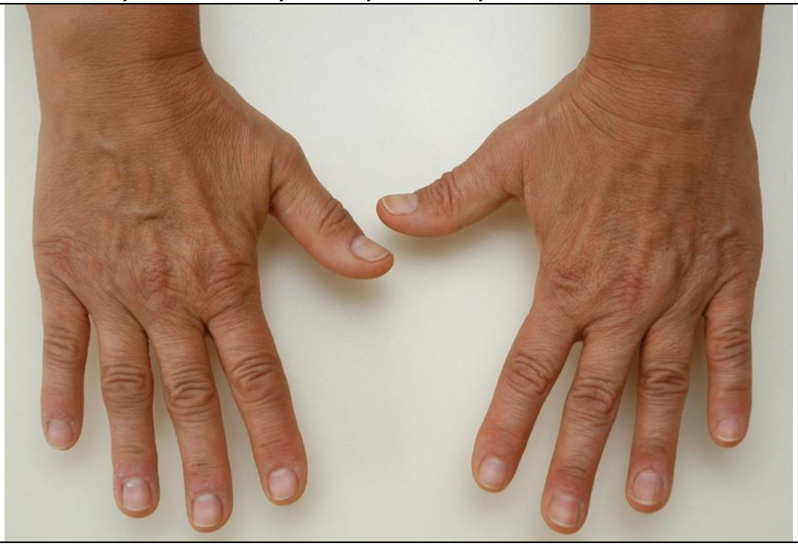

3 e 4 «Afaste os dedos longos das suas duas mãos, junta-los, afastar, juntar, afastar, juntar…»

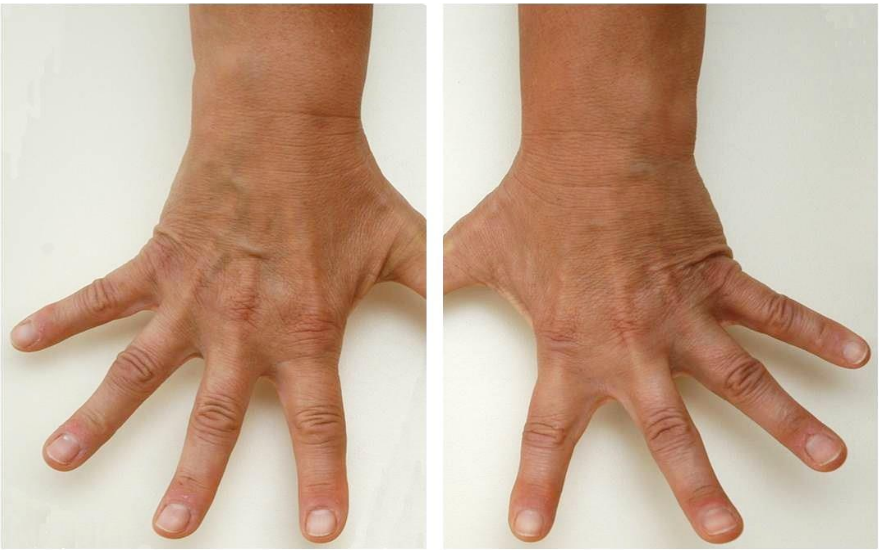

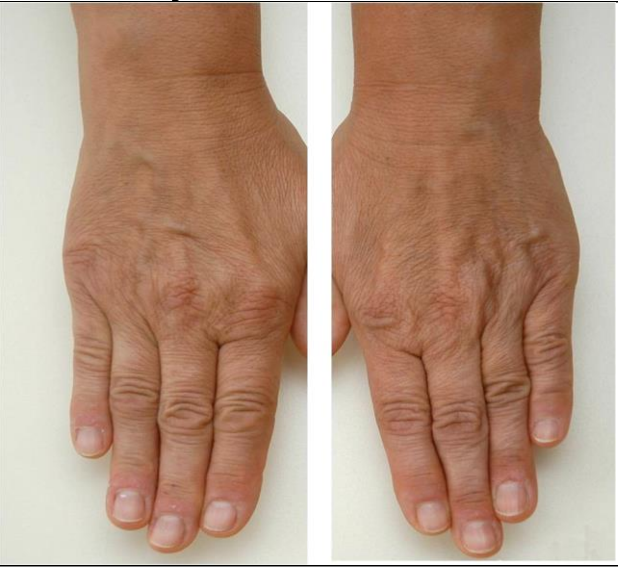

5 e 6 «Afaste os polegares esticados no mesmo plano que os outros dedos, junte-os, sempre esticados deslizando-os sobre o indicador até à 2ª comissura, afastar, juntar. … »

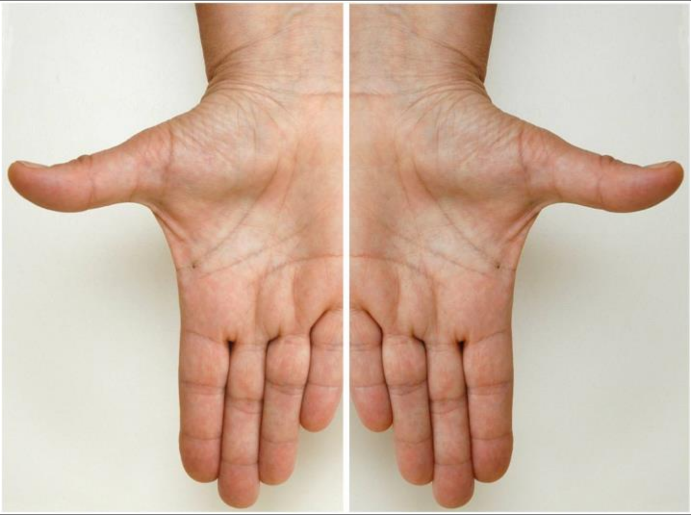

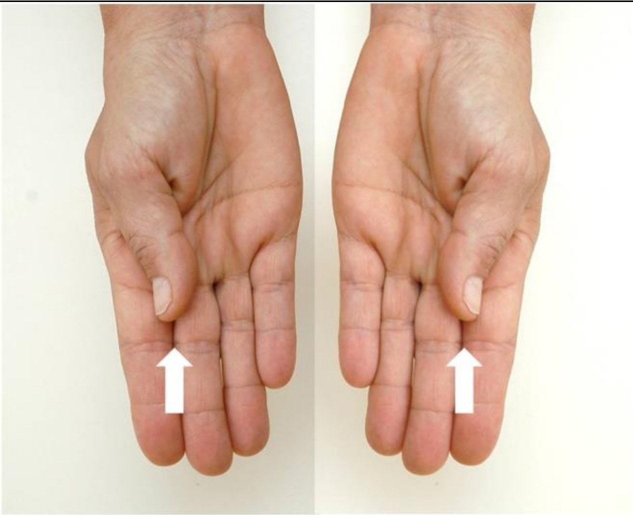

7 «Dos dois lados toque a ponta de cada dedo longo com o seu polegar fazendo bolas redondas, recomece …»

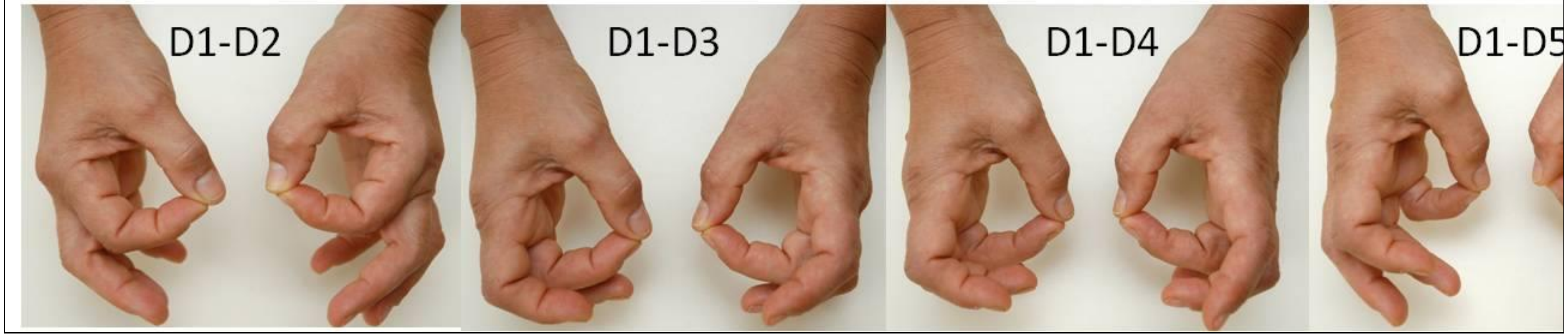

Atenção !!!Mesmo que uma só pinça «polegar-dedo longo» seja impossível de realizar, a nota global «1» será dada a este item.

Para os 3 itens contra resistência, cada mão é testada separadamente, o antebraço do paciente repousa sobre uma cunha triangular, a mão fica fora da cunha.
O Terapeuta Ocupacional começa por avaliar a mão sã do paciente, depois continua com a mão lesada efetuando as Resistências necessárias.

8 «Recomeçamos com as mesmas bolas redondas, mas desta vez resista à minha pressão, eu tento passar os meus dedos entre os seus (primeiro do lado são e depois do lado lesado) …»

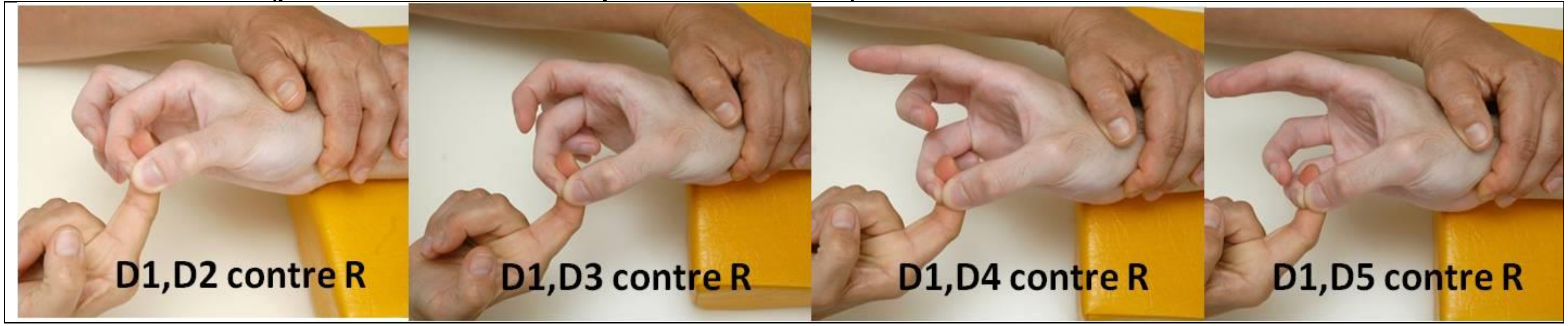

9 «Agora tente prender esta carta entre os seus dedos e eu vou puxar para tentar tira-la (primeiro do lado são e depois do lado lesado) …»

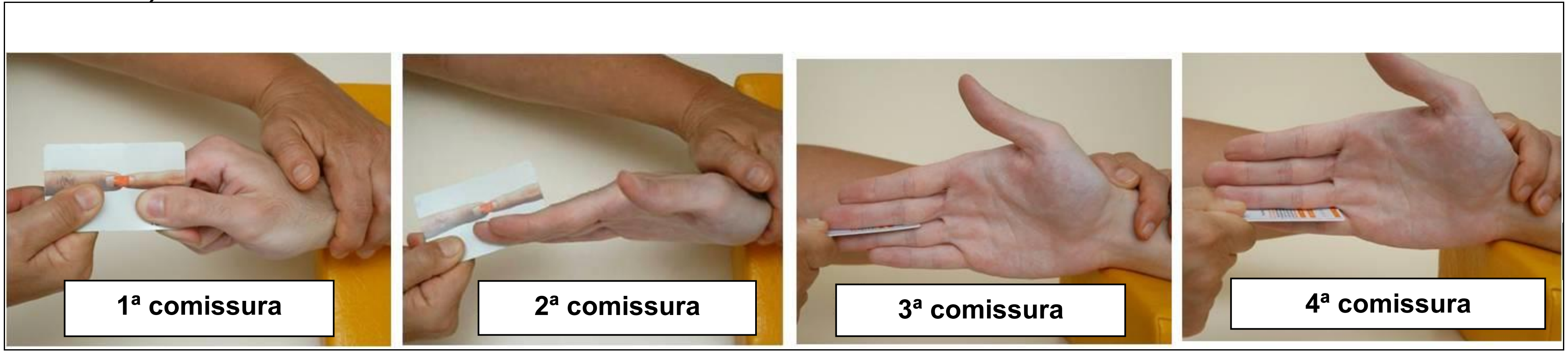

10 «Tente segurar na palma da mão este pequeno cilindro (bastão), suportado pelo terapeuta, com os dedos bem fechados, e eu vou puxar para tentar tirá-lo… (primeiro do lado são e depois do lado lesado, começando pelo de menor diâmetro até ao de maior diâmetro) …».

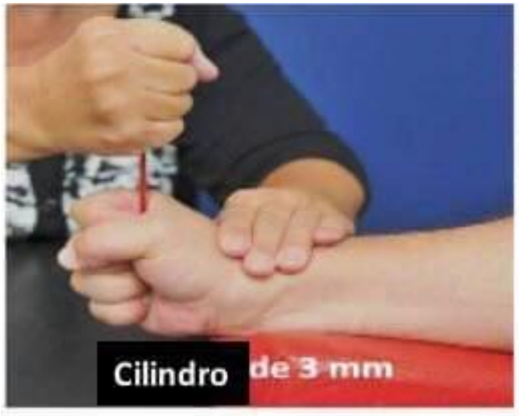

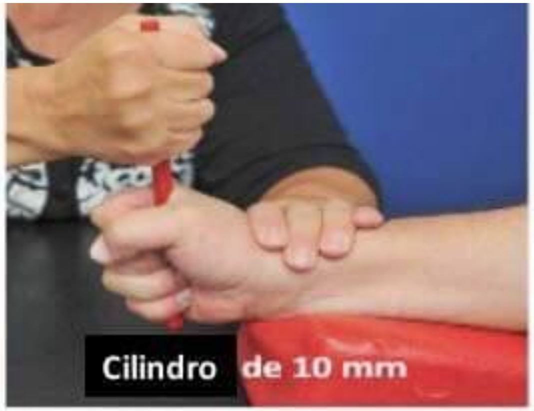

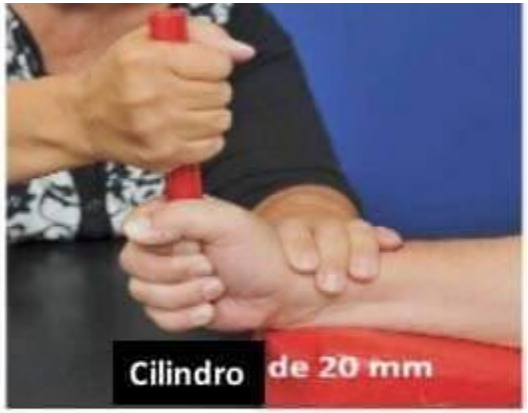

11 «entrelace os dedos como se estivesse a rezar, depois levante os cotovelos o mais possível até fazer uma linha horizontal ao nível do peito»

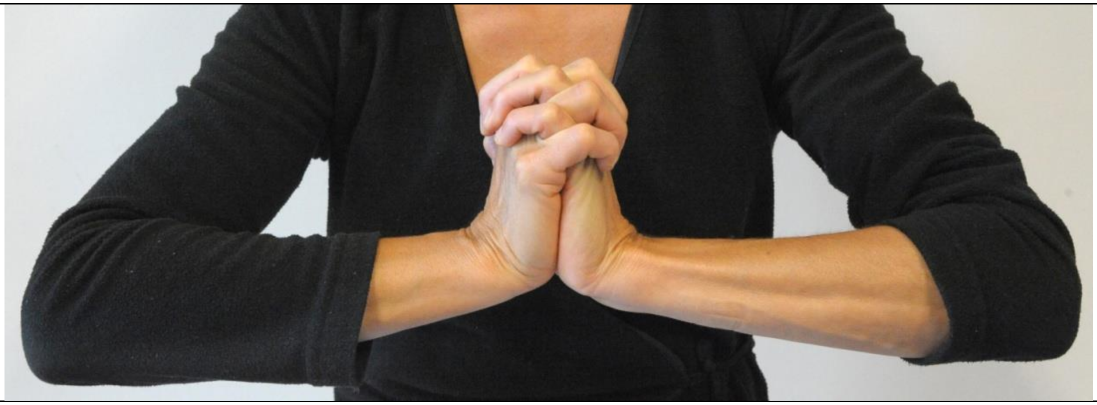

12 «junte as suas mãos com os dedos esticados e baixe os cotovelos até fazer uma linha horizontal junto ao peito …»

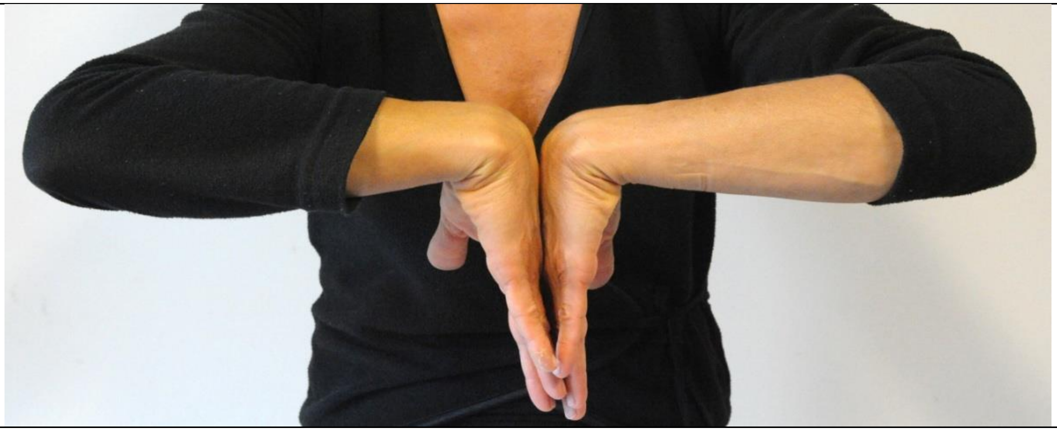

Para os itens 13 e 14, o paciente afasta-se da mesa, mantém os cotovelos colados ao corpo e fletidos a 90, os polegares esticados em direção ao teto.

13 e 14 «Afaste-se da mesa, coloque os seus cotovelos colados ao corpo e dobrados a 90º, com os seus polegares virados para cima. Vire as palmas das mãos para o teto, depois vire-as para o chão, para o teto, para o chão…»

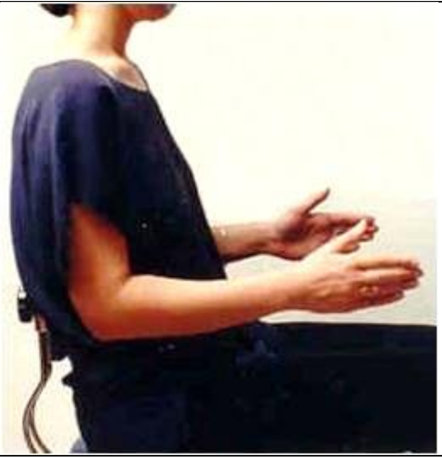

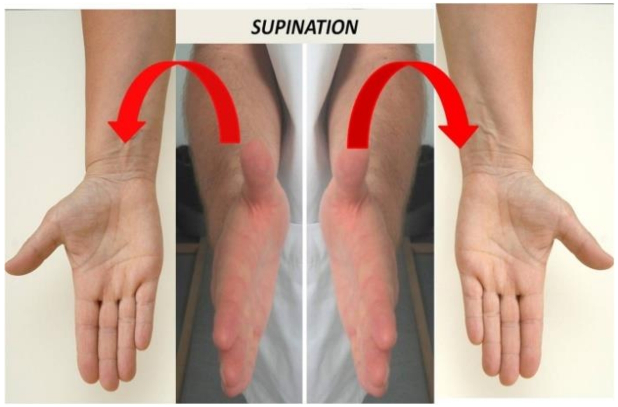

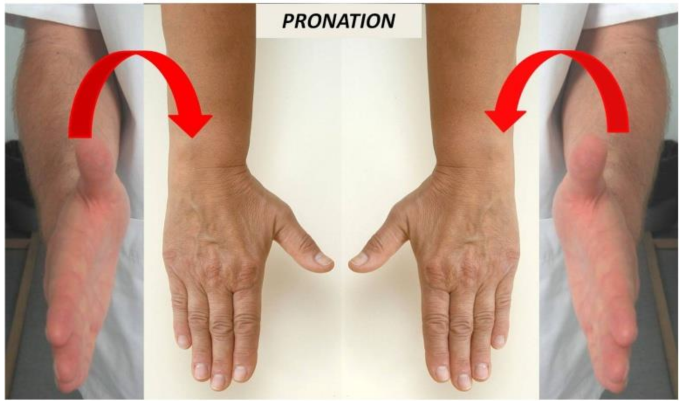

Cotação

Cotação para os itens de 1 a 7 e 11 a 14	MOVIMENTO COMPLETO *, HARMONIOSO E PRECISO	MOVIMENTO COMPLETO, MAS LENTO, DESARMONIOSO OU IMPRECISO	MOVIMENTO INCOMPLETO OU COM EXCLUSÃO OU COM COMPENSAÇÃO	MOVIMENTO IMPOSSIVEL**
	3	2	1	0

***Um «movimento completo» é um movimento efetuado em todas as suas amplitudes e com todas as suas qualidades: harmonia do gesto, boa velocidade e precisão graças à integridade física da mão, do punho e do comando voluntário*.**

****Um movimento pode ser «impossível» devido a uma contra indicação médica ou por uma incapacidade provisória ou definitiva.***

Cotação especifica para os movimentos contra resistência:

Para os itens 8 e 9

COTAÇÃO PARA OS ITENS 8 E 9	AGARRAR E MANTER COM UMA FORTE RESISTÊNCIA EFETUADA PELO TERAPEUTA	AGARRAR E MANTER COM UMA RESISTÊNCIA MÉDIA EFETUADA PELO TERAPEUTA	AGARRAR E MANTER COM UMA RESISTÊNCIA LIGEIRA EFETUADA PELO TERAPEUTA	AGARRAR SEM RESISTÊNCIA
	3	2	1	0

Para o item 10

COTAÇÃO PARA O ITEM 10	BASTÃO 3 MM MANTIDO CONTRA RESISTÊNCIA EFETUADA PELO TERAPEUTA	BASTÃO 10 MM MANTIDO CONTRA RESISTÊNCIA EFETUADA PELO TERAPEUTA	BASTÃO 20 MM MANTIDO CONTRA RESISTÊNCIA EFETUADA PELO TERAPEUTA	BASTÃO 3 MM NÃO MANTIDO CONTRA RESISTÊNCIA EFETUADA PELO TERAPEUTA
	3	2	1	0

Coeficientes

Todos estes itens não têm todos a mesma importância na função da mão, assim como cada um deles possui um coeficiente moderador de forma a hierarquiza-los.
Com efeito, é mais importante fechar completamente a mão do que abrir completamente, assim o coeficiente para o item fechar a mão é de 3 e para abrir a mão é de 2.

Quadro de coeficientes moderadores

FECHAR A MÃO	3
ABRIR A MÃO	2
AFASTAMENTO DOS DEDOS LONGOS	3
APROXIMAÇÃO DOS DEDOS LONGOS	2
ABDUÇÃO DO POLEGAR	3
ADUÇÃO DO POLEGAR	2
OPONÊNCIA DO POLEGAR AOS 4 DEDOS LONGOS	3
OPONÊNCIA DO POLEGAR AOS 4 DEDOS LONGOS CONTRA RESISTÊNCIA	2
PINÇAS LATERAIS COM UMA CARTA, CONTRA RESISTÊNCIA	2
PREENSÕES GLOBAIS DE 3 BASTÕES, CONTRA RESISTÊNCIA	3
FLEXÃO DO PUNHO	2
EXTENSÃO DO PUNHO	3
PRONAÇÃO	3
SUPINAÇÃO	2

Modo de cálculo

- Cada item é cotado de **0 a 3**.
- Cada cotação é **multiplicada pelo seu coeficiente** para se obter uma nota.
- O total da prova é obtido adicionando as 14 notas, o resultado **corresponde á soma das notas do lado lesado**
- Esta nota é **dividida por 105** (score máximo) depois multiplicada por **100** para obter a **percentagem de mobilidade da mão lesada em relação à mobilidade de uma mão sã.**

Exemplo: Score de uma mão sã = score máximo = 105 pontos
Score da mão lesada = 69

Far-se-á: $\frac{69}{105} \times 100 = 65{,}7$

Atenção!!!
No cálculo do resultado final da prova, se os valores não estão arredondados convém:
Arredondar para o número inferior quando a casa decimal é inferior ou igual a 5 e arredondar para o valor superior quando a casa decimal é superior a 5: **Assim 65,7 será 66 /10**

Interpretação: O paciente tem uma mobilidade de 66% ao nível da sua mão lesada (em relação a uma mão sã)

PROVA 2: FORÇA DE PREÊNSÃO

A medida da força de preensão faz–se com dois instrumentos de medida. Ela é feita primeiro no lado são e depois no lado lesado. Para o paciente se familiarizar, cada um dos aparelhos pode ser experimentado com o lado são antes de começar a prova.
Trata-se então de testar:
1• A FORÇA DO PUNHO com a ajuda de um dinamómetro de Jamar (regulado na segunda abertura), referência internacional, testando os músculos extrínsecos e os intrínsecos.
2• A FORÇA da pinça lateral entre a polpa do polegar e o bordo radial do indicador com ajuda de um dinamómetro “Pinchmeter”, testando intrínsecos e extrínsecos do polegar.

Material

- Um dinamómetro Jamar
- Um dinamómetro “Pinchmeter”

Modo de execução

Posição de partida: o paciente é instalado conforme as recomendações da Sociedade Americana de Reeducação da Mão:

- Sentado com as costas direitas
- Os pés assentes no chão
- Os ombros em adução neutra
- Os braços sem suporte
- Os cotovelos fletidos a 90°
- O antebraço em posição neutra
- O punho de 0 a 30° de extensão com um desvio cubital de 0 a 15º.

Os dinamómetros são suportados pelo Terapeuta Ocupacional. Para cada dinamómetro, o score é obtido fazendo a média das 3 medidas sucessivas. Estas são feitas alternadamente do lado são, depois lado lesado, depois lado são, lado lesado….

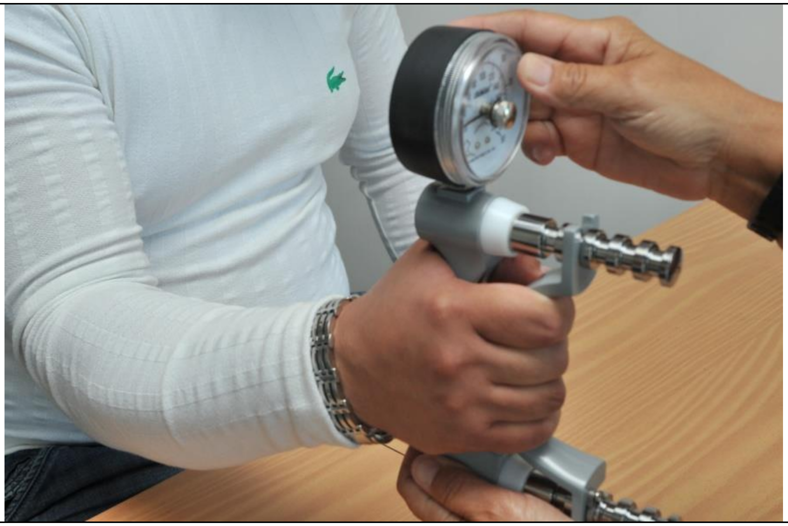

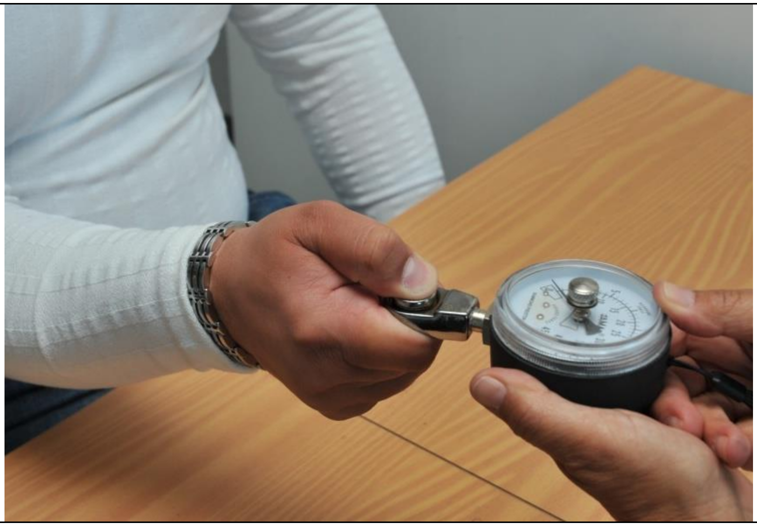

Modo de cálculo

Convencionou-se que a mão sã tem uma força máxima.
O valor obtido por esta, em cada um dos dinamómetros corresponde à nota máxima ou seja 10/10. Para cada dinamómetro a mão lesada é cotada em relação à mão sã, a sua nota é obtida fazendo uma regra de três simples:
Para cada dinamómetro a mão lesada é cotada em relação à mão sã, a sua nota é obtida fazendo uma regra de três simples:

°Por exemplo, para o dinamómetro Jamar:

Lado são = 48 kg; a nota do lado são corresponde a 10/10.
Lado lesado = 18 kg; a nota do lado lesado corresponde a:

Valor lado lesado ----------------------- X 10 = será Valor lado são **18 ---------- x 10 = 3,7 / 10 48**

$$\frac{\text{Valor lado lesado}}{\text{Valor lado são}} \times 10 = \text{será} \quad \frac{18}{48} \times 10 = 3{,}7 / 10$$

° Para o dinamómetro Pinch, os cálculos são os mesmos:

Lado são = 9,5 kg; a nota do lado são corresponde a 10/10.
Lado lesado = 3,5 kg; a nota do lado lesado corresponde a:

$$\frac{\text{Valor lado lesado}}{\text{Valor lado são}} \times 10 = \text{será} \quad \frac{3{,}5}{9{,}5} \times 10 = 1{,}2 / 10$$

Coeficientes

Cada um dos 2 dinamómetros tem um coeficiente de 5. Assim, no nosso exemplo, o lado lesado do paciente obtém:

° para o dinamómetro Jamar: 3,7 x 5 será 18,5 / 50.
° para o dinamómetro pinch: 1,2 x 5 será 6 / 50.
A soma para o lado lesado é feita: 18, 5 + 6 = 24,5

O total será de 100 do lado são e de 24,5 / 100.

Ponderação

Na literatura, os estudos de medida da força de preensão estabelecem em regra geral 8 a 12% de força de preensão a mais ao nível da mão dominante.

Assim convém reajustar o resultado:

°**se a mão dominante é a mão lesada,** são retirados 10% ao score obtido
°**se a mão não dominante é a mão lesada,** são adicionados 10% ao score obtido

Assim, no nosso exemplo:

°Total das notas lado são e não dominante: 100 /100

°Total das notas do lado lesado e dominante: 24,5 /100

Se a mão lesada for a dominante, é conveniente retirar 10%, retirar 2,4 a 24,5.
Obtemos: 24,5 – 2,4 = 22,1 será **22/100**

- ***Interpretação: Podemos dizer que este paciente tem uma força de preensão de 22% em relação à sua mão sã.***

- ***Se as duas mãos estão lesadas, o score obtido é comparado às médias estabelecidas a partir de um estudo suiço efetuado numa população sã, (496 homens e 482 mulheres dos 18 aos 85 anos ou mais) em 2009:***

FORÇA NO JAMAR EM KG					
**JAMAR HOMENS (496)**			**JAMAR MULHERES (482)**		
Idade	Mão Dominante	Mão não dominante	Idade	Mão Dominante	Mão não dominante
18 a 19 (33)	51,2	48,3	18 a 19 (31)	32	30,7
20 a 29 (59)	53,4	50,8	20 a29 (61)	33,8	32,5
30 a 39 (69)	55,4	53	30 a 39 (72)	34,8	33,6
40 a 49 (68)	53	56,7	40 a 49 (79)	34	34,1
50 a 59 (70)	52,2	55	50 a 59 (62)	32,8	32,6
60 a 69 (79)	45,4	44,9	60 a 69 (64)	29,1	28
70 a 79 (61)	39,2	38,7	70 a 79 (53)	25,7	24,8
80 a 85 (29)	30,7	29,4	80 a 85 (32)	19,2	19,7
> 85 (28)	22,4	23,2	> 85 (28)	16,9	16,7

FORÇA NO PINCH EM KG					
**PINCH HOMENS (496)**			**PINCH MULHERES (482)**		
Idade	Mão Dominante	Mão não dominante	Idade	Mão Dominante	Mão não dominante
18 a19 (33)	9,5	9,1	18 a 19 (31)	6,9	6,5
20 a 29 (59)	9,9	9,3	20 a 29 (61)	6,6	6,4
30 a 39 (69)	10,1	9,7	30 a 39 (72)	7	6,7
40 a 49 (68)	10	9,6	40 a49 (79)	7,1	6,8
50 a 59 (70)	10	9,6	50 a 59 (62)	6,8	6,6
60 a 69 (79)	9,2	8,8	60 a 69 (64)	6,5	6,2
70 a 79 (61)	8,2	7,8	70 a 79 (53)	5,4	5
80 a 85 (29)	6,4	6,5	80 a 85 (32)	4,3	3,9
> 85 (28)	5,4	5,5	> 85 (28)	3,1	2,8

PROVA MONOMANUAL 3: AGARRAR E DESLOCAR OBJECTOS MONOMANUALMENTE

Trata-se de testar a capacidade do paciente em:

- Agarrar em 20 objetos de tamanho, peso e forma diferentes colocados sobre um plano de referência numa ordem precisa.
- Transporta-los para um plano 50 cm mais alto e coloca-los nos sítios correspondentes.
- A prova começa com a mão sã e depois prossegue com a mão lesada. É cronometrada, assim o paciente concentra preferencialmente a sua atenção sobre a rapidez de execução do que a forma como o faz.

O paciente é assim mais natural e espontâneo nos seus gestos, o terapeuta ocupacional pode assim facilmente observar os defeitos de preensão como a exclusão, os defeitos de comando, as compensações……

Material

1-Um cubo de 10 cm de lado (700gr)
2- Um cubo de 7,5 cm de lado (300 gr)
3- Um cubo de 5 cm de lado (100 gr)
4- Um cubo de 2,5 cm de lado (10 gr)
5-Um cilindro de 10 cm de diâmetro e 12 cm de altura (700 gr)
6- Um cilindro de 7,5 cm de diâmetro e 11 cm de altura (300 gr)
7- Um cilindro de 5 cm de diâmetro e 10 cm de altura (100gr)
8- Um berlinde de 25 mm de diâmetro
9- Um berlinde de 16 mm de diâmetro
10-Uma bola de ténis
11-Um parafuso de 2 mm de diâmetro e 4cm de comprimento
12-Um isqueiro
13- Uma moeda de 15 mm de diâmetro
14-Uma moeda de 25 mm de diâmetro
15-Uma moeda de 30 mm de diâmetro
16-Uma chave plana
17-A fechadura correspondente
18-Um ferro de engomar com 2 kg
19-Um jarro com dois bicos contendo 500 ml de água
20-Um copo vulgar

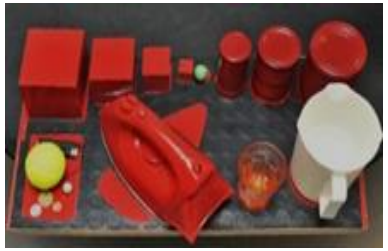

-1 plano de referência (30 cm por 60 cm) espaçado de um outro plano (de 30 cm por 6O cm) 50 cm mais alto.
A estrutura é colocada sobre uma mesa regulável em altura ou na parede com um sistema que permita regular em altura.
A mesa é regulada de forma a que o plano superior esteja à altura dos ombros do paciente em avaliação

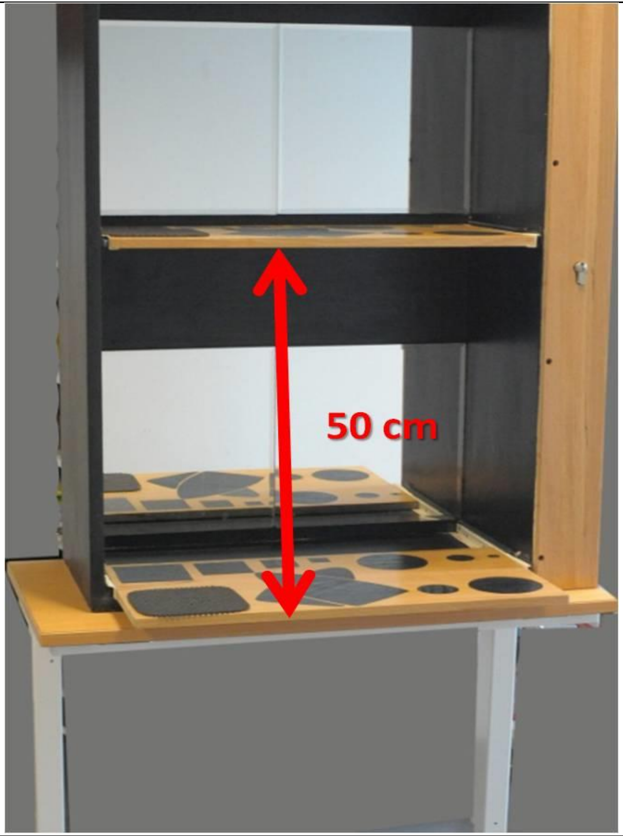

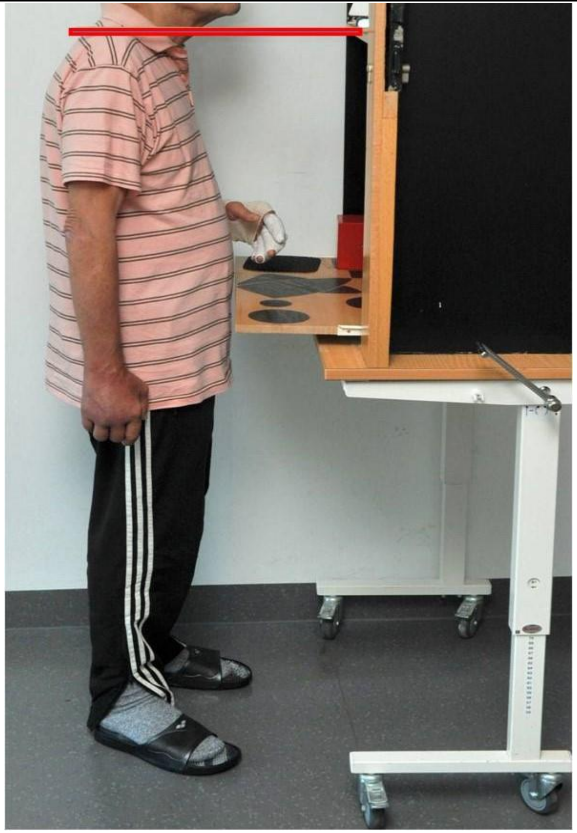

Um espelho colocado no fundo de cada prateleira permite uma observação mais fácil da mão.

Modo de aplicação

Atenção!!

3 objetos necessitam de uma ação antes de serem transportados para o plano mais alto:

-O isqueiro que se deve acender com uma pressão com o polegar
- A chave que se deve colocar na fechadura e rodá-la antes de a retirar e de a colocar sob o plano superior.
- A água do jarro com o qual se deve despejar uma parte da água em pronação até meio do copo, e depois o resto em supinação. A água do copo é de seguida colocada no jarro antes de transportar ambas as coisas, o jarro e copo para o plano superior.

Antes de iniciar a prova, o terapeuta ocupacional mostra ao paciente a maneira de realizar tudo explicando-lhe que ele vai ser cronometrado e deverá fazer o mais rápido possível:

«Agarre os objetos um a um, começando pela esquerda e continuando para a direita, primeiro a fila de trás, depois faça o mesmo da esquerda para a direita com os objetos da fila da frente.»
«Coloque os objetos nos espaços correspondentes, não se esquecendo de acender o isqueiro, de colocar a chave na fechadura e de deitar a água no jarro em pronação e em supinação»
«Faça o mais rápido possível pois está a ser cronometrado.»
«Está preparado? comeҫe !!…»

De seguida um exemplo de cada preensão, vista radial e cubital de cada um dos objetos assim como a ação a realizar em alguns deles. As imagens têm em conta o respeito pela ordem das preensões, e a regulação correta dos dois planos de referência

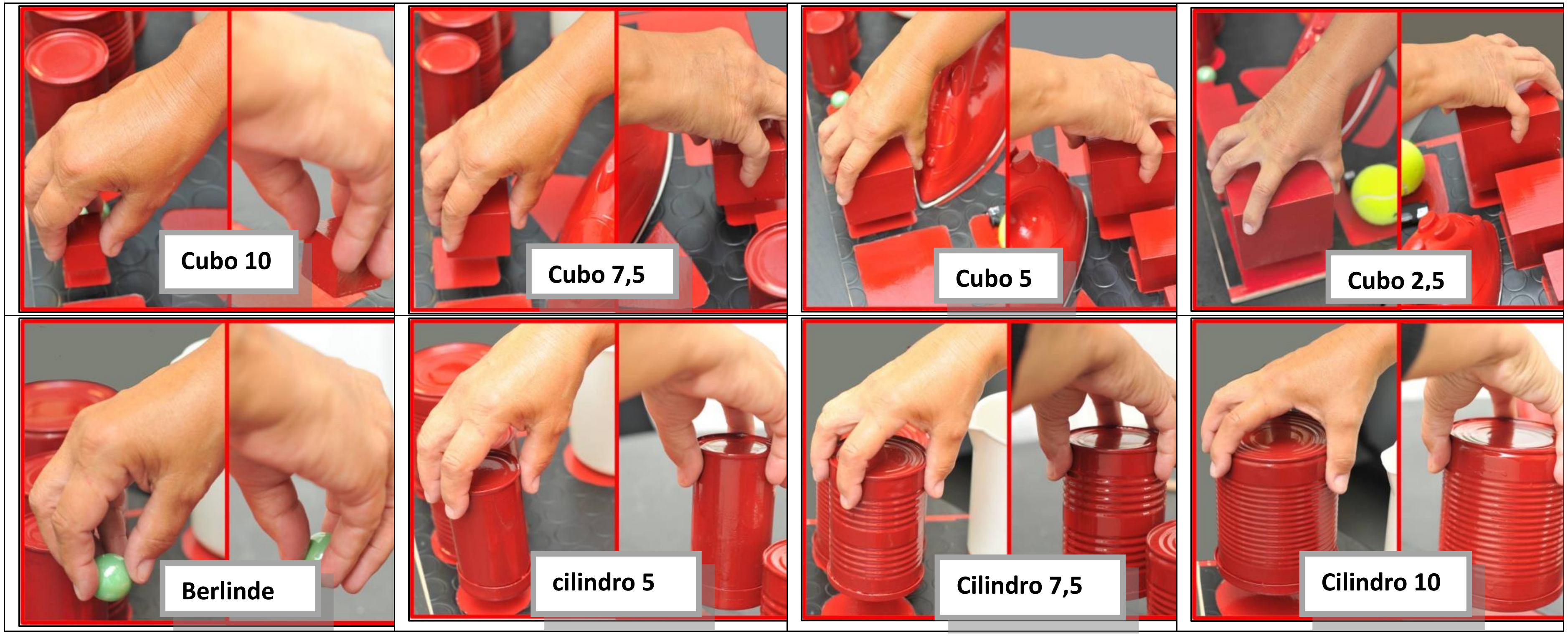

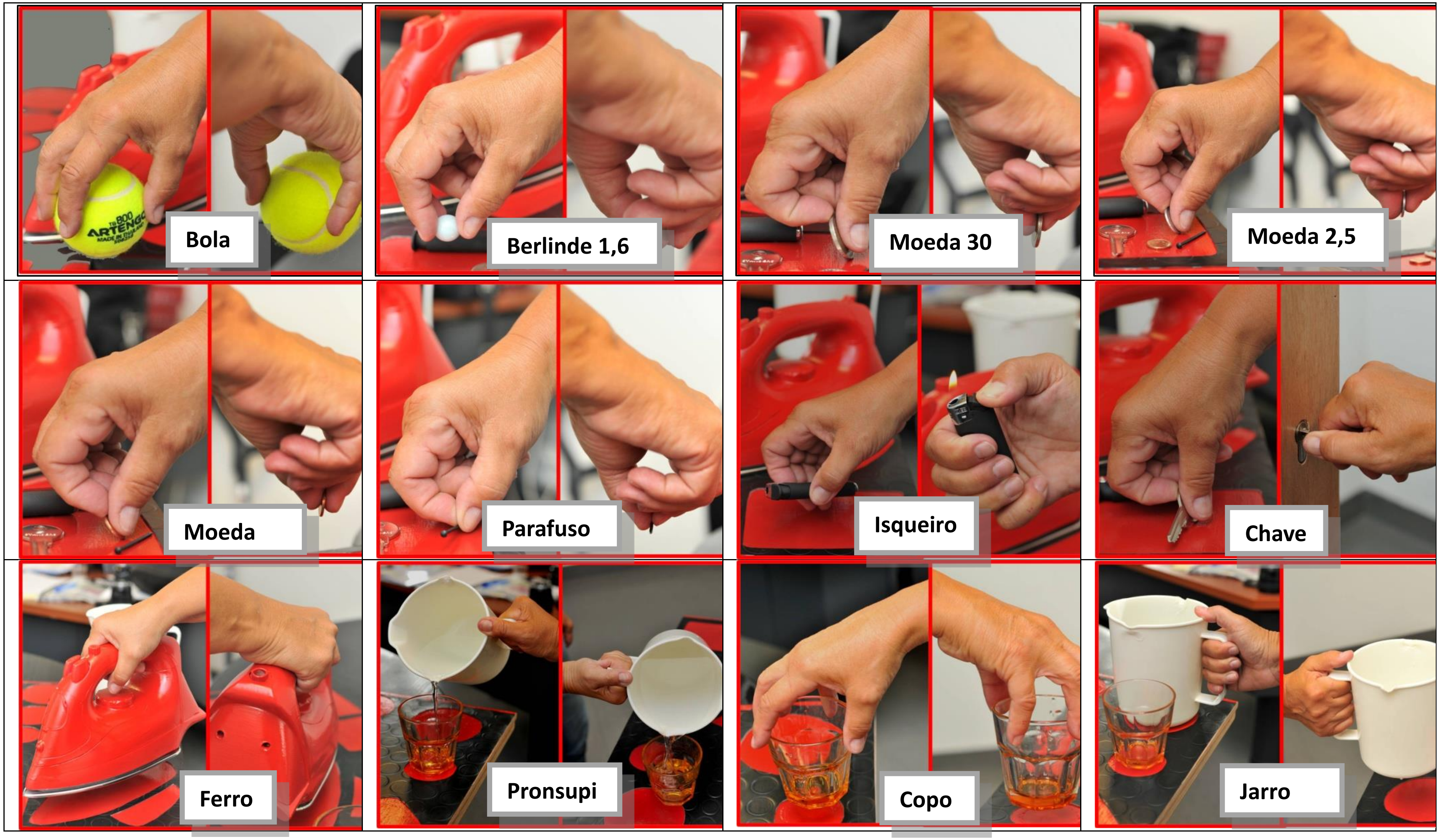
Bola
Berlinde 1,6
Moeda 30
Moeda 2,5
Moeda
Parafuso
Isqueiro
Chave
Ferro
Pronsupi
Copo
Jarro

COTAÇÃO

Para a cotação é necessário observar-se o agarrar, mas também o deslocar. A estrutura do móvel permite-nos uma boa observação e um registo rápido.

COTAÇÃO	AGARRAR CONFORME, HARMONIOSO E PRECISO	AGARRAR CONFORME, MAS LENTO, DESARMONIOSO OU IMPRECISO	AGARRAR COM EXCLUSÃO OU COMPENSAÇÃO	AGARRAR IMPOSSIVEL
**DESLOCAÇÃO RÁPIDA E PRECISA**	3	2	1	0
**DESLOCAÇÃO LENTA OU IMPRECISA**	2	2	1	0
**DESLOCAÇÃO COM COMPENSAÇÃO**	1	1	1	0
**DESLOCAÇÃO NÃO REALIZADA**	0	0	0	0

Assim, é suficiente que só o agarrar ou só ou o deslocar do objeto seja incorreto para que o item seja cotado com penalização.
Um paciente que apresente uma sequela definitiva (amputação digital, artrodese…) e efetue os itens nas condições próximas das normais, com uma otimização das suas possibilidades restantes. Nesses casos as compensações indispensáveis são permitidas.

Atenção!!
O tempo médio para realizar esta prova é inferior ou igual a um minuto para uma mão sã.
Em caso de ultrapassar o tempo, o score não é modificado, no entanto isso serve de informação suplementar para o terapeuta ocupacional na reeducação do seu paciente

Modo de cálculo

Cada item é cotado de 0 a 3.
O total da prova é obtido fazendo o total das 20 notas: esse total corresponde à soma das notas do lado lesado. Esta soma é de seguida dividida por 60 (score máximo) depois multiplicado por 100 para obter **a percentagem de utilização monomanual da mão lesada em relação a uma mão sã**.

Exemplo

- A soma das notas do lado são é igual ao score máximo, ou seja, 60 pontos.
- Aqui a soma das notas do lado lesado é igual a 46 pontos.

Depois, uma regra de três simples é aplicada dividindo a soma das notas do lado lesado pelo score máximo e multiplicando por 100:

$$\frac{46}{60} \times 100 = 76{,}6\%$$

Atenção!!!

Aquando do cálculo do resultado final, é conveniente arredondar para baixo quando a casa decimal é inferior ou igual a 5 e para cima quando a casa decimal é superior a 5

Assim, 76,6 será **77/100.**

Interpretação: Podemos dizer que este paciente tem uma utilização monomanual de 77% da sua mão lesada em relação às suas capacidades anteriores.

PROVA 4: FUNÇÃO BIMANUAL

Trata-se de testar a função bimanual do paciente com 20 tarefas da Vida Quotidiana verificando o respeito pela dominância. Esta tarefa é feita ao ritmo do paciente, sem cronometragem e sem conselhos particulares. No entanto esta prova não deverá exceder os 15 minutos.

Material

1. Um banco
2. Pasta de modelar
3. Talheres
4. Frasco de compota (tampa de rosca)
5. Garrafa de água (50 ml) com tampa de rosca
6. Porca e parafuso de 4 mm
7. Frasco de medicamento com tampa de abrir e não de desenroscar
8. Camisa com 3 botões de 12mm de diâmetro
9. Placa de madeira com 3 laços
10. Agulha com orifício grande e fio
11. Caixa de fósforos grande
12. Porta moedas (com uma mola e fecho éclair)
13. Folha de papel A4
14. Caneta tipo «bic»
15. Régua (22 cm de comprimento)
16. Cartão (1mm de espessura e 20 cm de comprimento)
17. Tesoura
18. Tira de metal de cobre (10/10mm)
19. Pinça corta metal
20. Folhas de jornal (4 dobras nas folhas num total de 32 dobras)

Modo de Aplicação

O terapeuta ocupacional dá sucessivamente as 20 tarefas a realizar, colocando sobre a mesa à frente do paciente, o material necessário para cada um dos itens. Explica-lhe que deve fazer como faz habitualmente, sem lhe dar outros conselhos para além daqueles que vai fornecendo ao mesmo tempo que o material de avaliação:
«Faça como puder, como faria em sua casa neste momento se estivesse sozinho em casa, sem ninguém para o ajudar…»

1- ***«Com os talheres, corte 3 pequenos bocados de pasta de modelar no seu prato como se fosse um beef.»***

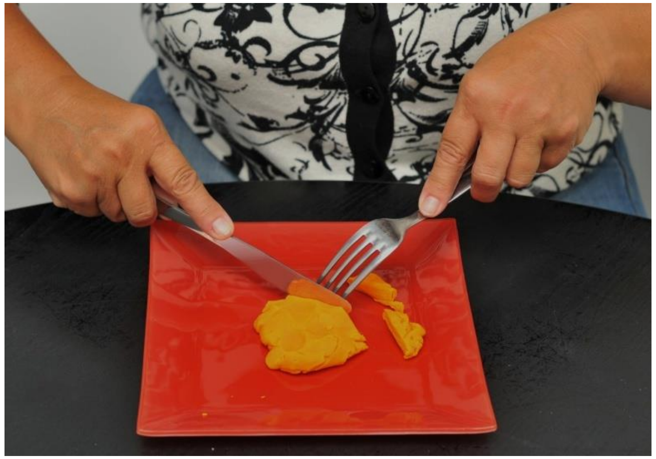

2- ***«Abra esta garrafa de água (500ml) volte a fecha-la, a tampa é de rosca»***

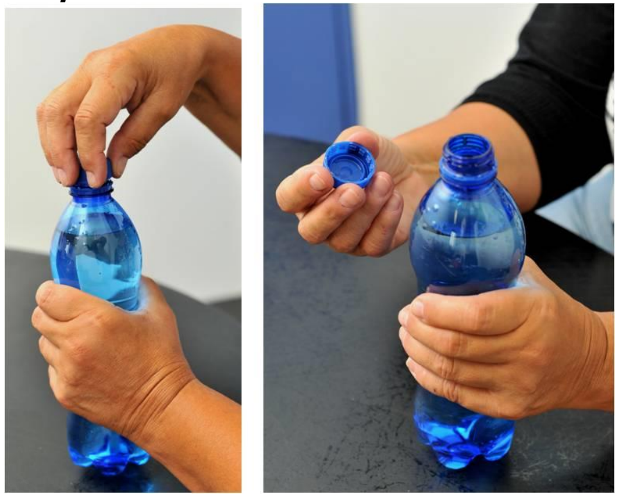

3 - ***«Abra este frasco de compota e volte a fechar»***
(de início, ele está bem fechado)

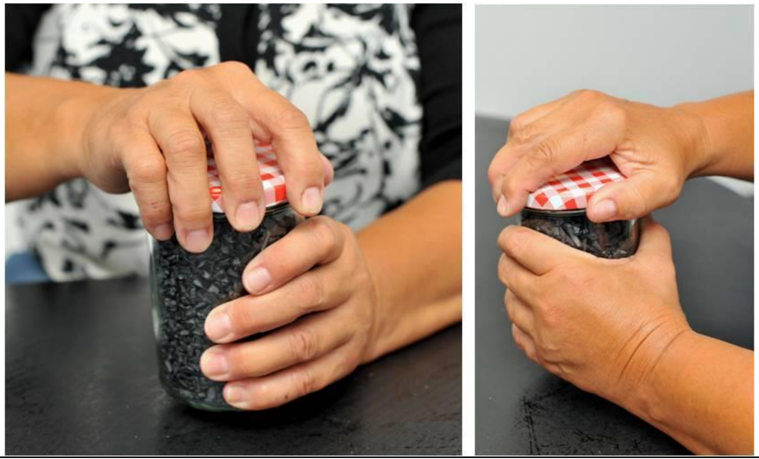

4 - «Destape este tubo e volte a fecha-lo, atenção a tampa não é enroscada.»

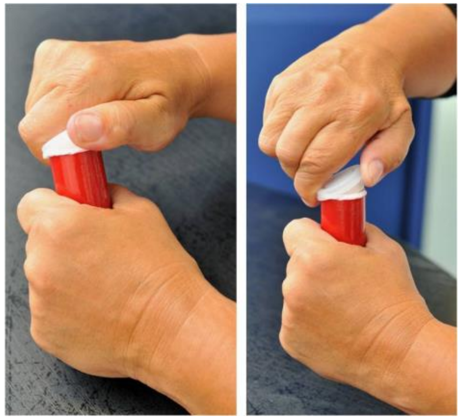

5- «Desabotoe os 3 botões desta camisa.»<br>***6- « Volte a abotoa-los. »***<br>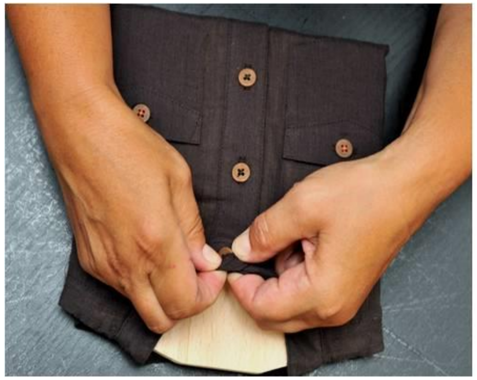 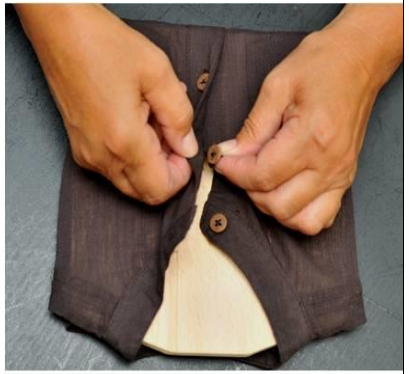	***7- « Desfaça estes 3 laços. »***<br>***8- «Volte a faze-los.»***<br>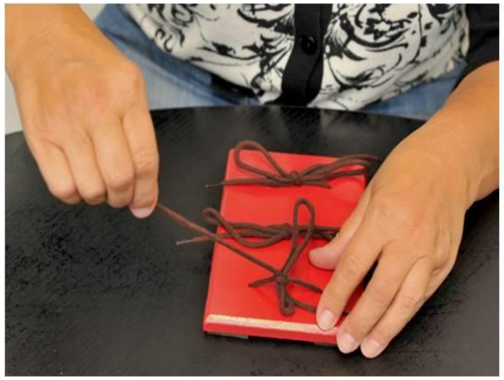 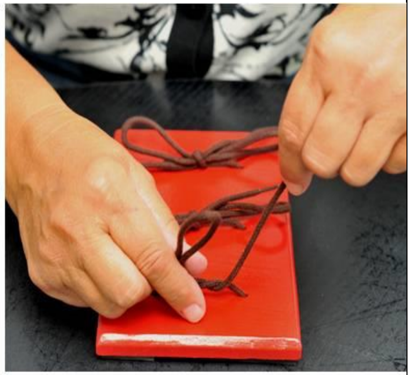
«Desenrosque esta porca e retire-a do parafuso e volte a enrosca-la pelo menos até ao meio»<br>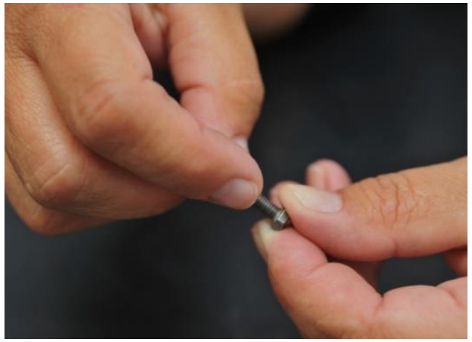	***10- «Enfie a linha nesta agulha.»***<br>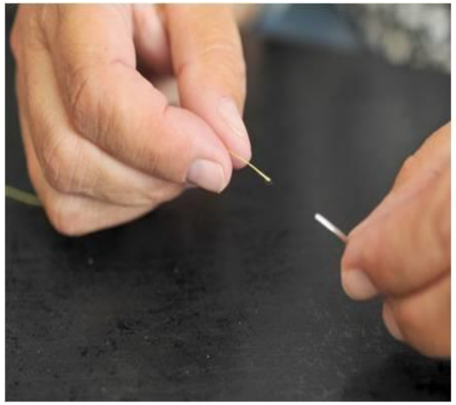 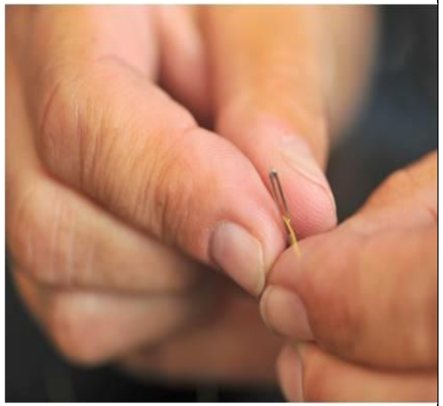

11- «Abra esta caixa de fósforos, tire um, acenda-o» 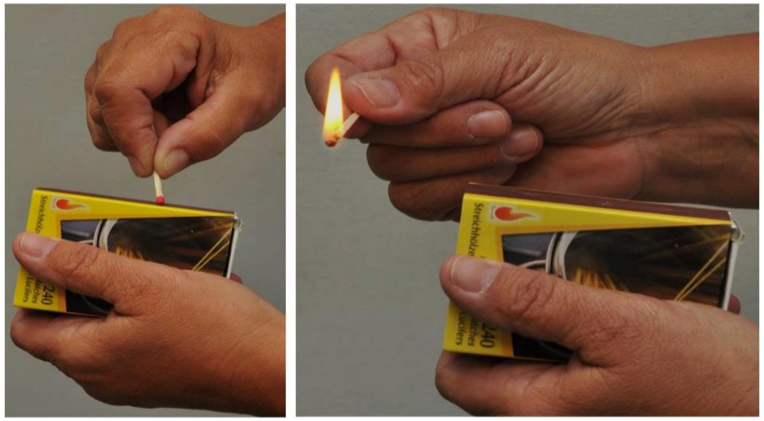	***12- «Apague o fósforo sem soprar, mas sacudindo-o»*** 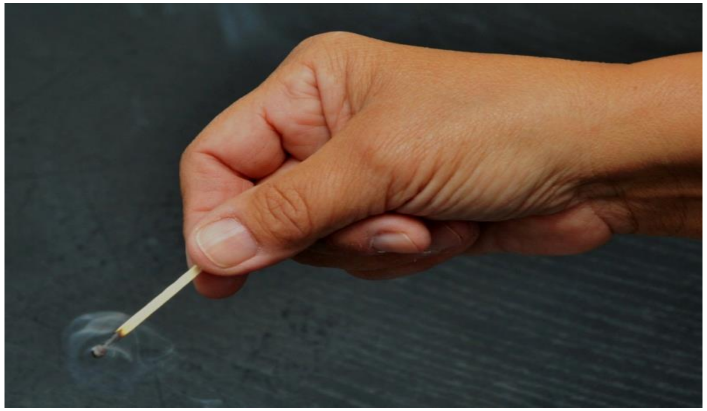
13- «Abra o fecho éclair deste porta moedas, mude as 5 moedas de compartimento e volte a fecha-lo. Abra o porta moedas do lado da mola, tire a nota, desdobre-a, volte a dobrar e coloque-a dentro.» 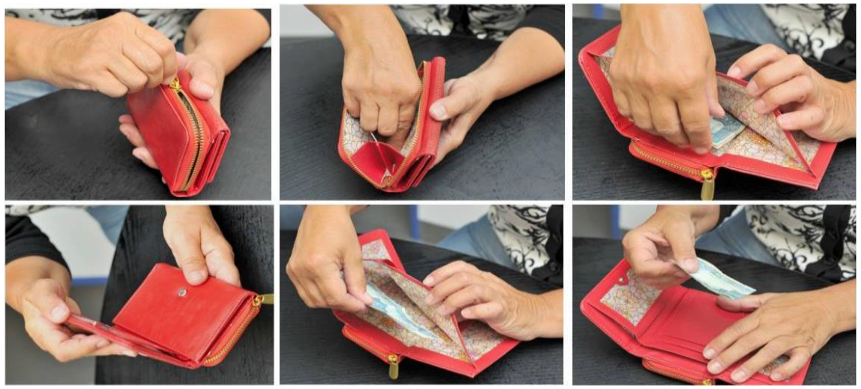	***14- Escreva esta frase:*** a mão é um dos mais belos utensílios do homem e da mulher. 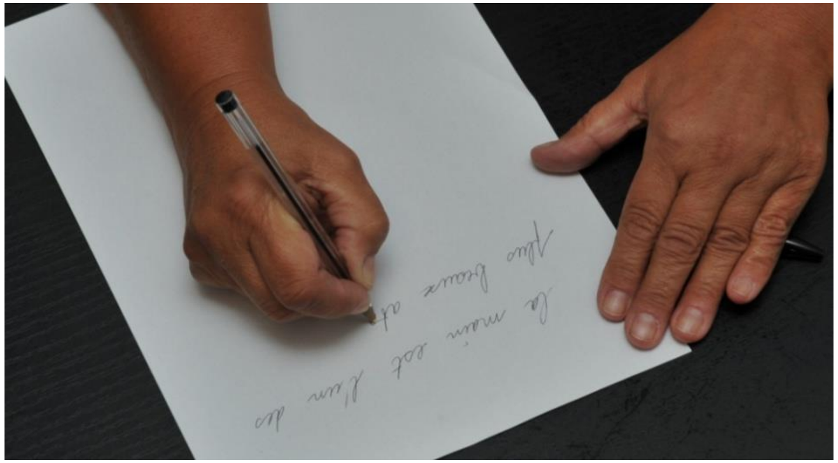

15- «Faça um traço com esta régua.» 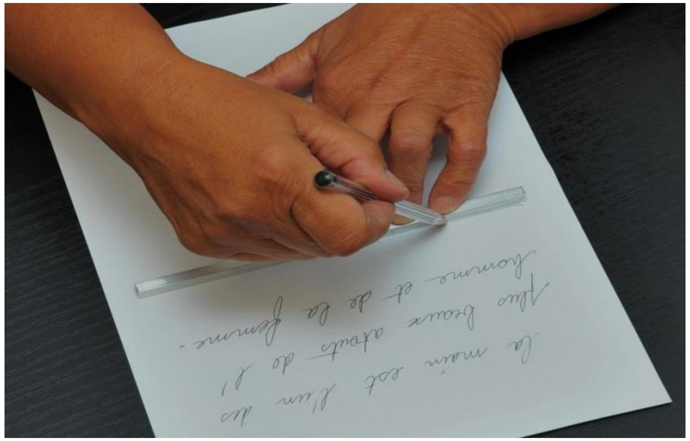	***16- «Dobre a folha sob o traço que fez.»*** 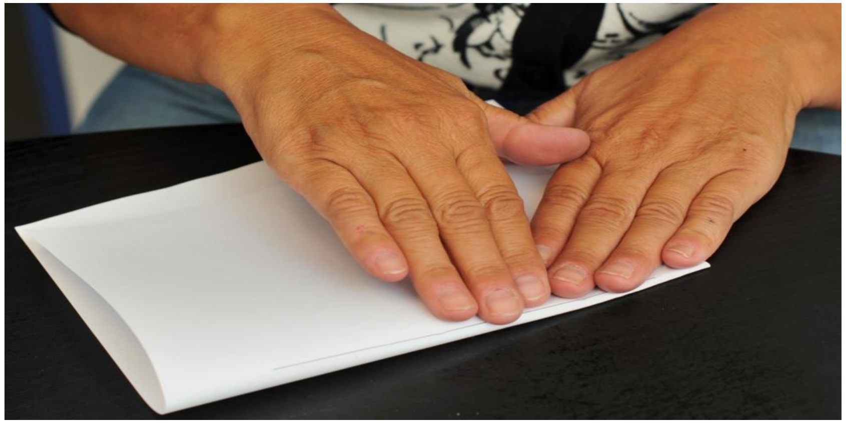
17- «Rasgue a folha ao longo da dobra.» 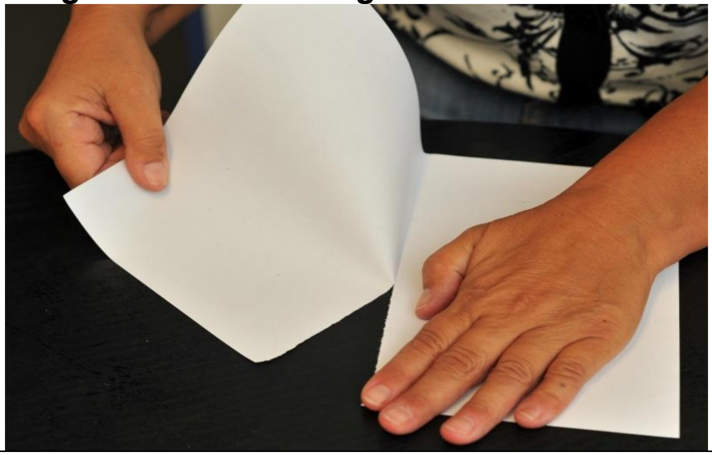	***18- «Corte uma tira de cartão com esta tesoura.»*** 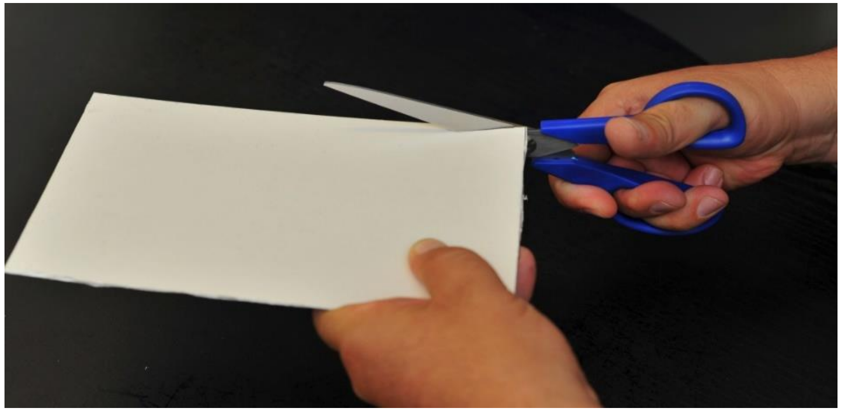

19- «Corte 3 pequenos bocados de metal com esta alicate.»

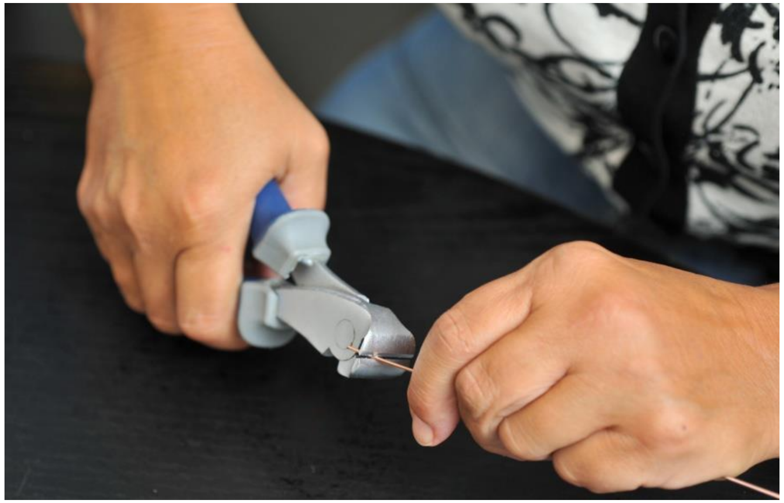

20- «Tente rasgar este jornal ao meio e deste lado.» (4 folhas dobradas, dobradas 3 vezes ou seja 32 espessura de folhas)

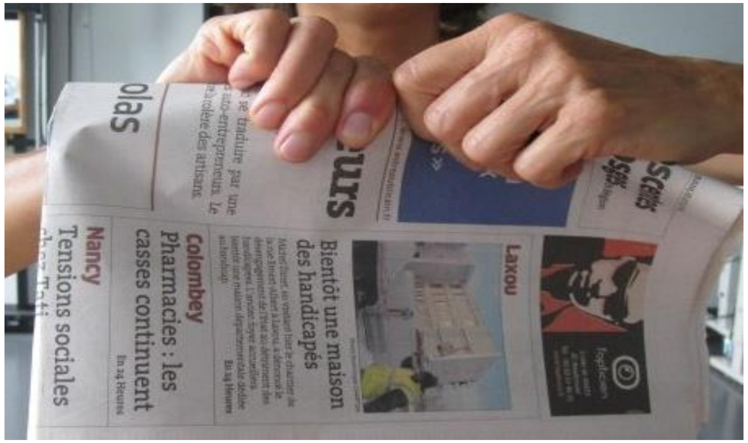

Cotação

COTAÇÃO PROVA 4	GESTO NORMAL*	GESTO COMPLETO, MAS LENTO OU IMPRECISO	GESTO INCOMPLETO OU COM EXCLUSÃO OU COM COMPENSAÇÃO	GESTO IMPOSSIVEL
	**3**	**2**	**1**	**0**

GESTO NORMAL*: no caso de amputação ou artrodeses as compensações indispensáveis são permitidas, conduzindo a uma cotação "gesto normal".

Esta prova serve igualmente para observar a eventual **transferência de dominância** sobretudo quando a mão dominante é a lesada

Por exemplo um paciente destro, que use a mão esquerda para abrir um bocal, mas utilizando bem a sua mão direita para o agarrar será cotado em «3» mas assinalado com um sinal **«-».**
O que significa: «conforme», mas transferência de dominância.
Se a dominância do paciente é respeitada será cotado «3» mas assinalado com um sinal **«+».**

Estas observações dizem respeito à dominância não influenciando nada sobre as notas, simplesmente dão-nos indicações complementares para orientar o seguimento da eventual reeducação

Modo de cálculo

- Cada item é notado de 0 a 3.
- O total das 20 notas é obtido adicionando-os.

- Este resultado é então dividido por 60 (score máximo) e multiplicado por 100 para obter a percentagem de utilização bimanual do paciente em relação a uma pessoa que tenha as duas mãos sãs.

Por exemplo:

- Soma das notas obtidas pelo paciente = 47 pontos
- Score máximo = 60 pontos

- Far-se-á: $\frac{47}{60} \times 100 = 78{,}3 / 100$

Atenção!!!

Aquando do cálculo do resultado final, é conveniente arredondar para baixo quando a casa decimal é inferior ou igual a 5 e arredondar para cima quando a casa decimal é superior ou igual a 5.

- Assim, 78,3 ficará **78/100.**

Interpretação: Podemos dizer que o paciente tem uma função bimanual de 78% em relação a uma pessoa que tenha todas as suas capacidades

MODO DE CALCULO GLOBAL DA AVALIAÇÃO

O score global é obtido fazendo a soma dos resultados obtidos em cada uma das 4 provas.

Exemplo:

- Prova de mobilidade da mão = 77
- Prova de força de preensão = 52
- Prova monomanual = 77
- Prova bimanual = 78

faremos: 77 + 52 + 77 + 78 = **284 pontos / 400 pontos (score máximo)**

Podemos igualmente dividir este resultado por 4, para termos um valor para 100 pontos ou percentagem mais fácil de reter: 284 será 284/4 = **71 %**

Estes resultados podem ser registados num histograma para que a leitura se torne agradável e fácil também para os pacientes e diferentes intervenientes da equipa clínica.

Interpretação: podemos dizer que a capacidade de utilização funcional da mão lesada do paciente é de 71% em relação a uma mão sã ou que ela está diminuída em 29% em relação a uma mão sã.

A Avaliação 400 Pontos objetiva a evolução do paciente, realçando os progressos ou pelo contrário reforçando a necessidade de continuar esforços.
Para além da avaliação global, são acentuados os setores particularmente deficitários, permitindo de reajustar melhor o tratamento de reeducação ou orientar para um tratamento cirúrgico.

MATERIAL NECESSÁRIO

Prova 1

- Uma cunha triangular é utilizada para os 3 itens com resistência
- Uma carta tipo carta de jogar ou cartão bancário
- Três cilindros de 22 cm de comprimento:
    - um de 3 mm de diâmetro
    - um de 10 mm de diâmetro
    - um de 20 mm de diâmetro

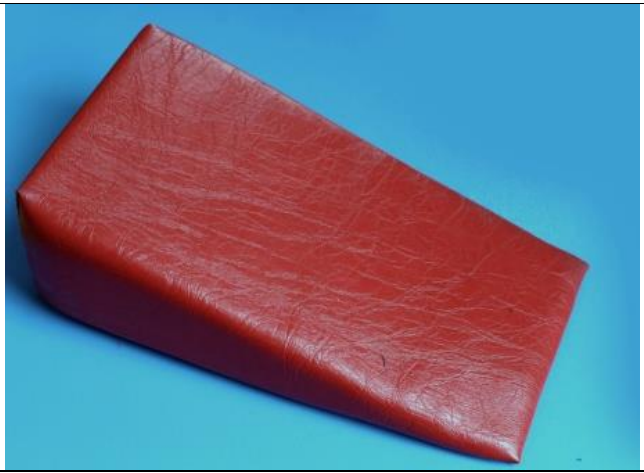	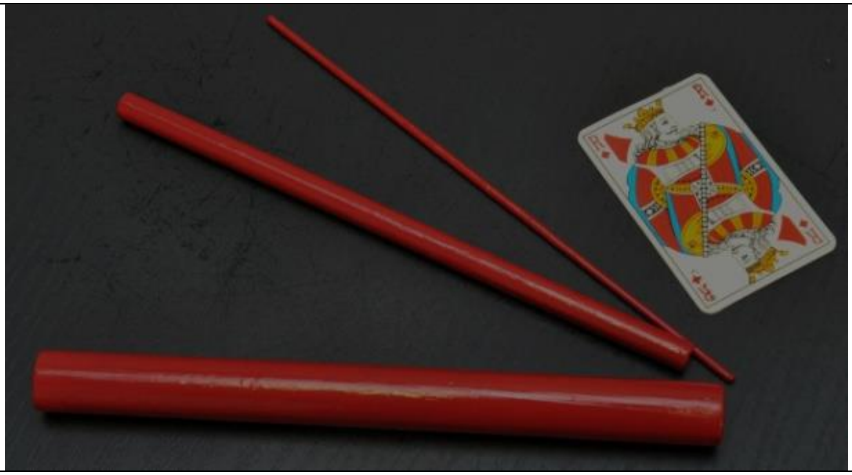

Prova 2

- Um Dinamométro Jamar
- Um Pinchmeter Jamar

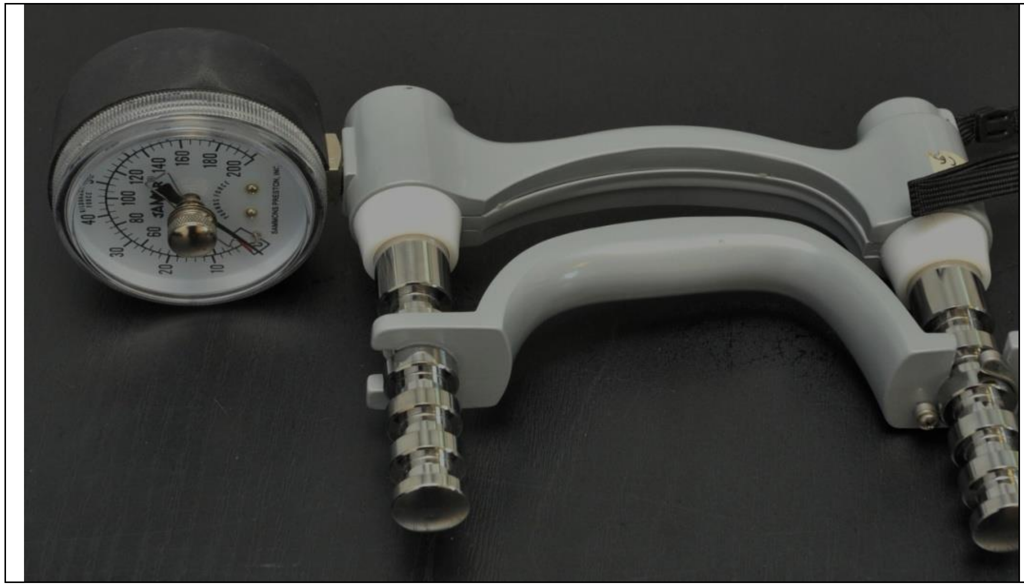

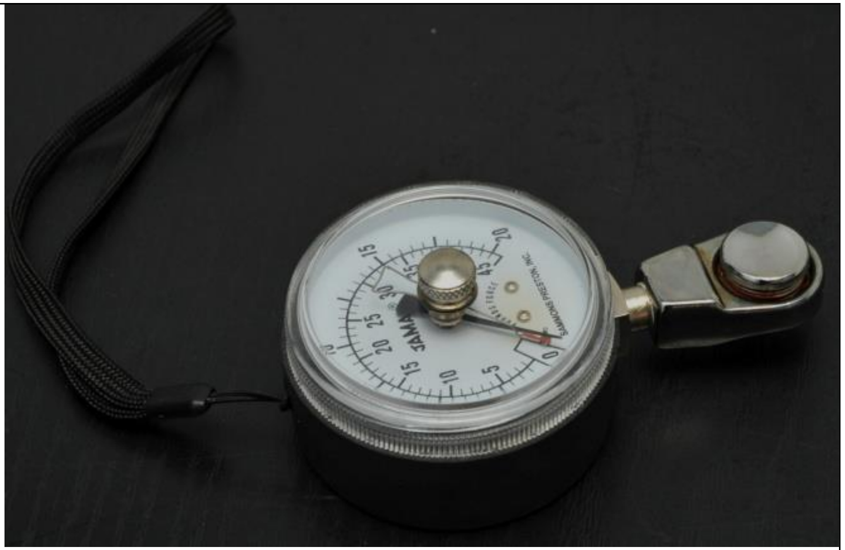

Prova 3

Um cronometro

Um cubo de 10 cm de lado (700gr)
Um cubo de 7,5 cm de lado (300 gr)
Um cubo de 5 cm de lado (100 gr)
Um cubo de 2,5 cm de lado (10 gr)

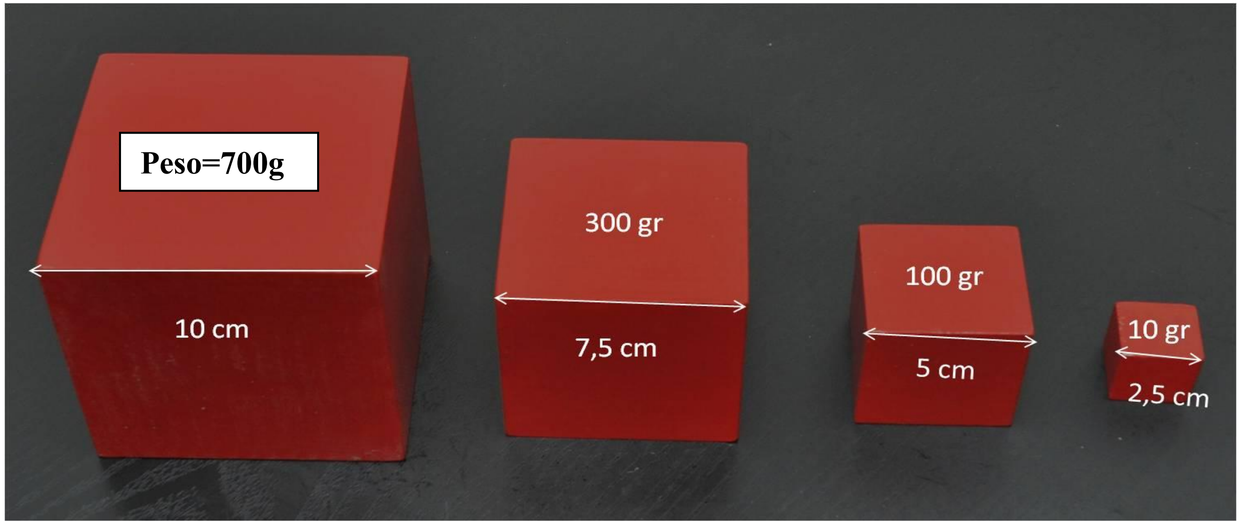

Um cilindro de 10 cm de diâmetro e 12 cm de altura (700 gr)
Um cilindro de 7,5 cm de diâmetro e 11 cm de altura (300 gr)
Um cilindro de 5 cm de diâmetro e 10 cm de altura (100gr)
Une berlinde de 25 mm de diâmetro

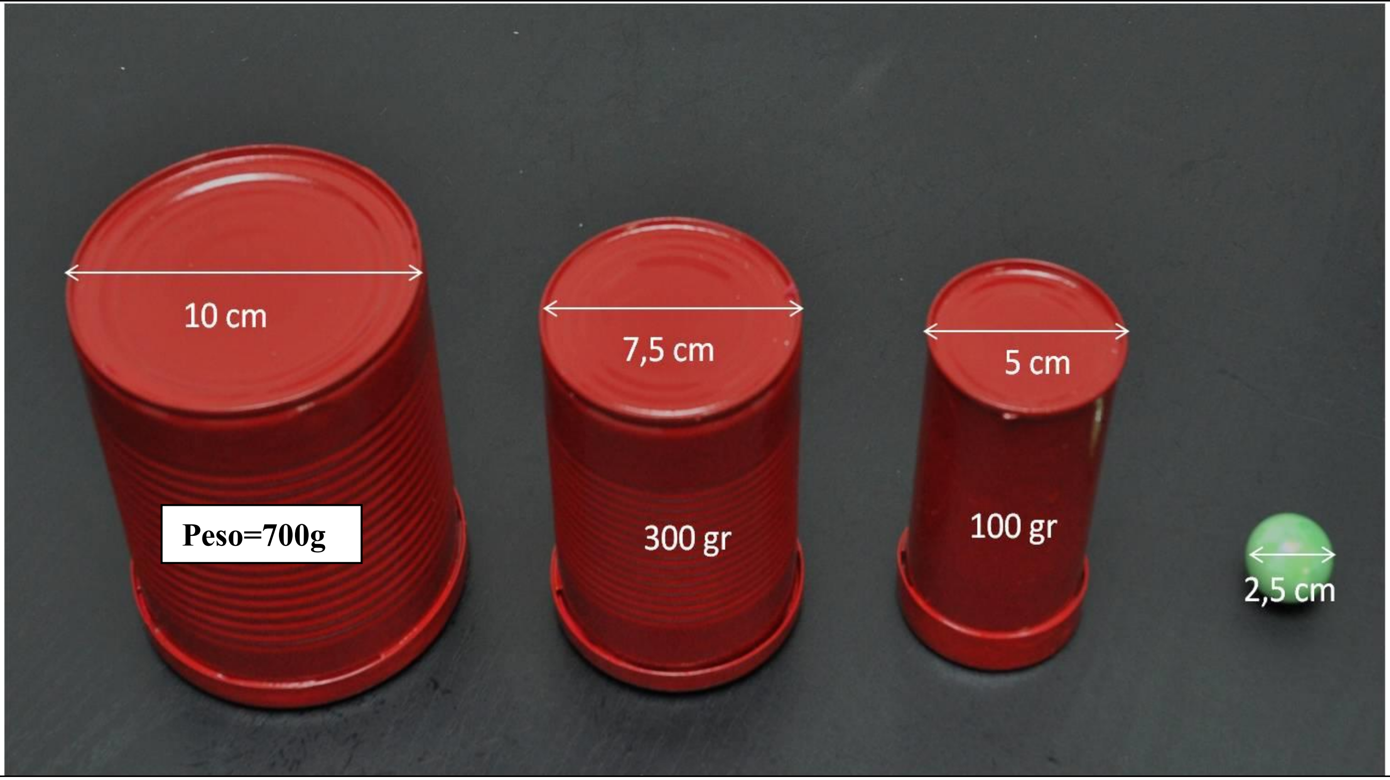

Uma bola de ténis
Uma moeda de 30 mm de diâmetro
Uma moeda de 25 mm de diâmetro
Uma moeda de 15 mm de diâmetro
Um berlinde de 16 mm de diâmetro
Um prego de 4 cm de comprimentos e 2 mm de diâmetro
Um isqueiro eletrónico

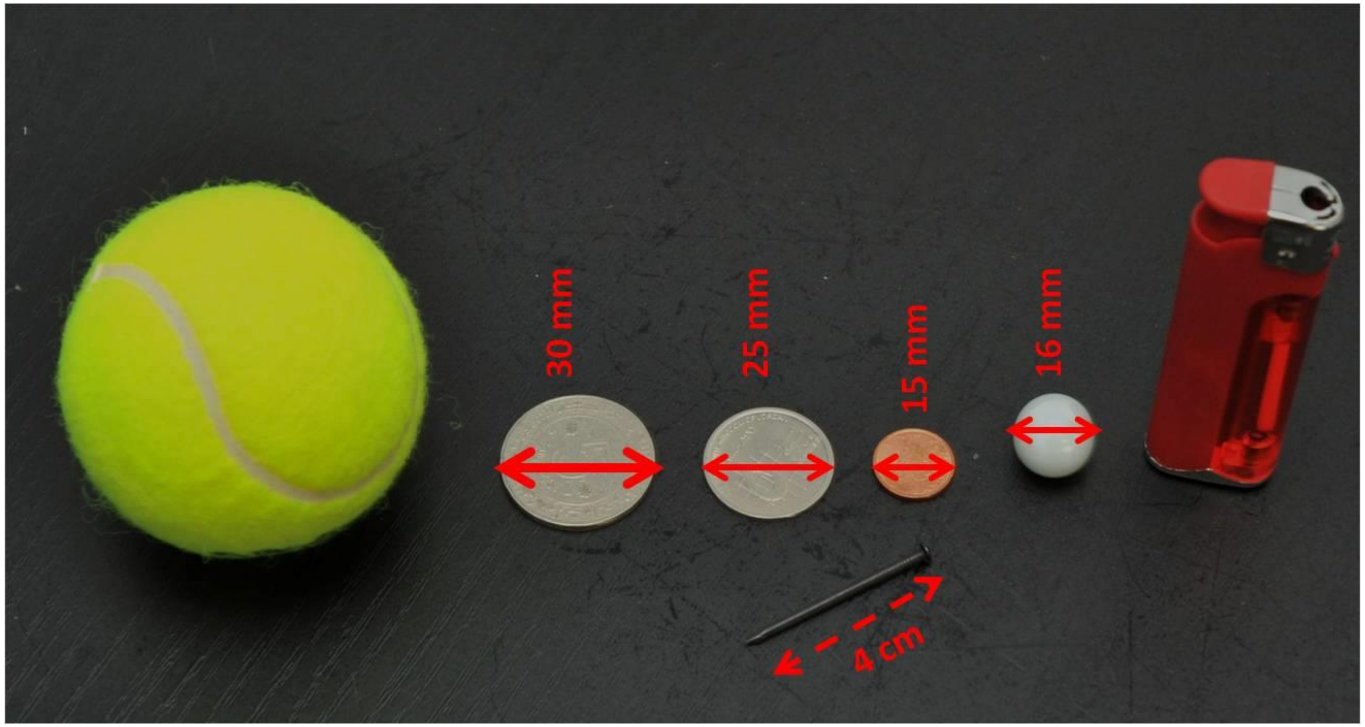

Uma chave achatada e a fechadura correspondente e um ferro de engomar de 2kg

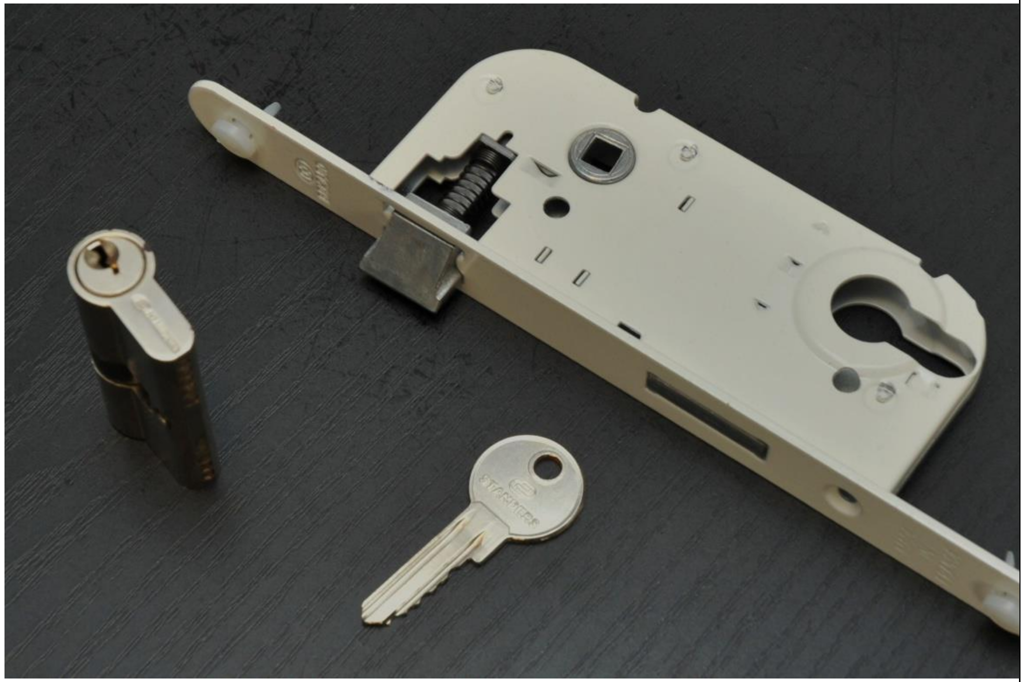	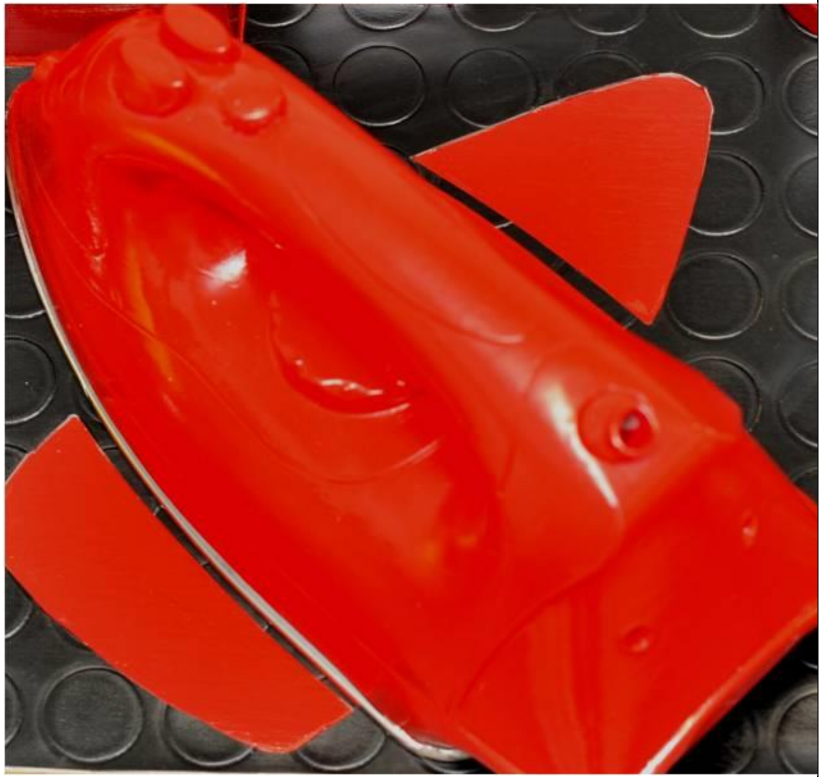

Um jarro com dois bicos para deitar a água com, com 500ml de água
Um copo vulgar

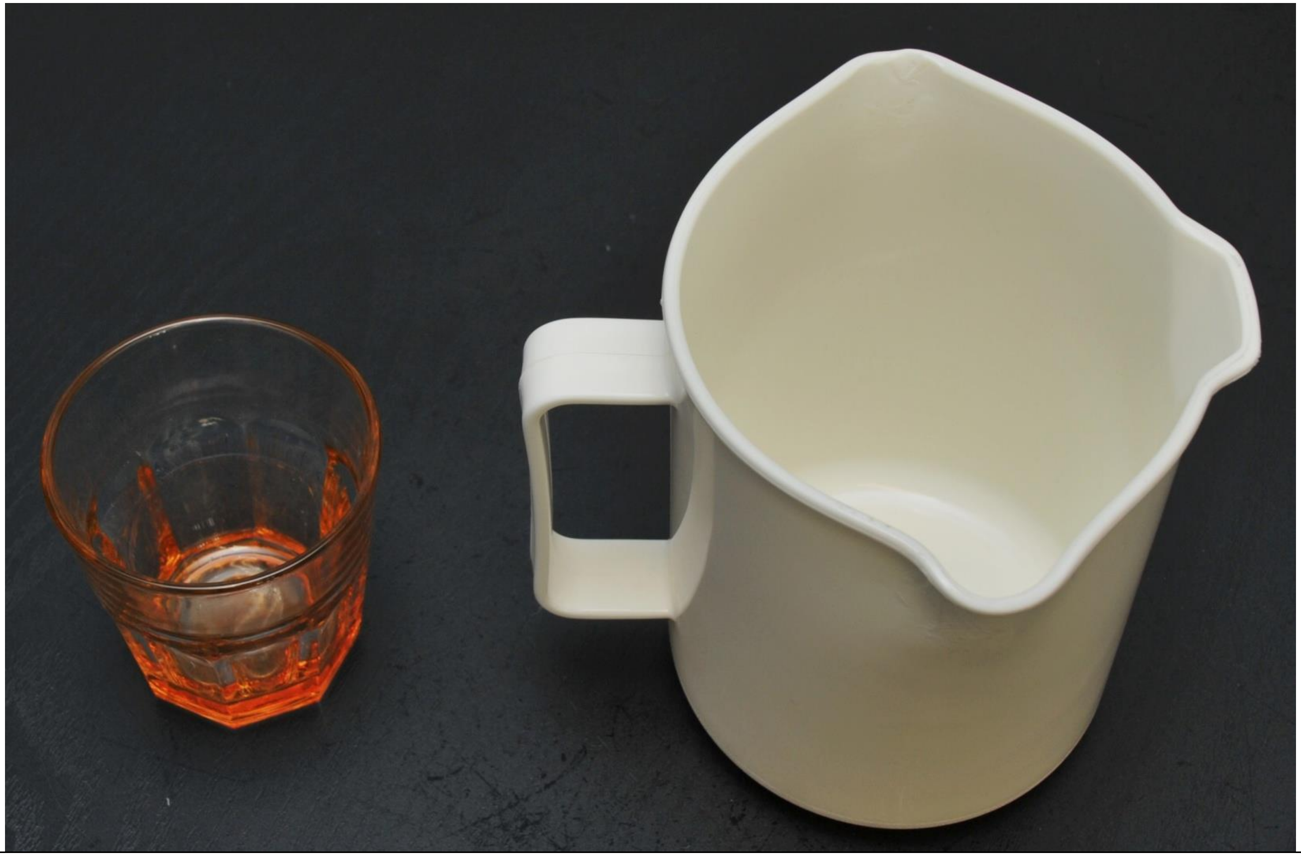

Prova 4

Um prato e massa de modelar, assim como talheres de uso corrente

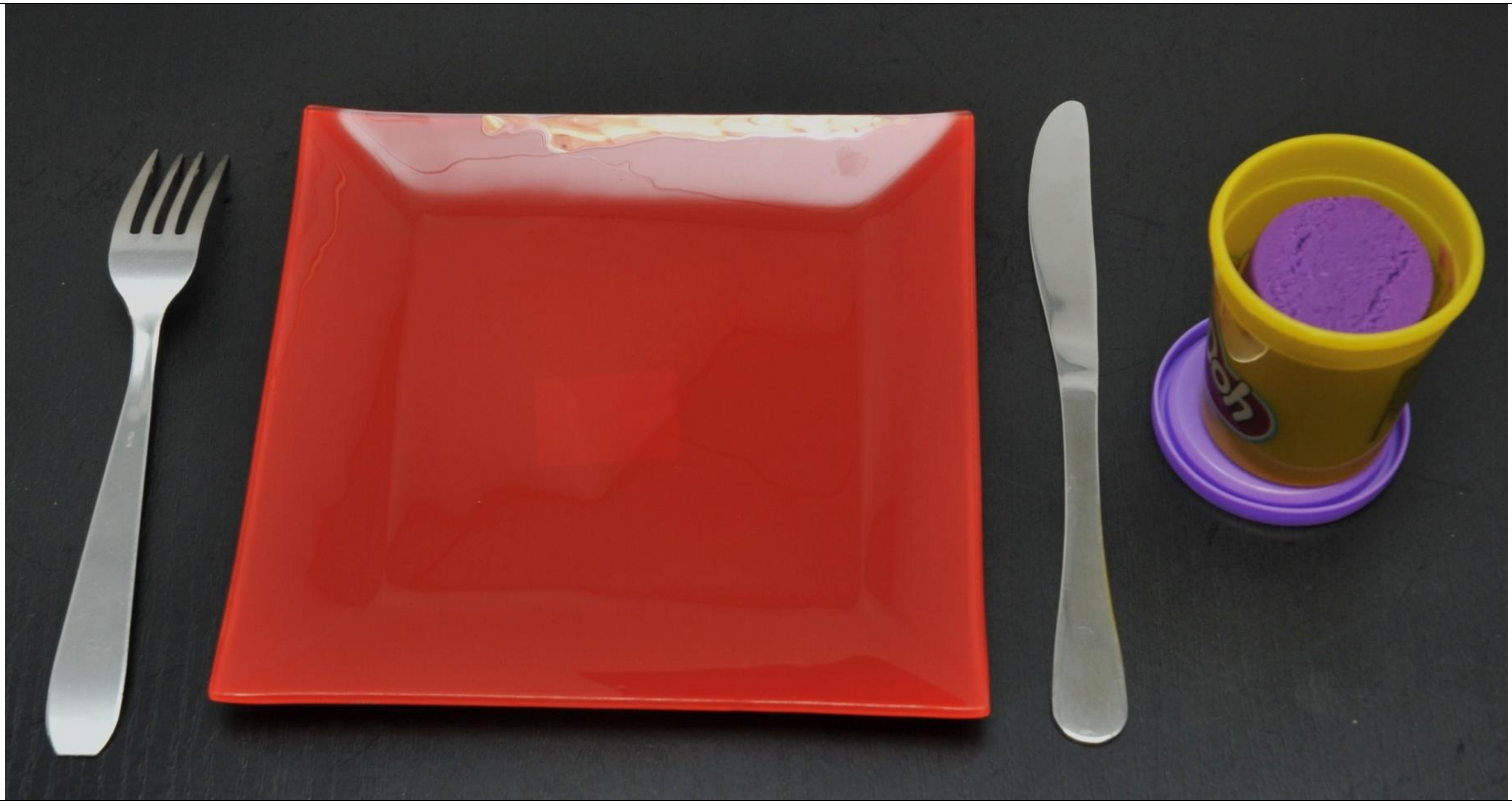

Um frasco de compota (cheio de partículas e tampa bem enroscada);
Uma garrafa de água (50 ml) com tampa de rosca
E um tubo de medicamento com tampa sem rosca

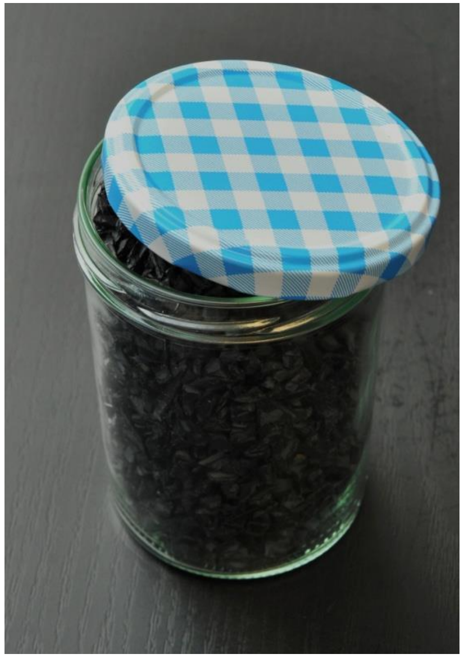

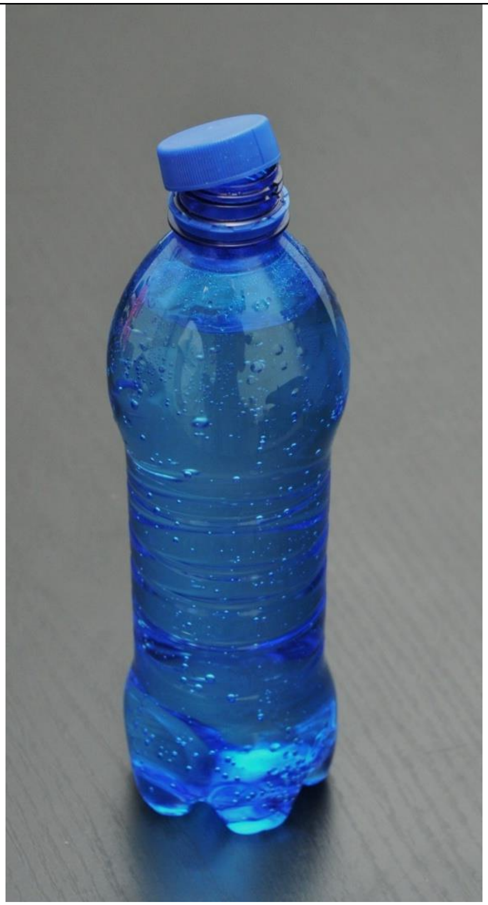

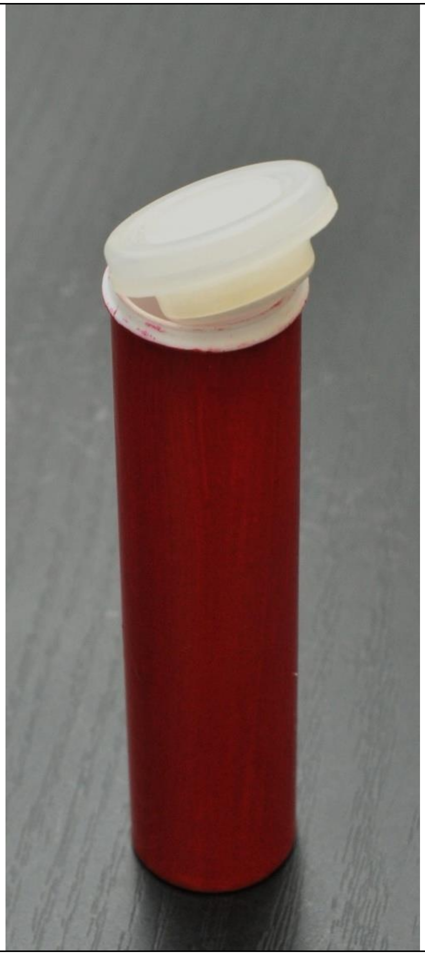

Um parafuso e porca de 4mm
Uma caixa de fósforos grande

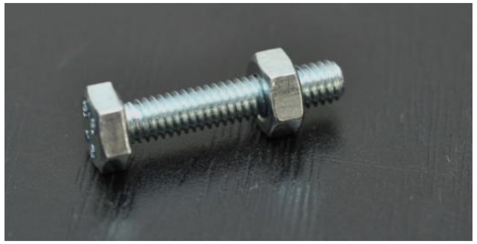

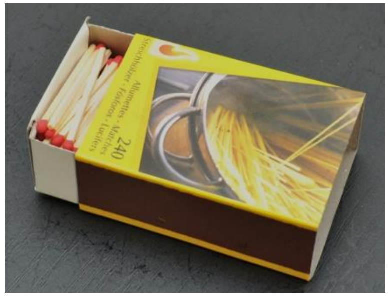

Uma camisa sobre uma prancha com 3 botões de 12mm de diâmetro e uma tábua com 3 laços
Uma agulha com buraco grande e linha

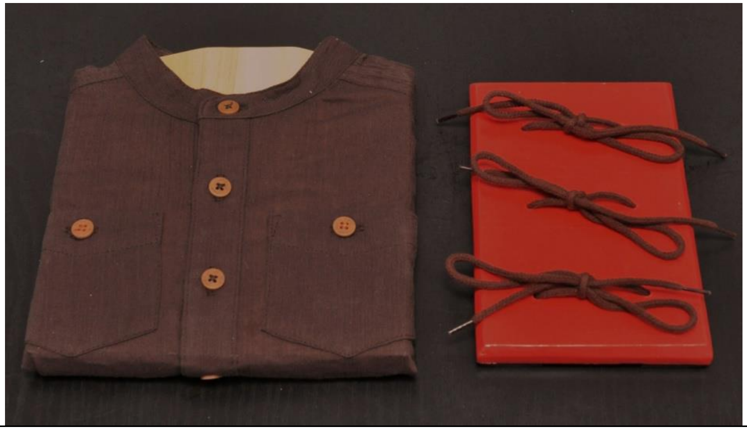

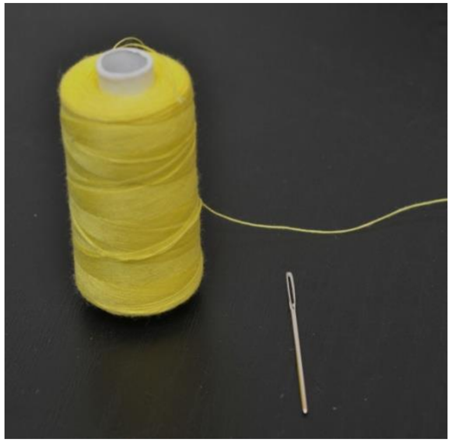

Um porta moedas (com botão de pressão e fecho éclair)

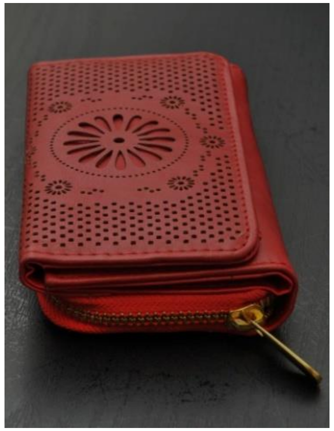

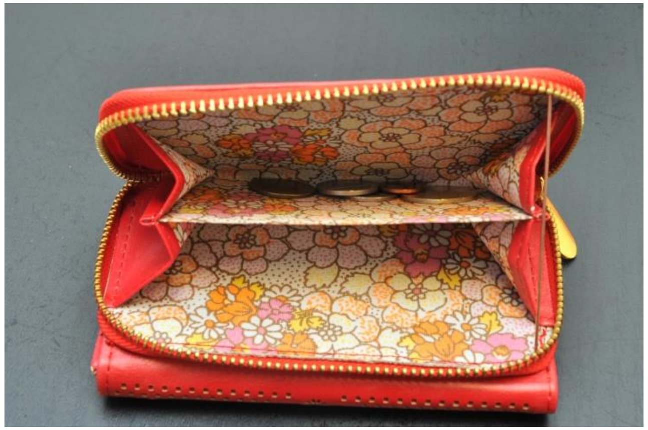

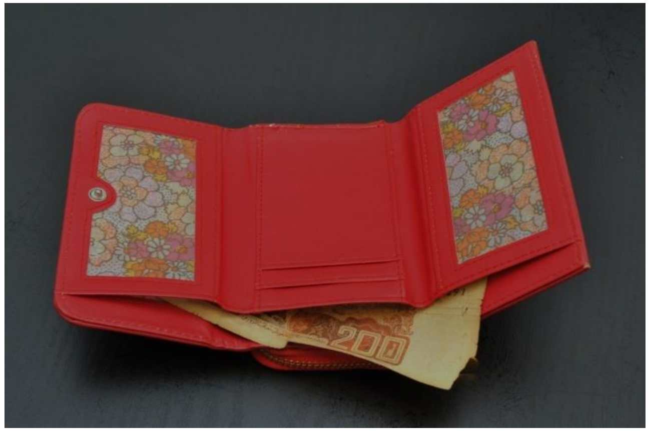

Uma folha de papel A4 e uma esferográfica «bic» e uma régua quadrara (22 cm de comprimento)
Folhas de jornal (4 dobras de folhas)

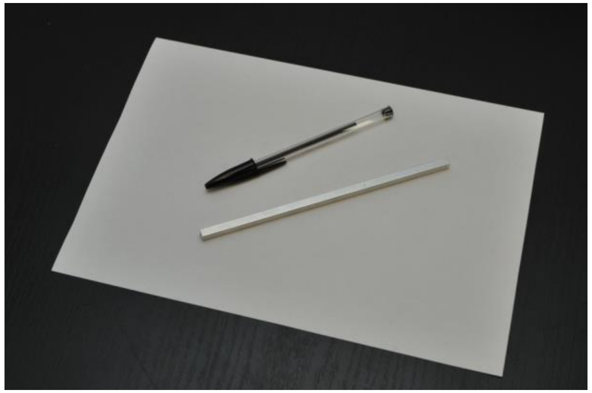

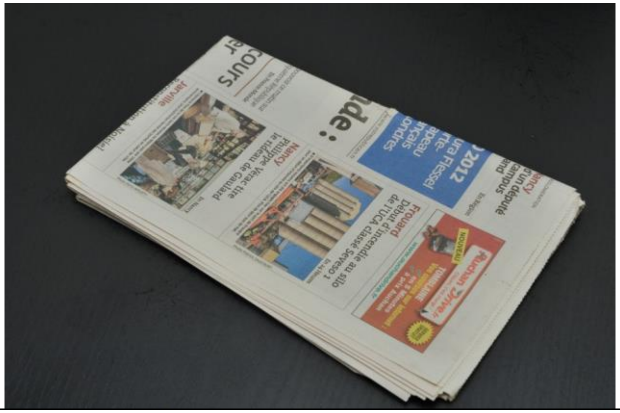

Cartão (1mm de espessura e 20 cm comprimento) e uma tesoura
Uma tira de metal de cobre (10/10mm) assim como uma pinça de cortar metal

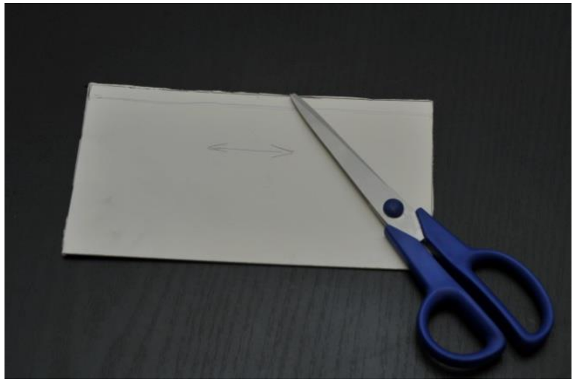	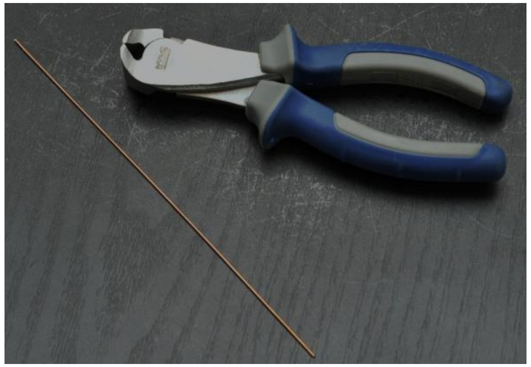
