# Supplementary material for: Prospective multicentre validation study of a new standardised version of the 400-point hand assessment
Source: BMC Musculoskelet Disord. 2020 May 20;21:313. doi: 10.1186/s12891-020-03303-4 (PMC7240941; doi:10.1186/s12891-020-03303-4)
Supplement: Supplementary file 4 — Additional file 4. [file 12891_2020_3303_MOESM4_ESM.pdf]

Supplementary material 4: patient's characteristics in the four centres

	Nancy	Sion	Saint Martin d'Hères	Faro
**Number of patients**	66	58	32	20
**Men**	41 (62.1%)	43 (74.1%)	26 (81.2%)	8 (40%)
**Women**	25 (37.9%)	15 (25.9%)	6 (18.8%)	12 (60%)
**Age (years)**<br>Mean +- SD	45.08 +- 13.01	42.40 +- 11.87	43.39 +- 16.38	48.26 +- 11.98
**Education formation**				
< 9 years	9 (13.6 %)	34 (58.6%)	2 (6.2%)	5 (25%)
>9 years	57 (86.4 %)	24 (41.4%)	30 (93.8%)	15 (75%)
**Employment status**				
Employee	53 (80.3%)	45(77.6%)	22 (68.8%)	15 (75%)
Unemployment	6 (9.1%)	13 (22.4%)	3 (9.4%)	3 (15%)
Retired	6 (9.1%)	0	5 (15.6%)	1 (5%)
Student	0	0	2 (6.2%)	0
Housewife	1 (1.5%)	0	0	1 (5%)
**Occupation**				
Worker/farm worker	24 (37.5%)	41 (70.7%)	12 (37.5%)	3 (17%)
Employee (office)	34 (53.1 %)	16 (27.6 %)	16 (50%)	14 (78%)
Manager/self employed	2 (3.1%)	1 (1.7%)	2 (6.25%)	1 (5%)
Other	4 (6.2%)	0	2 (6.25%)	0
**Work capacity**				
0%	61 (92.4%)	52 (89.7%)	23 (71.9%)	7 (36.8%)
Partial	2 (3%)	5 (8.6%)	0	1 (5.2%)
Complete	3 (4.6%)	1 (1.7%)	9 (28.1 %)	11 (57.9%)
**Diagnostic**				
Fracture hand/wrist	27 (40.9%)	24 (41.4%)	6 (18.8%)	8 (40%)
Sprain/luxation	3 (4.6%)	12 (21.7%)	0	3 (15%)
Isolated tendon	9 (13.6%)	9 (15.5%)	4 (12.5%)	1 (5%)
Complex lesion (> 3 structures)	11 (16.7%)	3 (5.2%)	12 (37.5%)	0
Others	16 (24.2%)	10 (17.2%)	10 (31.2%)	8 (40%)
**CRPS**				
Yes	24 (36.4%)	25 (43.1%)	0	1 (5%)
No	42 (63.6%)	33 (56.9%)	32 (100%)	19 (95%)
**Interval between injury and T0**				
Days mean+- SD	311 +- 311	563 +- 558	307 +- 508	124 +- 75
**Number of surgery**				
median +- SD	1.45 +- 1.07	1.60 +- 1.41	2.19 +- 1.15	0.65 +- 0.49
**Interval between last surgery and T0**<br>Days mean +-SD	177 +- 223	240 +- 144	79 +- 61	121 +- 103
**Pain at T0 before test**<br>median+- SD	1.55 +- 1.95	3.40 +- 2.44	1.09 +- 1.49	2.25 +- 2.38
Average pain	2.94 +- 1.97	4.25 +- 1.95	1.94 +- 1.44	4.35 +- 2.41
Maximum pain	4.61 +- 2.55	6.23 +- 2.52	3.91 +-2.84	6.05 +- 2.84
**Pain at T3 before test**<br>median+- SD	1.39 +- 1.75	3.79 +- 2.48	1.06 +- 1.26	1.5 +- 1.76
Average pain	2.55 +- 1.82	4.44 +- 2.18	2.19 +- 1.82	3.22 +- 2.13
Maximum pain	3.92 +- 2.66	6.46 +- 2.41	3.94 +- 2.87	4.28 +- 2.72
**Quick DASH/100**<br>median +- SD				
T0	42.18 +- 19.94	59.02 +- 15.88	44.43 +- 17.15	39.33 +- 24.13
T3	37.56 +- 19.29	48.16 +- 19.03	38.20 +- 17.10	30.56 +- 18.65
**400- point HA (in %) at T0**<br>median +- SD				
**Total score**	**52.80 +- 16.05**	**53.56 +- 18.95**	**68.98 +- 13.83**	**81.88 +- 13.08**
Test n°1	59.25 +- 14.96	58.39 +- 16.22	65.57 +-14.72	83.05 +- 13.36
Test n°2	47.31 +- 22.11	46.52 +- 26.74	46.39 +- 21.87	73.58 +-25.09
Test n°3	49.65 +- 20.93	52.41 +- 24.99	78.85 +-18.69	84.75+-15.46
Test n°4	55.0 +- 18.33	56.90 +- 21.30	85.10 +-9.67	86.17 +-9.16
**400-point HA (in %) at T3**<br>median +- SD				
**Total score**	**63.72 +- 16.15**	**64.83 +- 20.89**	**75.77 +- 12.75**	**89.70 +- 9.47**
Test n°1	66.31 +- 15.10	66.18 +- 16.70	74.38 +- 14.68	90.69 +- 11.10
Test n°2	53.16 +- 21.96	54.72 +- 27.26	53.10 +- 24.07	77.48 +- 19.97
Test n°3	66.04 +- 20.33	69.24 +- 26.20	85.75 +- 12.92	95.56 +- 9.31
Test n°4	69.37 +- 18.46	69.18 +- 23.77	89.84 +- 10.49	95.09 +- 5.95

Supplementary material 4: patient's characteristics in the four centres
